# Gray Matter Is Targeted in First-Attack Multiple Sclerosis

**DOI:** 10.1371/journal.pone.0066117

**Published:** 2013-09-10

**Authors:** Steven E. Schutzer, Thomas E. Angel, Tao Liu, Athena A. Schepmoes, Fang Xie, Jonas Bergquist, László Vécsei, Denes Zadori, David G. Camp, Bart K. Holland, Richard D. Smith, Patricia K. Coyle

**Affiliations:** 1 Departments of Medicine, and Epidemiology and Biostatistics, Rutgers University New Jersey Medical School, Newark, New Jersey, United States of America; 2 Biological Sciences Division, Pacific Northwest National Laboratory, Richland, Washington, United States of America; 3 Department of Chemistry-Biomedical Center, Analytical Chemistry and SciLife Lab, Uppsala University, Uppsala, Sweden; 4 Faculty of Medicine, Albert Szent-Györgyi Clinical Center and Neuroscience Research Group of the Hungarian Academy of Sciences and University of Szeged, Szeged, Hungary; 5 Department of Neurology, Stony Brook University, Stony Brook, New York, United States of America; University of Utah School of Medicine, United States of America

## Abstract

The cause of multiple sclerosis (MS), its driving pathogenesis at the earliest stages, and what factors allow the first clinical attack to manifest remain unknown. Some imaging studies suggest gray rather than white matter may be involved early, and some postulate this may be predictive of developing MS. Other imaging studies are in conflict. To determine if there was objective molecular evidence of gray matter involvement in early MS we used high-resolution mass spectrometry to identify proteins in the cerebrospinal fluid (CSF) of first-attack MS patients (two independent groups) compared to established relapsing remitting (RR) MS and controls. We found that the CSF proteins in first-attack patients were differentially enriched for gray matter components (axon, neuron, synapse). Myelin components did not distinguish these groups. The results support that gray matter dysfunction is involved early in MS, and also may be integral for the initial clinical presentation.

## Introduction

The cause of Multiple Sclerosis (MS) [1], its driving pathogenesis at the earliest stages, and how an area of the brain or spinal cord might be affected for the first clinical attack to manifest itself remain unknown. The first attack is a critical time-point to study in MS, since patients can be offered disease-modifying therapies.

The most common MS clinical subtype is relapsing remitting MS (RR-MS), characterized by discrete attacks resulting in neurologic deficits. This is how 85% of MS patients present, with the first attack considered a clinically isolated syndrome (CIS) [2]. Many, but not all CIS-like attacks, turn out to be MS. The majority of patients are women. Compared to men the disease occurs two to three times more frequently in females and is and is on the rise among young women [3].

Some imaging studies suggest gray rather than white matter changes occur early, and predict the development of MS but other imaging studies are in conflict [2], [4].

Cerebrospinal fluid (CSF) is an important body fluid to examine in MS because recent evidence suggests cell processing within the central nervous system (CNS) is a crucial component to the damage process. Meningeal and subarachnoid inflammation have been associated with cortical lesion development in very early MS patients [5], [6]. CSF is known to reflect the CNS microenvironment, and is already used to document presence of suggestive (although not conclusive) diagnostic immune abnormalities [7].

Mass spectrometry (this term is spelled out or if preceded by LC or if referring to tandem mass spectrometry it appears as *MS*, *italicized* this to distinguish it from the disease multiple sclerosis which is abbreviated as MS non-italicized) based proteomics offers an effective tool to evaluate CSF proteins. Using advanced proteomic techniques, we have previously examined CSF collected from healthy controls [8], and two disease groups with confounding symptoms, chronic fatigue syndrome (*CFS*) and neurologic post treatment Lyme disease syndrome (*nPTLS*) [9]. The proteomic results permitted separation of one disease from another. With high abundant protein depletion, liquid chromatographic (LC) peptide fractionation, and sensitive mass spectrometry detection, we identified 2,630 nonredundant proteins in normal CSF [8]. This has been the most comprehensive CSF protein analysis to date, reflecting the great sensitivity of our methods.

In the current study we examined CSF collected during an attack from the earliest identifiable MS time-point. CIS patients were confirmed as first-attack MS patients because they eventually met criteria for MS [10]. We compared the proteomic results to those from established RR-MS patients and controls (no overt neurologic disease). The goal was to determine whether the first-attack patients would have CSF proteins that could provide objective evidence to support or refute gray matter involvement in early MS.

## Results

Our CSF proteome analysis of first-attack MS patients, using two separate patient sets in multiple replicates, identified proteins that distinguished these patients from both established RR-MS and controls. The data provides credible evidence that gray matter is likely involved early in the MS process.

**Figure 1:**
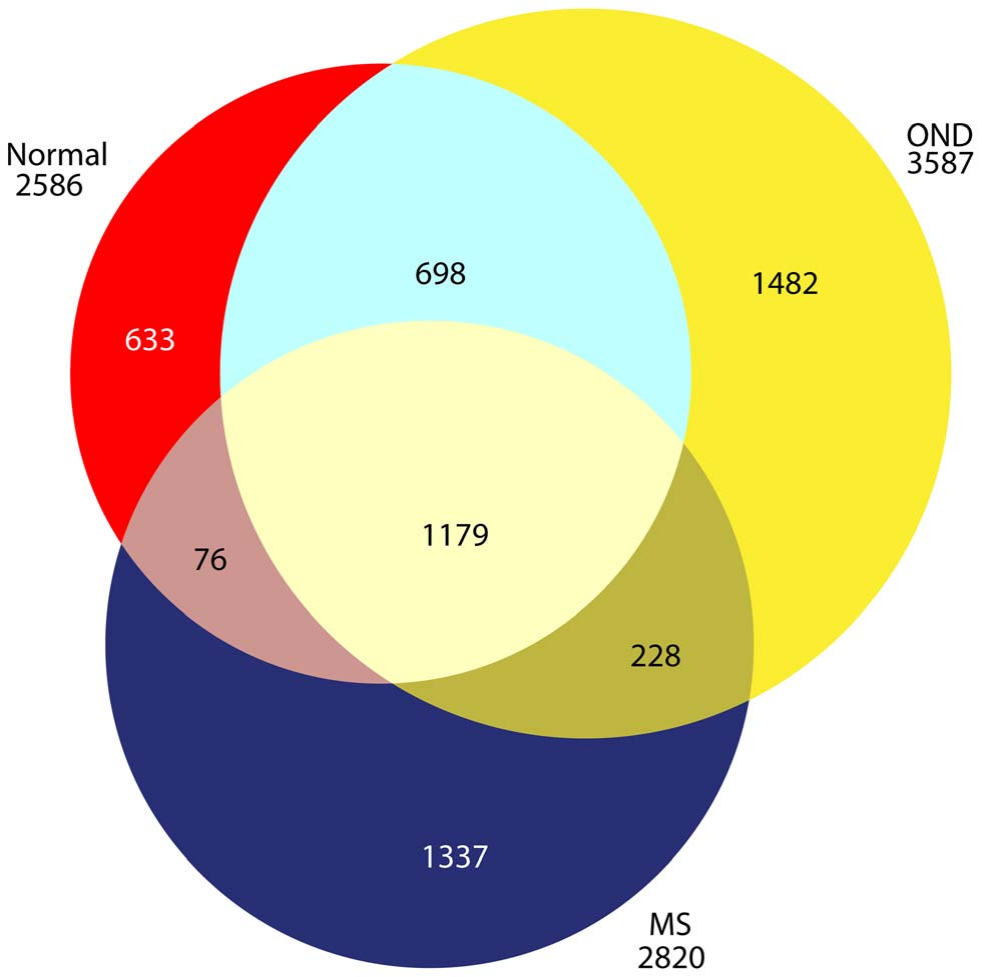
In-depth off-line 2D-LC-*MS/MS* analysis of the CSF proteome of a pooled sample composed of CSF from all MS patients resulted in the identification of 2820 proteins, and the comparison to previous results obtained from analyses of healthy normals [8] and other neurologic disease (OND) [9].

To gain a broad picture of what informative proteins are detectable in the CSF samples of MS, we first performed an in-depth analysis of the pooled immunoaffinity depleted CSF samples from all first-attack CIS MS plus established RR-MS patients (both the flow-through and bound immunoaffinity depletion fractions were subjected to offline 2D-LC-*MS*/*MS* analysis). We compared the results to CSF analysis from our published pooled healthy normals and pooled other neurologic diseases (ONDs) (i.e., *CFS* and *nPTLS*). We identified 2,820 proteins in MS CSF (Table S1), compared to 2,586 proteins in normal CSF and 3,587 proteins in OND CSF (**Figure 1**). There were 1,337 proteins unique to MS CSF, 633 proteins unique to healthy normal CSF, and 1,482 proteins unique to OND CSF.

**Figure 2:**
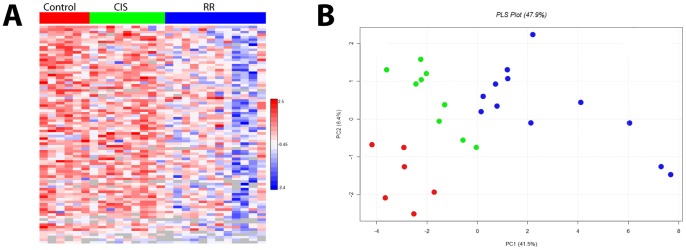
Label-free quantification of CSF proteins identified in patient and control samples. A) Following the 1D LC-MS analysis of immunodepleted CSF samples we identified peptides referable to 86 proteins that show significant difference in abundance by ANOVA (p-value <0.05) (Table S2). B) Partial least squares analysis of quantified proteins demonstrates that these three groups (control, first-attack CIS MS, RR-MS) can be distinguished from one another considering the CSF proteome.

In order to quantitatively compare all CSF samples available from the three patient groups (CIS: n = 9; RR: n = 12; and control: n = 6) and determine whether the CSF proteins could distinguish between groups, we performed direct LC- mass spectrometry analysis of all the individually immunodepleted samples of the three groups (first-attack, established RR, and controls) included in this study, and quantified peptide and protein abundances employing the accurate mass and time (AMT) tag label-free quantification approach. The term *direct* preceding LC is used to emphasize that we did it without data-dependent MS/MS. The advantage of immunoaffinity depletion to remove obscuring high abundance proteins is apparent, because without depletion we previously identified 284 proteins; following depletion we identified an average of 476 proteins in direct LC- mass spectrometry analyses of the individual CSF samples in the three groups. **Figure 2B** is the partial least squares analysis for the results from the label-free quantification of all the individual samples; displaying good separation of the three groups applying the CSF proteome quantification results. Analysis of the quantitative differences in protein abundance comparing control, first-attack and established RR-MS samples revealed group specific differences in protein abundance. We performed a statistical test of variance of differences (ANOVA) across all data sets based on clinical diagnoses (e.g., Control, first-attack, established RR-MS), followed by unsupervised hierarchical clustering analysis of the statistically significant proteins (p-value <0.05) (see **Figure 2A**).

We then selected the CNS-specific proteins detected in CSF which showed significant quantitative differences in the first-attack CIS MS group compared to established RR-MS and controls (**Table 1** **,** **Table 2** **,** **Table 3**). There were a total of 20 such proteins. Nine were significantly increased in first-attack CIS MS compared to both groups. The most striking increase was in soluble Nogo receptor. Five proteins were significantly decreased in first-attack CIS MS compared to both other groups. Another six proteins were significantly increased in the first-attack CIS MS group compared to established RR-MS, but significantly decreased compared to levels in control CSF. At least 15 of these 20 proteins (75%) affect synapse, axon, and neuron functioning (gray matter associated), as opposed to myelin (white matter). Myelin proteins were detected in both established RR-MS and first-attack MS including myelin oligodendrocyte glycoprotein, myelin-associated glycoprotein, and proteolipid protein. They did not exhibit quantifiable differences in abundance. Neuronal related proteins, such as amyloid precursor protein, and neuronal adhesion molecules, such as NCAM, were also found among the 20 proteins. In another set of first-attack MS CSF samples that were previously profiled by offline 2D-LC-MS/MS (without applying immunodepletion due to sample size limitations), all the above 20 CNS-specific proteins were detected at significantly higher concentrations than the myelin proteins.

**Table 1:** Significant CSF Brain Protein Changes in first-attack CIS-MS vs. established RR-MS and Controls Increased in first attack CIS-MS vs. established RR-MS, Controls.

	Fold change			
Protein name	First-attack CIS vs. RR	First-attack CIS vs. Control	Established RR vs. Control	Function
Nogo receptor	8.04	2.62	−3.07	Regulates axonal growth, regeneration, synaptic recovery; decreases amyloid beta levels
Kallikrein-6 (Neurosin)	2.79	1.06	−2.64	Serine protease, produced by activated macrophages, active against extracellular matrix, amyloid precursor protein, myelin basic protein, alpha synuclein.
Cerebellin-1	2.67	1.46	−1.83	Synapse integrity, plasticity, stimulates norepinephrine release
Ceruloplasmin	1.78	1.70	−1.05	Iron transport, binds copper
Dickkopf-3 (RIG-like 7–1)	1.78	1.12	−1.59	Affects synapse formation, signaling
Amyloid beta precursor- like protein 1	1.68	0.97	−1.73	Involved in synapse maturation, postsynaptic function, neurite outgrowth.
Activated leukocyte cell adhesion molecule (CD166)	1.45	1.16	−1.24	Neurite extension, controls MMP-2 activation,expressed on neurons, activated T and B cells, monocytes.
Neural cell adhesion molecule 2	2.34	1.05	−2.24	Type 1 membrane glycoprotein, implicated in interneuronal and glia-neuronal adhesion, reparative and remyelinating activity.
Neural epidermal growth factor like 2/cerebral protein-12	2.12	1.54	−1.38	Secreted glycoprotein involved in neural cell growth and differentiation.

**Table 2:** Decreased in first-attack CIS-MS vs established RR-MS and controls.

	Fold change			
Protein name	First-attack CIS vs. RR	First-attack CIS vs. Control	Established RR vs. Control	Function
Clusterin (Apolipoprotein J, complement lysis inhibitor)	−1.07	−1.65	−1.54	Secreted chaperone, involved in protein folding/aggregation, clearance of misfolded proteins, protects against apoptosis and complement cytolysis.
Brevican	−1.14	−2.25	−1.98	Brain specific proteoglycan involved in cortical CNS development.
Neuronal cadherin	−1.60	−2.14	−1.34	Synapse adhesion, axon outgrowth and guidance, neuronal recognition, dendritic spine density, adhesion molecule.
Chitinase-3-like 1 protein	−2.81	−1.43	1.97	Secreted by activated macrophages; plays role in response to pathogens, ability of cell to respond to microenvironment.
Neogenin	−2.83	−1.94	1.46	Transmembrane receptor involved in neuronal differentiation, apoptosis, repulsive axon guidance, cell adhesion mechanisms.

**Table 3:** Increased in first-attack CIS-MS vs established RR-MS, but Decreased in first-attack CIS-MS vs Controls.

	Fold change			
Protein name	First-attack CIS vs. RR	First-attack CIS vs. Control	Established RR vs. Control	Function
Multifunctional protein MFP (collagen alpha 1 18) chain, Endostatin)	3.11	−1.45	−4.51	Extracellular matrix protein, antiangiogenic
Dystroglycan 1	2.02	−1.06	−2.15	Laminin binding component,scaffolds axin to cytoskeleton,cell adhesion receptor.
Contactin 2	1.70	−1.17	−1.99	Neuronal membrane protein that functions as adhesion molecule,involved in axonal connections, expressed on axons and juxtaparanodal region of myelinating oligodendrocytes.
Ephrin type A receptor 4	1.40	−1.05	−1.46	Member of protein-tyrosine kinase family, involved in signal transduction,axon and dendritic development.
Neural cell adhesion molecule L1 like protein	1.30	−1.17	−1.52	Neural recognition molecule involved insignal transduction, synaptic plasticity, neuriote outgrowth, suppresses neuronal death.
Contactin 1	1.14	−1.45	−1.65	Neuronal membrane protein, axon-myelinating glial cell signaling, oligodendrocyte generation via NOTCH 1 ligand.

## Discussion

The proteomic data in our study is consistent with high resolution imaging studies suggesting gray matter is involved in the early stages of MS. Interesting observations from our data are that the CSF proteome appears to distinguish first-attack MS from RR-MS and controls and that the first-attack MS CSF proteome is distinguished from RR-MS and control proteomes by gray matter component changes, not myelin component changes.

First-attack CIS-MS patients showed distinct CSF proteomes from those of established RR-MS and controls. The difference in proteins is not explainable by changes associated with having an attack, in and of itself, since the majority of the RR-MS cohort had their CSF obtained following an attack. Rather, these differences suggest a unique association to the first attack. It has been thought previously that MS relapses represent injury to eloquent CNS [11], and the consequence of random formation of new macroscopic lesions (referred to pathologically as plaques, and visible on neuroimaging). Documentation of signature CSF proteins suggests that the first attack in MS may not be a random occurrence, but rather orchestrated by specific circumstances that culminate in clinical disease expression.

Careful CSF analysis should shed more light on the etiological factors associated with initiation of clinically apparent RR-MS. The first-attack MS patients showed identical patterns of increased and decreased quantities of proteins, different from established RR-MS patients (**Table 1** **,** **Table 2** **,** **Table 3**).

The number of proteins referable to synapse, axon, and neuronal function that distinguish the first-attack MS group is striking. Nogo receptor, out of proportion to any other known protein, was markedly elevated in the CSF of first-attack patients compared to both RR-MS and controls. Nogo receptors regulate dendritic spine morphology. High expression of receptor has been associated with poorer synapse functioning [12]. Soluble Nogo receptor enhances axonal regeneration, and rescues retinal ganglion cells and synapses from injury in a chronic glaucoma model [13], [14]. In a mouse model of chronic spinal cord injury, intrathecal injection of Nogo receptor enhanced axonal density and functional recovery [15]. Soluble Nogo, but not receptor, has been previously reported in the CSF of MS patients [16].

First-attack MS patients also showed a significant increase in their CSF of an axonal glycoprotein, contactin-2/TAG-1. This is a protein which earlier was reported as an autoimmune target in MS, with elevated levels of antibodies as well as T-cell responses in MS vs non-MS patients [17], [18]. There is an increasing literature on the importance of gray matter, neuronal and axonal involvement in MS, even at very early time-points [17]. Our findings support this, and indicate that axonal, neuronal and synaptic involvement may be required for the initial presentation of MS. It is interesting in this disease, which is characterized by demyelination as it progresses, that gray matter components may be diagnostically more useful than myelin components at the earliest stages.

Only four prior studies have performed proteomic analysis of CIS CSF samples. None of those studies used techniques that approached the sensitivity of the current analysis. Tumani et al [19] evaluated CSF from a total of 16 CIS patients, half of whom developed disease activity in the next two years to qualify for a diagnosis of relapsing MS. They noted a total of 2,193 2-D DIGE gel spots; nine showed quantitative differences between the two groups. In our analysis none of these nine proteins were uniquely associated with first-attack MS (CIS) versus established RR-MS.

Comabella et al. [20] screened pooled CSF from 30 CIS patients who were oligoclonal band negative in CSF, with normal brain MRI maintained over one to five years (non-MS CIS); and 30 CIS patients with oligoclonal band positivity, abnormal brain MRI, and conversion to clinically definite MS over the next five years. In their paper the CIS group is much more likely not to have MS. They identified a total of 267 proteins. The CIS-MS group showed differential expression of 23 proteins, with 17 upregulated and 6 downregulated. They then chose the three most consistently represented for validation (ceruloplasmin, vitamin D binding protein, chitinase-3-like protein 1). Only chitinase-3-like protein 1 could be “validated” by ELISA in additional patients. We did confirm elevated ceruloplasmin in our CIS cohort, but actually found chitinase-3-like protein to be significantly decreased in CIS. Kroksveen et al [21] also reported similar data to ours with respect to myelin related proteins not detected, or not differentiating, first-attack MS from established RR-MS.

Dhaunchak et al. evaluated 8 CIS pediatric patients who turned out to have RR-MS, and identified 67 proteins that differed from the non RR-MS group [22]. Of the top 16 such proteins, 20% dealt with the axoglial apparatus. They concluded that perturbed axoglial interactions must be involved in the early pathogenesis of MS. This study is not directly comparable, since it focused on pediatric patients, and did not compare first attack to established MS. Nevertheless it also linked gray matter rather than myelin components to CIS.

Our application of immunoaffinity depletion and the AMT tag strategy combining both offline 2D-LC-*MS/MS* (on pooled multiple sclerosis sample) and direct LC-*MS* analyses (on individual samples from the three groups) [23] on high-sensitivity nanoLC coupled to high-resolution mass spectrometers led to both broad proteome coverage and reliable protein identification and quantitation. It maximized the findings possible from size-limited CSF sample sets, contributing to truly comprehensive characterization of the CSF proteome in MS. The same strategy has recently led to the identification of disease-specific CSF proteins which differentiated *CFS* from *nPTLS*, as well as from healthy control CSF [9], demonstrating its effectiveness in proteomic investigations in the biofluids.

Although the use of our mass spectrometry based proteomics method was for research purposes, they may have added value to current magnetic resonance imaging (MRI) because conventional MRI generally does not detect gray matter lesions. That requires non conventional advanced imaging technologies [24].

In conclusion, although this is an early investigation, it has raised intriguing findings which suggest that the CIS/true first-attack presentation of MS may not be random. The data also indicate that the CSF proteome of these patients is distinguishable from established RR-MS, particularly by gray matter components (axon, neuron, synapse), and that gray matter rather than myelin is more proximally involved in the initiation of MS.

## Methods

### Ethics Statement

Approval for the conduct of this study was obtained from the Institutional Review Board of New Jersey Medical School, the Institutional Review Board of Pacific Northwest National Laboratory, the Human Ethics Committee at the Faculty of Medicine of Uppsala University, and the Human Investigation Review Board of the University of Szeged (in agreement with the Declaration of Helsinki). Written consent was obtained from subjects.

### Introduction: Proteome Analysis of Cerebrospinal Fluid

Proteomics analysis of CSF samples faces two major analytical challenges: extremely high dynamic range in protein concentration (e.g., the top-14 most abundant proteins consist of ∼95% of protein mass in CSF) and low overall protein concentration (i.e., typically 0.3 mg/mL, comparing to 60 mg/mL in blood plasma, under normal conditions). To maximize the findings possible from size-limited CSF sample sets, immunoaffinity depletion and the AMT tag strategy that combines both offline 2D-LC-*MS/MS* and direct LC-*MS* analyses were employed to provide both broad proteome coverage and reliable protein identification and quantitation. The offline 2D-LC-*MS/MS* analysis, where immunoaffinity-depleted CSF samples were fractionated into 30 fractions and each fraction was analyzed by highly sensitive LC-*MS/MS* on high-resolution Orbitrap Velos mass spectrometer, offers the broadest CSF proteome coverage. However, the use of immunoaffinity depletion and offline fractionation (30 fractions in this study) requires a large amount of starting material; hence it is best suited for deep profiling of the pooled MS sample for qualitative comparisons with the deep proteomes that were previously established for two ONDs and healthy controls. The direct LC- mass spectrometry analysis of individual CSF samples for label-free quantitation provides both high throughput measurements and good quantitation of relative protein abundance, and therefore uniquely suited for analysis of the entire set of individual CSF sample in the CIS, RR and control groups. Therefore, combining both the offline 2D-LC-*MS/MS* and direct LC- mass spectrometry analyses maximized the findings possible from size-limited CSF sample sets, contributing to truly comprehensive characterization of the CSF proteome in MS.

### Subjects and Samples

We collected CSF from three subject groups with IRB approvals. Group 1 involved 9 first-attack CIS patients who eventually met the criteria for MS [10]. There were 8 females and 1 male, ranging in age from 18 to 42 years. Three had optic neuritis and 6 a multifocal CNS syndrome. Patients underwent lumbar puncture within 8 weeks of symptom onset. All had abnormal conventional brain MRI suggestive for MS, and were shown to be CSF oligoclonal band positive. Group 2 involved 12 patients with established diagnosis of RR-MS by the 2005 McDonald criteria [10]. There were 9 females and 3 males, ranging in age from 19 to 47 years. Disease duration ranged from 3 months to 9 years. Seven underwent lumbar puncture within 8 weeks of a clinical relapse. All had abnormal brain MRI, and were shown to be CSF oligoclonal band positive. Group 3 involved 6 control subjects without overt organic CNS disease who underwent lumbar puncture for headache (n = 5) or tinnitus (n = 1). There were 4 females and 2 males, ranging in age from 31 to 54 years. In addition, for comparative purposes, we used previously published protein lists generated from 2 OND groups (CFS and neurologic PTLS,) [9], and more than 200 healthy and non-neurologic controls [8], [9].

We analyzed a separate group of 10 patients with CIS- first-attack MS. Because of limited volume, it did not have the advantage of the current methods of immunoaffinity depletion of abundant proteins (which can mask less abundant proteins) and high fractionation of the sample. Nevertheless, this independent group permitted us to evaluation whether the gray matter proteins described in the Results in the immunoaffinity-depleted patients was also found in this group.

All CSF samples were immediately processed (cells spun out, and CSF aliquoted) and frozen at −80°C. RBC counts were less than 10 per mm^3^.

### Immunodepletion of Abundant Proteins from CSF

All CSF samples in the primary groups had the 14 most abundant proteins removed employing immunodepletion as previously described [8] increasing the depth of proteome coverage. Briefly, prior to immunodepletion CSF samples were concentrated as follows: a 2.0 mL CSF aliquot was concentrated with a Millipore Amicon Ultra-4 3000 MWCO filter (Fisher Scientific, Pittsburgh, PA) to a final volume of 100 μl. The concentrated samples were then depleted of the 14 most abundant proteins using an IgY14 LC5 depletion column from Sigma (St. Louis, MO), and the depleted and bound proteins were collected. The depleted CSF fractions were then concentrated using a Millipore Amicon Ultra-15 3000 MWCO filter, (Fisher Scientific, Pittsburgh, PA), to a final volume of ∼200 μl. The bound fractions samples underwent a buffer exchange into 50 mM NH_4_HCO_3_ (Sigma, St. Louis, MO). The volume of the samples was then adjusted using 50 mM NH_4_HCO_3_ to ensure that all samples had the same volume for in-solution digestion.

Both the flow-through (lower abundance proteins) and bound fractions from the group pooled CSF samples were collected and processed identically by high-resolution two-dimensional liquid chromatography coupled to high performance tandem mass spectrometry (2D-LC-MS/MS) analysis. These analyses produced the in-depth characterization of the CSF proteome, and the combined results of abundant protein and less abundant protein fractions were used in the creation of an AMT tag database [25] for high throughput analysis of a larger number of individual subject samples using LC- mass spectrometry.

### Protein Digestion

Proteins isolated from CSF were digested with trypsin and processed as previously described [8]. Briefly, Solid urea (Sigma, St. Louis, MO) was added to each sample to a final concentration of 8 M. The samples were incubated at 37°C for 1 hour to denature the proteins. Following denaturation, the disulfide bonds were reduced using 10 mM DTT from Sigma (St. Louis, MO) for 1 hour at 37°C. Then the samples were alkylated with 40 mM iodoacetamide from (Sigma, St. Louis, MO) for 1 hour in the dark at 37°C. The samples were diluted 10× with 50 mM NH_4_HCO_3_. Following dilution, 1 mM CaCl_2_ from Sigma (St. Louis, MO) was added. Sequencing grade modified trypsin from Promega (Madison, WI) was then added in a 1∶50 trypsin-to-protein ration. The samples were incubated for 3 hour at 37°C. Following trypsin digestion and SPE clean-up utilizing C-18 SPE cartridges from Supelco (St. Louis, MO) samples were concentrated in a Speed-Vac and the final peptide concentration (BCA assay (Pierce, Rockford, IL) was determined. Lastly all tryptic digests were snap frozen in liquid nitrogen and stored at −80°C until further processing and analysis.

### High-pH Reversed-Phase LC Fractionation and LC-MS/MS Analysis

A total of 300 μg of tryptic peptides from both the IgY14 bound and flow-through fractions from the pooled *MS* CSF samples were fractionated by High pH reversed phase (HPRP) LC as previously described [26]. 30 HPRP fractions were collected and 20% of each fraction was injected for reversed-phase LC-*MS*/*MS* analysis. HPRP fractions of the IgY14 bound fraction samples were analyzed on an LTQ (ThermoFisher, San Jose, CA) linear ion trap, and HPRP fractions of the IgY14 flow-through fraction samples were analyzed on an LTQ-Orbitrap Velos (ThermoFisher) instrument, operated in data-dependent mode and same LC conditions as previously described [8]. Briefly, a custom HPLC system was configured using 65 mL Isco Model 65D syringe pumps (Isco, Lincoln, NE), 2-position Valco valves (Valco Instruments, Houston, TX), and a PAL autosampler (Leap Technologies, Carrboro, NC) to allow for fully automated sample analysis across four separate HPLC columns. RP capillary HPLC columns were manufactured in-house by slurry packing 3 mm Jupiter C18 particles (Phenomenex, Torrance, CA) into a 70 cm×75 mm i.d. fused silica capillary tubing (Polymicro Technologies, Phoenix, AZ). An exponential HPLC gradient of 100 min (from 0−70% B) was used for each analysis, with mobile phases consisting of 0.1% formic acid in water (A) and 0.1% formic acid in ACN (B). Electrospray emitters were custom made using 150 um o.d. x 20 um i.d. chemically etched fused silica [27]. The heated capillary temperature and spray voltage were 275°C and 2.2 kV, respectively. Data was acquired for 100 min, beginning 65 min after sample injection and 15 min into gradient. Orbitrap spectra (AGC 1×10^6^) were collected from 400–2000 m/z at a resolution of 30,000 while data dependent ion trap CID *MS*/*MS* (collision energy 35%, AGC 3×10^4^) spectra were acquired for the ten most abundant ions. A dynamic exclusion time of 180 sec was used to discriminate against previously analyzed ions.

### Direct Reversed-Phase Capillary LC- mass spectrometry Analysis

Label-free quantification of proteins in individual CSF samples was performed as previously described [8]. To analyze the unfractionated, individually immunodepleted CSF samples, the RPLC and LTQ-Orbitrap Velos mass spectrometer were operated under the similar conditions as described above except that the data dependent mode was set up so that full scan mass spectrometry spectra (m/z 400–2000) were acquired in the Orbitrap with resolution of 60,000 at m/z 400 (AGC 1×10^6^) while *MS/MS* spectra were acquired for the six most abundant ions (however *MS*/*MS* data acquired here were not used for the quantitative analysis).

### Data Analysis

The LTQ raw data from the pooled samples was extracted using Extract_*MS*n (version 3.0; ThermoFisher) and analyzed with the SEQUEST algorithm (V27 revision 12; ThermoFisher) searching the *MS*/*MS* data against the human IPI database (Version 3.40). Precursor mass tolerance of 3 daltons and 1 dalton for *MS*/*MS* ion masses without an enzyme defined, as well as static carboxyamidomethylation of cysteine and dynamic oxidation of methionine were used for the database search. The LTQ-Orbitrap Velos *MS*/*MS* data were first processed by in-house software Decon*MS*n [28] accurately determining the monoisotopic mass and charge state of parent ions, followed by SEQUEST search against the IPI database in the same fashion as described above, with the exception that a 0.1-dalton mass tolerance for parent ion masses and 1 dalton mass tolerance for fragment ion masses were used. Data filtering criteria based on the mass spectrometry -GF score and precursor ion mass accuracy (+/−10 ppm) and cut offs were developed using the decoy database approach and applied for filtering the raw data to limit false positive identifications to <1% at the peptide level [29]–[31].

The AMT tag strategy [25] was used for label-free quantification of mass spectrometry features observed in the LTQ-Orbitrap Velos analysis of the individual CSF samples from control and MS samples. The filtered *MS*/*MS* peptide identifications obtained from the 2D-LC-*MS*/*MS* analyses of all pooled CSF samples were included in an AMT tag database with their theoretical mass and normalized elution time (NET; from 0 to 1) recorded. LC- mass spectrometry datasets were then analyzed by in-house software VIPER [32] that detects features in mass–NET space and assigned them to peptides in the AMT tag database [33]. False discovery rate was controlled by filtering results for an FDR <3% filtering by STAC score [34] and mass measurement accuracy within 10 ppm.

The resulting lists of peptides from 2D-LC-*MS*/*MS* or direct LC- mass spectrometry analysis were further processed by ProteinProphet software [35] to remove redundancy in protein identification.

Data normalization and quantification of the changes in protein abundance between the normal CIS-MS and RR-MS CSF samples were performed and visualized using in-house software DAnTE [36]. Briefly, peptide intensities from the LC- mass spectrometry analyses of the individual samples were log2 transformed and normalized using a mean central tendency procedure. Peptide abundances from the individual samples were then “rolled up” to the protein level employing the R-rollup method (based on trends at peptide level) implemented in DAnTE. ANOVA, partial least squares (PLS) and clustering analyses were also performed using DAnTE.

Pathway Analysis of the data was performed with Ingenuity Pathways Analysis (Ingenuity Systems, www.ingenuity.com Accessed 2013), as we have done before [9]. Canonical pathway analysis identified the pathways from the Ingenuity Pathways Analysis library of canonical pathways that were most significant to the MS proteins identified. The significance of the associations were assessed with the Fisher's exact test.

## Supporting Information

Table S1 **Proteins Identified or Not in MS Patients Compared to Normals and Other Neurologic Disease (OND).**
(PDF)

Table S2 **Proteins with significant difference in abundance by ANOVA (p-value <0.05).**
(PDF)
