# Supplementary material for: Gray Matter Is Targeted in First-Attack Multiple Sclerosis
Source: PLoS One. 2013 Sep 10;8(9):e66117. doi: 10.1371/journal.pone.0066117 (PMC3769274; doi:10.1371/journal.pone.0066117)
Supplement: Table S1 — Proteins Identified or Not in MS Patients Compared to Normals and Other Neurologic Disease (OND). (PDF) [file pone.0066117.s001.pdf]

Table S1. Proteins Identified or Not in MS Patients Compared to Normals and Other Neurologic Disease (OND)					
**IPI**	**Gene**	**Protein Name**	**MS**	**Normal**	**OND**
IPI00000027	ADCYAP1	PITUITARY ADENYLATE CYCLASE-ACTIVATING POLYPEPTIDE PRECURSOR.	X	X	X
IPI00000044	PDGFB	PLATELET-DERIVED GROWTH FACTOR B CHAIN PRECURSOR.	X	X	X
IPI00000070	LDLR	LOW-DENSITY LIPOPROTEIN RECEPTOR PRECURSOR.	X	X	X
IPI00000130	SST	SOMATOSTATIN PRECURSOR.	X	X	X
IPI00000137	GNPTG	N-ACETYLGLUCOSAMINE-1-PHOSPHOTRANSFERASE SUBUNIT GAMMA PRECURSOR.	X	X	X
IPI00000138	MGAT1	ALPHA-1,3-MANNOSYL-GLYCOPROTEIN 2-BETA-N-ACETYLGLUCOSAMINYLTRANSFERASE.	X	X	X
IPI00000144	OXT	OXYTOCIN-NEUROPHYSIN 1 PRECURSOR.	X	X	X
IPI00000160	POMC	PROOPIOMELANOCORTIN PREPROPROTEIN.	X	X	X
IPI00000190	CD81	CD81 ANTIGEN.	X	X	X
IPI00000192	SON	ISOFORM F OF SON PROTEIN.	X	X	X
IPI00000230	TPM1	TROPOMYOSIN 1 ALPHA CHAIN ISOFORM 2.	X	X	X
IPI00000459	PRRG1	TRANSMEMBRANE GAMMA-CARBOXYGLUTAMIC ACID PROTEIN 1 PRECURSOR.	X	X	X
IPI00000775	LRIG1	ISOFORM 1 OF LEUCINE-RICH REPEATS AND IMMUNOGLOBULIN-LIKE DOMAINS PROTEIN 1 PRECURSOR.	X	X	X
IPI00000779	ADAM22	ISOFORM 1 OF ADAM 22 PRECURSOR.	X	X	X
IPI00000816	YWHAE	14-3-3 PROTEIN EPSILON.	X	X	X
IPI00000824	NTRK3	ISOFORM A OF NT-3 GROWTH FACTOR RECEPTOR PRECURSOR.	X	X	X
IPI00000828	PENK	PROENKEPHALIN A PRECURSOR.	X	X	X
IPI00000832	PDYN	BETA-NEOENDORPHIN-DYNORPHIN PRECURSOR.	X	X	X
IPI00000846	CHD4	ISOFORM 1 OF CHROMODOMAIN-HELICASE-DNA-BINDING PROTEIN 4.	X	X	X
IPI00000874	PRDX1	PEROXIREDOXIN-1.	X	X	X
IPI00000877	HYOU1	HYPOXIA UP-REGULATED PROTEIN 1 PRECURSOR.	X	X	X
IPI00000959	VIP	ISOFORM 1 OF VIP PEPTIDES PRECURSOR.	X	X	X
IPI00001399	AJAP1	ADHERENS JUNCTION-ASSOCIATED PROTEIN 1.	X	X	X
IPI00001477	DDR1	ISOFORM 1 OF EPITHELIAL DISCOIDIN DOMAIN-CONTAINING RECEPTOR 1 PRECURSOR.	X	X	X
IPI00001506	NPY	NEUROPEPTIDE Y PRECURSOR.	X	X	X
IPI00001592	GPNMB	ISOFORM 2 OF TRANSMEMBRANE GLYCOPROTEIN NMB PRECURSOR.	X	X	X
IPI00001593	PRCP	LYSOSOMAL PRO-X CARBOXYPEPTIDASE PRECURSOR.	X	X	X
IPI00001610	IGF1	INSULIN-LIKE GROWTH FACTOR IA PRECURSOR.	X	X	X
IPI00001611	IGF2	ISOFORM 1 OF INSULIN-LIKE GROWTH FACTOR II PRECURSOR.	X	X	X
IPI00001633	FLRT2	LEUCINE-RICH REPEAT TRANSMEMBRANE PROTEIN FLRT2 PRECURSOR.	X	X	X
IPI00001662	OPCML	OPIOID-BINDING PROTEIN/CELL ADHESION MOLECULE PRECURSOR.	X	X	X

Table S1. Proteins Identified or Not in MS Patients Compared to Normals and Other Neurologic Disease (OND)					
**IPI**	**Gene**	**Protein Name**	**MS**	**Normal**	**OND**
IPI00001734	PSAT1	ISOFORM 1 OF PHOSPHOSERINE AMINOTRANSFERASE.	X	X	X
IPI00001793	RFNG	BETA-1,3-N-ACETYLGLUCOSAMINYLTRANSFERASE RADICAL FRINGE.	X	X	X
IPI00001952	ENDOD1	ENDONUCLEASE DOMAIN-CONTAINING 1 PROTEIN PRECURSOR.	X	X	X
IPI00001960	CLIC4	CHLORIDE INTRACELLULAR CHANNEL PROTEIN 4.	X	X	X
IPI00002142	PCDH10	PROTOCADHERIN-10 PRECURSOR.	X	X	X
IPI00002147	CHI3L1	CHITINASE-3-LIKE PROTEIN 1 PRECURSOR.	X	X	X
IPI00002211	SEMA6A	ISOFORM 2 OF SEMAPHORIN-6A PRECURSOR.	X	X	X
IPI00002236	MFGE8	LACTADHERIN PRECURSOR.	X	X	X
IPI00002280	PCSK1N	PROSAAS PRECURSOR.	X	X	X
IPI00002307	NLGN3	ISOFORM 1 OF NEUROLIGIN-3 PRECURSOR.	X	X	X
IPI00002320	FLRT3	LEUCINE-RICH REPEAT TRANSMEMBRANE PROTEIN FLRT3 PRECURSOR.	X	X	X
IPI00002334	D4S234E	NEURON-SPECIFIC PROTEIN FAMILY MEMBER 1.	X	X	X
IPI00002406	BCAM	LUTHERAN BLOOD GROUP GLYCOPROTEIN PRECURSOR.	X	X	X
IPI00002525	NENF	NEUDESIN PRECURSOR.	X	X	X
IPI00002714	DKK3	DICKKOPF-RELATED PROTEIN 3 PRECURSOR.	X	X	X
IPI00002732	EXTL2	EXTL2 PROTEIN (FRAGMENT).	X	X	X
IPI00002745	CTSZ	CATHEPSIN Z PRECURSOR.	X	X	X
IPI00002790	SEL1L	ISOFORM 1 OF PROTEIN SEL-1 HOMOLOG 1 PRECURSOR.	X	X	X
IPI00002816	CTSF	CATHEPSIN F PRECURSOR.	X	X	X
IPI00002818	KLK11	ISOFORM 1 OF KALLIKREIN-11 PRECURSOR.	X	X	X
IPI00002925	CARTPT	COCAINE- AND AMPHETAMINE-REGULATED TRANSCRIPT PROTEIN PRECURSOR.	X	X	X
IPI00003021	ATP1A2	SODIUM/POTASSIUM-TRANSPORTING ATPASE SUBUNIT ALPHA-2 PRECURSOR.	X	X	X
IPI00003102	CNTFR	CILIARY NEUROTROPHIC FACTOR RECEPTOR ALPHA PRECURSOR.	X	X	X
IPI00003111	LOC652694	IG KAPPA CHAIN V-I REGION AU.	X	X	X
IPI00003176	HTRA1	SERINE PROTEASE HTRA1 PRECURSOR.	X	X	X
IPI00003269	DKFZp686D0972	HYPOTHETICAL PROTEIN LOC345651.	X	X	X
IPI00003351	ECM1	EXTRACELLULAR MATRIX PROTEIN 1 PRECURSOR.	X	X	X
IPI00003362	HSPA5	HSPA5 PROTEIN.	X	X	X
IPI00003366	NTRK2	ISOFORM TRKB OF BDNF/NT-3 GROWTH FACTORS RECEPTOR PRECURSOR.	X	X	X
IPI00003384	CELSR1	ISOFORM 1 OF CADHERIN EGF LAG SEVEN-PASS G-TYPE RECEPTOR 1 PRECURSOR.	X	X	X
IPI00003590	QSOX1	ISOFORM 1 OF SULFHYDRYL OXIDASE 1 PRECURSOR.	X	X	X
IPI00003802	MAN2A1	ALPHA-MANNOSIDASE 2.	X	X	X
IPI00003807	ACP2	LYSOSOMAL ACID PHOSPHATASE PRECURSOR.	X	X	X

Table S1. Proteins Identified or Not in MS Patients Compared to Normals and Other Neurologic Disease (OND)					
**IPI**	**Gene**	**Protein Name**	**MS**	**Normal**	**OND**
IPI00003865	HSPA8	ISOFORM 1 OF HEAT SHOCK COGNATE 71 KDA PROTEIN.	X	X	X
IPI00003907	PCDHGA12; PCDHGC5	ISOFORM 1 OF PROTOCADHERIN GAMMA C5 PRECURSOR.	X	X	X
IPI00003919	QPCT	GLUTAMINYL-PEPTIDE CYCLOTRANSFERASE PRECURSOR.	X	X	X
IPI00003971	RTN1	ISOFORM RTN1-A OF RETICULON-1.	X	X	X
IPI00004114	RNASE6	RIBONUCLEASE K6 PRECURSOR.	X	X	X
IPI00004315	SIGLEC9	SIALIC ACID-BINDING IG-LIKE LECTIN 9 PRECURSOR.	X	X	X
IPI00004367	FXYD6	FXYD DOMAIN-CONTAINING ION TRANSPORT REGULATOR 6 PRECURSOR.	X	X	X
IPI00004373	MBL2	MANNOSE-BINDING PROTEIN C PRECURSOR.	X	X	X
IPI00004409	DDR2	DISCOIDIN DOMAIN-CONTAINING RECEPTOR 2 PRECURSOR.	X	X	X
IPI00004413	TNFRSF21	TUMOR NECROSIS FACTOR RECEPTOR SUPERFAMILY MEMBER 21 PRECURSOR.	X	X	X
IPI00004433	CNTN6	CONTACTIN-6 PRECURSOR.	X	X	X
IPI00004440	PTPRN	RECEPTOR-TYPE TYROSINE-PROTEIN PHOSPHATASE-LIKE N PRECURSOR.	X	X	X
IPI00004573	PIGR	POLYMERIC IMMUNOGLOBULIN RECEPTOR PRECURSOR.	X	X	X
IPI00004656	B2M	BETA-2-MICROGLOBULIN.	X	X	X
IPI00004669	GALNT2	POLYPEPTIDE N-ACETYLGALACTOSAMINYLTRANSFERASE 2.	X	X	X
IPI00004798	CRISP3	CDNA FLJ75207.	X	X	X
IPI00004946	CXCL16	CHEMOKINE (C-X-C MOTIF) LIGAND 16.	X	X	X
IPI00004962	GOLIM4	GOLGI INTEGRAL MEMBRANE PROTEIN 4.	X	X	X
IPI00005123	EFNA3	EPHRIN-A3 PRECURSOR.	X	X	X
IPI00005126	EFNB2	EPHRIN-B2 PRECURSOR.	X	X	X
IPI00005142	FGFR1	ISOFORM 1 OF BASIC FIBROBLAST GROWTH FACTOR RECEPTOR 1 PRECURSOR.	X	X	X
IPI00005222	EPHB6	EPHRIN TYPE-B RECEPTOR 6 PRECURSOR.	X	X	X
IPI00005292	SPOCK1	TESTICAN-1 PRECURSOR.	X	X	X
IPI00005465	TSPYL2	TESTIS-SPECIFIC Y-ENCODED-LIKE PROTEIN 2.	X	X	X
IPI00005474	LHPP	PHOSPHOLYSINE PHOSPHOHISTIDINE INORGANIC PYROPHOSPHATE PHOSPHATASE.	X	X	X
IPI00005516	LRRC4	LEUCINE-RICH REPEAT-CONTAINING PROTEIN 4 PRECURSOR.	X	X	X
IPI00005517	EFNA5	EPHRIN-A5 PRECURSOR.	X	X	X
IPI00005707	MRC2	MACROPHAGE MANNOSE RECEPTOR 2 PRECURSOR.	X	X	X
IPI00005732	ACVR1B	ISOFORM 1 OF ACTIVIN RECEPTOR TYPE-1B PRECURSOR.	X	X	X
IPI00005774	LRP8	ISOFORM 1 OF LOW-DENSITY LIPOPROTEIN RECEPTOR-RELATED PROTEIN 8 PRECURSOR.	X	X	X
IPI00005794	PGCP	60 KDA PROTEIN.	X	X	X
IPI00005859	KRT75	KERATIN, TYPE II CYTOSKELETAL 75.	X	X	X

Table S1. Proteins Identified or Not in MS Patients Compared to Normals and Other Neurologic Disease (OND)					
**IPI**	**Gene**	**Protein Name**	**MS**	**Normal**	**OND**
IPI00005908	ADAMTS1	ADAMTS-1 PRECURSOR.	X	X	X
IPI00006009	PLEKHB1	ISOFORM 2 OF PLECKSTRIN HOMOLOGY DOMAIN-CONTAINING FAMILY B MEMBER 1.	X	X	X
IPI00006114	SERPINF1	PIGMENT EPITHELIUM-DERIVED FACTOR PRECURSOR.	X	X	X
IPI00006128	SPOCK2	TESTICAN-2 PRECURSOR.	X	X	X
IPI00006130	KIAA0494	UNCHARACTERIZED CALCIUM-BINDING PROTEIN KIAA0494.	X	X	X
IPI00006154	CFHR2	ISOFORM LONG OF COMPLEMENT FACTOR H-RELATED PROTEIN 2 PRECURSOR.	X	X	X
IPI00006166	GPR37	PROBABLE G-PROTEIN COUPLED RECEPTOR 37 PRECURSOR.	X	X	X
IPI00006444	SLC24A2	ISOFORM 1 OF SODIUM/POTASSIUM/CALCIUM EXCHANGER 2 PRECURSOR.	X	X	X
IPI00006451	NSF	VESICLE-FUSING ATPASE.	X	X	X
IPI00006482	ATP1A1	ISOFORM LONG OF SODIUM/POTASSIUM-TRANSPORTING ATPASE SUBUNIT ALPHA-1 PRECURSOR.	X	X	X
IPI00006524	KIAA0319	UNCHARACTERIZED PROTEIN KIAA0319 PRECURSOR.	X	X	X
IPI00006601	CHGB	SECRETOGRANIN-1 PRECURSOR.	X	X	X
IPI00006608	APP	ISOFORM APP770 OF AMYLOID BETA A4 PROTEIN PRECURSOR (FRAGMENT).	X	X	X
IPI00006644	PLXNB1	ISOFORM 2 OF PLEXIN-B1 PRECURSOR.	X	X	X
IPI00006657	FAM20B	PROTEIN FAM20B PRECURSOR.	X	X	X
IPI00006662	APOD	APOLIPOPROTEIN D PRECURSOR.	X	X	X
IPI00006713	DNAJC3	ISOFORM 1 OF DNAJ HOMOLOG SUBFAMILY C MEMBER 3.	X	X	X
IPI00006803	CHST10	CARBOHYDRATE SULFOTRANSFERASE 10.	X	X	X
IPI00006967	PCDH9	PROTOCADHERIN-9 PRECURSOR.	X	X	X
IPI00006971	CD248	ISOFORM 1 OF ENDOSIALIN PRECURSOR.	X	X	X
IPI00007102	GLOD4	UNCHARACTERIZED PROTEIN C17ORF25.	X	X	X
IPI00007199	SERPINA10	PROTEIN Z-DEPENDENT PROTEASE INHIBITOR PRECURSOR.	X	X	X
IPI00007221	SERPINA5	PLASMA SERINE PROTEASE INHIBITOR PRECURSOR.	X	X	X
IPI00007236	NLGN1	ISOFORM 2 OF NEUROLIGIN-1 PRECURSOR.	X	X	X
IPI00007617	OR52A1	OLFACTORY RECEPTOR 52A1.	X	X	X
IPI00007709	ADAM28	ISOFORM 1 OF ADAM 28 PRECURSOR.	X	X	X
IPI00007778	CTBS	DI-N-ACETYLCHITOBIASE PRECURSOR.	X	X	X
IPI00007797	FABP5; FABP5L7	FATTY ACID-BINDING PROTEIN, EPIDERMAL.	X	X	X
IPI00007853	IFI30	GAMMA-INTERFERON-INDUCIBLE LYSOSOMAL THIOL REDUCTASE PRECURSOR.	X	X	X
IPI00007921	NRXN2	ISOFORM 1 OF NEUREXIN-2-ALPHA PRECURSOR.	X	X	X
IPI00008085	SLC39A10	ZINC TRANSPORTER ZIP10 PRECURSOR.	X	X	X
IPI00008087	FSTL5	FOLLISTATIN-RELATED PROTEIN 5 PRECURSOR.	X	X	X

Table S1. Proteins Identified or Not in MS Patients Compared to Normals and Other Neurologic Disease (OND)					
**IPI**	**Gene**	**Protein Name**	**MS**	**Normal**	**OND**
IPI00008107	LRFN2	LEUCINE-RICH REPEAT AND FIBRONECTIN TYPE-III DOMAIN-CONTAINING PROTEIN 2 PRECURSOR.	X	X	X
IPI00008148	GFRA1	ISOFORM 1 OF GDNF FAMILY RECEPTOR ALPHA-1 PRECURSOR.	X	X	X
IPI00008207	MAN1B1	ENDOPLASMIC RETICULUM MANNOSYL-OLIGOSACCHARIDE 1,2-ALPHA-MANNOSIDASE.	X	X	X
IPI00008269	NDRG4	ISOFORM 5 OF PROTEIN NDRG4.	X	X	X
IPI00008290	EPHA5	ISOFORM 1 OF EPHRIN TYPE-A RECEPTOR 5 PRECURSOR.	X	X	X
IPI00008303	NAGPA	ISOFORM 1 OF N-ACETYLGLUCOSAMINE-1-PHOSPHODIESTER ALPHA-N-ACETYLGLUCOSAMINIDASE PRECURSOR.	X	X	X
IPI00008315	EPHB1	ISOFORM 1 OF EPHRIN TYPE-B RECEPTOR 1 PRECURSOR.	X	X	X
IPI00008318	EPHA4	EPHRIN TYPE-A RECEPTOR 4 PRECURSOR.	X	X	X
IPI00008494	ICAM1	INTERCELLULAR ADHESION MOLECULE 1 PRECURSOR.	X	X	X
IPI00008533	MMP17	ISOFORM LONG OF MATRIX METALLOPROTEINASE-17 PRECURSOR.	X	X	X
IPI00008556	F11	ISOFORM 1 OF COAGULATION FACTOR XI PRECURSOR.	X	X	X
IPI00008586	CSPG5	ISOFORM 1 OF CHONDROITIN SULFATE PROTEOGLYCAN 5 PRECURSOR.	X	X	X
IPI00008603	ACTA2	ACTIN, AORTIC SMOOTH MUSCLE.	X	X	X
IPI00008780	STC2	STANNIOCALCIN-2 PRECURSOR.	X	X	X
IPI00008787	NAGLU	ALPHA-N-ACETYLGLUCOSAMINIDASE PRECURSOR.	X	X	X
IPI00008944	SCG5	ISOFORM 1 OF NEUROENDOCRINE PROTEIN 7B2 PRECURSOR.	X	X	X
IPI00008994	NDRG2	ISOFORM 1 OF PROTEIN NDRG2.	X	X	X
IPI00008997	WFDC1	WAP FOUR-DISULFIDE CORE DOMAIN PROTEIN 1 PRECURSOR.	X	X	X
IPI00009028	CLEC3B	TETRANECTIN PRECURSOR.	X	X	X
IPI00009030	LAMP2	ISOFORM LAMP-2A OF LYSOSOME-ASSOCIATED MEMBRANE GLYCOPROTEIN 2 PRECURSOR.	X	X	X
IPI00009054	BMP1	ISOFORM BMP1-3 OF BONE MORPHOGENETIC PROTEIN 1 PRECURSOR.	X	X	X
IPI00009123	NUCB2	NUCLEOBINDIN-2 PRECURSOR.	X	X	X
IPI00009145	MAN1A2	MANNOSYL-OLIGOSACCHARIDE 1,2-ALPHA-MANNOSIDASE IB.	X	X	X
IPI00009276	PROCR	ENDOTHELIAL PROTEIN C RECEPTOR PRECURSOR.	X	X	X
IPI00009294	CRIM1	CYSTEINE-RICH MOTOR NEURON 1 PROTEIN PRECURSOR.	X	X	X
IPI00009362	SCG2	SECRETOGRANIN-2 PRECURSOR.	X	X	X
IPI00009477	ICAM2	INTERCELLULAR ADHESION MOLECULE 2 PRECURSOR.	X	X	X
IPI00009619	CADM3	ISOFORM 2 OF CELL ADHESION MOLECULE 3 PRECURSOR.	X	X	X
IPI00009792	IGHV1OR15-1	IG HEAVY CHAIN V-I REGION V35 PRECURSOR.	X	X	X
IPI00009793	C1RL	COMPLEMENT C1R-LIKE PROTEIN.	X	X	X

Table S1. Proteins Identified or Not in MS Patients Compared to Normals and Other Neurologic Disease (OND)					
**IPI**	**Gene**	**Protein Name**	**MS**	**Normal**	**OND**
IPI00009802	VCAN	ISOFORM V0 OF VERSICAN CORE PROTEIN PRECURSOR.	X	X	X
IPI00009865	KRT10	KERATIN, TYPE I CYTOSKELETAL 10.	X	X	X
IPI00009867	KRT5	KERATIN, TYPE II CYTOSKELETAL 5.	X	X	X
IPI00009881	GNAS	NEUROENDOCRINE SECRETORY PROTEIN 55.	X	X	X
IPI00009901	NUTF2	NUCLEAR TRANSPORT FACTOR 2.	X	X	X
IPI00009904	PDIA4	PROTEIN DISULFIDE-ISOMERASE A4 PRECURSOR.	X	X	X
IPI00009920	C6	COMPLEMENT COMPONENT 6 PRECURSOR.	X	X	X
IPI00009950	LMAN2	VESICULAR INTEGRAL-MEMBRANE PROTEIN VIP36 PRECURSOR.	X	X	X
IPI00009997	B3GNT1	N-ACETYLLACTOSAMINIDE BETA-1,3-N-ACETYLGLUCOSAMINYLTRANSFERASE.	X	X	X
IPI00010148	PCP4	BRAIN-SPECIFIC POLYPEPTIDE PEP-19.	X	X	X
IPI00010154	GDI1	RAB GDP DISSOCIATION INHIBITOR ALPHA.	X	X	X
IPI00010182	DBI	ISOFORM A 1 OF ACYL-COA-BINDING PROTEIN.	X	X	X
IPI00010193	IFNAR2	ISOFORM 1 OF INTERFERON-ALPHA/BETA RECEPTOR BETA CHAIN PRECURSOR.	X	X	X
IPI00010295	CPN1	CARBOXYPEPTIDASE N CATALYTIC CHAIN PRECURSOR.	X	X	X
IPI00010348	DNASE2	DEOXYRIBONUCLEASE-2-ALPHA PRECURSOR.	X	X	X
IPI00010362	HLA-E	MAJOR HISTOCOMPATIBILITY COMPLEX, CLASS I, E PRECURSOR.	X	X	X
IPI00010381	SORCS3	VPS10 DOMAIN-CONTAINING RECEPTOR SORCS3 PRECURSOR.	X	X	X
IPI00010402	SH3BGRL3	PUTATIVE UNCHARACTERIZED PROTEIN.	X	X	X
IPI00010471	LCP1	PLASTIN-2.	X	X	X
IPI00010706	GSS	GLUTATHIONE SYNTHETASE.	X	X	X
IPI00010790	BGN	BIGLYCAN PRECURSOR.	X	X	X
IPI00010796	P4HB	PROTEIN DISULFIDE-ISOMERASE PRECURSOR.	X	X	X
IPI00010949	SIAE	ISOFORM 1 OF SIALATE O-ACETYLESTERASE PRECURSOR.	X	X	X
IPI00011140	NOV	PROTEIN NOV HOMOLOG PRECURSOR.	X	X	X
IPI00011192	PRSS27	SERINE PROTEASE 27 PRECURSOR.	X	X	X
IPI00011218	CSF1R	MACROPHAGE COLONY-STIMULATING FACTOR 1 RECEPTOR PRECURSOR.	X	X	X
IPI00011229	CTSD	CATHEPSIN D PRECURSOR.	X	X	X
IPI00011252	C8A	COMPLEMENT COMPONENT C8 ALPHA CHAIN PRECURSOR.	X	X	X
IPI00011261	C8G	COMPLEMENT COMPONENT C8 GAMMA CHAIN PRECURSOR.	X	X	X
IPI00011264	CFHR1	COMPLEMENT FACTOR H-RELATED PROTEIN 1 PRECURSOR.	X	X	X
IPI00011302	CD59	CD59 GLYCOPROTEIN PRECURSOR.	X	X	X
IPI00011518	BACE1	ISOFORM A OF BETA-SECRETASE 1 PRECURSOR.	X	X	X
IPI00011605	CBLN1	CEREBELLIN-1 PRECURSOR.	X	X	X

Table S1. Proteins Identified or Not in MS Patients Compared to Normals and Other Neurologic Disease (OND)					
IPI	Gene	Protein Name	MS	Normal	OND
IPI00011651	PTPRG	ISOFORM 1 OF RECEPTOR-TYPE TYROSINE-PROTEIN PHOSPHATASE GAMMA PRECURSOR.	X	X	X
IPI00011654	TUBB	TUBULIN BETA CHAIN.	X	X	X
IPI00011662	SPINT2	KUNITZ-TYPE PROTEASE INHIBITOR 2 PRECURSOR.	X	X	X
IPI00011730	EMILIN3	EMILIN-3 PRECURSOR.	X	X	X
IPI00011732	GFRA2	ISOFORM 1 OF GDNF FAMILY RECEPTOR ALPHA-2 PRECURSOR.	X	X	X
IPI00011899	BAMBI	BMP AND ACTIVIN MEMBRANE-BOUND INHIBITOR HOMOLOG PRECURSOR.	X	X	X
IPI00011994	ENPP5	ECTONUCLEOTIDE PYROPHOSPHATASE/PHOSPHODIESTERASE FAMILY MEMBER 5 PRECURSOR.	X	X	X
IPI00012011	CFL1	COFILIN-1.	X	X	X
IPI00012044	NRG3	ISOFORM 1 OF PRO-NEUREGULIN-3, MEMBRANE-BOUND ISOFORM PRECURSOR.	X	X	X
IPI00012058	BDNF	BRAIN-DERIVED NEUROTROPHIC FACTOR PRECURSOR.	X	X	X
IPI00012075	NPPC	C-TYPE NATRIURETIC PEPTIDE PRECURSOR.	X	X	X
IPI00012102	GNS	N-ACETYLGLUCOSAMINE-6-SULFATASE PRECURSOR.	X	X	X
IPI00012269	MMRN1	MULTIMERIN-1 PRECURSOR.	X	X	X
IPI00012303	SELENBP1	SELENIUM-BINDING PROTEIN 1.	X	X	X
IPI00012386	COCH	COCHLIN PRECURSOR.	X	X	X
IPI00012391	APC	ISOFORM LONG OF ADENOMATOUS POLYPOSIS COLI PROTEIN.	X	X	X
IPI00012440	FUCA2	PLASMA ALPHA-L-FUCOSIDASE PRECURSOR.	X	X	X
IPI00012503	PSAP	ISOFORM SAP-MU-0 OF PROACTIVATOR POLYPEPTIDE PRECURSOR.	X	X	X
IPI00012510	EMILIN2	EMILIN-2 PRECURSOR.	X	X	X
IPI00012545	TGOLN2	ISOFORM TGN51 OF TRANS-GOLGI NETWORK INTEGRAL MEMBRANE PROTEIN 2 PRECURSOR.	X	X	X
IPI00012585	HEXB	BETA-HEXOSAMINIDASE BETA CHAIN PRECURSOR.	X	X	X
IPI00012887	CTSL1	CATHEPSIN L1 PRECURSOR.	X	X	X
IPI00012989	MAN2B1	LYSOSOMAL ALPHA-MANNOSIDASE PRECURSOR.	X	X	X
IPI00013162	CD200	ISOFORM 1 OF OX-2 MEMBRANE GLYCOPROTEIN PRECURSOR.	X	X	X
IPI00013179	PTGDS	PROSTAGLANDIN-H2 D-ISOMERASE PRECURSOR.	X	X	X
IPI00013299	NBL1	NEUROBLASTOMA, SUPPRESSION OF TUMORIGENICITY 1.	X	X	X
IPI00013303	LSAMP	LIMBIC SYSTEM-ASSOCIATED MEMBRANE PROTEIN PRECURSOR.	X	X	X
IPI00013495	ABCF1	ISOFORM 2 OF ATP-BINDING CASSETTE SUB-FAMILY F MEMBER 1.	X	X	X
IPI00013569	PAPPA2	ISOFORM 1 OF PAPPALYSIN-2 PRECURSOR.	X	X	X
IPI00013682	ART3	ISOFORM 3 OF ECTO-ADP-RIBOSYLTRANSFERASE 3 PRECURSOR.	X	X	X
IPI00013698	ASAH1	ACID CERAMIDASE PRECURSOR.	X	X	X

Table S1. Proteins Identified or Not in MS Patients Compared to Normals and Other Neurologic Disease (OND)					
**IPI**	**Gene**	**Protein Name**	**MS**	**Normal**	**OND**
IPI00013701	PNOC	NOCICEPTIN PRECURSOR.	X	X	X
IPI00013897	ADAM10	ADAM 10 PRECURSOR.	X	X	X
IPI00013976	LAMB1	LAMININ SUBUNIT BETA-1 PRECURSOR.	X	X	X
IPI00013991	TPM2	ISOFORM 1 OF TROPOMYOSIN BETA CHAIN.	X	X	X
IPI00014048	RNASE1	RIBONUCLEASE PANCREATIC PRECURSOR.	X	X	X
IPI00014371	CDH18	CADHERIN-18 PRECURSOR.	X	X	X
IPI00014439	QDPR	DIHYDROPTERIDINE REDUCTASE.	X	X	X
IPI00014572	SPARC	SPARC PRECURSOR.	X	X	X
IPI00014592	CHAD	CHONDROADHERIN PRECURSOR.	X	X	X
IPI00014964	LY6H	LYMPHOCYTE ANTIGEN 6H PRECURSOR.	X	X	X
IPI00015049	RGMA	ISOFORM 2 OF REPULSIVE GUIDANCE MOLECULE A PRECURSOR.	X	X	X
IPI00015102	ALCAM	ISOFORM 1 OF CD166 ANTIGEN PRECURSOR.	X	X	X
IPI00015260	NELL2	PROTEIN KINASE C-BINDING PROTEIN NELL2 PRECURSOR.	X	X	X
IPI00015315	ECM2	EXTRACELLULAR MATRIX PROTEIN 2 PRECURSOR.	X	X	X
IPI00015346	CELSR2	CADHERIN EGF LAG SEVEN-PASS G-TYPE RECEPTOR 2 PRECURSOR.	X	X	X
IPI00015351	C1orf128	ISOFORM 1 OF UPF0424 PROTEIN C1ORF128.	X	X	X
IPI00015479	C12orf49	UPF0454 PROTEIN C12ORF49 PRECURSOR.	X	X	X
IPI00015525	MMRN2	MULTIMERIN-2 PRECURSOR.	X	X	X
IPI00015688	GPC1	GLYPICAN-1 PRECURSOR.	X	X	X
IPI00015842	RCN1	RETICULOCALBIN-1 PRECURSOR.	X	X	X
IPI00015881	CSF1	ISOFORM 1 OF MACROPHAGE COLONY-STIMULATING FACTOR 1 PRECURSOR.	X	X	X
IPI00015902	PDGFRB	BETA-TYPE PLATELET-DERIVED GROWTH FACTOR RECEPTOR PRECURSOR.	X	X	X
IPI00015911	DLD	DIHYDROLIPOYL DEHYDROGENASE, MITOCHONDRIAL PRECURSOR.	X	X	X
IPI00015964	GAP43	NEUROMODULIN.	X	X	X
IPI00016014	ITM2C	ISOFORM 1 OF INTEGRAL MEMBRANE PROTEIN 2C.	X	X	X
IPI00016150	SERPINI1	NEUROSERPIN PRECURSOR.	X	X	X
IPI00016334	MCAM	ISOFORM 1 OF CELL SURFACE GLYCOPROTEIN MUC18 PRECURSOR.	X	X	X
IPI00016371	ERBB4	ISOFORM JM-A OF RECEPTOR TYROSINE-PROTEIN KINASE ERBB-4 PRECURSOR.	X	X	X
IPI00016422	DCC	NETRIN RECEPTOR DCC PRECURSOR.	X	X	X
IPI00016467	SLITRK3	SLIT AND NTRK-LIKE PROTEIN 3 PRECURSOR.	X	X	X
IPI00016621	AP2A2	ADAPTOR-RELATED PROTEIN COMPLEX 2, ALPHA 2 SUBUNIT VARIANT (FRAGMENT).	X	X	X
IPI00016645	EPHA7	ISOFORM 1 OF EPHRIN TYPE-A RECEPTOR 7 PRECURSOR.	X	X	X
IPI00016666	MT3	METALLOTHIONEIN-3.	X	X	X
IPI00016679	SLITRK5	SLIT AND NTRK-LIKE PROTEIN 5 PRECURSOR.	X	X	X

Table S1. Proteins Identified or Not in MS Patients Compared to Normals and Other Neurologic Disease (OND)					
**IPI**	**Gene**	**Protein Name**	**MS**	**Normal**	**OND**
IPI00016862	GSR	ISOFORM MITOCHONDRIAL OF GLUTATHIONE REDUCTASE, MITOCHONDRIAL PRECURSOR.	X	X	X
IPI00016870	ZP2	ZONA PELLUCIDA SPERM-BINDING PROTEIN 2 PRECURSOR.	X	X	X
IPI00016915	IGFBP7	INSULIN-LIKE GROWTH FACTOR-BINDING PROTEIN 7 PRECURSOR.	X	X	X
IPI00017257	CTSO	CATHEPSIN O PRECURSOR.	X	X	X
IPI00017557	SFRP4	SECRETED FRIZZLED-RELATED PROTEIN 4 PRECURSOR.	X	X	X
IPI00017569	FAIM2	FAS APOPTOTIC INHIBITORY MOLECULE 2.	X	X	X
IPI00017601	CP	CERULOPLASMIN PRECURSOR.	X	X	X
IPI00017696	C1S	COMPLEMENT C1S SUBCOMPONENT PRECURSOR.	X	X	X
IPI00017704	COTL1	COACTOSIN-LIKE PROTEIN.	X	X	X
IPI00017968	ADM	ADM PRECURSOR.	X	X	X
IPI00018136	VCAM1	ISOFORM 1 OF VASCULAR CELL ADHESION PROTEIN 1 PRECURSOR.	X	X	X
IPI00018146	YWHAQ	14-3-3 PROTEIN THETA.	X	X	X
IPI00018206	GOT2	ASPARTATE AMINOTRANSFERASE, MITOCHONDRIAL PRECURSOR.	X	X	X
IPI00018219	TGFBI	TRANSFORMING GROWTH FACTOR-BETA-INDUCED PROTEIN IG-H3 PRECURSOR.	X	X	X
IPI00018236	GM2A	GANGLIOSIDE GM2 ACTIVATOR PRECURSOR.	X	X	X
IPI00018274	EGFR	ISOFORM 1 OF EPIDERMAL GROWTH FACTOR RECEPTOR PRECURSOR.	X	X	X
IPI00018276	SEZ6L2	SEIZURE RELATED 6 HOMOLOG (MOUSE)-LIKE 2 ISOFORM 1.	X	X	X
IPI00018305	IGFBP3	INSULIN-LIKE GROWTH FACTOR-BINDING PROTEIN 3 PRECURSOR.	X	X	X
IPI00018396	CBLN4	CEREBELLIN-4 PRECURSOR.	X	X	X
IPI00018909	TFF3	TREFOIL FACTOR 3 PRECURSOR.	X	X	X
IPI00018941	CALCB	CALCITONIN GENE-RELATED PEPTIDE 2 PRECURSOR.	X	X	X
IPI00019038	LYZ	LYSOZYME C PRECURSOR.	X	X	X
IPI00019146	CXADR	ISOFORM 1 OF COXSACKIEVIRUS AND ADENOVIRUS RECEPTOR PRECURSOR.	X	X	X
IPI00019157	CSPG4	CHONDROITIN SULFATE PROTEOGLYCAN 4 PRECURSOR.	X	X	X
IPI00019176	RARRES2	RETINOIC ACID RECEPTOR RESPONDER PROTEIN 2 PRECURSOR.	X	X	X
IPI00019209	SEMA3C	SEMAPHORIN-3C PRECURSOR.	X	X	X
IPI00019359	KRT9	KERATIN, TYPE I CYTOSKELETAL 9.	X	X	X
IPI00019372	SRGN	SERGLYCIN PRECURSOR.	X	X	X
IPI00019399	SAA4	SERUM AMYLOID A-4 PROTEIN PRECURSOR.	X	X	X
IPI00019501	EFNB3	EPHRIN-B3 PRECURSOR.	X	X	X
IPI00019533	CHI3L2	CHITINASE-3-LIKE PROTEIN 2 PRECURSOR.	X	X	X
IPI00019568	F2	PROTHROMBIN PRECURSOR (FRAGMENT).	X	X	X
IPI00019576	F10	COAGULATION FACTOR X PRECURSOR.	X	X	X

Table S1. Proteins Identified or Not in MS Patients Compared to Normals and Other Neurologic Disease (OND)					
**IPI**	**Gene**	**Protein Name**	**MS**	**Normal**	**OND**
IPI00019580	PLG	PLASMINOGEN PRECURSOR.	X	X	X
IPI00019581	F12	COAGULATION FACTOR XII PRECURSOR.	X	X	X
IPI00019591	CFB	ISOFORM 1 OF COMPLEMENT FACTOR B PRECURSOR (FRAGMENT).	X	X	X
IPI00019755	GSTO1	GLUTATHIONE TRANSFERASE OMEGA-1.	X	X	X
IPI00019771	CX3CL1	FRACTALKINE PRECURSOR.	X	X	X
IPI00019906	BSG	ISOFORM 2 OF BASIGIN PRECURSOR.	X	X	X
IPI00019943	AFM	AFAMIN PRECURSOR.	X	X	X
IPI00019954	CST6	CYSTATIN-M PRECURSOR.	X	X	X
IPI00020008	NEDD8	NEDD8 PRECURSOR.	X	X	X
IPI00020012	APLP1	AMYLOID-LIKE PROTEIN 1 PRECURSOR.	X	X	X
IPI00020019	ADIPOQ	ADIPONECTIN PRECURSOR.	X	X	X
IPI00020091	ORM2	ALPHA-1-ACID GLYCOPROTEIN 2 PRECURSOR.	X	X	X
IPI00020131	SOS1	SON OF SEVENLESS HOMOLOG 1.	X	X	X
IPI00020396	PCSK6	ISOFORM PACE4A-I OF PROPROTEIN CONVERTASE SUBTILISIN/KEXIN TYPE 6 PRECURSOR.	X	X	X
IPI00020431	TGFBR2	ISOFORM 1 OF TGF-BETA RECEPTOR TYPE-2 PRECURSOR.	X	X	X
IPI00020501	MYH11	MYOSIN-11.	X	X	X
IPI00020557	LRP1	PROLOW-DENSITY LIPOPROTEIN RECEPTOR-RELATED PROTEIN 1 PRECURSOR.	X	X	X
IPI00020599	CALR	CALRETICULIN PRECURSOR.	X	X	X
IPI00020747	SCN3B	SODIUM CHANNEL SUBUNIT BETA-3 PRECURSOR.	X	X	X
IPI00020977	CTGF	ISOFORM 1 OF CONNECTIVE TISSUE GROWTH FACTOR PRECURSOR.	X	X	X
IPI00020984	CANX	CALNEXIN PRECURSOR.	X	X	X
IPI00020986	LUM	LUMICAN PRECURSOR.	X	X	X
IPI00020987	PRELP	PROLARGIN PRECURSOR.	X	X	X
IPI00020990	OMD	OSTEOMODULIN PRECURSOR.	X	X	X
IPI00020996	IGFALS	INSULIN-LIKE GROWTH FACTOR-BINDING PROTEIN COMPLEX ACID LABILE CHAIN PRECURSOR.	X	X	X
IPI00021000	SPP1	ISOFORM A OF OSTEOPONTIN PRECURSOR.	X	X	X
IPI00021033	COL3A1	ISOFORM 1 OF COLLAGEN ALPHA-1(III) CHAIN PRECURSOR.	X	X	X
IPI00021263	YWHAZ	14-3-3 PROTEIN ZETA/DELTA.	X	X	X
IPI00021275	EPHB2	ISOFORM 1 OF EPHRIN TYPE-B RECEPTOR 2 PRECURSOR.	X	X	X
IPI00021304	KRT2	KERATIN, TYPE II CYTOSKELETAL 2 EPIDERMAL.	X	X	X
IPI00021364	CFP	PROPERDIN PRECURSOR.	X	X	X
IPI00021447	AMY2B	ALPHA-AMYLASE 2B PRECURSOR.	X	X	X

Table S1. Proteins Identified or Not in MS Patients Compared to Normals and Other Neurologic Disease (OND)					
**IPI**	**Gene**	**Protein Name**	**MS**	**Normal**	**OND**
IPI00021485	LRRN1	LEUCINE-RICH REPEAT NEURONAL PROTEIN 1 PRECURSOR.	X	X	X
IPI00021552	B3GALNT1	UDP-GALNAC:BETA-1,3-N-ACETYLGALACTOSAMINYLTRANSFERASE 1.	X	X	X
IPI00021727	C4BPA	C4B-BINDING PROTEIN ALPHA CHAIN PRECURSOR.	X	X	X
IPI00021817	PROC	VITAMIN K-DEPENDENT PROTEIN C PRECURSOR.	X	X	X
IPI00021834	TFPI	ISOFORM ALPHA OF TISSUE FACTOR PATHWAY INHIBITOR PRECURSOR.	X	X	X
IPI00021841	APOA1	APOLIPOPROTEIN A-I PRECURSOR.	X	X	X
IPI00021842	APOE	APOLIPOPROTEIN E PRECURSOR.	X	X	X
IPI00021854	APOA2	APOLIPOPROTEIN A-II PRECURSOR.	X	X	X
IPI00021855	APOC1	APOLIPOPROTEIN C-I PRECURSOR.	X	X	X
IPI00021856	APOC2	APOLIPOPROTEIN C-II PRECURSOR.	X	X	X
IPI00021857	APOC3	APOLIPOPROTEIN C-III PRECURSOR.	X	X	X
IPI00021885	FGA	ISOFORM 1 OF FIBRINOGEN ALPHA CHAIN PRECURSOR.	X	X	X
IPI00021891	FGG	ISOFORM GAMMA-B OF FIBRINOGEN GAMMA CHAIN PRECURSOR.	X	X	X
IPI00021900	TNFSF12	TUMOR NECROSIS FACTOR LIGAND SUPERFAMILY MEMBER 12.	X	X	X
IPI00021903	ADAM23	ISOFORM ALPHA OF ADAM 23 PRECURSOR.	X	X	X
IPI00021983	NCSTN	ISOFORM 1 OF NICASTRIN PRECURSOR.	X	X	X
IPI00021985	TM9SF4	TRANSMEMBRANE 9 SUPERFAMILY PROTEIN MEMBER 4.	X	X	X
IPI00021997	CREG1	PROTEIN CREG1 PRECURSOR.	X	X	X
IPI00022039	CD84	ISOFORM 3 OF SLAM FAMILY MEMBER 5 PRECURSOR.	X	X	X
IPI00022229	APOB	APOLIPOPROTEIN B-100 PRECURSOR.	X	X	X
IPI00022284	PRNP	MAJOR PRION PROTEIN PRECURSOR.	X	X	X
IPI00022296	KIT	MAST/STEM CELL GROWTH FACTOR RECEPTOR PRECURSOR.	X	X	X
IPI00022331	LCAT	PHOSPHATIDYLCHOLINE-STEROL ACYLTRANSFERASE PRECURSOR.	X	X	X
IPI00022333	BAI1	BRAIN-SPECIFIC ANGIOGENESIS INHIBITOR 1 PRECURSOR.	X	X	X
IPI00022337	RBP3	INTERPHOTORECEPTOR RETINOID-BINDING PROTEIN PRECURSOR.	X	X	X
IPI00022367	ASTN1	ISOFORM 2 OF ASTROTACTIN-1 PRECURSOR.	X	X	X
IPI00022371	HRG	HISTIDINE-RICH GLYCOPROTEIN PRECURSOR.	X	X	X
IPI00022389	CRP	ISOFORM 1 OF C-REACTIVE PROTEIN PRECURSOR.	X	X	X
IPI00022391	APCS	SERUM AMYLOID P-COMPONENT PRECURSOR.	X	X	X
IPI00022392	C1QA	COMPLEMENT C1Q SUBCOMPONENT SUBUNIT A PRECURSOR.	X	X	X
IPI00022394	C1QC	COMPLEMENT C1Q SUBCOMPONENT SUBUNIT C PRECURSOR.	X	X	X
IPI00022395	C9	COMPLEMENT COMPONENT C9 PRECURSOR.	X	X	X
IPI00022417	LRG1	LEUCINE-RICH ALPHA-2-GLYCOPROTEIN PRECURSOR.	X	X	X
IPI00022420	RBP4	PLASMA RETINOL-BINDING PROTEIN PRECURSOR.	X	X	X

Table S1. Proteins Identified or Not in MS Patients Compared to Normals and Other Neurologic Disease (OND)					
**IPI**	**Gene**	**Protein Name**	**MS**	**Normal**	**OND**
IPI00022426	AMBP	AMBP PROTEIN PRECURSOR.	X	X	X
IPI00022429	ORM1	ALPHA-1-ACID GLYCOPROTEIN 1 PRECURSOR.	X	X	X
IPI00022431	AHSG	ALPHA-2-HS-GLYCOPROTEIN PRECURSOR.	X	X	X
IPI00022432	TTR	TRANSTHYRETIN PRECURSOR.	X	X	X
IPI00022434	ALB	UNCHARACTERIZED PROTEIN ALB.	X	X	X
IPI00022445	PPBP	PLATELET BASIC PROTEIN PRECURSOR.	X	X	X
IPI00022463	TF	SEROTRANSFERRIN PRECURSOR.	X	X	X
IPI00022488	HPX	HEMOPEXIN PRECURSOR.	X	X	X
IPI00022608	SORL1	SORTILIN-RELATED RECEPTOR PRECURSOR.	X	X	X
IPI00022640	NRGN	NEUROGRANIN.	X	X	X
IPI00022649	SLC12A2	ISOFORM 1 OF SOLUTE CARRIER FAMILY 12 MEMBER 2.	X	X	X
IPI00022792	MFAP4	MICROFIBRIL-ASSOCIATED GLYCOPROTEIN 4 PRECURSOR.	X	X	X
IPI00022810	CTSC	DIPEPTIDYL-PEPTIDASE 1 PRECURSOR.	X	X	X
IPI00022822	COL18A1	ISOFORM 2 OF COLLAGEN ALPHA-1(XVIII) CHAIN PRECURSOR.	X	X	X
IPI00022891	SLC25A4	ADP/ATP TRANSLOCASE 1.	X	X	X
IPI00022892	THY1	THY-1 MEMBRANE GLYCOPROTEIN PRECURSOR.	X	X	X
IPI00022895	A1BG	ALPHA-1B-GLYCOPROTEIN PRECURSOR.	X	X	X
IPI00022937	F5	COAGULATION FACTOR V.	X	X	X
IPI00022959	PVRL3	ISOFORM 1 OF POLIOVIRUS RECEPTOR-RELATED PROTEIN 3 PRECURSOR.	X	X	X
IPI00022977	CKB	CREATINE KINASE B-TYPE.	X	X	X
IPI00023014	VWF	VON WILLEBRAND FACTOR PRECURSOR.	X	X	X
IPI00023019	SHBG	ISOFORM 1 OF SEX HORMONE-BINDING GLOBULIN PRECURSOR.	X	X	X
IPI00023359	MCAT	ISOFORM 1 OF MALONYL COA-ACYL CARRIER PROTEIN TRANSACYLASE, MITOCHONDRIAL PRECURSOR.	X	X	X
IPI00023505	FCGR2A	LOW AFFINITY IMMUNOGLOBULIN GAMMA FC REGION RECEPTOR II-A PRECURSOR.	X	X	X
IPI00023542	TMED9	TRANSMEMBRANE EMP24 PROTEIN TRANSPORT DOMAIN CONTAINING 9.	X	X	X
IPI00023576	LRRTM2	LEUCINE-RICH REPEAT TRANSMEMBRANE NEURONAL PROTEIN 2 PRECURSOR.	X	X	X
IPI00023643	SEMA6C	SEMA DOMAIN, TRANSMEMBRANE DOMAIN (TM), AND CYTOPLASMIC DOMAIN, (SEMAPHORIN) 6C.	X	X	X
IPI00023648	ISLR	IMMUNOGLOBULIN SUPERFAMILY CONTAINING LEUCINE-RICH REPEAT PROTEIN PRECURSOR.	X	X	X
IPI00023673	LGALS3BP	GALECTIN-3-BINDING PROTEIN PRECURSOR.	X	X	X
IPI00023728	GGH	GAMMA-GLUTAMYL HYDROLASE PRECURSOR.	X	X	X
IPI00023751	MSTN	GROWTH/DIFFERENTIATION FACTOR 8 PRECURSOR.	X	X	X

Table S1. Proteins Identified or Not in MS Patients Compared to Normals and Other Neurologic Disease (OND)					
**IPI**	**Gene**	**Protein Name**	**MS**	**Normal**	**OND**
IPI00023807	SEMA4D	SEMAPHORIN-4D PRECURSOR.	X	X	X
IPI00023824	FBLN2	FIBULIN-2 PRECURSOR.	X	X	X
IPI00023845	KLK6	KALLIKREIN-6 PRECURSOR.	X	X	X
IPI00023858	FCGR3B	FC-GAMMA RECEPTOR IIIB.	X	X	X
IPI00024012	FZD7	FRIZZLED-7 PRECURSOR.	X	X	X
IPI00024034	CDH4	CADHERIN-4 PRECURSOR.	X	X	X
IPI00024035	CDH6	ISOFORM 1 OF CADHERIN-6 PRECURSOR.	X	X	X
IPI00024036	CDH8	CADHERIN-8 PRECURSOR.	X	X	X
IPI00024046	CDH13	CADHERIN-13 PRECURSOR.	X	X	X
IPI00024048	CDH15	CADHERIN-15 PRECURSOR.	X	X	X
IPI00024105	C1QTNF5	COMPLEMENT C1Q TUMOR NECROSIS FACTOR-RELATED PROTEIN 5 PRECURSOR.	X	X	X
IPI00024129	PPIC	PEPTIDYL-PROLYL CIS-TRANS ISOMERASE C.	X	X	X
IPI00024272	DGCR2	INTEGRAL MEMBRANE PROTEIN DGCR2/IDD PRECURSOR.	X	X	X
IPI00024284	HSPG2	BASEMENT MEMBRANE-SPECIFIC HEPARAN SULFATE PROTEOGLYCAN CORE PROTEIN PRECURSOR.	X	X	X
IPI00024307	EFNB1	EPHRIN-B1 PRECURSOR.	X	X	X
IPI00024466	UGCGL1	UDP-GLUCOSE CERAMIDE GLUCOSYLTRANSFERASE-LIKE 1 ISOFORM 1.	X	X	X
IPI00024570	SEMA3G	SEMAPHORIN-3G PRECURSOR.	X	X	X
IPI00024572	ASPH	ASPARTATE BETA-HYDROXYLASE ISOFORM E.	X	X	X
IPI00024587	CALY	D1 DOPAMINE RECEPTOR-INTERACTING PROTEIN CALCYON.	X	X	X
IPI00024601	CA10	CARBONIC ANHYDRASE-RELATED PROTEIN 10.	X	X	X
IPI00024621	OLFML3	ISOFORM 1 OF OLFACTOMEDIN-LIKE PROTEIN 3 PRECURSOR.	X	X	X
IPI00024825	PRG4	ISOFORM A OF PROTEOGLYCAN-4 PRECURSOR.	X	X	X
IPI00024853	PRX	ISOFORM 1 OF PERIAXIN.	X	X	X
IPI00024966	CNTN2	CONTACTIN-2 PRECURSOR.	X	X	X
IPI00025092	MYBPC1	MYOSIN-BINDING PROTEIN C, SLOW-TYPE.	X	X	X
IPI00025110	MSLN	ISOFORM 2 OF MESOTHELIN PRECURSOR.	X	X	X
IPI00025204	CD5L	CD5 ANTIGEN-LIKE PRECURSOR.	X	X	X
IPI00025252	PDIA3	PROTEIN DISULFIDE-ISOMERASE A3 PRECURSOR.	X	X	X
IPI00025257	SEMA7A	SEMAPHORIN-7A PRECURSOR.	X	X	X
IPI00025276	TNXB	ISOFORM XB OF TENASCIN-X PRECURSOR.	X	X	X
IPI00025318	SH3BGRL	SH3 DOMAIN-BINDING GLUTAMIC ACID-RICH-LIKE PROTEIN.	X	X	X
IPI00025363	GFAP	ISOFORM 1 OF GLIAL FIBRILLARY ACIDIC PROTEIN.	X	X	X
IPI00025365	EDN3	ISOFORM LONG OF ENDOTHELIN-3 PRECURSOR.	X	X	X

Table S1. Proteins Identified or Not in MS Patients Compared to Normals and Other Neurologic Disease (OND)					
**IPI**	**Gene**	**Protein Name**	**MS**	**Normal**	**OND**
IPI00025426	PZP	PREGNANCY ZONE PROTEIN PRECURSOR.	X	X	X
IPI00025465	OGN	MIMECAN PRECURSOR.	X	X	X
IPI00025809	MGAT2	ALPHA-1,6-MANNOSYL-GLYCOPROTEIN 2-BETA-N-ACETYLGLUCOSAMINYLTRANSFERASE.	X	X	X
IPI00025812	CA11	CARBONIC ANHYDRASE-RELATED PROTEIN 11 PRECURSOR.	X	X	X
IPI00025818	GALNT1	ISOFORM 1 OF POLYPEPTIDE N-ACETYLGALACTOSAMINYLTRANSFERASE 1.	X	X	X
IPI00025840	EFNA1	ISOFORM 1 OF EPHRIN-A1 PRECURSOR.	X	X	X
IPI00025846	DSC2	ISOFORM 2A OF DESMOCOLLIN-2 PRECURSOR.	X	X	X
IPI00025854	SERPINA2	SIMILAR TO ALPHA-1-ANTITRYPSIN-RELATED PROTEIN PRECURSOR.	X	X	X
IPI00025864	BCHE	CHOLINESTERASE PRECURSOR.	X	X	X
IPI00026050	CLN5	CEROID-LIPOFUSCINOSIS NEURONAL PROTEIN 5.	X	X	X
IPI00026103	ACHE	ACHE PROTEIN.	X	X	X
IPI00026104	IDS	ISOFORM LONG OF IDURONATE 2-SULFATASE PRECURSOR.	X	X	X
IPI00026154	PRKCSH	GLUCOSIDASE 2 SUBUNIT BETA PRECURSOR.	X	X	X
IPI00026174	CCK	CHOLECYSTOKININS PRECURSOR.	X	X	X
IPI00026197	IGKV4-1	SIMILAR TO IG KAPPA CHAIN V-IV REGION PRECURSOR.	X	X	X
IPI00026199	GPX3	GLUTATHIONE PEROXIDASE 3 PRECURSOR.	X	X	X
IPI00026216	NPEPPS	PUROMYCIN-SENSITIVE AMINOPEPTIDASE.	X	X	X
IPI00026237	MAG	MYELIN-ASSOCIATED GLYCOPROTEIN PRECURSOR.	X	X	X
IPI00026240	BST1	ADP-RIBOSYL CYCLASE 2 PRECURSOR.	X	X	X
IPI00026259	AGA	N.	X	X	X
IPI00026270	CPM	CARBOXYPEPTIDASE M PRECURSOR.	X	X	X
IPI00026314	GSN	ISOFORM 1 OF GELSOLIN PRECURSOR.	X	X	X
IPI00026530	LMAN1	PROTEIN ERGIC-53 PRECURSOR.	X	X	X
IPI00026800	SCRG1	SCRAPIE-RESPONSIVE PROTEIN 1 PRECURSOR.	X	X	X
IPI00026944	NID1	ISOFORM 1 OF NIDOGEN-1 PRECURSOR.	X	X	X
IPI00026946	NPTX2	NEURONAL PENTRAXIN-2 PRECURSOR.	X	X	X
IPI00026991	GALNT6	POLYPEPTIDE N-ACETYLGALACTOSAMINYLTRANSFERASE 6.	X	X	X
IPI00027038	VSIG4	ISOFORM 1 OF V-SET AND IMMUNOGLOBULIN DOMAIN-CONTAINING PROTEIN 4 PRECURSOR.	X	X	X
IPI00027078	CPD	CARBOXYPEPTIDASE D PRECURSOR.	X	X	X
IPI00027087	L1CAM	ISOFORM 1 OF NEURAL CELL ADHESION MOLECULE L1 PRECURSOR.	X	X	X
IPI00027166	TIMP2	METALLOPROTEINASE INHIBITOR 2 PRECURSOR.	X	X	X
IPI00027174	FGFR3	ISOFORM 1 OF FIBROBLAST GROWTH FACTOR RECEPTOR 3 PRECURSOR.	X	X	X

Table S1. Proteins Identified or Not in MS Patients Compared to Normals and Other Neurologic Disease (OND)					
**IPI**	**Gene**	**Protein Name**	**MS**	**Normal**	**OND**
IPI00027192	PLOD1	PROCOLLAGEN-LYSINE,2-OXOGLUTARATE 5-DIOXYGENASE 1 PRECURSOR.	X	X	X
IPI00027230	HSP90B1	ENDOPLASMIN PRECURSOR.	X	X	X
IPI00027310	MEGF8	ISOFORM 1 OF MULTIPLE EPIDERMAL GROWTH FACTOR-LIKE DOMAINS 8.	X	X	X
IPI00027341	CAPG	MACROPHAGE-CAPPING PROTEIN.	X	X	X
IPI00027350	PRDX2	PEROXIREDOXIN-2.	X	X	X
IPI00027377	ACAN	AGGRECAN ISOFORM 2 PRECURSOR.	X	X	X
IPI00027462	S100A9	PROTEIN S100-A9.	X	X	X
IPI00027466	CA4	CARBONIC ANHYDRASE 4 PRECURSOR.	X	X	X
IPI00027482	SERPINA6	CORTICOSTEROID-BINDING GLOBULIN PRECURSOR.	X	X	X
IPI00027493	LOC442497; SLC3A2	4F2 CELL-SURFACE ANTIGEN HEAVY CHAIN.	X	X	X
IPI00027497	GPI	GLUCOSE-6-PHOSPHATE ISOMERASE.	X	X	X
IPI00027703	MAN2A2	ISOFORM LONG OF ALPHA-MANNOSIDASE IIX.	X	X	X
IPI00027780	MMP2	72 KDA TYPE IV COLLAGENASE PRECURSOR.	X	X	X
IPI00027827	SOD3	EXTRACELLULAR SUPEROXIDE DISMUTASE [CU-ZN] PRECURSOR.	X	X	X
IPI00027847	LPL	LIPOPROTEIN LIPASE PRECURSOR.	X	X	X
IPI00027848	MRC1	MACROPHAGE MANNOSE RECEPTOR 1 PRECURSOR.	X	X	X
IPI00027851	HEXA	BETA-HEXOSAMINIDASE ALPHA CHAIN PRECURSOR.	X	X	X
IPI00027972	LILRA2	ISOFORM 1 OF LEUKOCYTE IMMUNOGLOBULIN-LIKE RECEPTOR SUBFAMILY A MEMBER 2 PRECURSOR.	X	X	X
IPI00028015	LAIR1	ISOFORM 2 OF LEUKOCYTE-ASSOCIATED IMMUNOGLOBULIN-LIKE RECEPTOR 1 PRECURSOR.	X	X	X
IPI00028193	KNDC1	192 KDA PROTEIN.	X	X	X
IPI00028381	DLK2	ISOFORM 1 OF DELTA-LIKE PROTEIN 2 PRECURSOR.	X	X	X
IPI00028387	C20orf116	ISOFORM 1 OF UNCHARACTERIZED PROTEIN C20ORF116 PRECURSOR.	X	X	X
IPI00028413	ITIH3	ISOFORM 1 OF INTER-ALPHA-TRYPSIN INHIBITOR HEAVY CHAIN H3 PRECURSOR.	X	X	X
IPI00028553	MINPP1	ISOFORM 2 OF MULTIPLE INOSITOL POLYPHOSPHATE PHOSPHATASE 1 PRECURSOR.	X	X	X
IPI00028714	MGP	MATRIX GLA PROTEIN PRECURSOR.	X	X	X
IPI00028908	NID2	NIDOGEN-2 PRECURSOR.	X	X	X
IPI00028911	DAG1	DYSTROGLYCAN PRECURSOR.	X	X	X
IPI00028931	DSG2	DESMOGLEIN-2 PRECURSOR.	X	X	X
IPI00029046	KIAA0152	UNCHARACTERIZED PROTEIN KIAA0152 PRECURSOR.	X	X	X
IPI00029050	LARGE	ISOFORM 1 OF GLYCOSYLTRANSFERASE-LIKE PROTEIN LARGE1.	X	X	X
IPI00029061	SEPP1	SELENOPROTEIN P PRECURSOR.	X	X	X

Table S1. Proteins Identified or Not in MS Patients Compared to Normals and Other Neurologic Disease (OND)					
**IPI**	**Gene**	**Protein Name**	**MS**	**Normal**	**OND**
IPI00029131	PCSK2	NEUROENDOCRINE CONVERTASE 2 PRECURSOR.	X	X	X
IPI00029193	HGFAC	HEPATOCYTE GROWTH FACTOR ACTIVATOR PRECURSOR.	X	X	X
IPI00029235	IGFBP6	INSULIN-LIKE GROWTH FACTOR-BINDING PROTEIN 6 PRECURSOR.	X	X	X
IPI00029236	IGFBP5	INSULIN-LIKE GROWTH FACTOR-BINDING PROTEIN 5 PRECURSOR.	X	X	X
IPI00029260	CD14	MONOCYTE DIFFERENTIATION ANTIGEN CD14 PRECURSOR.	X	X	X
IPI00029273	MET	ISOFORM 1 OF HEPATOCYTE GROWTH FACTOR RECEPTOR PRECURSOR.	X	X	X
IPI00029343	CNTNAP2	ISOFORM 1 OF CONTACTIN-ASSOCIATED PROTEIN-LIKE 2 PRECURSOR.	X	X	X
IPI00029605	GALNS	N-ACETYLGALACTOSAMINE-6-SULFATASE PRECURSOR.	X	X	X
IPI00029606	ADAM17	ISOFORM B OF ADAM 17 PRECURSOR.	X	X	X
IPI00029658	EFEMP1	ISOFORM 1 OF EGF-CONTAINING FIBULIN-LIKE EXTRACELLULAR MATRIX PROTEIN 1 PRECURSOR.	X	X	X
IPI00029722	KIF5A	KINESIN HEAVY CHAIN ISOFORM 5A.	X	X	X
IPI00029723	FSTL1	FOLLISTATIN-RELATED PROTEIN 1 PRECURSOR.	X	X	X
IPI00029739	CFH	ISOFORM 1 OF COMPLEMENT FACTOR H PRECURSOR.	X	X	X
IPI00029751	CNTN1	ISOFORM 1 OF CONTACTIN-1 PRECURSOR.	X	X	X
IPI00029756	MERTK	PROTO-ONCOGENE TYROSINE-PROTEIN KINASE MER PRECURSOR.	X	X	X
IPI00029817	NEU1	SIALIDASE-1 PRECURSOR.	X	X	X
IPI00029819	NOTCH3	NEUROGENIC LOCUS NOTCH HOMOLOG PROTEIN 3 PRECURSOR.	X	X	X
IPI00029863	SERPINF2	SERPINF2 PROTEIN.	X	X	X
IPI00029997	PGLS	6-PHOSPHOGLUCONOLACTONASE.	X	X	X
IPI00030075	FGL2	FIBROLEUKIN PRECURSOR.	X	X	X
IPI00030111	GDF11	GROWTH/DIFFERENTIATION FACTOR 11 PRECURSOR.	X	X	X
IPI00030255	PLOD3	PROCOLLAGEN-LYSINE,2-OXOGLUTARATE 5-DIOXYGENASE 3 PRECURSOR.	X	X	X
IPI00030634	GGTL3	ISOFORM 1 OF GAMMA-GLUTAMYLTRANSFERASE 4 PRECURSOR.	X	X	X
IPI00030739	APOM	APOLIPOPROTEIN M.	X	X	X
IPI00030887	TYRO3	TYROSINE-PROTEIN KINASE RECEPTOR TYRO3 PRECURSOR.	X	X	X
IPI00031008	TNC	ISOFORM 1 OF TENASCIN PRECURSOR.	X	X	X
IPI00031086	IGFBP1	INSULIN-LIKE GROWTH FACTOR-BINDING PROTEIN 1 PRECURSOR.	X	X	X
IPI00031121	CPE	CARBOXYPEPTIDASE E PRECURSOR.	X	X	X
IPI00031461	GDI2	RAB GDP DISSOCIATION INHIBITOR BETA.	X	X	X
IPI00031510	SEMA3A	SEMAPHORIN-3A PRECURSOR.	X	X	X
IPI00031549	DSC3	ISOFORM 3A OF DESMOCOLLIN-3 PRECURSOR.	X	X	X
IPI00031564	C7orf24	UNCHARACTERIZED PROTEIN C7ORF24.	X	X	X
IPI00031708	FAH	FUMARYLACETOACETASE.	X	X	X

Table S1. Proteins Identified or Not in MS Patients Compared to Normals and Other Neurologic Disease (OND)					
**IPI**	**Gene**	**Protein Name**	**MS**	**Normal**	**OND**
IPI00031718	ENTPD4	ISOFORM 1 OF ECTONUCLEOSIDE TRIPHOSPHATE DIPHOSPHOHYDROLASE 4.	X	X	X
IPI00031769	C2orf40	ESOPHAGEAL CANCER-RELATED GENE 4 PROTEIN PRECURSOR.	X	X	X
IPI00031789	IL1RAP	ISOFORM 1 OF INTERLEUKIN-1 RECEPTOR ACCESSORY PROTEIN PRECURSOR.	X	X	X
IPI00031821	ITM2B	INTEGRAL MEMBRANE PROTEIN 2B.	X	X	X
IPI00032179	SERPINC1	ANTITHROMBIN III VARIANT.	X	X	X
IPI00032187	NISCH	NISCHARIN.	X	X	X
IPI00032220	AGT	ANGIOTENSINOGEN PRECURSOR.	X	X	X
IPI00032258	C4A; C4B	COMPLEMENT C4-A PRECURSOR.	X	X	X
IPI00032288	MANSC1	MANSC DOMAIN-CONTAINING PROTEIN 1 PRECURSOR.	X	X	X
IPI00032291	C5	COMPLEMENT C5 PRECURSOR.	X	X	X
IPI00032292	TIMP1	METALLOPROTEINASE INHIBITOR 1 PRECURSOR.	X	X	X
IPI00032293	CST3	CYSTATIN-C PRECURSOR.	X	X	X
IPI00032328	KNG1	ISOFORM HMW OF KININOGEN-1 PRECURSOR.	X	X	X
IPI00032405	GPR37L1	ENDOTHELIN B RECEPTOR-LIKE PROTEIN 2 PRECURSOR.	X	X	X
IPI00032532	GAS6	ISOFORM 2 OF GROWTH ARREST-SPECIFIC PROTEIN 6 PRECURSOR.	X	X	X
IPI00032826	ST13	HSC70-INTERACTING PROTEIN.	X	X	X
IPI00033466	CLEC11A	C-TYPE LECTIN DOMAIN FAMILY 11 MEMBER A PRECURSOR.	X	X	X
IPI00033560	PTPRR	ISOFORM ALPHA OF RECEPTOR-TYPE TYROSINE-PROTEIN PHOSPHATASE R PRECURSOR.	X	X	X
IPI00034319	CUTA	ISOFORM A OF PROTEIN CUTA PRECURSOR.	X	X	X
IPI00043215	IGSF1	IMMUNOGLOBULIN SUPERFAMILY, MEMBER 1 ISOFORM 1.	X	X	X
IPI00043756	SLC39A12	ISOFORM 3 OF ZINC TRANSPORTER ZIP12.	X	X	X
IPI00043810	PRRT1	ISOFORM 1 OF PROLINE-RICH TRANSMEMBRANE PROTEIN 1.	X	X	X
IPI00044369	PLXDC2	ISOFORM 1 OF PLEXIN DOMAIN-CONTAINING PROTEIN 2 PRECURSOR.	X	X	X
IPI00044600	SORCS2	VPS10 DOMAIN-CONTAINING RECEPTOR SORCS2 PRECURSOR.	X	X	X
IPI00044743	TMEM132B	ISOFORM 1 OF TRANSMEMBRANE PROTEIN 132B.	X	X	X
IPI00044751	MPHOSPH1	ISOFORM 1 OF M-PHASE PHOSPHOPROTEIN 1.	X	X	X
IPI00045536	CHID1	ISOFORM 3 OF CHITINASE DOMAIN-CONTAINING PROTEIN 1 PRECURSOR.	X	X	X
IPI00045841	LRP11	ISOFORM 1 OF LOW-DENSITY LIPOPROTEIN RECEPTOR-RELATED PROTEIN 11 PRECURSOR.	X	X	X
IPI00056357	C19orf10	UNCHARACTERIZED PROTEIN C19ORF10 PRECURSOR.	X	X	X
IPI00056478	IGSF8	ISOFORM 1 OF IMMUNOGLOBULIN SUPERFAMILY MEMBER 8 PRECURSOR.	X	X	X
IPI00059366	H2AFY	H2A HISTONE FAMILY, MEMBER Y ISOFORM 2.	X	X	X
IPI00060310	PLD4	PHOSPHOLIPASE D4.	X	X	X

Table S1. Proteins Identified or Not in MS Patients Compared to Normals and Other Neurologic Disease (OND)					
IPI	Gene	Protein Name	MS	Normal	OND
IPI00060715	KCTD12	BTB/POZ DOMAIN-CONTAINING PROTEIN KCTD12.	X	X	X
IPI00061354	BAZ2B	ISOFORM 2 OF BROMODOMAIN ADJACENT TO ZINC FINGER DOMAIN PROTEIN 2B.	X	X	X
IPI00061977	IGHA1; IGHV3OR16-13	IGHA1 PROTEIN.	X	X	X
IPI00063048	ST6GAL2	ISOFORM 2 OF BETA-GALACTOSIDE ALPHA-2,6-SIALYLTRANSFERASE 2.	X	X	X
IPI00064377	RELT	TUMOR NECROSIS FACTOR RECEPTOR SUPERFAMILY MEMBER 19L PRECURSOR.	X	X	X
IPI00064607	MEGF10	ISOFORM 1 OF MULTIPLE EPIDERMAL GROWTH FACTOR-LIKE DOMAINS 10 PRECURSOR.	X	X	X
IPI00064667	CNDP1	BETA-ALA-HIS DIPEPTIDASE PRECURSOR.	X	X	X
IPI00064935	ALPK3	ALPHA-PROTEIN KINASE 3.	X	X	X
IPI00065931	AKAP13	ISOFORM 2 OF A-KINASE ANCHOR PROTEIN 13.	X	X	X
IPI00069058	VGF	VGF NERVE GROWTH FACTOR INDUCIBLE PRECURSOR.	X	X	X
IPI00072917	COL6A3	ALPHA 3 TYPE VI COLLAGEN ISOFORM 3 PRECURSOR.	X	X	X
IPI00073763	SEMA4C	SEMAPHORIN-4C PRECURSOR.	X	X	X
IPI00075248	CALM1; CALM2; CALM3	CALMODULIN.	X	X	X
IPI00099670	CEL	CARBOXYL ESTER LIPASE PRECURSOR.	X	X	X
IPI00101608	CRELD1	ISOFORM 2 OF CYSTEINE-RICH WITH EGF-LIKE DOMAIN PROTEIN 1 PRECURSOR.	X	X	X
IPI00102435	COL21A1	COLLAGEN, TYPE XXI, ALPHA 1 PRECURSOR.	X	X	X
IPI00102543	SLITRK1	SLIT AND NTRK-LIKE PROTEIN 1 PRECURSOR.	X	X	X
IPI00102575	ATAD5	ATPASE FAMILY, AAA DOMAIN CONTAINING 5.	X	X	X
IPI00103175	CANT1	ISOFORM 1 OF SOLUBLE CALCIUM-ACTIVATED NUCLEOTIDASE 1.	X	X	X
IPI00103471	SELM	THIOREDOXIN-LIKE SELENOPROTEIN M PRECURSOR.	X	X	X
IPI00103552	MUC16	MUCIN-16.	X	X	X
IPI00103597	SORCS1	ISOFORM 1 OF VPS10 DOMAIN-CONTAINING RECEPTOR SORCS1 PRECURSOR.	X	X	X
IPI00103755	UNC5D	ISOFORM 2 OF NETRIN RECEPTOR UNC5D PRECURSOR.	X	X	X
IPI00104074	CD163	ISOFORM 1 OF SCAVENGER RECEPTOR CYSTEINE-RICH TYPE 1 PROTEIN M130 PRECURSOR.	X	X	X
IPI00106502	KEAP1	KELCH-LIKE ECH-ASSOCIATED PROTEIN 1.	X	X	X
IPI00107819	PTPRD	PTPRD PROTEIN.	X	X	X
IPI00107831	PTPRF	RECEPTOR-TYPE TYROSINE-PROTEIN PHOSPHATASE F PRECURSOR.	X	X	X
IPI00149097	SEMA4A	SEMAPHORIN-4A PRECURSOR.	X	X	X
IPI00150881	C6orf204	ISOFORM 1 OF COILED-COIL DOMAIN-CONTAINING PROTEIN C6ORF204.	X	X	X

Table S1. Proteins Identified or Not in MS Patients Compared to Normals and Other Neurologic Disease (OND)					
**IPI**	**Gene**	**Protein Name**	**MS**	**Normal**	**OND**
IPI00152418	CD55	DECAY-ACCELERATING FACTOR SPLICING VARIANT 4.	X	X	X
IPI00152524	NETO1	ISOFORM 3 OF NEUROPILIN AND TOLLOID-LIKE PROTEIN 1 PRECURSOR.	X	X	X
IPI00152540	CD109	ISOFORM 1 OF CD109 ANTIGEN PRECURSOR.	X	X	X
IPI00152847	WFIKKN2	WAP, KAZAL, IMMUNOGLOBULIN, KUNITZ AND NTR DOMAIN-CONTAINING PROTEIN 2 PRECURSOR.	X	X	X
IPI00152850	JAM3	JUNCTIONAL ADHESION MOLECULE 3 PRECURSOR.	X	X	X
IPI00153049	MXRA8	ISOFORM 2 OF MATRIX-REMODELING-ASSOCIATED PROTEIN 8 PRECURSOR.	X	X	X
IPI00154734	SEZ6	SEIZURE RELATED 6 HOMOLOG ISOFORM 1.	X	X	X
IPI00154742	IGL@	IGL@ PROTEIN.	X	X	X
IPI00154858	PEAR1	PLATELET ENDOTHELIAL AGGREGATION RECEPTOR 1 PRECURSOR.	X	X	X
IPI00155729	PLXNB3	PLEXIN-B3 PRECURSOR.	X	X	X
IPI00156171	ENPP2	ISOFORM 1 OF ECTONUCLEOTIDE PYROPHOSPHATASE/PHOSPHODIESTERASE FAMILY MEMBER 2 PRECURSOR.	X	X	X
IPI00157414	ENPP6	ECTONUCLEOTIDE PYROPHOSPHATASE/PHOSPHODIESTERASE FAMILY MEMBER 6 PRECURSOR.	X	X	X
IPI00159927	NCAN	NEUROCAN CORE PROTEIN PRECURSOR.	X	X	X
IPI00160552	TNR	ISOFORM 1 OF TENASCIN-R PRECURSOR.	X	X	X
IPI00162329	TMEM25	ISOFORM 1 OF TRANSMEMBRANE PROTEIN 25 PRECURSOR.	X	X	X
IPI00162547	LPHN3	LATROPHILIN 3 PRECURSOR.	X	X	X
IPI00163207	PGLYRP2	ISOFORM 1 OF N-ACETYLMURAMOYL-L-ALANINE AMIDASE PRECURSOR.	X	X	X
IPI00163563	PEBP4	PEBP FAMILY PROTEIN PRECURSOR.	X	X	X
IPI00163724	HCN3	POTASSIUM/SODIUM HYPERPOLARIZATION-ACTIVATED CYCLIC NUCLEOTIDE-GATED CHANNEL 3.	X	X	X
IPI00165044	C4orf18	ISOFORM 2 OF UNCHARACTERIZED PROTEIN C4ORF18.	X	X	X
IPI00165125	C14orf37	ISOFORM 1 OF UNCHARACTERIZED PROTEIN C14ORF37 PRECURSOR.	X	X	X
IPI00165438	NRP1	MUSCLE TYPE NEUROPILIN 1.	X	X	X
IPI00165949	ERAP1	ISOFORM 2 OF ENDOPLASMIC RETICULUM AMINOPEPTIDASE 1.	X	X	X
IPI00165972	CFD	COMPLEMENT FACTOR D PREPROPROTEIN.	X	X	X
IPI00165975	ISLR2	CYSTEINE-RICH FLANKING REGION, C-TERMINAL DOMAIN CONTAINING PROTEIN.	X	X	X
IPI00166039	SCOTIN	ISOFORM 1 OF SCOTIN PRECURSOR.	X	X	X
IPI00166075	LGI3	LEUCINE-RICH REPEAT LGI FAMILY MEMBER 3 PRECURSOR.	X	X	X
IPI00166339	EPHA10	ISOFORM 1 OF EPHRIN TYPE-A RECEPTOR 10 PRECURSOR.	X	X	X
IPI00166392	CADM1	IMMUNOGLOBULIN SUPERFAMILY MEMBER 4.	X	X	X

Table S1. Proteins Identified or Not in MS Patients Compared to Normals and Other Neurologic Disease (OND)					
**IPI**	**Gene**	**Protein Name**	**MS**	**Normal**	**OND**
IPI00166613	GALNTL1	ISOFORM 1 OF PUTATIVE POLYPEPTIDE N-ACETYLGALACTOSAMINYLTRANSFERASE-LIKE PROTEIN 1.	X	X	X
IPI00166622	TMEM132C	SIMILAR TO CG14446-PA.	X	X	X
IPI00166729	AZGP1	ALPHA-2-GLYCOPROTEIN 1, ZINC.	X	X	X
IPI00166766	MGC45438	HYPOTHETICAL PROTEIN LOC146556 ISOFORM 2.	X	X	X
IPI00167093	CFHR1	COMPLEMENT FACTOR H-RELATED 1.	X	X	X
IPI00167215	HEPACAM	ISOFORM 1 OF HEPATOCYTE CELL ADHESION MOLECULE PRECURSOR.	X	X	X
IPI00167619	LRTM2	LEUCINE-RICH REPEAT AND TRANSMEMBRANE DOMAIN-CONTAINING PROTEIN 2 PRECURSOR.	X	X	X
IPI00167710	FBLN7	ISOFORM 1 OF FIBULIN-7 PRECURSOR.	X	X	X
IPI00168479	APOA1BP	ISOFORM 1 OF APOLIPOPROTEIN A-I-BINDING PROTEIN PRECURSOR.	X	X	X
IPI00168626	GALNTL4	ISOFORM 1 OF PUTATIVE POLYPEPTIDE N-ACETYLGALACTOSAMINYLTRANSFERASE-LIKE PROTEIN 4.	X	X	X
IPI00168847	HYAL1	ISOFORM 2 OF HYALURONIDASE-1 PRECURSOR.	X	X	X
IPI00168866	MDGA1	MAM DOMAIN CONTAINING GLYCOSYLPHOSPHATIDYLINOSITOL ANCHOR 1.	X	X	X
IPI00168884	ATP6AP2	RENIN RECEPTOR PRECURSOR.	X	X	X
IPI00169285	P76	PUTATIVE PHOSPHOLIPASE B-LIKE 2 PRECURSOR.	X	X	X
IPI00169383	PGK1	PHOSPHOGLYCERATE KINASE 1.	X	X	X
IPI00170692	VAPA	VESICLE-ASSOCIATED MEMBRANE PROTEIN-ASSOCIATED PROTEIN A.	X	X	X
IPI00171410	C3orf21	ISOFORM 1 OF UNCHARACTERIZED PROTEIN C3ORF21.	X	X	X
IPI00171411	GOLM1	GOLGI PHOSPHOPROTEIN 2.	X	X	X
IPI00171412	SUMF2	ISOFORM 1 OF SULFATASE-MODIFYING FACTOR 2 PRECURSOR.	X	X	X
IPI00171438	MUTED; TXNDC5	THIOREDOXIN DOMAIN-CONTAINING PROTEIN 5 PRECURSOR.	X	X	X
IPI00171473	SPON1	SPONDIN-1 PRECURSOR.	X	X	X
IPI00171928	ANGPTL7	ANGIOPOIETIN-RELATED PROTEIN 7 PRECURSOR.	X	X	X
IPI00172450	CAMK2G	ISOFORM 4 OF CALCIUM/CALMODULIN-DEPENDENT PROTEIN KINASE TYPE II GAMMA CHAIN.	X	X	X
IPI00176193	COL14A1	ISOFORM 1 OF COLLAGEN ALPHA-1(XIV) CHAIN PRECURSOR.	X	X	X
IPI00176221	NEGR1	NEURONAL GROWTH REGULATOR 1 PRECURSOR.	X	X	X
IPI00176424	NLGN2	NEUROLIGIN-2 PRECURSOR.	X	X	X
IPI00176427	CADM4	CELL ADHESION MOLECULE 4 PRECURSOR.	X	X	X
IPI00176458	PCDH1	PROTOCADHERIN 1 ISOFORM 2 PRECURSOR.	X	X	X
IPI00176581	FANCM	ISOFORM 1 OF FANCONI ANEMIA GROUP M PROTEIN.	X	X	X

Table S1. Proteins Identified or Not in MS Patients Compared to Normals and Other Neurologic Disease (OND)					
**IPI**	**Gene**	**Protein Name**	**MS**	**Normal**	**OND**
IPI00177543	PAM	PEPTIDYLGLYCINE ALPHA-AMIDATING MONOOXYGENASE ISOFORM A, PREPROPROTEIN.	X	X	X
IPI00178926	IGJ	IMMUNOGLOBULIN J CHAIN.	X	X	X
IPI00181079	METRNL	METEORIN-LIKE PROTEIN PRECURSOR.	X	X	X
IPI00182194	ODZ2	TENEURIN-2.	X	X	X
IPI00182944	CAMK2B	ISOFORM 3 OF CALCIUM/CALMODULIN-DEPENDENT PROTEIN KINASE TYPE II BETA CHAIN.	X	X	X
IPI00183321	GALNAC4S-6ST	ISOFORM 1 OF N-ACETYLGALACTOSAMINE 4-SULFATE 6-O-SULFOTRANSFERASE.	X	X	X
IPI00183445	LPHN1	ISOFORM 1 OF LATROPHILIN-1 PRECURSOR.	X	X	X
IPI00183487	XYLT1	XYLOSYLTRANSFERASE 1.	X	X	X
IPI00184019	PILRA	ISOFORM 3 OF PAIRED IMMUNOGLOBULIN-LIKE TYPE 2 RECEPTOR ALPHA PRECURSOR.	X	X	X
IPI00184094	B3GNT8	UDP-GLCNAC:BETAGAL BETA-1,3-N-ACETYLGLUCOSAMINYLTRANSFERASE 8.	X	X	X
IPI00184851	ST3GAL6	TYPE 2 LACTOSAMINE ALPHA-2,3-SIALYLTRANSFERASE.	X	X	X
IPI00185661	USP32	UBIQUITIN CARBOXYL-TERMINAL HYDROLASE 32.	X	X	X
IPI00186903	APOL1	ISOFORM 2 OF APOLIPOPROTEIN-L1 PRECURSOR.	X	X	X
IPI00215767	B4GALT1	ISOFORM LONG OF BETA-1,4-GALACTOSYLTRANSFERASE 1.	X	X	X
IPI00215983	CA1	CARBONIC ANHYDRASE 1.	X	X	X
IPI00215997	CD9	CD9 ANTIGEN.	X	X	X
IPI00216138	TAGLN	TRANSGELIN.	X	X	X
IPI00216171	ENO2	GAMMA-ENOLASE.	X	X	X
IPI00216298	TXN	THIOREDOXIN.	X	X	X
IPI00216318	YWHAB	ISOFORM LONG OF 14-3-3 PROTEIN BETA/ALPHA.	X	X	X
IPI00216319	YWHAH	14-3-3 PROTEIN ETA.	X	X	X
IPI00216461	ACYP2	ACYLPHOSPHATASE-2.	X	X	X
IPI00216691	PFN1	PROFILIN-1.	X	X	X
IPI00216728	NRXN3	NEUREXIN 3-ALPHA.	X	X	X
IPI00216774	CBLN2	CEREBELLIN-2.	X	X	X
IPI00216882	MASP1	MANNAN-BINDING LECTIN SERINE PROTEASE 1 ISOFORM 3.	X	X	X
IPI00216983	CA3	CARBONIC ANHYDRASE 3.	X	X	X
IPI00217023	MMAA	MMAA PROTEIN.	X	X	X
IPI00217146	SLITRK4	SLIT AND NTRK-LIKE PROTEIN 4 PRECURSOR.	X	X	X

Table S1. Proteins Identified or Not in MS Patients Compared to Normals and Other Neurologic Disease (OND)					
**IPI**	**Gene**	**Protein Name**	**MS**	**Normal**	**OND**
IPI00217345	B3GNT2	ISOFORM 2 OF UDP-GLCNAC:BETAGAL BETA-1,3-N-ACETYLGLUCOSAMINYLTRANSFERASE 2.	X	X	X
IPI00217466	HIST1H1D	HISTONE H1.3.	X	X	X
IPI00217493	MB	MYOGLOBIN.	X	X	X
IPI00217759	FUT11	ISOFORM 1 OF ALPHA-(1,3)-FUCOSYLTRANSFERASE 11.	X	X	X
IPI00217882	SORT1	SORTILIN PRECURSOR.	X	X	X
IPI00217963	KRT16	KERATIN, TYPE I CYTOSKELETAL 16.	X	X	X
IPI00218046	HS6ST3	HEPARAN-SULFATE 6-O-SULFOTRANSFERASE 3.	X	X	X
IPI00218192	ITIH4	ISOFORM 2 OF INTER-ALPHA-TRYPSIN INHIBITOR HEAVY CHAIN H4 PRECURSOR.	X	X	X
IPI00218398	MMP14	MATRIX METALLOPROTEINASE-14 PRECURSOR.	X	X	X
IPI00218407	ALDOB	FRUCTOSE-BISPHOSPHATE ALDOLASE B.	X	X	X
IPI00218413	BTD	BIOTINIDASE PRECURSOR.	X	X	X
IPI00218414	CA2	CARBONIC ANHYDRASE 2.	X	X	X
IPI00218474	ENO3	BETA-ENOLASE.	X	X	X
IPI00218487	GJA1	GAP JUNCTION ALPHA-1 PROTEIN.	X	X	X
IPI00218539	COL11A1	ISOFORM B OF COLLAGEN ALPHA-1(XI) CHAIN PRECURSOR.	X	X	X
IPI00218570	PGAM2	PHOSPHOGLYCERATE MUTASE 2.	X	X	X
IPI00218725	LAMA2	LAMININ ALPHA 2 SUBUNIT ISOFORM B PRECURSOR.	X	X	X
IPI00218730	PDE6A	ROD CGMP-SPECIFIC 3',5'-CYCLIC PHOSPHODIESTERASE SUBUNIT ALPHA.	X	X	X
IPI00218732	PON1	SERUM PARAOXONASE/ARYLESTERASE 1.	X	X	X
IPI00218733	SOD1	SUPEROXIDE DISMUTASE.	X	X	X
IPI00218795	SELL	L-SELECTIN PRECURSOR.	X	X	X
IPI00218834	FCGR3A	LOW AFFINITY IMMUNOGLOBULIN GAMMA FC REGION RECEPTOR III-A PRECURSOR.	X	X	X
IPI00218875	SPP1	ISOFORM C OF OSTEOPONTIN PRECURSOR.	X	X	X
IPI00219025	GLRX	GLUTAREDOXIN-1.	X	X	X
IPI00219029	GOT1	ASPARTATE AMINOTRANSFERASE, CYTOPLASMIC.	X	X	X
IPI00219077	LTA4H	ISOFORM 1 OF LEUKOTRIENE A-4 HYDROLASE.	X	X	X
IPI00219129	NQO2	RIBOSYLDIHYDRONICOTINAMIDE DEHYDROGENASE.	X	X	X
IPI00219131	ICOSLG	ISOFORM 1 OF ICOS LIGAND PRECURSOR.	X	X	X
IPI00219217	LDHB	L-LACTATE DEHYDROGENASE B CHAIN.	X	X	X
IPI00219219	LGALS1	GALECTIN-1.	X	X	X
IPI00219301	MARCKS	MYRISTOYLATED ALANINE-RICH C-KINASE SUBSTRATE.	X	X	X
IPI00219365	MSN	MOESIN.	X	X	X
IPI00219420	SMC3	STRUCTURAL MAINTENANCE OF CHROMOSOMES PROTEIN 3.	X	X	X

Table S1. Proteins Identified or Not in MS Patients Compared to Normals and Other Neurologic Disease (OND)					
**IPI**	**Gene**	**Protein Name**	**MS**	**Normal**	**OND**
IPI00219425	PVR	ISOFORM BETA OF POLIOVIRUS RECEPTOR PRECURSOR.	X	X	X
IPI00219446	PEBP1	PHOSPHATIDYLETHANOLAMINE-BINDING PROTEIN 1.	X	X	X
IPI00219465	TCN2	TRANSCOBALAMIN-2 PRECURSOR.	X	X	X
IPI00219468	PFN2	ISOFORM IIA OF PROFILIN-2.	X	X	X
IPI00219757	GSTP1	GLUTATHIONE S-TRANSFERASE P.	X	X	X
IPI00219798	ROBO1	ISOFORM 1 OF ROUNDABOUT HOMOLOG 1 PRECURSOR.	X	X	X
IPI00219806	S100A7	PROTEIN S100-A7.	X	X	X
IPI00219930	CRABP1	CELLULAR RETINOIC ACID-BINDING PROTEIN 1.	X	X	X
IPI00220301	PRDX6	PEROXIREDOXIN-6.	X	X	X
IPI00220327	KRT1	KERATIN, TYPE II CYTOSKELETAL 1.	X	X	X
IPI00220342	DDAH1	N(G),N(G)-DIMETHYLARGININE DIMETHYLAMINOHYDROLASE 1.	X	X	X
IPI00220362	HSPE1	10 KDA HEAT SHOCK PROTEIN, MITOCHONDRIAL.	X	X	X
IPI00220562	NPTX1	NEURONAL PENTRAXIN-1 PRECURSOR.	X	X	X
IPI00220642	YWHAG	14-3-3 PROTEIN GAMMA.	X	X	X
IPI00220644	PKM2	ISOFORM M1 OF PYRUVATE KINASE ISOZYMES M1/M2.	X	X	X
IPI00220706	HBG1	HEMOGLOBIN SUBUNIT GAMMA-1.	X	X	X
IPI00220739	PGRMC1	MEMBRANE-ASSOCIATED PROGESTERONE RECEPTOR COMPONENT 1.	X	X	X
IPI00220766	GLO1	LACTOYLGLUTATHIONE LYASE.	X	X	X
IPI00220791	AMPH	AMPHIPHYSIN I VARIANT CT2.	X	X	X
IPI00220827	TMSB10	THYMOSIN BETA-10.	X	X	X
IPI00221178	TPD52L2	ISOFORM 2 OF TUMOR PROTEIN D54.	X	X	X
IPI00221224	ANPEP	AMINOPEPTIDASE N.	X	X	X
IPI00221255	MYLK	ISOFORM 2 OF MYOSIN LIGHT CHAIN KINASE, SMOOTH MUSCLE.	X	X	X
IPI00241562	RELN	REELIN ISOFORM A.	X	X	X
IPI00242956	FCGBP	IGGFC-BINDING PROTEIN PRECURSOR.	X	X	X
IPI00246058	PDCD6IP	PDCD6IP PROTEIN.	X	X	X
IPI00247243		31 KDA PROTEIN.	X	X	X
IPI00250724	C18orf51	PROTEIN KINASE-LIKE DOMAIN CONTAINING PROTEIN.	X	X	X
IPI00257882	PEPD	XAA-PRO DIPEPTIDASE.	X	X	X
IPI00259102	EPDR1	MAMMALIAN EPENDYMIN-RELATED PROTEIN 1 PRECURSOR.	X	X	X
IPI00289058	LYNX1	LY-6/NEUROTOXIN-LIKE PROTEIN 1 PRECURSOR.	X	X	X
IPI00289083	CACHD1	ISOFORM 1 OF VWFA AND CACHE DOMAIN-CONTAINING PROTEIN 1 PRECURSOR.	X	X	X
IPI00289204	RTN4R	RETICULON-4 RECEPTOR PRECURSOR.	X	X	X
IPI00289501	VGF	NEUROSECRETORY PROTEIN VGF PRECURSOR.	X	X	X

Table S1. Proteins Identified or Not in MS Patients Compared to Normals and Other Neurologic Disease (OND)					
**IPI**	**Gene**	**Protein Name**	**MS**	**Normal**	**OND**
IPI00289819	IGF2R	CATION-INDEPENDENT MANNOSE-6-PHOSPHATE RECEPTOR PRECURSOR.	X	X	X
IPI00289876	STX7	ISOFORM 1 OF SYNTAXIN-7.	X	X	X
IPI00289924	ST8SIA5	ALPHA-2,8-SIALYLTRANSFERASE 8E.	X	X	X
IPI00289926	LILRB4	LEUKOCYTE IMMUNOGLOBULIN-LIKE RECEPTOR, SUBFAMILY B, MEMBER 4 ISOFORM 2.	X	X	X
IPI00290078	KRT4	KERATIN 4.	X	X	X
IPI00290085	CDH2	CADHERIN-2 PRECURSOR.	X	X	X
IPI00290283	MASP1	MANNAN-BINDING LECTIN SERINE PROTEASE 1 ISOFORM 2 PRECURSOR.	X	X	X
IPI00290315	CHGA	CHROMOGRANIN-A PRECURSOR.	X	X	X
IPI00290328	PTPRJ	RECEPTOR-TYPE TYROSINE-PROTEIN PHOSPHATASE ETA PRECURSOR.	X	X	X
IPI00290358	LOC283951	PUTATIVE UNCHARACTERIZED PROTEIN GS103.	X	X	X
IPI00290744	C5orf40	FIBRONECTIN TYPE-III DOMAIN-CONTAINING PROTEIN C5ORF40.	X	X	X
IPI00290826	TMEM157	TRANSMEMBRANE PROTEIN 157 PRECURSOR.	X	X	X
IPI00290856	LYVE1	LYMPHATIC VESSEL ENDOTHELIAL HYALURONIC ACID RECEPTOR 1 PRECURSOR.	X	X	X
IPI00291005	MDH1	MALATE DEHYDROGENASE, CYTOPLASMIC.	X	X	X
IPI00291006	MDH2	MALATE DEHYDROGENASE, MITOCHONDRIAL PRECURSOR.	X	X	X
IPI00291136	COL6A1	COLLAGEN ALPHA-1(VI) CHAIN PRECURSOR.	X	X	X
IPI00291175	VCL	ISOFORM 1 OF VINCULIN.	X	X	X
IPI00291262	CLU	CLUSTERIN PRECURSOR.	X	X	X
IPI00291807	CPAMD8	C3 AND PZP-LIKE, ALPHA-2-MACROGLOBULIN DOMAIN CONTAINING 8.	X	X	X
IPI00291866	SERPING1	PLASMA PROTEASE C1 INHIBITOR PRECURSOR.	X	X	X
IPI00291987	IGFBPL1	INSULIN-LIKE GROWTH FACTOR-BINDING PROTEIN-LIKE 1 PRECURSOR.	X	X	X
IPI00292071	SCG3	SECRETOGRANIN-3 PRECURSOR.	X	X	X
IPI00292150	LTBP2	LATENT-TRANSFORMING GROWTH FACTOR BETA-BINDING PROTEIN 2 PRECURSOR.	X	X	X
IPI00292218	MST1	HEPATOCYTE GROWTH FACTOR-LIKE PROTEIN PRECURSOR.	X	X	X
IPI00292300	CNTNAP5	CONTACTIN ASSOCIATED PROTEIN-LIKE 5.	X	X	X
IPI00292304	C9orf4	UNCHARACTERIZED PROTEIN C9ORF4.	X	X	X
IPI00292530	ITIH1	INTER-ALPHA-TRYPSIN INHIBITOR HEAVY CHAIN H1 PRECURSOR.	X	X	X
IPI00292550	GALNT13	ISOFORM 1 OF POLYPEPTIDE N-ACETYLGALACTOSAMINYLTRANSFERASE 13.	X	X	X
IPI00292732	FMOD	FIBROMODULIN PRECURSOR.	X	X	X
IPI00292791	CNTN3	CONTACTIN-3 PRECURSOR.	X	X	X
IPI00292946	SERPINA7	THYROXINE-BINDING GLOBULIN PRECURSOR.	X	X	X
IPI00292950	SERPIND1	SERPIN PEPTIDASE INHIBITOR, CLADE D (HEPARIN COFACTOR), MEMBER 1.	X	X	X
IPI00293088	GAA	LYSOSOMAL ALPHA-GLUCOSIDASE PRECURSOR.	X	X	X

Table S1. Proteins Identified or Not in MS Patients Compared to Normals and Other Neurologic Disease (OND)					
**IPI**	**Gene**	**Protein Name**	**MS**	**Normal**	**OND**
IPI00293276	MIF	MACROPHAGE MIGRATION INHIBITORY FACTOR.	X	X	X
IPI00293303	LGMN	LEGUMAIN PRECURSOR.	X	X	X
IPI00293464	DDB1	DNA DAMAGE-BINDING PROTEIN 1.	X	X	X
IPI00293723	NXPH4	NEUREXOPHILIN-4 PRECURSOR.	X	X	X
IPI00293748	MINPP1	ISOFORM 1 OF MULTIPLE INOSITOL POLYPHOSPHATE PHOSPHATASE 1 PRECURSOR.	X	X	X
IPI00293757	UNC5C	ISOFORM 1 OF NETRIN RECEPTOR UNC5C PRECURSOR.	X	X	X
IPI00293836	CADM2	ISOFORM 3 OF CELL ADHESION MOLECULE 2 PRECURSOR.	X	X	X
IPI00293849	PTPRM	RECEPTOR-TYPE TYROSINE-PROTEIN PHOSPHATASE MU PRECURSOR.	X	X	X
IPI00293925	FCN3	ISOFORM 1 OF FICOLIN-3 PRECURSOR.	X	X	X
IPI00293971	ATP1B2	SODIUM/POTASSIUM-TRANSPORTING ATPASE SUBUNIT BETA-2.	X	X	X
IPI00294004	PROS1	VITAMIN K-DEPENDENT PROTEIN S PRECURSOR.	X	X	X
IPI00294395	C8B	COMPLEMENT COMPONENT C8 BETA CHAIN PRECURSOR.	X	X	X
IPI00294615	FBLN5	FIBULIN-5 PRECURSOR.	X	X	X
IPI00295386	CBR1	CARBONYL REDUCTASE [NADPH] 1.	X	X	X
IPI00295399	CDH10	CADHERIN-10 PRECURSOR.	X	X	X
IPI00295414	COL15A1	COLLAGEN ALPHA-1(XV) CHAIN PRECURSOR.	X	X	X
IPI00295542	NUCB1	NUCLEOBINDIN-1 PRECURSOR.	X	X	X
IPI00295618	PECAM1	ISOFORM LONG OF PLATELET ENDOTHELIAL CELL ADHESION MOLECULE PRECURSOR.	X	X	X
IPI00295741	CTSB	CATHEPSIN B PRECURSOR.	X	X	X
IPI00295767	OLFM2	NOELIN-2 PRECURSOR.	X	X	X
IPI00295832	OMG	OLIGODENDROCYTE-MYELIN GLYCOPROTEIN PRECURSOR.	X	X	X
IPI00296058	EFEMP2	EGF-CONTAINING FIBULIN-LIKE EXTRACELLULAR MATRIX PROTEIN 2 PRECURSOR.	X	X	X
IPI00296141	DPP7	DIPEPTIDYL-PEPTIDASE 2 PRECURSOR.	X	X	X
IPI00296165	ACYP1; C17orf13; C1R	COMPLEMENT C1R SUBCOMPONENT PRECURSOR.	X	X	X
IPI00296176	F9	COAGULATION FACTOR IX PRECURSOR.	X	X	X
IPI00296441	ADA	ADENOSINE DEAMINASE.	X	X	X
IPI00296534	FBLN1	ISOFORM D OF FIBULIN-1 PRECURSOR.	X	X	X
IPI00296537	FBLN1	ISOFORM C OF FIBULIN-1 PRECURSOR.	X	X	X
IPI00296608	C7	COMPLEMENT COMPONENT C7 PRECURSOR.	X	X	X
IPI00296777	SPARCL1	SPARC-LIKE PROTEIN 1 PRECURSOR.	X	X	X
IPI00296922	LAMB2	LAMININ SUBUNIT BETA-2 PRECURSOR.	X	X	X
IPI00296992	AXL	AXL RECEPTOR TYROSINE KINASE ISOFORM 1.	X	X	X

**Table S1. Proteins Identified or Not in MS Patients Compared to Normals and Other Neurologic Disease (OND)**					
**IPI**	**Gene**	**Protein Name**	**MS**	**Normal**	**OND**
IPI00297040	SPINK6	SERINE PROTEASE INHIBITOR KAZAL-TYPE 6 PRECURSOR.	X	X	X
IPI00297124	IL6ST	ISOFORM 1 OF INTERLEUKIN-6 RECEPTOR SUBUNIT BETA PRECURSOR.	X	X	X
IPI00297180	CDH9	CADHERIN-9 PRECURSOR.	X	X	X
IPI00297181	CDH7	CADHERIN-7 PRECURSOR.	X	X	X
IPI00297188	BAI2	BRAIN-SPECIFIC ANGIOGENESIS INHIBITOR 2 PRECURSOR.	X	X	X
IPI00297208	KIAA1276	SIMILAR TO MYOSIN-10.	X	X	X
IPI00297224	SUSD5	SUSHI DOMAIN-CONTAINING PROTEIN 5.	X	X	X
IPI00297251	MGRN1	ISOFORM 2 OF PROBABLE E3 UBIQUITIN-PROTEIN LIGASE MGRN1.	X	X	X
IPI00297252	SULF2	ISOFORM 1 OF EXTRACELLULAR SULFATASE SULF-2 PRECURSOR.	X	X	X
IPI00297263	HEG1	ISOFORM 1 OF PROTEIN HEG HOMOLOG 1 PRECURSOR.	X	X	X
IPI00297284	IGFBP2	INSULIN-LIKE GROWTH FACTOR-BINDING PROTEIN 2 PRECURSOR.	X	X	X
IPI00297444	CD177	ISOFORM 1 OF CD177 ANTIGEN PRECURSOR.	X	X	X
IPI00297487	CTSH	CATHEPSIN H PRECURSOR.	X	X	X
IPI00297646	COL1A1	COLLAGEN ALPHA-1(I) CHAIN PRECURSOR.	X	X	X
IPI00297655	NOTCH2	NEUROGENIC LOCUS NOTCH HOMOLOG PROTEIN 2 PRECURSOR.	X	X	X
IPI00297714	SNCG	GAMMA-SYNUCLEIN.	X	X	X
IPI00298237	TPP1	ISOFORM 1 OF TRIPEPTIDYL-PEPTIDASE 1 PRECURSOR.	X	X	X
IPI00298281	LAMC1	LAMININ SUBUNIT GAMMA-1 PRECURSOR.	X	X	X
IPI00298388	PIK3IP1	ISOFORM 1 OF PHOSPHOINOSITIDE-3-KINASE-INTERACTING PROTEIN 1 PRECURSOR.	X	X	X
IPI00298497	FGB	FIBRINOGEN BETA CHAIN PRECURSOR.	X	X	X
IPI00298547	PARK7	PROTEIN DJ-1.	X	X	X
IPI00298702	SLC39A6	SOLUTE CARRIER FAMILY 39 (ZINC TRANSPORTER), MEMBER 6 ISOFORM 1.	X	X	X
IPI00298793	MANBA	BETA-MANNOSIDASE PRECURSOR.	X	X	X
IPI00298828	APOH	BETA-2-GLYCOPROTEIN 1 PRECURSOR.	X	X	X
IPI00298971	VTN	VITRONECTIN PRECURSOR.	X	X	X
IPI00299024	BASP1	BRAIN ACID SOLUBLE PROTEIN 1.	X	X	X
IPI00299059	CHL1	ISOFORM 2 OF NEURAL CELL ADHESION MOLECULE L1-LIKE PROTEIN PRECURSOR.	X	X	X
IPI00299083	JAM2	JUNCTIONAL ADHESION MOLECULE B PRECURSOR.	X	X	X
IPI00299086	SDCBP	SYNTENIN-1.	X	X	X
IPI00299150	CTSS	CATHEPSIN S PRECURSOR.	X	X	X
IPI00299299	STCH	STRESS 70 PROTEIN CHAPERONE MICROSOME-ASSOCIATED 60 KDA PROTEIN PRECURSOR.	X	X	X
IPI00299485	CD93	COMPLEMENT COMPONENT C1Q RECEPTOR PRECURSOR.	X	X	X

Table S1. Proteins Identified or Not in MS Patients Compared to Normals and Other Neurologic Disease (OND)					
IPI	Gene	Protein Name	MS	Normal	OND
IPI00299503	GPLD1	ISOFORM 1 OF PHOSPHATIDYLINOSITOL-GLYCAN-SPECIFIC PHOSPHOLIPASE D PRECURSOR.	X	X	X
IPI00299547	LCN2	NEUTROPHIL GELATINASE-ASSOCIATED LIPOCALIN PRECURSOR.	X	X	X
IPI00299652	ADAM11	ISOFORM LONG OF ADAM 11 PRECURSOR.	X	X	X
IPI00299699	NPDC1	NEURAL PROLIFERATION DIFFERENTIATION AND CONTROL PROTEIN 1 PRECURSOR.	X	X	X
IPI00299724	SIRPB1	ISOFORM 1 OF SIGNAL REGULATORY PROTEIN BETA-1 PRECURSOR.	X	X	X
IPI00299738	PCOLCE	PROCOLLAGEN C-ENDOPEPTIDASE ENHANCER 1 PRECURSOR.	X	X	X
IPI00300020	SLC1A2	EXCITATORY AMINO ACID TRANSPORTER 2.	X	X	X
IPI00300241	LRRC4B	LEUCINE-RICH REPEAT-CONTAINING PROTEIN 4B PRECURSOR.	X	X	X
IPI00300407	SDC2	SYNDECAN-2 PRECURSOR.	X	X	X
IPI00300725	KRT6A	KERATIN, TYPE II CYTOSKELETAL 6A.	X	X	X
IPI00300838	CHST8	CARBOHYDRATE SULFOTRANSFERASE 8.	X	X	X
IPI00301143	PI16	ISOFORM 1 OF PEPTIDASE INHIBITOR 16 PRECURSOR.	X	X	X
IPI00301180	SLC12A5	ISOFORM 2 OF SOLUTE CARRIER FAMILY 12 MEMBER 5.	X	X	X
IPI00301255	IGSF21	IMMUNOGLOBULIN SUPERFAMILY MEMBER 21 PRECURSOR.	X	X	X
IPI00301294	FAM134A	PROTEIN FAM134A.	X	X	X
IPI00301364	SKP1	ISOFORM 1 OF S-PHASE KINASE-ASSOCIATED PROTEIN 1A.	X	X	X
IPI00301395	CPVL	PROBABLE SERINE CARBOXYPEPTIDASE CPVL PRECURSOR.	X	X	X
IPI00301459	LYPLA3	1-O-ACYLCERAMIDE SYNTHASE PRECURSOR.	X	X	X
IPI00301579	NPC2	EPIDIDYMAL SECRETORY PROTEIN E1 PRECURSOR.	X	X	X
IPI00301812	SMOC1	ISOFORM 1 OF SPARC-RELATED MODULAR CALCIUM-BINDING PROTEIN 1 PRECURSOR.	X	X	X
IPI00301865	TMEM132A	ISOFORM 1 OF TRANSMEMBRANE PROTEIN 132A PRECURSOR.	X	X	X
IPI00301961	PCSK1	NEUROENDOCRINE CONVERTASE 1 PRECURSOR.	X	X	X
IPI00302181	CACNA2D3	ISOFORM 1 OF VOLTAGE-DEPENDENT CALCIUM CHANNEL SUBUNIT ALPHA-2/DELTA-3 PRECURSOR.	X	X	X
IPI00302592	FLNA	FILAMIN A, ALPHA ISOFORM 1.	X	X	X
IPI00302641	FAT2	PROTOCADHERIN FAT 2 PRECURSOR.	X	X	X
IPI00302840	ATP1A3	SODIUM/POTASSIUM-TRANSPORTING ATPASE SUBUNIT ALPHA-3.	X	X	X
IPI00303071	CECR1	CAT EYE SYNDROME CRITICAL REGION PROTEIN 1 PRECURSOR.	X	X	X
IPI00303161	ESAM	ENDOTHELIAL CELL-SELECTIVE ADHESION MOLECULE PRECURSOR.	X	X	X
IPI00303318	FAM49B	PROTEIN FAM49B.	X	X	X
IPI00303894	FAM3A	PROTEIN FAM3A PRECURSOR.	X	X	X
IPI00304273	APOA4	APOLIPOPROTEIN A-IV PRECURSOR.	X	X	X

**Table S1. Proteins Identified or Not in MS Patients Compared to Normals and Other Neurologic Disease (OND)**					
**IPI**	**Gene**	**Protein Name**	**MS**	**Normal**	**OND**
IPI00304840	COL6A2	ISOFORM 2C2 OF COLLAGEN ALPHA-2(VI) CHAIN PRECURSOR.	X	X	X
IPI00304865	TGFBR3	TRANSFORMING GROWTH FACTOR, BETA RECEPTOR III.	X	X	X
IPI00304962	COL1A2	COLLAGEN ALPHA-2(I) CHAIN PRECURSOR.	X	X	X
IPI00305380	IGFBP4	INSULIN-LIKE GROWTH FACTOR-BINDING PROTEIN 4 PRECURSOR.	X	X	X
IPI00305461	ITIH2	INTER-ALPHA-TRYPSIN INHIBITOR HEAVY CHAIN H2 PRECURSOR.	X	X	X
IPI00305975	SPON2	SPONDIN-2 PRECURSOR.	X	X	X
IPI00306322	COL4A2	COLLAGEN ALPHA-2(IV) CHAIN PRECURSOR.	X	X	X
IPI00306339	SPP1	SECRETED PHOSPHOPROTEIN 1 ISOFORM B.	X	X	X
IPI00306710	CHRD	ISOFORM 1 OF CHORDIN PRECURSOR.	X	X	X
IPI00306844	CRHBP	CORTICOTROPIN-RELEASING FACTOR-BINDING PROTEIN PRECURSOR.	X	X	X
IPI00306853	CHST3	CARBOHYDRATE SULFOTRANSFERASE 3.	X	X	X
IPI00307276	ADAMTS4	ADAMTS-4 PRECURSOR.	X	X	X
IPI00307591	ZNF609	ZINC FINGER PROTEIN 609.	X	X	X
IPI00307592	ABCA2	ATP-BINDING CASSETTE, SUB-FAMILY A, MEMBER 2 ISOFORM A.	X	X	X
IPI00328113	FBN1	FIBRILLIN-1 PRECURSOR.	X	X	X
IPI00328243	PLD3	PHOSPHOLIPASE D3.	X	X	X
IPI00328257	AP1B1	ISOFORM A OF AP-1 COMPLEX SUBUNIT BETA-1.	X	X	X
IPI00328391	GALNT7	N-ACETYLGALACTOSAMINYLTRANSFERASE 7.	X	X	X
IPI00328431	UNC5B	ISOFORM 1 OF NETRIN RECEPTOR UNC5B PRECURSOR.	X	X	X
IPI00328488	MAN2B2	ISOFORM 1 OF EPIDIDYMIS-SPECIFIC ALPHA-MANNOSIDASE PRECURSOR.	X	X	X
IPI00328520	PRRT2	ISOFORM 2 OF PROLINE-RICH TRANSMEMBRANE PROTEIN 2.	X	X	X
IPI00328550	THBS4	THROMBOSPONDIN-4 PRECURSOR.	X	X	X
IPI00328609	SERPINA4	KALLISTATIN PRECURSOR.	X	X	X
IPI00328703	OAF	OUT AT FIRST PROTEIN HOMOLOG PRECURSOR.	X	X	X
IPI00328745	RTN4RL1	RETICULON-4 RECEPTOR-LIKE 1 PRECURSOR.	X	X	X
IPI00328746	RTN4RL2	RETICULON-4 RECEPTOR-LIKE 2 PRECURSOR.	X	X	X
IPI00329332	STX12	SYNTAXIN-12.	X	X	X
IPI00329352	NOMO1; NOMO3	NODAL MODULATOR 1 PRECURSOR.	X	X	X
IPI00329482	LAMA4	ISOFORM 1 OF LAMININ SUBUNIT ALPHA-4 PRECURSOR.	X	X	X
IPI00329685	ARSA	PUTATIVE UNCHARACTERIZED PROTEIN DKFZP686G12235.	X	X	X
IPI00329688	YIPF3	PROTEIN YIPF3.	X	X	X
IPI00329775	CPB2	ISOFORM 1 OF CARBOXYPEPTIDASE B2 PRECURSOR.	X	X	X

Table S1. Proteins Identified or Not in MS Patients Compared to Normals and Other Neurologic Disease (OND)					
**IPI**	**Gene**	**Protein Name**	**MS**	**Normal**	**OND**
IPI00333140	DNER	DELTA AND NOTCH-LIKE EPIDERMAL GROWTH FACTOR-RELATED RECEPTOR PRECURSOR.	X	X	X
IPI00333776	NRCAM	ISOFORM 1 OF NEURONAL CELL ADHESION MOLECULE PRECURSOR.	X	X	X
IPI00334238	NPTXR	NEURONAL PENTRAXIN RECEPTOR.	X	X	X
IPI00334282	FAM3C	PROTEIN FAM3C PRECURSOR.	X	X	X
IPI00337351	MDGA2	MAM DOMAIN-CONTAINING GLYCOSYLPHOSPHATIDYLINOSITOL ANCHOR PROTEIN 2 PRECURSOR.	X	X	X
IPI00337548	CGREF1	CELL GROWTH REGULATOR WITH EF HAND DOMAIN PROTEIN 1.	X	X	X
IPI00374065	MIA3	SIMILAR TO MELANOMA INHIBITORY ACTIVITY 3 ISOFORM 1.	X	X	X
IPI00374563	AGRN	AGRIN PRECURSOR.	X	X	X
IPI00374590	CASC4	CANCER SUSCEPTIBILITY CANDIDATE 4 ISOFORM A.	X	X	X
IPI00374914	LOC401115	HYPOTHETICAL PROTEIN.	X	X	X
IPI00375364	CHIT1	ISOFORM 3 OF CHITOTRIOSIDASE-1 PRECURSOR.	X	X	X
IPI00376131	hCG_2040376	SIMILAR TO LEUCINE RICH REPEAT NEURONAL 6C.	X	X	X
IPI00376394	QSOX2	SULFHYDRYL OXIDASE 2 PRECURSOR.	X	X	X
IPI00376427	NCAM2	NEURAL CELL ADHESION MOLECULE 2 PRECURSOR.	X	X	X
IPI00376689	KIAA1199	ISOFORM 1 OF PROTEIN KIAA1199 PRECURSOR.	X	X	X
IPI00382420		IG LAMBDA CHAIN V-I REGION HA.	X	X	X
IPI00382442		IG LAMBDA CHAIN V-V REGION DEL.	X	X	X
IPI00382474		IG HEAVY CHAIN V-III REGION TRO.	X	X	X
IPI00382476		IG HEAVY CHAIN V-III REGION WEA.	X	X	X
IPI00382486		IG HEAVY CHAIN V-III REGION NIE.	X	X	X
IPI00382488		IG HEAVY CHAIN V-III REGION HIL.	X	X	X
IPI00382499		IG HEAVY CHAIN V-III REGION JON.	X	X	X
IPI00382682		PUTATIVE MATRIX CELL ADHESION MOLECULE-3.	X	X	X
IPI00383032	HAVCR2	ISOFORM 2 OF HEPATITIS A VIRUS CELLULAR RECEPTOR 2 PRECURSOR.	X	X	X
IPI00383603		ANTI-THYROGLOBULIN LIGHT CHAIN VARIABLE REGION (FRAGMENT).	X	X	X
IPI00383732		VH3 PROTEIN (FRAGMENT).	X	X	X
IPI00383953		VH4 HEAVY CHAIN VARIABLE REGION PRECURSOR (FRAGMENT).	X	X	X
IPI00384016	DLST	FULL-LENGTH CDNA 5-PRIME END OF CLONE CS0DJ009YL13 OF T CELLS (JURKAT CELL LINE) OF HOMO SAPIENS (FRAGMENT).	X	X	X
IPI00384051	PSME2	UNCHARACTERIZED PROTEIN PSME2.	X	X	X
IPI00384225	METRN	METEORIN PRECURSOR.	X	X	X

Table S1. Proteins Identified or Not in MS Patients Compared to Normals and Other Neurologic Disease (OND)					
IPI	Gene	Protein Name	MS	Normal	OND
IPI00384391		MYOSIN-REACTIVE IMMUNOGLOBULIN HEAVY CHAIN VARIABLE REGION (FRAGMENT).	X	X	X
IPI00384392		MYOSIN-REACTIVE IMMUNOGLOBULIN HEAVY CHAIN VARIABLE REGION (FRAGMENT).	X	X	X
IPI00384395		MYOSIN-REACTIVE IMMUNOGLOBULIN HEAVY CHAIN VARIABLE REGION.	X	X	X
IPI00384402		MYOSIN-REACTIVE IMMUNOGLOBULIN KAPPA CHAIN VARIABLE REGION (FRAGMENT).	X	X	X
IPI00384404		RHEUMATOID FACTOR RF-ET9 (FRAGMENT).	X	X	X
IPI00384444	KRT14	KERATIN, TYPE I CYTOSKELETAL 14.	X	X	X
IPI00384697	ALB	ISOFORM 2 OF SERUM ALBUMIN PRECURSOR.	X	X	X
IPI00384722	C19orf63	ISOFORM 2 OF UPF0510 PROTEIN C19ORF63 PRECURSOR.	X	X	X
IPI00385143		MICROFIBRILLAR PROTEIN 2 (FRAGMENT).	X	X	X
IPI00385252		IG KAPPA CHAIN V-III REGION GOL.	X	X	X
IPI00385253		IG KAPPA CHAIN V-III REGION CLL PRECURSOR.	X	X	X
IPI00385980	ROBO2	ROBO2 ISOFORM A.	X	X	X
IPI00385985		IG LAMBDA CHAIN V-III REGION LOI.	X	X	X
IPI00386131		IG KAPPA CHAIN V-III REGION IARC/BL41 PRECURSOR.	X	X	X
IPI00386133		IG KAPPA CHAIN V-IV REGION B17 PRECURSOR.	X	X	X
IPI00386879	IGHA1; IGHV3OR16-13	CDNA FLJ14473 FIS, CLONE MAMMA1001080, HIGHLY SIMILAR TO HOMO SAPIENS SNC73 PROTEIN (SNC73) MRNA.	X	X	X
IPI00387024		IG KAPPA CHAIN V-I REGION CAR.	X	X	X
IPI00387097		IG KAPPA CHAIN V-I REGION LAY.	X	X	X
IPI00387105		IG KAPPA CHAIN V-I REGION MEV.	X	X	X
IPI00387106		IG KAPPA CHAIN V-I REGION NI.	X	X	X
IPI00387110		IG KAPPA CHAIN V-II REGION MIL.	X	X	X
IPI00387113		IG KAPPA CHAIN V-III REGION B6.	X	X	X
IPI00387115		IG KAPPA CHAIN V-III REGION SIE.	X	X	X
IPI00387116		IG KAPPA CHAIN V-III REGION NG9 PRECURSOR (FRAGMENT).	X	X	X
IPI00387117	IGKV3D-20	IG KAPPA CHAIN V-III REGION TI.	X	X	X
IPI00387120		IG KAPPA CHAIN V-IV REGION LEN.	X	X	X
IPI00387168	PCSK9	ISOFORM 1 OF PROPROTEIN CONVERTASE SUBTILISIN/KEXIN TYPE 9 PRECURSOR.	X	X	X
IPI00394655	NFASC	ISOFORM 4 OF NEUROFASCIN PRECURSOR.	X	X	X
IPI00394870	VWC2	BRORIN PRECURSOR.	X	X	X
IPI00395488	VASN	VASORIN PRECURSOR.	X	X	X

Table S1. Proteins Identified or Not in MS Patients Compared to Normals and Other Neurologic Disease (OND)					
**IPI**	**Gene**	**Protein Name**	**MS**	**Normal**	**OND**
IPI00396423	CLSTN3	ALCADEIN BETA.	X	X	X
IPI00396930		UNCHARACTERIZED PROTEIN ENSP00000353216 (FRAGMENT).	X	X	X
IPI00397645	MXRA7	ISOFORM 2 OF MATRIX-REMODELING-ASSOCIATED PROTEIN 7.	X	X	X
IPI00397949	GPR56	G PROTEIN-COUPLED RECEPTOR 56 ISOFORM B.	X	X	X
IPI00400986	KIAA1731	HYPOTHETICAL PROTEIN LOC85459.	X	X	X
IPI00401264	TXNDC4	THIOREDOXIN DOMAIN-CONTAINING PROTEIN 4 PRECURSOR.	X	X	X
IPI00401283	MEGF9	MULTIPLE EPIDERMAL GROWTH FACTOR-LIKE DOMAINS 9 PRECURSOR.	X	X	X
IPI00402157	CBLN3	CEREBELLIN-3 PRECURSOR.	X	X	X
IPI00410079	FAM82C	ISOFORM 1 OF PROTEIN FAM82C.	X	X	X
IPI00410122	PLXDC1	ISOFORM 1 OF PLEXIN DOMAIN-CONTAINING PROTEIN 1 PRECURSOR.	X	X	X
IPI00410487	TWSG1	ISOFORM 1 OF TWISTED GASTRULATION PROTEIN HOMOLOG 1 PRECURSOR.	X	X	X
IPI00410714	HBA1; HBA2	HEMOGLOBIN SUBUNIT ALPHA.	X	X	X
IPI00411680	PCMT1	ISOFORM 1 OF PROTEIN-L-ISOASPARTATE(D-ASPARTATE) O-METHYLTRANSFERASE.	X	X	X
IPI00411706	ESD	S-FORMYLGLUTATHIONE HYDROLASE.	X	X	X
IPI00412264	PTN	PLEIOTROPHIN PRECURSOR.	X	X	X
IPI00412541	GPR158	PROBABLE G-PROTEIN COUPLED RECEPTOR 158 PRECURSOR.	X	X	X
IPI00412987	GMFB	GMFB PROTEIN.	X	X	X
IPI00412988	NTNG1	ISOFORM 1 OF NETRIN-G1 PRECURSOR.	X	X	X
IPI00413344	CFL2	COFILIN-2.	X	X	X
IPI00413451	SERPINB6	PUTATIVE UNCHARACTERIZED PROTEIN DKFZP686I04222.	X	X	X
IPI00413641	AKR1B1	ALDOSE REDUCTASE.	X	X	X
IPI00413778	FKBP1A	PEPTIDYL-PROLYL CIS-TRANS ISOMERASE.	X	X	X
IPI00414294	LOC729956	HYPOTHETICAL PROTEIN.	X	X	X
IPI00414467	COLEC12	COLLECTIN SUB-FAMILY MEMBER 12.	X	X	X
IPI00414717	GLG1	GOLGI APPARATUS PROTEIN 1.	X	X	X
IPI00414896	RNASET2	ISOFORM 1 OF RIBONUCLEASE T2 PRECURSOR.	X	X	X
IPI00414909	NAGA	ALPHA-N-ACETYLGALACTOSAMINIDASE PRECURSOR.	X	X	X
IPI00414984	SGCE	SARCOGLYCAN, EPSILON ISOFORM 1.	X	X	X
IPI00415032	NRCAM	ISOFORM 4 OF NEURONAL CELL ADHESION MOLECULE PRECURSOR.	X	X	X
IPI00418163	C4B	C4B1.	X	X	X
IPI00418262	ALDOC	FRUCTOSE-BISPHOSPHATE ALDOLASE C.	X	X	X
IPI00418471	VIM	VIMENTIN.	X	X	X
IPI00418531	GLDN	ISOFORM 1 OF GLIOMEDIN.	X	X	X

Table S1. Proteins Identified or Not in MS Patients Compared to Normals and Other Neurologic Disease (OND)					
**IPI**	**Gene**	**Protein Name**	**MS**	**Normal**	**OND**
IPI00419585	LOC654188; PPIA; PPIAL3	PEPTIDYL-PROLYL CIS-TRANS ISOMERASE A.	X	X	X
IPI00419595	PODXL2	ISOFORM 1 OF PODOCALYXIN-LIKE PROTEIN 2 PRECURSOR.	X	X	X
IPI00419720		DERMOKINE GAMMA-1.	X	X	X
IPI00419724	SEMA4B	SEMAPHORIN 4B PRECURSOR.	X	X	X
IPI00419966	ABI3BP	ISOFORM 2 OF TARGET OF NESH-SH3 PRECURSOR.	X	X	X
IPI00423463	IGHG1	PUTATIVE UNCHARACTERIZED PROTEIN DKFZP686O01196.	X	X	X
IPI00424119	FZD3	FRIZZLED-3 PRECURSOR.	X	X	X
IPI00426051		PUTATIVE UNCHARACTERIZED PROTEIN DKFZP686C15213.	X	X	X
IPI00426066	DEPDC5	PLECKSTRIN/ G-PROTEIN, INTERACTING REGION DOMAIN CONTAINING PROTEIN.	X	X	X
IPI00428511	NRXN1	NEUREXIN-1-BETA PRECURSOR.	X	X	X
IPI00428967	TICAM2; TMED7	TOLL-LIKE RECEPTOR ADAPTER MOLECULE 2.	X	X	X
IPI00431738	IL1RAPL1	X-LINKED INTERLEUKIN-1 RECEPTOR ACCESSORY PROTEIN-LIKE 1 PRECURSOR.	X	X	X
IPI00432525	SIGLEC14	SIALIC ACID-BINDING IG-LIKE LECTIN 14 PRECURSOR.	X	X	X
IPI00432723	XYLT2	ISOFORM 1 OF XYLOSYLTRANSFERASE 2.	X	X	X
IPI00439446	MAN1A1	MAN1A1 PROTEIN.	X	X	X
IPI00440577	IGKV2-24	IGKV2-24 PROTEIN.	X	X	X
IPI00441498	FOLR1	FOLATE RECEPTOR ALPHA PRECURSOR.	X	X	X
IPI00442911	IGHV4-31	CDNA FLJ26266 FIS, CLONE DMC05613.	X	X	X
IPI00443799	MGC15523	HYPOTHETICAL PROTEIN LOC124565 ISOFORM A.	X	X	X
IPI00443909	CNPY2	ISOFORM 1 OF PROTEIN CANOPY HOMOLOG 2 PRECURSOR.	X	X	X
IPI00445716	GFRA3	ISOFORM 1 OF GDNF FAMILY RECEPTOR ALPHA-3 PRECURSOR.	X	X	X
IPI00448925	IGHG1	IGHG1 PROTEIN.	X	X	X
IPI00451625	CRTAC1	ISOFORM 2 OF CARTILAGE ACIDIC PROTEIN 1 PRECURSOR.	X	X	X
IPI00455667	LOC402665	HYPOTHETICAL PROTEIN LOC402665.	X	X	X
IPI00455739	LFNG	ISOFORM 1 OF BETA-1,3-N-ACETYLGLUCOSAMINYLTRANSFERASE LUNATIC FRINGE.	X	X	X
IPI00456623	BCAN	ISOFORM 1 OF BREVICAN CORE PROTEIN PRECURSOR.	X	X	X
IPI00456736	RGMB	ISOFORM 1 OF RGM DOMAIN FAMILY MEMBER B PRECURSOR.	X	X	X
IPI00456969	DYNC1H1	DYNEIN HEAVY CHAIN, CYTOSOLIC.	X	X	X
IPI00465028	TPI1	ISOFORM 1 OF TRIOSEPHOSPHATE ISOMERASE.	X	X	X
IPI00465184	GDA	GUANINE DEAMINASE.	X	X	X
IPI00465248	ENO1	ISOFORM ALPHA-ENOLASE OF ALPHA-ENOLASE.	X	X	X
IPI00465255	PRAP1	ISOFORM 1 OF PROLINE-RICH ACIDIC PROTEIN 1 PRECURSOR.	X	X	X

Table S1. Proteins Identified or Not in MS Patients Compared to Normals and Other Neurologic Disease (OND)					
**IPI**	**Gene**	**Protein Name**	**MS**	**Normal**	**OND**
IPI00465315	CYCS	CYTOCHROME C.	X	X	X
IPI00465322	BOC	UNCHARACTERIZED PROTEIN BOC.	X	X	X
IPI00465325	LINGO1	LEUCINE-RICH REPEAT NEURONAL 6A.	X	X	X
IPI00465436	CAT	CATALASE.	X	X	X
IPI00465439	ALDOA	FRUCTOSE-BISPHOSPHATE ALDOLASE A.	X	X	X
IPI00470535	CACNA2D1	DIHYDROPYRIDINE RECEPTOR ALPHA 2 SUBUNIT.	X	X	X
IPI00470607	FAM20C	FAMILY WITH SEQUENCE SIMILARITY 20, MEMBER C.	X	X	X
IPI00470625	NRN1	NEURITIN PRECURSOR.	X	X	X
IPI00470766	OLFML2B	ISOFORM 1 OF OLFACTOMEDIN-LIKE PROTEIN 2B PRECURSOR.	X	X	X
IPI00472345	IGHG3	IGHG3 PROTEIN.	X	X	X
IPI00472961	IGKC	IGKC PROTEIN.	X	X	X
IPI00473011	HBB; HBD	HEMOGLOBIN SUBUNIT DELTA.	X	X	X
IPI00477597	HPR	ISOFORM 1 OF HAPTOGLOBIN-RELATED PROTEIN PRECURSOR.	X	X	X
IPI00477611	COL5A1	184 KDA PROTEIN.	X	X	X
IPI00477714	IGLV8-61	V3-4 PROTEIN.	X	X	X
IPI00477747	FSTL4	ISOFORM 1 OF FOLLISTATIN-RELATED PROTEIN 4 PRECURSOR.	X	X	X
IPI00477804		IMMUNGLOBULIN HEAVY CHAIN VARIABLE REGION (FRAGMENT).	X	X	X
IPI00477868	LAMA5	LAMA5 PROTEIN.	X	X	X
IPI00477992	C1QB	COMPLEMENT COMPONENT 1, Q SUBCOMPONENT, B CHAIN PRECURSOR.	X	X	X
IPI00478003	A2M	ALPHA-2-MACROGLOBULIN PRECURSOR.	X	X	X
IPI00478483	LAMC3	172 KDA PROTEIN.	X	X	X
IPI00478890	SPOCK3	ISOFORM 1 OF TESTICAN-3 PRECURSOR.	X	X	X
IPI00478892	LRIG2	LEUCINE-RICH REPEATS AND IMMUNOGLOBULIN-LIKE DOMAINS PROTEIN 2 PRECURSOR.	X	X	X
IPI00478997	IGLV4-69	V5-6 PROTEIN.	X	X	X
IPI00479997	STMN1	STATHMIN.	X	X	X
IPI00549330	IGKV3D-15	MYOSIN-REACTIVE IMMUNOGLOBULIN LIGHT CHAIN VARIABLE REGION.	X	X	X
IPI00550115	SMPDL3B	ISOFORM 1 OF ACID SPHINGOMYELINASE-LIKE PHOSPHODIESTERASE 3B PRECURSOR.	X	X	X
IPI00550731		PUTATIVE UNCHARACTERIZED PROTEIN.	X	X	X
IPI00550991	SERPINA3	ALPHA-1-ANTICHYMOTRYPSIN PRECURSOR.	X	X	X
IPI00552874	IGLV2-11	V1-3 PROTEIN.	X	X	X
IPI00552905	PRRT3	ISOFORM 1 OF PROLINE-RICH TRANSMEMBRANE PROTEIN 3 PRECURSOR.	X	X	X
IPI00553138	VAMP2	VESICLE-ASSOCIATED MEMBRANE PROTEIN 2.	X	X	X

Table S1. Proteins Identified or Not in MS Patients Compared to Normals and Other Neurologic Disease (OND)					
**IPI**	**Gene**	**Protein Name**	**MS**	**Normal**	**OND**
IPI00554474	LOC284297	HYPOTHETICAL LOC284297.	X	X	X
IPI00554799	SPRN	SHADOW OF PRION PROTEIN.	X	X	X
IPI00555812	GC	VITAMIN D-BINDING PROTEIN PRECURSOR.	X	X	X
IPI00556287		PUTATIVE UNCHARACTERIZED PROTEIN.	X	X	X
IPI00607580	MEGF8	MULTIPLE EGF-LIKE-DOMAINS 8.	X	X	X
IPI00607600	APLP1	AMYLOID PRECURSOR-LIKE PROTEIN 1 ISOFORM 1 PRECURSOR.	X	X	X
IPI00641251	CD320	CD320 ANTIGEN PRECURSOR.	X	X	X
IPI00641737	HP	HAPTOGLOBIN PRECURSOR.	X	X	X
IPI00642259	DST	DYSTONIN.	X	X	X
IPI00642632		C7 PROTEIN.	X	X	X
IPI00644346	ADAMTSL2	ADAMTS-LIKE PROTEIN 2 PRECURSOR.	X	X	X
IPI00645206	PCDH17	ISOFORM 1 OF PROTOCADHERIN-17 PRECURSOR.	X	X	X
IPI00646291	GPR180	INTEGRAL MEMBRANE PROTEIN GPR180 PRECURSOR.	X	X	X
IPI00646304	PPIB	PEPTIDYLPROLYL ISOMERASE B PRECURSOR.	X	X	X
IPI00646689	TXNDC17	THIOREDOXIN DOMAIN-CONTAINING PROTEIN 17.	X	X	X
IPI00647027	CHGB	32 KDA PROTEIN.	X	X	X
IPI00647704	IGHA1; IGHV3OR16-13	CDNA FLJ41552 FIS, CLONE COLON2004478, HIGHLY SIMILAR TO PROTEIN TRO ALPHA1 H,MYELOMA.	X	X	X
IPI00654755	HBB	HEMOGLOBIN SUBUNIT BETA.	X	X	X
IPI00654888	KLKB1	PLASMA KALLIKREIN PRECURSOR.	X	X	X
IPI00718806	AHRR; PDCD6	ARYLHYDROCARBON RECEPTOR REPRESSOR.	X	X	X
IPI00718977	GRIA4	GLUTAMATE RECEPTOR, IONOTROPHIC, AMPA 4 ISOFORM 2 PRECURSOR.	X	X	X
IPI00735451		UNCHARACTERIZED PROTEIN ENSP00000375035.	X	X	X
IPI00736860	ELK2P1	ELK2, MEMBER OF ETS ONCOGENE FAMILY, PSEUDOGENE 1.	X	X	X
IPI00738499	FTL	FERRITIN LIGHT CHAIN.	X	X	X
IPI00739099	COL5A2	COLLAGEN ALPHA-2(V) CHAIN PRECURSOR.	X	X	X
IPI00739827	LAMP2	ISOFORM LAMP-2B OF LYSOSOME-ASSOCIATED MEMBRANE GLYCOPROTEIN 2 PRECURSOR.	X	X	X
IPI00741710	SNED1	ISOFORM 2 OF SUSHI, NIDOGEN AND EGF-LIKE DOMAIN-CONTAINING PROTEIN 1 PRECURSOR.	X	X	X
IPI00742696	GC	VITAMIN D-BINDING PROTEIN PRECURSOR.	X	X	X
IPI00743194		KAPPA LIGHT CHAIN VARIABLE REGION (FRAGMENT).	X	X	X
IPI00743766	FETUB	FETUIN-B PRECURSOR.	X	X	X
IPI00743963	IGKC	IG KAPPA CHAIN V-I REGION HK101 PRECURSOR (FRAGMENT).	X	X	X

Table S1. Proteins Identified or Not in MS Patients Compared to Normals and Other Neurologic Disease (OND)					
**IPI**	**Gene**	**Protein Name**	**MS**	**Normal**	**OND**
IPI00744692	TALDO1	TRANSALDOLASE.	X	X	X
IPI00745660	IGL@	IGL@ PROTEIN.	X	X	X
IPI00746623	HABP2	HYALURONAN-BINDING PROTEIN 2 PRECURSOR.	X	X	X
IPI00746963	IGKC	IGKC PROTEIN.	X	X	X
IPI00747849	ATP1B1	ISOFORM 1 OF SODIUM/POTASSIUM-TRANSPORTING ATPASE SUBUNIT BETA-1.	X	X	X
IPI00748265		RHEUMATOID FACTOR RF-ET13.	X	X	X
IPI00748312	PTPRZ1	PROTEIN TYROSINE PHOSPHATASE, RECEPTOR-TYPE, ZETA1 PRECURSOR.	X	X	X
IPI00748955	GP1BA	PLATELET GLYCOPROTEIN IB ALPHA POLYPEPTIDE PRECURSOR.	X	X	X
IPI00749328	LOC729085	HYPOTHETICAL PROTEIN.	X	X	X
IPI00761159	IGHM	IGHM PROTEIN.	X	X	X
IPI00783024		MYOSIN-REACTIVE IMMUNOGLOBULIN HEAVY CHAIN VARIABLE REGION (FRAGMENT).	X	X	X
IPI00783287		IMMUNGLOBULIN HEAVY CHAIN VARIABLE REGION (FRAGMENT).	X	X	X
IPI00783390	CHL1	ISOFORM 1 OF NEURAL CELL ADHESION MOLECULE L1-LIKE PROTEIN PRECURSOR.	X	X	X
IPI00783393		IMMUNGLOBULIN HEAVY CHAIN VARIABLE REGION (FRAGMENT).	X	X	X
IPI00783399	TMEM132D	ISOFORM 1 OF TRANSMEMBRANE PROTEIN 132D PRECURSOR.	X	X	X
IPI00783665	LAMA5	LAMININ SUBUNIT ALPHA-5 PRECURSOR.	X	X	X
IPI00783987	C3	COMPLEMENT C3 PRECURSOR (FRAGMENT).	X	X	X
IPI00784119	ATP6AP1	VACUOLAR ATP SYNTHASE SUBUNIT S1 PRECURSOR.	X	X	X
IPI00784154	HSPD1	60 KDA HEAT SHOCK PROTEIN, MITOCHONDRIAL PRECURSOR.	X	X	X
IPI00784156	AP2B1	ISOFORM 1 OF AP-2 COMPLEX SUBUNIT BETA-1.	X	X	X
IPI00784430	IGKV3D-11	SIMILAR TO IG KAPPA CHAIN V-III REGION VG PRECURSOR.	X	X	X
IPI00784519		PUTATIVE UNCHARACTERIZED PROTEIN.	X	X	X
IPI00784758	LOC100126583	PUTATIVE UNCHARACTERIZED PROTEIN DKFZP686M08189.	X	X	X
IPI00784807		PUTATIVE UNCHARACTERIZED PROTEIN.	X	X	X
IPI00784810	IGHV4-31	IGHV4-31 PROTEIN.	X	X	X
IPI00784828		PUTATIVE UNCHARACTERIZED PROTEIN DKFZP686C11235.	X	X	X
IPI00784830	LOC100126583	CDNA FLJ41981 FIS, CLONE SMINT2011888, HIGHLY SIMILAR TO PROTEIN TRO ALPHA1 H,MYELOMA.	X	X	X
IPI00784842	IGHV4-31	PUTATIVE UNCHARACTERIZED PROTEIN DKFZP686G11190.	X	X	X
IPI00784865	IGK@	IGK@ PROTEIN.	X	X	X
IPI00784942		PUTATIVE UNCHARACTERIZED PROTEIN DKFZP686E23209.	X	X	X

Table S1. Proteins Identified or Not in MS Patients Compared to Normals and Other Neurologic Disease (OND)					
IPI	Gene	Protein Name	MS	Normal	OND
IPI00784950	LOC100126583	PUTATIVE UNCHARACTERIZED PROTEIN DKFZP686L19235.	X	X	X
IPI00784969	LOC100126583	PUTATIVE UNCHARACTERIZED PROTEIN.	X	X	X
IPI00784998		PUTATIVE UNCHARACTERIZED PROTEIN DKFZP686M24218.	X	X	X
IPI00785067	IGH@	IGH@ PROTEIN.	X	X	X
IPI00785079		PUTATIVE UNCHARACTERIZED PROTEIN.	X	X	X
IPI00787853	IMPAD1	INOSITOL MONOPHOSPHATASE 3.	X	X	X
IPI00789234	VSTM2A	IMMUNOGLOBULIN V-SET DOMAIN CONTAINING PROTEIN.	X	X	X
IPI00789259	IGLV1-40	V1-13 PROTEIN (FRAGMENT).	X	X	X
IPI00790122	TNFSF12	27 KDA PROTEIN.	X	X	X
IPI00793166	SPARCL1	15 KDA PROTEIN.	X	X	X
IPI00793576	PRB4; PRH1; PRH2	7 KDA PROTEIN.	X	X	X
IPI00794184	CP	97 KDA PROTEIN.	X	X	X
IPI00795918	NCAM1	NEURAL CELL ADHESION MOLECULE 1 ISOFORM 2.	X	X	X
IPI00815926	IGHG1	IGHG1 PROTEIN.	X	X	X
IPI00816555		IGLV2-14 PROTEIN.	X	X	X
IPI00816626	PLXNB2	PLXNB2 PROTEIN.	X	X	X
IPI00816737		RHEUMATOID FACTOR D5 HEAVY CHAIN (FRAGMENT).	X	X	X
IPI00816775		F5-20 (FRAGMENT).	X	X	X
IPI00816794		REV25-2 (FRAGMENT).	X	X	X
IPI00827485		BRE (FRAGMENT).	X	X	X
IPI00827510		HRV FAB 026-VL (FRAGMENT).	X	X	X
IPI00827522	IGLV1-44	ANTI-STREPTOCOCCAL/ANTI-MYOSIN IMMUNOGLOBULIN LAMBDA LIGHT CHAIN VARIABLE REGION (FRAGMENT).	X	X	X
IPI00827560		HRV FAB N27-VL (FRAGMENT).	X	X	X
IPI00827580		IMMUNOGOBULIN KAPPA, VJ REGION (FRAGMENT).	X	X	X
IPI00827581		VARIABLE IMMNOGLOBULIN ANTI-ESTRADIOL HEAVY CHAIN (FRAGMENT).	X	X	X
IPI00827637		K LIGHT CHAIN VARIABLE REGION (FRAGMENT).	X	X	X
IPI00827724		RHEUMATOID FACTOR VH I REGION PRECURSOR (FRAGMENT).	X	X	X
IPI00827839		VK3 PROTEIN (FRAGMENT).	X	X	X
IPI00827876		HEAVY CHAIN FAB (FRAGMENT).	X	X	X
IPI00827892		VH87-2 PROTEIN (FRAGMENT).	X	X	X

Table S1. Proteins Identified or Not in MS Patients Compared to Normals and Other Neurologic Disease (OND)					
**IPI**	**Gene**	**Protein Name**	**MS**	**Normal**	**OND**
IPI00827906		ANTI-MUCIN1 LIGHT CHAIN VARIABLE REGION (FRAGMENT).	X	X	X
IPI00827939		ANTI-MUCIN1 LIGHT CHAIN VARIABLE REGION (FRAGMENT).	X	X	X
IPI00827978		VL4 PROTEIN (FRAGMENT).	X	X	X
IPI00828083		HEAVY CHAIN FAB (FRAGMENT).	X	X	X
IPI00828105		ANTI-MPL SCFV (FRAGMENT).	X	X	X
IPI00828205	IGHM	IGHM PROTEIN.	X	X	X
IPI00829711	IGHA2	UNCHARACTERIZED PROTEIN IGHA2 (FRAGMENT).	X	X	X
IPI00829767	IGHG2	UNCHARACTERIZED PROTEIN IGHG2 (FRAGMENT).	X	X	X
IPI00829810		UNCHARACTERIZED PROTEIN ENSP00000375027.	X	X	X
IPI00829812		UNCHARACTERIZED PROTEIN ENSP00000375011.	X	X	X
IPI00829827		UNCHARACTERIZED PROTEIN ENSP00000374804.	X	X	X
IPI00829834		IG KAPPA CHAIN V-III REGION VH PRECURSOR.	X	X	X
IPI00829836		UNCHARACTERIZED PROTEIN ENSP00000374797.	X	X	X
IPI00829877	IGL@	IGL@ PROTEIN.	X	X	X
IPI00829980		MYOSIN-REACTIVE IMMUNOGLOBULIN LIGHT CHAIN VARIABLE REGION (FRAGMENT).	X	X	X
IPI00830018		UNCHARACTERIZED PROTEIN ENSP00000374807.	X	X	X
IPI00830025		UNCHARACTERIZED PROTEIN ENSP00000375021.	X	X	X
IPI00830035		SIMILAR TO ANTI-STREPTOCOCCAL/ANTI-MYOSIN IMMUNOGLOBULIN KAPPA LIGHT CHAIN VARIABLE REGION.	X	X	X
IPI00830057		UNCHARACTERIZED PROTEIN ENSP00000374791.	X	X	X
IPI00830122		A30.	X	X	X
IPI00843910	FUCA1	TISSUE ALPHA-L-FUCOSIDASE PRECURSOR.	X	X	X
IPI00844156	SERPINC1	SERPINC1 PROTEIN.	X	X	X
IPI00845354	IGKC	IGKC PROTEIN.	X	X	X
IPI00852577	IGLC1	IGLC1 PROTEIN.	X	X	X
IPI00852979	C11orf41	HYPOTHETICAL PROTEIN LOC25758.	X	X	X
IPI00853045	IGKC	ANTI-RHD MONOCLONAL T125 KAPPA LIGHT CHAIN PRECURSOR.	X	X	X
IPI00853369	PLXNB2	PLEXIN-B2 PRECURSOR.	X	X	X
IPI00854624		UNCHARACTERIZED PROTEIN ENSP00000375043.	X	X	X
IPI00854644		UNCHARACTERIZED PROTEIN ENSP00000374805.	X	X	X
IPI00854745		UNCHARACTERIZED PROTEIN ENSP00000375019.	X	X	X
IPI00854841		UNCHARACTERIZED PROTEIN ENSP00000375033.	X	X	X
IPI00855824	DPP6	DIPEPTIDYL-PEPTIDASE 6 ISOFORM 3.	X	X	X
IPI00871326	PLXNA1	PLEXIN A1.	X	X	X

Table S1. Proteins Identified or Not in MS Patients Compared to Normals and Other Neurologic Disease (OND)					
IPI	Gene	Protein Name	MS	Normal	OND
IPI00872363	PTPRD	PTPRD PROTEIN.	X	X	X
IPI00879436	TCN2	47 KDA PROTEIN.	X	X	X
IPI00880120	ABHD14A	ABHYDROLASE DOMAIN-CONTAINING PROTEIN 14A.	X	X	X
IPI00883753	NRCAM	NRCAM PROTEIN.	X	X	X
IPI00883765		SIMILAR TO IMMUNGLOBULIN HEAVY CHAIN VARIABLE REGION.	X	X	X
IPI00883855		SIMILAR TO HEPATITIS B VIRUS RECEPTOR BINDING PROTEIN.	X	X	X
IPI00884080		SIMILAR TO IMMUNGLOBULIN HEAVY CHAIN VARIABLE REGION.	X	X	X
IPI00884389		SIMILAR TO IMMUNGLOBULIN HEAVY CHAIN VARIABLE REGION.	X	X	X
IPI00001712	CTNNA3	ISOFORM 1 OF CATENIN ALPHA-3.	X	X	
IPI00004346	CCR10	C-C CHEMOKINE RECEPTOR TYPE 10.	X	X	
IPI00005675	NKRF	NF-KAPPA-B-REPRESSING FACTOR.	X	X	
IPI00005719	RAB1A	ISOFORM 1 OF RAS-RELATED PROTEIN RAB-1A.	X	X	
IPI00005981	TAGLN3	TRANSGELIN-3.	X	X	
IPI00007928	PRPF8	PRE-MRNA-PROCESSING-SPLICING FACTOR 8.	X	X	
IPI00008091	INOC1	PUTATIVE DNA HELICASE INO80 COMPLEX HOMOLOG 1.	X	X	
IPI00008282	PDE1A	ISOFORM 1 OF CALCIUM/CALMODULIN-DEPENDENT 3',5'-CYCLIC NUCLEOTIDE PHOSPHODIESTERASE 1A.	X	X	
IPI00008404	LOC652788	ISOFORM LONG OF SEGMENT POLARITY PROTEIN DISHEVELLED HOMOLOG DVL-1-LIKE.	X	X	
IPI00009791	ATP2B2	ISOFORM WB OF PLASMA MEMBRANE CALCIUM-TRANSPORTING ATPASE 2.	X	X	
IPI00010303	SERPINB4	SERPIN B4.	X	X	
IPI00010360	COL4A3	ISOFORM 1 OF COLLAGEN ALPHA-3(IV) CHAIN PRECURSOR.	X	X	
IPI00010405	ROR1	ISOFORM LONG OF TYROSINE-PROTEIN KINASE TRANSMEMBRANE RECEPTOR ROR1 PRECURSOR.	X	X	
IPI00011564	SDC4	SYNDECAN-4 PRECURSOR.	X	X	
IPI00013290	HDGF2	HEPATOMA-DERIVED GROWTH FACTOR-RELATED PROTEIN 2 ISOFORM 1.	X	X	
IPI00013508	ACTN1	ALPHA-ACTININ-1.	X	X	
IPI00013933	DSP	ISOFORM DPI OF DESMOPLAKIN.	X	X	
IPI00015285	PCYT2	ETHANOLAMINE-PHOSPHATE CYTIDYLYLTRANSFERASE.	X	X	
IPI00016949	SLC4A4	ISOFORM 4 OF ELECTROGENIC SODIUM BICARBONATE COTRANSPORTER 1.	X	X	
IPI00017163	HECW2	ISOFORM 1 OF E3 UBIQUITIN-PROTEIN LIGASE HECW2.	X	X	
IPI00017567	ENG	ISOFORM LONG OF ENDOGLIN PRECURSOR.	X	X	
IPI00018027	AGGF1	ISOFORM 1 OF ANGIOGENIC FACTOR WITH G PATCH AND FHA DOMAINS 1.	X	X	
IPI00018275	PRND	PRION-LIKE PROTEIN DOPPEL PRECURSOR.	X	X	

Table S1. Proteins Identified or Not in MS Patients Compared to Normals and Other Neurologic Disease (OND)					
**IPI**	**Gene**	**Protein Name**	**MS**	**Normal**	**OND**
IPI00019502	MYH9	MYOSIN-9.	X	X	
IPI00020884	PLXNA3	PLEXIN-A3 PRECURSOR.	X	X	
IPI00021363	JARID1A	HISTONE DEMETHYLASE JARID1A.	X	X	
IPI00021766	RTN4	ISOFORM 1 OF RETICULON-4.	X	X	
IPI00021831	PRKAR1A	CAMP-DEPENDENT PROTEIN KINASE TYPE I-ALPHA REGULATORY SUBUNIT.	X	X	
IPI00023315	GDF10	BONE MORPHOGENETIC PROTEIN 3B PRECURSOR.	X	X	
IPI00024689	AQP1	AQUAPORIN-1.	X	X	
IPI00024802	BTAF1	TATA-BINDING PROTEIN-ASSOCIATED FACTOR 172.	X	X	
IPI00027547	DCD	DERMCIDIN PRECURSOR.	X	X	
IPI00028450	SLC8A1	ISOFORM 1 OF SODIUM/CALCIUM EXCHANGER 1 PRECURSOR.	X	X	
IPI00028932	MAST3	MICROTUBULE-ASSOCIATED SERINE/THREONINE-PROTEIN KINASE 3.	X	X	
IPI00029012	EIF3A	EUKARYOTIC TRANSLATION INITIATION FACTOR 3 SUBUNIT A.	X	X	
IPI00029107	WRN	WERNER SYNDROME ATP-DEPENDENT HELICASE.	X	X	
IPI00029372	C4orf15	UNCHARACTERIZED PROTEIN C4ORF15.	X	X	
IPI00030352	C10orf28	ISOFORM 2 OF GROWTH INHIBITION AND DIFFERENTIATION-RELATED PROTEIN 88.	X	X	
IPI00030741	LCA5L	UNCHARACTERIZED PROTEIN C21ORF13.	X	X	
IPI00031907	TMEM108	ISOFORM 1 OF TRANSMEMBRANE PROTEIN 108 PRECURSOR.	X	X	
IPI00034006	PTPN23	TYROSINE-PROTEIN PHOSPHATASE NON-RECEPTOR TYPE 23.	X	X	
IPI00043201	CENPJ	CENTROMERE PROTEIN J.	X	X	
IPI00167941	MDN1	MIDASIN.	X	X	
IPI00168806	MLL3	ISOFORM 1 OF MYELOID/LYMPHOID OR MIXED-LINEAGE LEUKEMIA PROTEIN 3 HOMOLOG.	X	X	
IPI00183002	PPP1R12A	ISOFORM 1 OF PROTEIN PHOSPHATASE 1 REGULATORY SUBUNIT 12A.	X	X	
IPI00184650	BHLHB4	CLASS B BASIC HELIX-LOOP-HELIX PROTEIN 4.	X	X	
IPI00184997	DLG7	CDNA FLJ78771, HIGHLY SIMILAR TO HOMO SAPIENS DISCS, LARGE HOMOLOG 7 (DROSOPHILA), MRNA.	X	X	
IPI00186581	OS9	AMPLIFIED IN OSTEOSARCOMA ISOFORM 2 PRECURSOR.	X	X	
IPI00215746	FABP4	FATTY ACID-BINDING PROTEIN, ADIPOCYTE.	X	X	
IPI00221235	NUP160	NUCLEOPORIN 160KDA.	X	X	
IPI00240793	ATP11B	PROBABLE PHOSPHOLIPID-TRANSPORTING ATPASE IF.	X	X	
IPI00289271	PPFIA2	LIPRIN-ALPHA-2.	X	X	
IPI00293679	KCNQ4	ISOFORM 1 OF POTASSIUM VOLTAGE-GATED CHANNEL SUBFAMILY KQT MEMBER 4.	X	X	
IPI00294713	MASP2	ISOFORM 1 OF MANNAN-BINDING LECTIN SERINE PROTEASE 2 PRECURSOR.	X	X	
IPI00295577	PTPRB	RECEPTOR-TYPE TYROSINE-PROTEIN PHOSPHATASE BETA PRECURSOR.	X	X	

Table S1. Proteins Identified or Not in MS Patients Compared to Normals and Other Neurologic Disease (OND)					
**IPI**	**Gene**	**Protein Name**	**MS**	**Normal**	**OND**
IPI00296374	ZFPL1	ZINC FINGER PROTEIN-LIKE 1.	X	X	
IPI00298258	UNC13B	UNC13B PROTEIN.	X	X	
IPI00300052	KRT84	KERATIN TYPE II CUTICULAR HB4.	X	X	
IPI00307611	MAST4	ISOFORM 1 OF MICROTUBULE-ASSOCIATED SERINE/THREONINE-PROTEIN KINASE 4.	X	X	
IPI00328361	SARS2	SERYL-TRNA SYNTHETASE, MITOCHONDRIAL PRECURSOR.	X	X	
IPI00328826	CADPS2	UNCHARACTERIZED PROTEIN CADPS2.	X	X	
IPI00333371	L3MBTL	ISOFORM 2 OF LETHAL.	X	X	
IPI00337385	PRPF40A	ISOFORM 1 OF PRE-MRNA-PROCESSING FACTOR 40 HOMOLOG A.	X	X	
IPI00374862	KLHL5	ISOFORM 1 OF KELCH-LIKE PROTEIN 5.	X	X	
IPI00410585	CRB2	ISOFORM 1 OF CRUMBS HOMOLOG 2 PRECURSOR.	X	X	
IPI00429191	ETF1	EUKARYOTIC PEPTIDE CHAIN RELEASE FACTOR SUBUNIT 1.	X	X	
IPI00456635	UNC13D	ISOFORM 1 OF PROTEIN UNC-13 HOMOLOG D.	X	X	
IPI00470490	NCOA1	ISOFORM 1 OF NUCLEAR RECEPTOR COACTIVATOR 1.	X	X	
IPI00470805	MDC1	ISOFORM 2 OF MEDIATOR OF DNA DAMAGE CHECKPOINT PROTEIN 1.	X	X	
IPI00472332	LOC653441	SIMILAR TO POLYHOMEOTIC 1-LIKE ISOFORM 4.	X	X	
IPI00641181	MARCKSL1	MARCKS-RELATED PROTEIN.	X	X	
IPI00737920	DNAH3	SIMILAR TO DYNEIN, AXONEMAL, HEAVY POLYPEPTIDE 1.	X	X	
IPI00737969	MICAL3	MICROTUBULE ASSOCIATED MONOXYGENASE, CALPONIN AND LIM DOMAIN CONTAINING 3.	X	X	
IPI00743284	MTR	METHIONINE SYNTHASE.	X	X	
IPI00853516	DNAH17	DYNEIN, AXONEMAL, HEAVY CHAIN 17.	X	X	
IPI00856012	COL6A6	COLLAGEN TYPE VI ALPHA 6.	X	X	
IPI00000203	LRP6	LOW-DENSITY LIPOPROTEIN RECEPTOR-RELATED PROTEIN 6 PRECURSOR.	X		X
IPI00000436	CDH22	CADHERIN-22 PRECURSOR.	X		X
IPI00000784	MEF2D	ISOFORM MEF2DAB OF MYOCYTE-SPECIFIC ENHANCER FACTOR 2D.	X		X
IPI00000873	VARS	VALYL-TRNA SYNTHETASE.	X		X
IPI00000949	CRYM	MU-CRYSTALLIN HOMOLOG.	X		X
IPI00001328		PROTEIN.	X		X
IPI00001528	IL18BP	ISOFORM C OF INTERLEUKIN-18-BINDING PROTEIN PRECURSOR.	X		X
IPI00002405	OAS3	2'-5'-OLIGOADENYLATE SYNTHETASE 3.	X		X
IPI00002689	TRPC4	ISOFORM ALPHA OF SHORT TRANSIENT RECEPTOR POTENTIAL CHANNEL 4.	X		X
IPI00002899	LY6E	LYMPHOCYTE ANTIGEN 6E PRECURSOR.	X		X
IPI00003375	CCL14	ISOFORM HCC-1 OF C-C MOTIF CHEMOKINE 14 PRECURSOR.	X		X
IPI00003469		IG KAPPA CHAIN V-I REGION WEA.	X		X

Table S1. Proteins Identified or Not in MS Patients Compared to Normals and Other Neurologic Disease (OND)					
**IPI**	**Gene**	**Protein Name**	**MS**	**Normal**	**OND**
IPI00003564	LY86	LYMPHOCYTE ANTIGEN 86 PRECURSOR.	X		X
IPI00003927	PPID	40 KDA PEPTIDYL-PROLYL CIS-TRANS ISOMERASE.	X		X
IPI00004379	HMX1	HOMEOBOX PROTEIN HMX1.	X		X
IPI00004521	ETAA1	EWING'S TUMOR-ASSOCIATED ANTIGEN 1.	X		X
IPI00004970	UTP20	SMALL SUBUNIT PROCESSOME COMPONENT 20 HOMOLOG.	X		X
IPI00005104	CHUK	INHIBITOR OF NUCLEAR FACTOR KAPPA-B KINASE SUBUNIT ALPHA.	X		X
IPI00005667	N4BP1	NEDD4-BINDING PROTEIN 1.	X		X
IPI00005806	ST7	ST7 FORM 2 SPLICE VARIANT A.	X		X
IPI00006196	NUMA1	ISOFORM 2 OF NUCLEAR MITOTIC APPARATUS PROTEIN 1.	X		X
IPI00006648	RABGAP1L	IDN4-GGTR14 PROTEIN.	X		X
IPI00006653	ATP2C2	CALCIUM-TRANSPORTING ATPASE TYPE 2C MEMBER 2.	X		X
IPI00006705	SCGB1A1	UTEROGLOBIN PRECURSOR.	X		X
IPI00006904	AVEN	CELL DEATH REGULATOR AVEN.	X		X
IPI00006932	LUC7L2	ISOFORM 1 OF PUTATIVE RNA-BINDING PROTEIN LUC7-LIKE 2.	X		X
IPI00007127	DPEP2	ISOFORM 1 OF DIPEPTIDASE 2 PRECURSOR.	X		X
IPI00007729	NOL7	ISOFORM 1 OF NUCLEOLAR PROTEIN 7.	X		X
IPI00007800	ANGPTL2	ANGIOPOIETIN-RELATED PROTEIN 2 PRECURSOR.	X		X
IPI00008403	CHST7	CARBOHYDRATE SULFOTRANSFERASE 7.	X		X
IPI00008455	MYO6	ISOFORM 2 OF MYOSIN-VI.	X		X
IPI00008554	ANG	ANGIOGENIN PRECURSOR.	X		X
IPI00008752	MT1G	ISOFORM 1 OF METALLOTHIONEIN-1G.	X		X
IPI00008832	GAS1	GROWTH ARREST-SPECIFIC PROTEIN 1 PRECURSOR.	X		X
IPI00008913	ST6GALNAC5	ALPHA-N-ACETYLGALACTOSAMINIDE ALPHA-2,6-SIALYLTRANSFERASE 5.	X		X
IPI00009286	MLL	ISOFORM 1 OF ZINC FINGER PROTEIN HRX.	X		X
IPI00009305	GNPDA1	GLUCOSAMINE-6-PHOSPHATE ISOMERASE.	X		X
IPI00009329	UTRN	UTROPHIN.	X		X
IPI00010676	PLAUR	ISOFORM 1 OF UROKINASE PLASMINOGEN ACTIVATOR SURFACE RECEPTOR PRECURSOR.	X		X
IPI00010859	APITD1; CORT	CORTISTATIN PREPROPROTEIN.	X		X
IPI00010869	AGRP	AGOUTI-RELATED PROTEIN PRECURSOR.	X		X
IPI00011062	CPS1	ISOFORM 1 OF CARBAMOYL-PHOSPHATE SYNTHASE [AMMONIA], MITOCHONDRIAL PRECURSOR.	X		X
IPI00011092	USP26	UBIQUITIN CARBOXYL-TERMINAL HYDROLASE 26.	X		X
IPI00011961	SIGLEC1	ISOFORM 1 OF SIALOADHESIN PRECURSOR.	X		X

Table S1. Proteins Identified or Not in MS Patients Compared to Normals and Other Neurologic Disease (OND)					
IPI	Gene	Protein Name	MS	Normal	OND
IPI00012390	HOXD4	HOMEOBOX PROTEIN HOX-D4.	X		X
IPI00012540	PROM1	PROMININ-1 PRECURSOR.	X		X
IPI00012622	ARHGAP20	ISOFORM 1 OF RHO GTPASE-ACTIVATING PROTEIN 20.	X		X
IPI00014845	DNAH8	ISOFORM 1 OF CILIARY DYNEIN HEAVY CHAIN 8.	X		X
IPI00014852	PGM5	ISOFORM 1 OF PHOSPHOGLUCOMUTASE-LIKE PROTEIN 5.	X		X
IPI00015343	EDG1	ENDOTHELIAL DIFFERENTIATION, SPHINGOLIPID G-PROTEIN-COUPLED RECEPTOR, 1.	X		X
IPI00015614	PRSS3	ISOFORM A OF TRYPSIN-3 PRECURSOR.	X		X
IPI00015877	TMEM59L	TRANSMEMBRANE PROTEIN 59-LIKE PRECURSOR.	X		X
IPI00016703	DHCR24	24-DEHYDROCHOLESTEROL REDUCTASE PRECURSOR.	X		X
IPI00016848	C20orf103	UNCHARACTERIZED PROTEIN C20ORF103 PRECURSOR.	X		X
IPI00017231	NRSN2	NEURENSIN-2.	X		X
IPI00017451	SF3A1	SPLICING FACTOR 3 SUBUNIT 1.	X		X
IPI00017480	SHH	SONIC HEDGEHOG PROTEIN PRECURSOR.	X		X
IPI00018134	FLJ10357	ISOFORM 4 OF PROTEIN SOLO.	X		X
IPI00018335	FLT1	ISOFORM FLT1 OF VASCULAR ENDOTHELIAL GROWTH FACTOR RECEPTOR 1 PRECURSOR.	X		X
IPI00020228	FZD6	FRIZZLED-6 PRECURSOR.	X		X
IPI00020406	MGAT3	BETA-1,4-MANNOSYL-GLYCOPROTEIN 4-BETA-N-ACETYLGLUCOSAMINYLTRANSFERASE.	X		X
IPI00021129	AP3B1	ISOFORM 1 OF AP-3 COMPLEX SUBUNIT BETA-1.	X		X
IPI00021786	RAF1	RAF PROTO-ONCOGENE SERINE/THREONINE-PROTEIN KINASE.	X		X
IPI00022020	INPP4B	TYPE II INOSITOL-3,4-BISPHOSPHATE 4-PHOSPHATASE.	X		X
IPI00022629	PRC1	ISOFORM 1 OF PROTEIN REGULATOR OF CYTOKINESIS 1.	X		X
IPI00023105	PAMCI	PEPTIDYLGLYCINE ALPHA-AMIDATING MONOOXYGENASE COOH-TERMINAL INTERACTOR.	X		X
IPI00023283	TTN	ISOFORM 2 OF TITIN.	X		X
IPI00023501	TNFRSF1B	ISOFORM 1 OF TUMOR NECROSIS FACTOR RECEPTOR SUPERFAMILY MEMBER 1B PRECURSOR.	X		X
IPI00023604	TRH	THYROLIBERIN PRECURSOR.	X		X
IPI00023868	ABCC2	CANALICULAR MULTISPECIFIC ORGANIC ANION TRANSPORTER 1.	X		X
IPI00024134	IGKC	IG KAPPA CHAIN V-I REGION WALKER PRECURSOR.	X		X
IPI00024714	RGS12	ISOFORM 1 OF REGULATOR OF G-PROTEIN SIGNALING 12.	X		X
IPI00024975	KIF15	KINESIN-LIKE PROTEIN KIF15.	X		X

Table S1. Proteins Identified or Not in MS Patients Compared to Normals and Other Neurologic Disease (OND)					
**IPI**	**Gene**	**Protein Name**	**MS**	**Normal**	**OND**
IPI00025155	FSTL3	FOLLISTATIN-RELATED PROTEIN 3 PRECURSOR.	X		X
IPI00025295	ENTPD6	ECTONUCLEOSIDE TRIPHOSPHATE DIPHOSPHOHYDROLASE 6.	X		X
IPI00025753	DSG1	DESMOGLEIN-1 PRECURSOR.	X		X
IPI00026970	SUPT16H	FACT COMPLEX SUBUNIT SPT16.	X		X
IPI00027212	INSRR	INSULIN RECEPTOR-RELATED PROTEIN PRECURSOR.	X		X
IPI00027765	RTBDN	RETBINDIN ISOFORM 2.	X		X
IPI00029628	RCN2	RETICULOCALBIN-2 PRECURSOR.	X		X
IPI00030153	TDRD6	TUDOR DOMAIN-CONTAINING PROTEIN 6.	X		X
IPI00031171	IL6R	ISOFORM 1 OF INTERLEUKIN-6 RECEPTOR ALPHA CHAIN PRECURSOR.	X		X
IPI00031630	SILV	MELANOCYTE PROTEIN PMEL 17 PRECURSOR.	X		X
IPI00041588	RALGPS1	RAL GUANINE NUCLEOTIDE EXCHANGE FACTOR RALGPS1A.	X		X
IPI00048230	NXPH1	NEUREXOPHILIN-1 PRECURSOR.	X		X
IPI00054521	FZD1	FRIZZLED-1 PRECURSOR.	X		X
IPI00056040	NRN1L	NEURITIN 1-LIKE.	X		X
IPI00056309	LEAP2	LIVER-EXPRESSED ANTIMICROBIAL PEPTIDE 2 PRECURSOR.	X		X
IPI00057815	SLC2A7	INTESTINAL FACILITATIVE GLUCOSE TRANSPORTER 7.	X		X
IPI00059279	EXOC4	EXOCYST COMPLEX COMPONENT 4.	X		X
IPI00062467	ZNF274	ISOFORM 1 OF ZINC FINGER PROTEIN 274.	X		X
IPI00064429	DKFZP586H21 23	ISOFORM 2 OF INACTIVE SERINE PROTEASE RAMP PRECURSOR.	X		X
IPI00064767	ARHGAP17	ISOFORM 1 OF RHO GTPASE-ACTIVATING PROTEIN 17.	X		X
IPI00065351	ZNF354B	ZINC FINGER PROTEIN 354B.	X		X
IPI00065533	FAM20A	PROTEIN FAM20A PRECURSOR.	X		X
IPI00083708	BAT2D1	BAT2-ISO.	X		X
IPI00088953	C17orf38	PHOSPHOINOSITIDE 3-KINASE REGULATORY SUBUNIT 6.	X		X
IPI00100910	ARMC5	ISOFORM 2 OF ARMADILLO REPEAT-CONTAINING PROTEIN 5.	X		X
IPI00150057	SMARCC2	ISOFORM 2 OF SWI/SNF-RELATED MATRIX-ASSOCIATED ACTIN-DEPENDENT REGULATOR OF CHROMATIN SUBFAMILY C MEMBER 2.	X		X
IPI00152033	TRPM8	ISOFORM 1 OF TRANSIENT RECEPTOR POTENTIAL CATION CHANNEL SUBFAMILY M MEMBER 8.	X		X
IPI00152257	C10orf63	ENKURIN.	X		X
IPI00152462	DNAH3	CILIARY DYNEIN HEAVY CHAIN 3.	X		X
IPI00152535	CHD5	CHROMODOMAIN-HELICASE-DNA-BINDING PROTEIN 5.	X		X
IPI00152685	TTC15	TETRATRICOPEPTIDE REPEAT PROTEIN 15.	X		X

Table S1. Proteins Identified or Not in MS Patients Compared to Normals and Other Neurologic Disease (OND)					
**IPI**	**Gene**	**Protein Name**	**MS**	**Normal**	**OND**
IPI00152692	DTD1	D-TYROSYL-TRNA(TYR) DEACYLASE 1.	X		X
IPI00159072	ROD1	57 KDA PROTEIN.	X		X
IPI00164352	ZNF292	ZINC FINGER PROTEIN 292.	X		X
IPI00164776	TMEM198	SIMILAR TO RIKEN CDNA A230078I05 GENE.	X		X
IPI00167920	GOLGA9P	CDNA FLJ35785 FIS, CLONE TESTI2005603, MODERATELY SIMILAR TO HOMO SAPIENS 88-KDA GOLGI PROTEIN (GM88) MRNA.	X		X
IPI00168239	GLIS1	ZINC FINGER PROTEIN GLIS1.	X		X
IPI00168418	C11orf64	PUTATIVE UNCHARACTERIZED PROTEIN C11ORF64.	X		X
IPI00174347	DUSP27	INACTIVE DUAL SPECIFICITY PHOSPHATASE 27.	X		X
IPI00176210	FLJ40243	HYPOTHETICAL PROTEIN LOC133558.	X		X
IPI00177890	SMARCD2	54 KDA PROTEIN.	X		X
IPI00216003	CUL5	CULLIN-5.	X		X
IPI00216423	ITSN2	ISOFORM 4 OF INTERSECTIN-2.	X		X
IPI00217507	NEFM	NEUROFILAMENT MEDIUM POLYPEPTIDE.	X		X
IPI00217688	C12orf60	UNCHARACTERIZED PROTEIN C12ORF60.	X		X
IPI00217691	C17orf57	ISOFORM 1 OF EF-HAND DOMAIN-CONTAINING PROTEIN C17ORF57.	X		X
IPI00217787	C12orf53	ISOFORM 1 OF UNCHARACTERIZED PROTEIN C12ORF53 PRECURSOR.	X		X
IPI00219358	MPI	ISOFORM 1 OF MANNOSE-6-PHOSPHATE ISOMERASE.	X		X
IPI00239405	SYNE2	ISOFORM 1 OF NESPRIN-2.	X		X
IPI00256974	OSTN	OSTEOCRIN PRECURSOR.	X		X
IPI00289540	USH2A	ISOFORM 2 OF USHERIN PRECURSOR.	X		X
IPI00289758	CAPN2	CALPAIN-2 CATALYTIC SUBUNIT PRECURSOR.	X		X
IPI00289849	ELFN2	LEUCINE-RICH REPEAT AND FIBRONECTIN TYPE-III DOMAIN-CONTAINING PROTEIN 6 PRECURSOR.	X		X
IPI00291796	BTBD12	BTB (POZ) DOMAIN CONTAINING 12.	X		X
IPI00291878	SFTPD	PULMONARY SURFACTANT-ASSOCIATED PROTEIN D PRECURSOR.	X		X
IPI00293200	PARC	OTTHUMP00000016423.	X		X
IPI00293588	TMEFF1	ISOFORM 1 OF TOMOREGULIN-1 PRECURSOR.	X		X
IPI00294650	FRZB	SECRETED FRIZZLED-RELATED PROTEIN 3 PRECURSOR.	X		X
IPI00295940	UNC84B	PROTEIN UNC-84 HOMOLOG B.	X		X
IPI00296798	FIBCD1	ISOFORM 1 OF FIBRINOGEN C DOMAIN-CONTAINING PROTEIN 1.	X		X
IPI00296830	LUZP1	ISOFORM 1 OF LEUCINE ZIPPER PROTEIN 1.	X		X
IPI00297315	ADRA2B	ALPHA-2B ADRENERGIC RECEPTOR.	X		X
IPI00302614	VTCN1	V-SET DOMAIN CONTAINING T CELL ACTIVATION INHIBITOR 1.	X		X

Table S1. Proteins Identified or Not in MS Patients Compared to Normals and Other Neurologic Disease (OND)					
IPI	Gene	Protein Name	MS	Normal	OND
IPI00303152	COL22A1	COLLAGEN, TYPE XXII, ALPHA 1.	X		X
IPI00303335	NEB	NEBULIN.	X		X
IPI00304533	MFRP	MEMBRANE FRIZZLED-RELATED PROTEIN.	X		X
IPI00304992	ZNF503	ISOFORM 1 OF ZINC FINGER PROTEIN 503.	X		X
IPI00306851	LRP4	LOW-DENSITY LIPOPROTEIN RECEPTOR-RELATED PROTEIN 4 PRECURSOR.	X		X
IPI00328118	SPAG5	SPERM-ASSOCIATED ANTIGEN 5.	X		X
IPI00328762	ABCA13	ISOFORM 1 OF ATP-BINDING CASSETTE SUB-FAMILY A MEMBER 13.	X		X
IPI00335711	DNAH11	CILIARY DYNEIN HEAVY CHAIN 11.	X		X
IPI00339217	OVCH1	OVOCHYMASE-1 PRECURSOR.	X		X
IPI00375294	LAMA1	LAMININ SUBUNIT ALPHA-1 PRECURSOR.	X		X
IPI00376197	C1QL2	COMPLEMENT C1Q-LIKE PROTEIN 2 PRECURSOR.	X		X
IPI00376210	GPR142	PROBABLE G-PROTEIN COUPLED RECEPTOR 142.	X		X
IPI00376436	VPS13B	ISOFORM 4 OF VACUOLAR PROTEIN SORTING-ASSOCIATED PROTEIN 13B.	X		X
IPI00382534		IG HEAVY CHAIN V-II REGION OU.	X		X
IPI00382539		IG HEAVY CHAIN V-II REGION WAH.	X		X
IPI00384393		MYOSIN-REACTIVE IMMUNOGLOBULIN HEAVY CHAIN VARIABLE REGION (FRAGMENT).	X		X
IPI00385683		IG HEAVY CHAIN V-III REGION GAR.	X		X
IPI00386418	MYEF2	ISOFORM 2 OF MYELIN EXPRESSION FACTOR 2.	X		X
IPI00387025		IG KAPPA CHAIN V-I REGION DEE.	X		X
IPI00387100		IG KAPPA CHAIN V-I REGION ROY.	X		X
IPI00396145	PIP5K3	FYVE FINGER-CONTAINING PHOSPHOINOSITIDE KINASE.	X		X
IPI00397526	MYH10	ISOFORM 1 OF MYOSIN-10.	X		X
IPI00398007	USP40	UBIQUITIN SPECIFIC PROTEASE 40.	X		X
IPI00398728	RP1L1	ISOFORM 1 OF RETINITIS PIGMENTOSA 1-LIKE 1 PROTEIN.	X		X
IPI00402005	ZNF528	SIMILAR TO DNA-BINDING PROTEIN.	X		X
IPI00402280	CHADL	ISOFORM 1 OF SMALL LEUCINE-RICH PROTEOGLYCAN FAMILY MEMBER LOC150356 PRECURSOR.	X		X
IPI00410127	TACC2	TRANSFORMING, ACIDIC COILED-COIL CONTAINING PROTEIN 2.	X		X
IPI00410325	LDLRAD3	LOW-DENSITY LIPOPROTEIN RECEPTOR CLASS A DOMAIN-CONTAINING PROTEIN 3 PRECURSOR.	X		X
IPI00410666	SCRIB	ISOFORM 3 OF PROTEIN LAP4.	X		X
IPI00412647	THADA	CDNA: FLJ21792 FIS, CLONE HEP00441.	X		X
IPI00412982	NOTCH1	NEUROGENIC LOCUS NOTCH HOMOLOG PROTEIN 1 PRECURSOR.	X		X

Table S1. Proteins Identified or Not in MS Patients Compared to Normals and Other Neurologic Disease (OND)					
**IPI**	**Gene**	**Protein Name**	**MS**	**Normal**	**OND**
IPI00413266	IQCA	ISOFORM 1 OF IQ AND AAA DOMAIN-CONTAINING PROTEIN.	X		X
IPI00414927	C1orf32	TRANSMEMBRANE AND IMMUNOGLOBULIN DOMAIN-CONTAINING PROTEIN C1ORF32.	X		X
IPI00415037	ADAM12	ISOFORM 12L OF ADAM 12 PRECURSOR.	X		X
IPI00418966	LOC123688	HYPOTHETICAL PROTEIN LOC123688 ISOFORM 2.	X		X
IPI00419675	LOC150763	HYPOTHETICAL PROTEIN LOC150763.	X		X
IPI00419922	IQCE	ISOFORM 1 OF IQ DOMAIN-CONTAINING PROTEIN E.	X		X
IPI00433499	RHBDF2	RHOMBOID, VEINLET-LIKE 6 ISOFORM 1.	X		X
IPI00442474	COG7	CDNA FLJ27204 FIS, CLONE SYN03230.	X		X
IPI00442909	IGHV4-31	CDNA FLJ26301 FIS, CLONE DMC07540.	X		X
IPI00449923	RAI1	ISOFORM 1 OF RETINOIC ACID-INDUCED PROTEIN 1.	X		X
IPI00456484	PDE4DIP	ISOFORM 3 OF MYOMEGALIN.	X		X
IPI00456601	SYNPO2L	ISOFORM 1 OF SYNAPTOPODIN 2-LIKE PROTEIN.	X		X
IPI00456604	FAM19A1	PROTEIN FAM19A1 PRECURSOR.	X		X
IPI00456676	FBXO38	ISOFORM 1 OF F-BOX ONLY PROTEIN 38.	X		X
IPI00457109	ABCA12	ISOFORM 2 OF ATP-BINDING CASSETTE SUB-FAMILY A MEMBER 12.	X		X
IPI00465147	C9orf97	ISOFORM 1 OF UNCHARACTERIZED PROTEIN C9ORF97.	X		X
IPI00477539	C14orf102	UNCHARACTERIZED PROTEIN C14ORF102.	X		X
IPI00477820	FREM1	ISOFORM 1 OF FRAS1-RELATED EXTRACELLULAR MATRIX PROTEIN 1 PRECURSOR.	X		X
IPI00478586	VPS13A	ISOFORM 2 OF VACUOLAR PROTEIN SORTING-ASSOCIATED PROTEIN 13A.	X		X
IPI00478921	MPZ	MYELIN PROTEIN ZERO.	X		X
IPI00479279	CCDC144A; CCDC144B; LOC652491; LOC731479	ISOFORM 1 OF COILED-COIL DOMAIN-CONTAINING PROTEIN 144A.	X		X
IPI00514774	PAN3	ISOFORM 1 OF PAB-DEPENDENT POLY(A)-SPECIFIC RIBONUCLEASE SUBUNIT 3.	X		X
IPI00551062	CNPY3	ISOFORM 1 OF PROTEIN CANOPY HOMOLOG 3 PRECURSOR.	X		X
IPI00552787	RBM20	SIMILAR TO MATRIN-3.	X		X
IPI00553215	IGLV2-18	V1-5 PROTEIN.	X		X
IPI00556253	C6orf199	ISOFORM 1 OF COILED-COIL DOMAIN-CONTAINING PROTEIN C6ORF199.	X		X
IPI00604620	NCL	ISOFORM 1 OF NUCLEOLIN.	X		X
IPI00641214	ADCY10	177 KDA PROTEIN.	X		X
IPI00644752	LOC643669	HYPOTHETICAL PROTEIN ISOFORM 1.	X		X
IPI00655865	DIAPH3	ISOFORM 2 OF PROTEIN DIAPHANOUS HOMOLOG 3.	X		X

Table S1. Proteins Identified or Not in MS Patients Compared to Normals and Other Neurologic Disease (OND)					
**IPI**	**Gene**	**Protein Name**	**MS**	**Normal**	**OND**
IPI00658109	CKMT1A; CKMT1B	CREATINE KINASE, UBIQUITOUS MITOCHONDRIAL PRECURSOR.	X		X
IPI00735531	SHANK3	PROLINE-RICH SYNAPSE-ASSOCIATED PROTEIN 2 ISOFORM 4.	X		X
IPI00737363	INTS1	SIMILAR TO CG3173-PA ISOFORM 7.	X		X
IPI00739423	LOC377711	SIMILAR TO C11.1 CG12132-PA ISOFORM 11.	X		X
IPI00742682	TPR	NUCLEAR PORE COMPLEX-ASSOCIATED PROTEIN TPR.	X		X
IPI00743696	COL4A1	UNCHARACTERIZED PROTEIN COL4A1.	X		X
IPI00745775		SIMILAR TO PR DOMAIN CONTAINING 4.	X		X
IPI00760941	DKFZp434J1015	UNCHARACTERIZED PROTEIN ENSP00000371687.	X		X
IPI00782992	SRRM2	ISOFORM 1 OF SERINE/ARGININE REPETITIVE MATRIX PROTEIN 2.	X		X
IPI00783186	ANKRD17	ISOFORM 2 OF ANKYRIN REPEAT DOMAIN-CONTAINING PROTEIN 17.	X		X
IPI00783950	TTN	ISOFORM 6 OF TITIN.	X		X
IPI00784257	FOLR2	FOLATE RECEPTOR BETA PRECURSOR.	X		X
IPI00784385	ADCY1	ADENYLATE CYCLASE TYPE 1.	X		X
IPI00788118	LOC727942	SIMILAR TO PHOSPHODIESTERASE 4D INTERACTING PROTEIN ISOFORM 2.	X		X
IPI00792933	ZNF462	ZINC FINGER PROTEIN 462.	X		X
IPI00794779	POLQ	DNA POLYMERASE THETA.	X		X
IPI00815662		RHEUMATOID FACTOR G9 HEAVY CHAIN (FRAGMENT).	X		X
IPI00816409		V[GAMMA]1 PROTEIN (FRAGMENT).	X		X
IPI00827486		RHEUMATOID FACTOR LIGHT CHAIN VARIABLE REGION PRECURSOR (FRAGMENT).	X		X
IPI00827643		HRV FAB 027-VL (FRAGMENT).	X		X
IPI00827789		HRV FAB N6-VL (FRAGMENT).	X		X
IPI00827815		LIGHT CHAIN FAB (FRAGMENT).	X		X
IPI00829697		UNCHARACTERIZED PROTEIN ENSP00000375030.	X		X
IPI00829750		UNCHARACTERIZED PROTEIN ENSP00000375006.	X		X
IPI00829845		UNCHARACTERIZED PROTEIN ENSP00000375026.	X		X
IPI00829979		UNCHARACTERIZED PROTEIN ENSP00000375008.	X		X
IPI00854589		UNCHARACTERIZED PROTEIN ENSP00000375004.	X		X
IPI00876962	INF2	ISOFORM 2 OF INVERTED FORMIN-2.	X		X
IPI00879915	C6	15 KDA PROTEIN.	X		X
IPI00880011		116 KDA PROTEIN.	X		X
IPI00884022		SIMILAR TO IMMUNGLOBULIN HEAVY CHAIN VARIABLE REGION.	X		X
IPI00000013	CTSL2	CATHEPSIN L2 PRECURSOR.	X		

Table S1. Proteins Identified or Not in MS Patients Compared to Normals and Other Neurologic Disease (OND)					
**IPI**	**Gene**	**Protein Name**	**MS**	**Normal**	**OND**
IPI00000033	MLLT3	PROTEIN AF-9.	X		
IPI00000655	SLC16A2	SOLUTE CARRIER FAMILY 16, MEMBER 2.	X		
IPI00000728	USP15	ISOFORM 1 OF UBIQUITIN CARBOXYL-TERMINAL HYDROLASE 15.	X		
IPI00000769	KIF22	KINESIN-LIKE PROTEIN KIF22.	X		
IPI00000868	MATK	MEGAKARYOCYTE-ASSOCIATED TYROSINE-PROTEIN KINASE.	X		
IPI00000870	CGB; CGB5; CGB7; CGB8	CHORIOGONADOTROPIN SUBUNIT BETA PRECURSOR.	X		
IPI00000924	MUC5B	MUCIN (FRAGMENT).	X		
IPI00001062	MYD88	MYELOID DIFFERENTIATION PRIMARY RESPONSE PROTEIN MYD88.	X		
IPI00001159	GCN1L1	GCN1-LIKE PROTEIN 1.	X		
IPI00001382	TAP2	TRANSPORTER 2, ATP-BINDING CASSETTE, SUB-FAMILY B ISOFORM 2.	X		
IPI00001458	KNTC1	KINETOCHORE-ASSOCIATED PROTEIN 1.	X		
IPI00001525	ZNF169	ZINC FINGER PROTEIN 169.	X		
IPI00001557	SLC9A5	SODIUM/HYDROGEN EXCHANGER 5.	X		
IPI00001562	PCYT1B	ISOFORM 2 OF CHOLINE-PHOSPHATE CYTIDYLYLTRANSFERASE B.	X		
IPI00001729	MMP24	MATRIX METALLOPROTEINASE-24 PRECURSOR.	X		
IPI00001759	OLR1	OXIDIZED LOW-DENSITY LIPOPROTEIN RECEPTOR 1.	X		
IPI00001891	AUP1	ISOFORM LONG OF ANCIENT UBIQUITOUS PROTEIN 1 PRECURSOR.	X		
IPI00001955	MORF4	TRANSCRIPTION FACTOR-LIKE PROTEIN MORF4.	X		
IPI00002089	CDC25B	CDC25B PHOSPHATASE.	X		
IPI00002144	MARCH4	E3 UBIQUITIN-PROTEIN LIGASE MARCH4 PRECURSOR.	X		
IPI00002224	ADIPOR2	ADIPONECTIN RECEPTOR PROTEIN 2.	X		
IPI00002252	CD68	ISOFORM LONG OF MACROSIALIN PRECURSOR.	X		
IPI00002255	LRBA	LIPOPOLYSACCHARIDE-RESPONSIVE AND BEIGE-LIKE ANCHOR PROTEIN.	X		
IPI00002286	ANKRD11	ANKYRIN REPEAT DOMAIN-CONTAINING PROTEIN 11.	X		
IPI00002335	HTT	HUNTINGTIN.	X		
IPI00002435	CD27	CD27 ANTIGEN PRECURSOR.	X		
IPI00002483	SLC30A1	ZINC TRANSPORTER 1.	X		
IPI00002519	SHMT1	ISOFORM 1 OF SERINE HYDROXYMETHYLTRANSFERASE, CYTOSOLIC.	X		
IPI00002580	PIK3C2A	PHOSPHATIDYLINOSITOL-4-PHOSPHATE 3-KINASE C2 DOMAIN-CONTAINING ALPHA POLYPEPTIDE.	X		
IPI00002831	SAP30L	HISTONE DEACETYLASE COMPLEX SUBUNIT SAP30L.	X		
IPI00003369	LIMD1	LIM DOMAIN-CONTAINING PROTEIN 1.	X		
IPI00003431	MAPK6	MITOGEN-ACTIVATED PROTEIN KINASE 6.	X		

Table S1. Proteins Identified or Not in MS Patients Compared to Normals and Other Neurologic Disease (OND)					
IPI	Gene	Protein Name	MS	Normal	OND
IPI00003448	MIA	MELANOMA-DERIVED GROWTH REGULATORY PROTEIN PRECURSOR.	X		
IPI00003451	CYP1B1	CYTOCHROME P450 1B1.	X		
IPI00003467	GABRB3	ISOFORM 1 OF GAMMA-AMINOBUTYRIC ACID RECEPTOR SUBUNIT BETA-3 PRECURSOR.	X		
IPI00003495	MFHAS1	MALIGNANT FIBROUS HISTIOCYTOMA-AMPLIFIED SEQUENCE 1.	X		
IPI00003813	CADM1	ISOFORM 1 OF CELL ADHESION MOLECULE 1 PRECURSOR.	X		
IPI00003824	PPIL2	ISOFORM 1 OF PEPTIDYL-PROLYL CIS-TRANS ISOMERASE-LIKE 2.	X		
IPI00003888	PCDHGA7	ISOFORM 1 OF PROTOCADHERIN GAMMA A7 PRECURSOR.	X		
IPI00003964	USP9X	UBIQUITIN SPECIFIC PROTEASE 9, X-LINKED ISOFORM 4.	X		
IPI00004233	MKI67	ISOFORM LONG OF ANTIGEN KI-67.	X		
IPI00004334	GPR3	PROBABLE G-PROTEIN COUPLED RECEPTOR 3.	X		
IPI00004425	C15orf2	PROTEIN C15ORF2.	X		
IPI00004550	KRT24	KERATIN, TYPE I CYTOSKELETAL 24.	X		
IPI00004551	NLRP1	ISOFORM 4 OF NACHT, LRR AND PYD DOMAINS-CONTAINING PROTEIN 1.	X		
IPI00004933	KCNK12	POTASSIUM CHANNEL SUBFAMILY K MEMBER 12.	X		
IPI00005039	MTIF2	TRANSLATION INITIATION FACTOR IF-2, MITOCHONDRIAL PRECURSOR.	X		
IPI00005120	MAGEL2	MAGE-LIKE PROTEIN 2.	X		
IPI00005154	SSRP1	FACT COMPLEX SUBUNIT SSRP1.	X		
IPI00005401	GALNT5	POLYPEPTIDE N-ACETYLGALACTOSAMINYLTRANSFERASE 5.	X		
IPI00005491	CLSTN2	CALSYNTENIN-2 PRECURSOR.	X		
IPI00005565	DGKQ	DIACYLGLYCEROL KINASE THETA.	X		
IPI00005592	PDE1B	CALCIUM/CALMODULIN-DEPENDENT 3',5'-CYCLIC NUCLEOTIDE PHOSPHODIESTERASE 1B.	X		
IPI00005666	JMJD2A	JMJC DOMAIN-CONTAINING HISTONE DEMETHYLATION PROTEIN 3A.	X		
IPI00005731	BMPR1A	BONE MORPHOGENETIC PROTEIN RECEPTOR TYPE IA PRECURSOR.	X		
IPI00005750	PKDREJ	PKDREJ (FRAGMENT).	X		
IPI00005792	PABPN1	ISOFORM 1 OF POLYADENYLATE-BINDING PROTEIN 2.	X		
IPI00005811	MLH3	ISOFORM 1 OF DNA MISMATCH REPAIR PROTEIN MLH3.	X		
IPI00005904	DDX20	PROBABLE ATP-DEPENDENT RNA HELICASE DDX20.	X		
IPI00006099	BMS1	RIBOSOME BIOGENESIS PROTEIN BMS1 HOMOLOG.	X		
IPI00006180	SOX12	SOX-12 PROTEIN.	X		
IPI00006204	KCNAB3	VOLTAGE-GATED POTASSIUM CHANNEL SUBUNIT BETA-3.	X		
IPI00006217	GRIN2D	GLUTAMATE [NMDA] RECEPTOR SUBUNIT EPSILON-4 PRECURSOR.	X		
IPI00006288	SLIT2	ISOFORM 1 OF SLIT HOMOLOG 2 PROTEIN PRECURSOR.	X		

Table S1. Proteins Identified or Not in MS Patients Compared to Normals and Other Neurologic Disease (OND)					
**IPI**	**Gene**	**Protein Name**	**MS**	**Normal**	**OND**
IPI00006560	SERPINB13	ISOFORM 1 OF SERPIN B13.	X		
IPI00006725	DDX23	PROBABLE ATP-DEPENDENT RNA HELICASE DDX23.	X		
IPI00006735	ECEL1	ISOFORM 1 OF ENDOTHELIN-CONVERTING ENZYME-LIKE 1.	X		
IPI00006892	ZNF280C	PUTATIVE UNCHARACTERIZED PROTEIN.	X		
IPI00006982	XAGE1; XAGE1; XAGE1B; XAGE1C; XAGE1D; XAGE1E	X ANTIGEN FAMILY, MEMBER 1 ISOFORM 2.	X		
IPI00007122	KIAA1009	ISOFORM 1 OF PROTEIN QN1 HOMOLOG.	X		
IPI00007165	DOLK	TRANSMEMBRANE PROTEIN 15.	X		
IPI00007266	ALAS1	ISOFORM 1 OF 5-AMINOLEVULINATE SYNTHASE, NONSPECIFIC, MITOCHONDRIAL PRECURSOR.	X		
IPI00007317	MTMR15	ISOFORM 1 OF COILED-COIL DOMAIN-CONTAINING PROTEIN MTMR15.	X		
IPI00007395	MTL5	METALLOTHIONEIN-LIKE 5, TESTIS-SPECIFIC ISOFORM A.	X		
IPI00007427	AGR2	AGR2.	X		
IPI00007515	ADAM30	ISOFORM ALPHA OF ADAM 30 PRECURSOR.	X		
IPI00007641	IRAK4	INTERLEUKIN-1 RECEPTOR-ASSOCIATED KINASE 4.	X		
IPI00007695	SHANK1	ISOFORM 1 OF SH3 AND MULTIPLE ANKYRIN REPEAT DOMAINS PROTEIN 1.	X		
IPI00007806	STOML1	ISOFORM 1 OF STOMATIN-LIKE PROTEIN 1.	X		
IPI00007821	ZNF215	ZINC FINGER PROTEIN 215.	X		
IPI00007906		MYOSIN-REACTIVE IMMUNOGLOBULIN HEAVY CHAIN VARIABLE REGION (FRAGMENT).	X		
IPI00007979	ND2	NADH-UBIQUINONE OXIDOREDUCTASE CHAIN 2.	X		
IPI00008054	BRPF3	BROMODOMAIN AND PHD FINGER-CONTAINING PROTEIN 3.	X		
IPI00008192	DLX5	HOMEOBOX PROTEIN DLX-5.	X		
IPI00008200	YEATS2	ISOFORM 1 OF YEATS DOMAIN-CONTAINING PROTEIN 2.	X		
IPI00008255	AZI2	ISOFORM 1 OF 5-AZACYTIDINE-INDUCED PROTEIN 2.	X		
IPI00008416	C1orf89	MIRO DOMAIN-CONTAINING PROTEIN C1ORF89.	X		
IPI00008486	PADI3	PROTEIN-ARGININE DEIMINASE TYPE-3.	X		
IPI00008542	FBXO28	F-BOX ONLY PROTEIN 28.	X		
IPI00008790	GALC	GALACTOSYLCERAMIDASE ISOFORM A PRECURSOR.	X		
IPI00008982	ALDH18A1	ISOFORM LONG OF DELTA-1-PYRROLINE-5-CARBOXYLATE SYNTHETASE.	X		

Table S1. Proteins Identified or Not in MS Patients Compared to Normals and Other Neurologic Disease (OND)					
**IPI**	**Gene**	**Protein Name**	**MS**	**Normal**	**OND**
IPI00008993	TRAT1	T-CELL RECEPTOR-ASSOCIATED TRANSMEMBRANE ADAPTER 1.	X		
IPI00009126	ACTL7B	ACTIN-LIKE PROTEIN 7B.	X		
IPI00009346	TMEM14C	TRANSMEMBRANE PROTEIN 14C.	X		
IPI00009379	IGHMBP2	DNA-BINDING PROTEIN SMUBP-2.	X		
IPI00009448	TNFAIP3	TUMOR NECROSIS FACTOR, ALPHA-INDUCED PROTEIN 3.	X		
IPI00009450	KITLG	ISOFORM 1 OF KIT LIGAND PRECURSOR.	X		
IPI00009707	RMND5A	ISOFORM 1 OF PROTEIN RMD5 HOMOLOG A.	X		
IPI00009713	SOX9	TRANSCRIPTION FACTOR SOX-9.	X		
IPI00009775	NINJ2	NINJURIN-2.	X		
IPI00009803	ITGA4	INTEGRIN ALPHA-4 PRECURSOR.	X		
IPI00009852	ATP6V0A4	VACUOLAR PROTON TRANSLOCATING ATPASE 116 KDA SUBUNIT A ISOFORM 4.	X		
IPI00009859	THEG	ISOFORM 1 OF TESTICULAR HAPLOID EXPRESSED GENE PROTEIN.	X		
IPI00009866	KRT13	ISOFORM 1 OF KERATIN, TYPE I CYTOSKELETAL 13.	X		
IPI00009923	P4HA1	ISOFORM 1 OF PROLYL 4-HYDROXYLASE SUBUNIT ALPHA-1 PRECURSOR.	X		
IPI00010099	IL1RAPL2	X-LINKED INTERLEUKIN-1 RECEPTOR ACCESSORY PROTEIN-LIKE 2 PRECURSOR.	X		
IPI00010172	GIPR	ISOFORM SHORT OF GASTRIC INHIBITORY POLYPEPTIDE RECEPTOR PRECURSOR.	X		
IPI00010178	TH	ISOFORM 3 OF TYROSINE 3-MONOOXYGENASE.	X		
IPI00010196	NRIP1	NUCLEAR RECEPTOR-INTERACTING PROTEIN 1.	X		
IPI00010232	PEX3	PEROXISOMAL BIOGENESIS FACTOR 3.	X		
IPI00010244	MRPS11	ISOFORM 1 OF 28S RIBOSOMAL PROTEIN S11, MITOCHONDRIAL PRECURSOR.	X		
IPI00010257	ERAF	ALPHA-HEMOGLOBIN-STABILIZING PROTEIN.	X		
IPI00010289	DRD1	D(1A) DOPAMINE RECEPTOR.	X		
IPI00010322	PDZRN3	ISOFORM 1 OF PDZ DOMAIN-CONTAINING RING FINGER PROTEIN 3.	X		
IPI00010338	F3	TISSUE FACTOR PRECURSOR.	X		
IPI00010418	MYO1C	MYOSIN-IC.	X		
IPI00010486	HPS5	ISOFORM 1 OF HERMANSKY-PUDLAK SYNDROME 5 PROTEIN.	X		
IPI00010550	FOXL2	TRANSCRIPTION FACTOR FOXL2 MUTANT 1.	X		
IPI00010720	CCT5	T-COMPLEX PROTEIN 1 SUBUNIT EPSILON.	X		
IPI00010754	HOXA11	HOMEOBOX PROTEIN HOX-A11.	X		
IPI00010847	CRYBA1	ISOFORM A3 OF BETA-CRYSTALLIN A3.	X		
IPI00010884	LOC390688	CDC37-LIKE GENE.	X		
IPI00011031	WNT7B	PROTEIN WNT-7B PRECURSOR.	X		
IPI00011197	SNX25	SORTING NEXIN 25.	X		
IPI00011473	TRIM55	ISOFORM 3 OF TRIPARTITE MOTIF-CONTAINING PROTEIN 55.	X		

Table S1. Proteins Identified or Not in MS Patients Compared to Normals and Other Neurologic Disease (OND)					
**IPI**	**Gene**	**Protein Name**	**MS**	**Normal**	**OND**
IPI00011547	NAIP	BACULOVIRAL IAP REPEAT-CONTAINING PROTEIN 1.	X		
IPI00011635	BCL2L13	ISOFORM 2 OF BCL-2-LIKE 13 PROTEIN.	X		
IPI00011650	TREH	TREHALASE PRECURSOR.	X		
IPI00011692	IVL	INVOLUCRIN.	X		
IPI00011694	PRSS1	TRYPSIN-1 PRECURSOR.	X		
IPI00011919	FADD	PROTEIN FADD.	X		
IPI00011938	ADCY6	ISOFORM 1 OF ADENYLATE CYCLASE TYPE 6.	X		
IPI00012069	NQO1	NAD.	X		
IPI00012093	STK25	SERINE/THREONINE-PROTEIN KINASE 25.	X		
IPI00012119	DCN	ISOFORM A OF DECORIN PRECURSOR.	X		
IPI00012213	CARD14	CASPASE RECRUITMENT DOMAIN-CONTAINING PROTEIN 14.	X		
IPI00012345	SFRS6	ISOFORM SRP55-1 OF SPLICING FACTOR, ARGININE/SERINE-RICH 6.	X		
IPI00012347	MXRA5	MATRIX-REMODELING-ASSOCIATED PROTEIN 5 PRECURSOR.	X		
IPI00012426	SCPEP1	ISOFORM 1 OF RETINOID-INDUCIBLE SERINE CARBOXYPEPTIDASE PRECURSOR.	X		
IPI00012487	FPRL1	FMLP-RELATED RECEPTOR I.	X		
IPI00012512	RRAS2	RAS-RELATED PROTEIN R-RAS2 PRECURSOR.	X		
IPI00012645	SPTBN2	ISOFORM 1 OF SPECTRIN BETA CHAIN, BRAIN 2.	X		
IPI00012796	GAD2	GLUTAMATE DECARBOXYLASE 2.	X		
IPI00012918	ARID5A	ISOFORM 1 OF AT-RICH INTERACTIVE DOMAIN-CONTAINING PROTEIN 5A.	X		
IPI00012977	BTN3A3	BUTYROPHILIN SUBFAMILY 3 MEMBER A3 PRECURSOR.	X		
IPI00013193	MYO7A	ISOFORM 1 OF MYOSIN-VIIA.	X		
IPI00013210	BTN1A1	BUTYROPHILIN SUBFAMILY 1 MEMBER A1 PRECURSOR.	X		
IPI00013384	KSR1	ISOFORM 2 OF KINASE SUPPRESSOR OF RAS 1.	X		
IPI00013441	SUV420H2	ISOFORM 1 OF HISTONE-LYSINE N-METHYLTRANSFERASE SUV420H2.	X		
IPI00013623	SLC27A3	ISOFORM 1 OF LONG-CHAIN FATTY ACID TRANSPORT PROTEIN 3.	X		
IPI00013717	AQP6	AQUAPORIN-6.	X		
IPI00013885	CASP14	CASPASE-14 PRECURSOR.	X		
IPI00013983	RET	PROTO-ONCOGENE TYROSINE-PROTEIN KINASE RECEPTOR RET PRECURSOR.	X		
IPI00014068	PAK4	ISOFORM 1 OF SERINE/THREONINE-PROTEIN KINASE PAK 4.	X		
IPI00014168	HSAJ2425	PROTEIN P65.	X		
IPI00014482	ALKBH1	ALKYLATED DNA REPAIR PROTEIN ALKB HOMOLOG 1.	X		
IPI00014540	HOXB4	HOMEOBOX PROTEIN HOX-B4.	X		
IPI00014757	NKAPL	NKAP-LIKE PROTEIN.	X		
IPI00014874	ZNF562	ISOFORM 1 OF ZINC FINGER PROTEIN 562.	X		

Table S1. Proteins Identified or Not in MS Patients Compared to Normals and Other Neurologic Disease (OND)					
**IPI**	**Gene**	**Protein Name**	**MS**	**Normal**	**OND**
IPI00014939	MKKS	MCKUSICK-KAUFMAN/BARDET-BIEDL SYNDROMES PUTATIVE CHAPERONIN.	X		
IPI00015134	HTR2B	5-HYDROXYTRYPTAMINE RECEPTOR 2B.	X		
IPI00015137	CCR2; FLJ78302	ISOFORM A OF C-C CHEMOKINE RECEPTOR TYPE 2.	X		
IPI00015170		PUTATIVE UNCHARACTERIZED PROTEIN (FRAGMENT).	X		
IPI00015618	CCDC49	ISOFORM 1 OF COILED-COIL DOMAIN-CONTAINING PROTEIN 49.	X		
IPI00015785	CRB1	ISOFORM 2 OF CRUMBS HOMOLOG 1 PRECURSOR.	X		
IPI00015869	TCHH	TRICHOHYALIN.	X		
IPI00015892	CCRK	CELL CYCLE RELATED KINASE.	X		
IPI00016066	LOC729608	SIMILAR TO PUTATIVE BRIX DOMAIN CONTAINING PROTEIN 1P.	X		
IPI00016067	MMP19	ISOFORM 1 OF MATRIX METALLOPROTEINASE-19 PRECURSOR.	X		
IPI00016121	CXorf27	HUNTINGTIN-INTERACTING PROTEIN HYPM.	X		
IPI00016387	PCF11	PRE-MRNA CLEAVAGE COMPLEX 2 PROTEIN PCF11.	X		
IPI00016472	ZC3H13	ISOFORM 2 OF ZINC FINGER CCCH DOMAIN-CONTAINING PROTEIN 13.	X		
IPI00016475	GOLGA8A	ISOFORM 1 OF GOLGIN SUBFAMILY A MEMBER 8A.	X		
IPI00016478	C1orf109	ISOFORM 1 OF UNCHARACTERIZED PROTEIN C1ORF109.	X		
IPI00016481	TXNL4B	THIOREDOXIN-LIKE PROTEIN 4B.	X		
IPI00016536	PIGF	ISOFORM 1 OF PHOSPHATIDYLINOSITOL-GLYCAN BIOSYNTHESIS CLASS F PROTEIN.	X		
IPI00016559	THG1L	PROBABLE TRNA(HIS) GUANYLYLTRANSFERASE.	X		
IPI00016574		CDNA FLJ13262 FIS, CLONE OVARC1000912.	X		
IPI00016637	OXSM	3-OXOACYL-[ACYL-CARRIER-PROTEIN] SYNTHASE, MITOCHONDRIAL PRECURSOR.	X		
IPI00016827	SLC27A5	BILE ACYL-COA SYNTHETASE.	X		
IPI00017227	UBTD1	UBIQUITIN DOMAIN-CONTAINING PROTEIN 1.	X		
IPI00017303	MSH2	DNA MISMATCH REPAIR PROTEIN MSH2.	X		
IPI00017386	C1orf107	CDNA FLJ12704 FIS, CLONE NT2RP1000836.	X		
IPI00017643	BANP	PUTATIVE UNCHARACTERIZED PROTEIN DKFZP761H172.	X		
IPI00017878	RP4-692D3.1	CDNA: FLJ20972 FIS, CLONE ADSU01569.	X		
IPI00017921	BICC1	ISOFORM 2 OF PROTEIN BICAUDAL C HOMOLOG 1.	X		
IPI00017972	ZNF703	ISOFORM 1 OF ZINC FINGER PROTEIN 703.	X		
IPI00018009	EDC3	ENHANCER OF MRNA-DECAPPING PROTEIN 3.	X		
IPI00018019	GPATCH2	ISOFORM 1 OF G PATCH DOMAIN-CONTAINING PROTEIN 2.	X		
IPI00018110	TAF11	TRANSCRIPTION INITIATION FACTOR TFIID SUBUNIT 11.	X		
IPI00018198	CEP27	ISOFORM 1 OF CENTROSOMAL PROTEIN OF 27 KDA.	X		
IPI00018258	SPAG4	SPERM-ASSOCIATED ANTIGEN 4 PROTEIN.	X		

Table S1. Proteins Identified or Not in MS Patients Compared to Normals and Other Neurologic Disease (OND)					
**IPI**	**Gene**	**Protein Name**	**MS**	**Normal**	**OND**
IPI00018288	POLR2C	DNA-DIRECTED RNA POLYMERASE II SUBUNIT RPB3.	X		
IPI00018320	PCDH11Y	ISOFORM 2 OF PROTOCADHERIN-11 Y-LINKED PRECURSOR.	X		
IPI00018370	SVIL	ISOFORM 2 OF SUPERVILLIN.	X		
IPI00018471	SMO	SMOOTHENED HOMOLOG PRECURSOR.	X		
IPI00018776	ZNF445	TRANSCRIPTION FACTOR (FRAGMENT).	X		
IPI00018829	SPTBN4	ISOFORM 1 OF SPECTRIN BETA CHAIN, BRAIN 3.	X		
IPI00018934	SCN1A	ISOFORM 1 OF SODIUM CHANNEL PROTEIN TYPE 1 SUBUNIT ALPHA.	X		
IPI00019226	BRD8	ISOFORM 1 OF BROMODOMAIN-CONTAINING PROTEIN 8.	X		
IPI00019350	STEAP3	ISOFORM 1 OF METALLOREDUCTASE STEAP3.	X		
IPI00019459	MAP3K7IP1	MITOGEN-ACTIVATED PROTEIN KINASE KINASE KINASE 7-INTERACTING PROTEIN 1.	X		
IPI00019505	COX8A	CYTOCHROME C OXIDASE POLYPEPTIDE VIII-LIVER/HEART, MITOCHONDRIAL PRECURSOR.	X		
IPI00019871	HSD17B3	TESTOSTERONE 17-BETA-DEHYDROGENASE 3.	X		
IPI00019977	GTF2E1	TRANSCRIPTION INITIATION FACTOR IIE SUBUNIT ALPHA.	X		
IPI00020037	USF2	ISOFORM USF2A OF UPSTREAM STIMULATORY FACTOR 2.	X		
IPI00020078	HNF1B	ISOFORM A OF HEPATOCYTE NUCLEAR FACTOR 1-BETA.	X		
IPI00020134	SOS2	SON OF SEVENLESS HOMOLOG 2.	X		
IPI00020153	BSN	PROTEIN BASSOON.	X		
IPI00020416	TPP2	TRIPEPTIDYL-PEPTIDASE 2.	X		
IPI00020443	DOK5	ISOFORM 1 OF DOCKING PROTEIN 5.	X		
IPI00020513	ZYX	ZYXIN.	X		
IPI00020918	AFF3	AF4/FMR2 FAMILY, MEMBER 3 ISOFORM 1.	X		
IPI00020942	GUCY2F	RETINAL GUANYLYL CYCLASE 2 PRECURSOR.	X		
IPI00021258	ARFIP1	ISOFORM B OF ARFAPTIN-1.	X		
IPI00021300	TAS2R1	TASTE RECEPTOR TYPE 2 MEMBER 1.	X		
IPI00021358	TACR3	NEUROMEDIN-K RECEPTOR.	X		
IPI00021396	KDR	VASCULAR ENDOTHELIAL GROWTH FACTOR RECEPTOR 2 PRECURSOR.	X		
IPI00021399	FGFBP1	FIBROBLAST GROWTH FACTOR-BINDING PROTEIN 1 PRECURSOR.	X		
IPI00021416	KATNAL2	40 KDA PROTEIN.	X		
IPI00021449	ITPKB	ISOFORM 1 OF INOSITOL-TRISPHOSPHATE 3-KINASE B.	X		
IPI00021518	DDB2	ISOFORM 1 OF DNA DAMAGE-BINDING PROTEIN 2.	X		
IPI00021569	MBTPS1	MEMBRANE-BOUND TRANSCRIPTION FACTOR SITE-1 PROTEASE PRECURSOR.	X		
IPI00021578	CFHR4	COMPLEMENT FACTOR H-RELATED PROTEIN 4 PRECURSOR.	X		
IPI00021671	KIAA0922	HYPOTHETICAL PROTEIN LOC23240.	X		

Table S1. Proteins Identified or Not in MS Patients Compared to Normals and Other Neurologic Disease (OND)					
IPI	Gene	Protein Name	MS	Normal	OND
IPI00021780	TGFB3	TRANSFORMING GROWTH FACTOR BETA-3 PRECURSOR.	X		
IPI00022042	KIAA0241	ISOFORM 2 OF PROTEIN KIAA0241.	X		
IPI00022204	SERPINB3	SERPIN B3.	X		
IPI00022300	METTL7A	METHYLTRANSFERASE-LIKE PROTEIN 7A PRECURSOR.	X		
IPI00022355	OR2D2	OLFACTORY RECEPTOR 2D2.	X		
IPI00022402	FAM32A	PROTEIN FAM32A.	X		
IPI00022418	FN1	ISOFORM 1 OF FIBRONECTIN PRECURSOR.	X		
IPI00022430	GAPDHS	GLYCERALDEHYDE-3-PHOSPHATE DEHYDROGENASE, TESTIS-SPECIFIC.	X		
IPI00022443	AFP	ALPHA-FETOPROTEIN PRECURSOR.	X		
IPI00022449	DOCK2	ISOFORM 1 OF DEDICATOR OF CYTOKINESIS PROTEIN 2.	X		
IPI00022450	TBC1D5	TBC1 DOMAIN FAMILY MEMBER 5.	X		
IPI00022465	CIT	ISOFORM 1 OF CITRON RHO-INTERACTING KINASE.	X		
IPI00022536	RPS6KA4	ISOFORM 1 OF RIBOSOMAL PROTEIN S6 KINASE ALPHA-4.	X		
IPI00022557	F2RL3	PROTEINASE-ACTIVATED RECEPTOR 4 PRECURSOR.	X		
IPI00022560	CH25H	CHOLESTEROL 25-HYDROXYLASE.	X		
IPI00022620	SLURP1	SECRETED LY-6/UPAR-RELATED PROTEIN 1 PRECURSOR.	X		
IPI00022633	TNK1	ISOFORM 1 OF NON-RECEPTOR TYROSINE-PROTEIN KINASE TNK1.	X		
IPI00022793	HADHB	TRIFUNCTIONAL ENZYME SUBUNIT BETA, MITOCHONDRIAL PRECURSOR.	X		
IPI00022850	GRIK1	ISOFORM 1 OF GLUTAMATE RECEPTOR, IONOTROPIC KAINATE 1 PRECURSOR.	X		
IPI00022865	CCNA2	CYCLIN-A2.	X		
IPI00022883	AP3M2	AP-3 COMPLEX SUBUNIT MU-2.	X		
IPI00022933	CD74	ISOFORM LONG OF HLA CLASS II HISTOCOMPATIBILITY ANTIGEN GAMMA CHAIN.	X		
IPI00022975	ALOX5AP	ARACHIDONATE 5-LIPOXYGENASE-ACTIVATING PROTEIN.	X		
IPI00023035	SLC20A1	SODIUM-DEPENDENT PHOSPHATE TRANSPORTER 1.	X		
IPI00023186	TOM1L1	TOM1-LIKE PROTEIN 1.	X		
IPI00023211	RND1	RHO-RELATED GTP-BINDING PROTEIN RHO6 PRECURSOR.	X		
IPI00023299	DDX12	PROBABLE ATP-DEPENDENT RNA HELICASE DDX12.	X		
IPI00023339	CREBBP	CREB-BINDING PROTEIN.	X		
IPI00023502	FCGR1A	ISOFORM A OF HIGH AFFINITY IMMUNOGLOBULIN GAMMA FC RECEPTOR I PRECURSOR.	X		
IPI00023527	CDKL1	CYCLIN-DEPENDENT KINASE-LIKE 1.	X		
IPI00023647	UBA6	ISOFORM 1 OF UBIQUITIN-LIKE MODIFIER-ACTIVATING ENZYME 6.	X		
IPI00023736	CORO2A	CORONIN-2A.	X		
IPI00023977	TTC3	ISOFORM TRPDI OF TETRATRICOPEPTIDE REPEAT PROTEIN 3.	X		

Table S1. Proteins Identified or Not in MS Patients Compared to Normals and Other Neurologic Disease (OND)					
**IPI**	**Gene**	**Protein Name**	**MS**	**Normal**	**OND**
IPI00024007	SMTN	ISOFORM B OF SMOOTHELIN.	X		
IPI00024031	SLC2A9	SOLUTE CARRIER FAMILY 2, FACILITATED GLUCOSE TRANSPORTER MEMBER 9.	X		
IPI00024097	TES	ISOFORM 1 OF TESTIN.	X		
IPI00024291	BAD	BCL2 ANTAGONIST OF CELL DEATH.	X		
IPI00024568	GLTSCR1	GLIOMA TUMOR SUPPRESSOR CANDIDATE REGION GENE 1.	X		
IPI00024645	NT5M	MITOCHONDRIAL 5' NUCLEOTIDASE.	X		
IPI00024787	SLC27A2	VERY LONG-CHAIN ACYL-COA SYNTHETASE.	X		
IPI00024811	MPZL2	MYELIN PROTEIN ZERO-LIKE PROTEIN 2 PRECURSOR.	X		
IPI00024856	KIAA1622	ISOFORM 2 OF UNCHARACTERIZED PROTEIN KIAA1622.	X		
IPI00024880	SCUBE2	ISOFORM 1 OF SIGNAL PEPTIDE, CUB AND EGF-LIKE DOMAIN-CONTAINING PROTEIN 2 PRECURSOR.	X		
IPI00024900	SBNO2	STRAWBERRY NOTCH HOMOLOG 2 ISOFORM 2.	X		
IPI00024970	INCENP	INNER CENTROMERE PROTEIN.	X		
IPI00024990	ALDH6A1	METHYLMALONATE-SEMIALDEHYDE DEHYDROGENASE [ACYLATING], MITOCHONDRIAL PRECURSOR.	X		
IPI00025255	SNN	STANNIN.	X		
IPI00025260	SEC14L3	SEC14-LIKE PROTEIN 3.	X		
IPI00025323	MAP9	ISOFORM 1 OF MICROTUBULE-ASSOCIATED PROTEIN 9.	X		
IPI00025418	COL7A1	ISOFORM 1 OF COLLAGEN ALPHA-1(VII) CHAIN PRECURSOR.	X		
IPI00025427	RNASE3	EOSINOPHIL CATIONIC PROTEIN PRECURSOR.	X		
IPI00025428		57 KDA PROTEIN.	X		
IPI00025683	TACC1	ISOFORM 1 OF TRANSFORMING ACIDIC COILED-COIL-CONTAINING PROTEIN 1.	X		
IPI00025792	SUPT3H	ISOFORM 1 OF TRANSCRIPTION INITIATION PROTEIN SPT3 HOMOLOG.	X		
IPI00026133	PTH2R	PARATHYROID HORMONE 2 RECEPTOR PRECURSOR.	X		
IPI00026189	LOC56251	H_YH95C04.1 PROTEIN (FRAGMENT).	X		
IPI00026233	CCDC71	COILED-COIL DOMAIN-CONTAINING PROTEIN 71.	X		
IPI00026256	FLG	FILAGGRIN.	X		
IPI00026544	GBP3	ISOFORM 1 OF GUANYLATE-BINDING PROTEIN 3.	X		
IPI00026638	SYT13	SYNAPTOTAGMIN-13.	X		
IPI00026650	HLA-A; HLA-A29.1; HLA-B; HLA-C; LOC730410; MICA	HLA CLASS I HISTOCOMPATIBILITY ANTIGEN, CW-1 ALPHA CHAIN PRECURSOR.	X		

Table S1. Proteins Identified or Not in MS Patients Compared to Normals and Other Neurologic Disease (OND)					
**IPI**	**Gene**	**Protein Name**	**MS**	**Normal**	**OND**
IPI00026813	FNTA	PROTEIN FARNESYLTRANSFERASE/GERANYLGERANYLTRANSFERASE TYPE-1 SUBUNIT ALPHA.	X		
IPI00026846	SLC6A1	SODIUM- AND CHLORIDE-DEPENDENT GABA TRANSPORTER 1.	X		
IPI00026926	GUCA2A	GUANYLIN PRECURSOR.	X		
IPI00026973	FZD10	FRIZZLED-10 PRECURSOR.	X		
IPI00027035	XAB1	XPA-BINDING PROTEIN 1.	X		
IPI00027107	TUFM	TU TRANSLATION ELONGATION FACTOR, MITOCHONDRIAL.	X		
IPI00027146	GLUD2	GLUTAMATE DEHYDROGENASE 2, MITOCHONDRIAL PRECURSOR.	X		
IPI00027233	SCO1	SCO1 PROTEIN HOMOLOG, MITOCHONDRIAL PRECURSOR.	X		
IPI00027268	BRCA1	BREAST CANCER 1, EARLY ONSET ISOFORM BRCA1-DELTA9-11.	X		
IPI00027285	SNRPB	ISOFORM SM-B' OF SMALL NUCLEAR RIBONUCLEOPROTEIN-ASSOCIATED PROTEINS B AND B'.	X		
IPI00027412	CEACAM6	CARCINOEMBRYONIC ANTIGEN-RELATED CELL ADHESION MOLECULE 6 PRECURSOR.	X		
IPI00027508	IL1R1	INTERLEUKIN-1 RECEPTOR TYPE I PRECURSOR.	X		
IPI00027509	MMP9	MATRIX METALLOPROTEINASE-9 PRECURSOR.	X		
IPI00027725	CASP4	CASPASE-4 PRECURSOR.	X		
IPI00027800	CCDC3	COILED-COIL DOMAIN-CONTAINING PROTEIN 3 PRECURSOR.	X		
IPI00027804	SSTR1	SOMATOSTATIN RECEPTOR TYPE 1.	X		
IPI00027988	CTCF	TRANSCRIPTIONAL REPRESSOR CTCF.	X		
IPI00028152	POLD4	DNA POLYMERASE SUBUNIT DELTA-4.	X		
IPI00028229	UGT2A3	UDP-GLUCURONOSYLTRANSFERASE 2A3 PRECURSOR.	X		
IPI00028318	PHACTR1	ISOFORM 1 OF PHOSPHATASE AND ACTIN REGULATOR 1.	X		
IPI00028328	TAS2R7	TASTE RECEPTOR TYPE 2 MEMBER 7.	X		
IPI00028369	KIAA1715	ISOFORM 1 OF PROTEIN LUNAPARK.	X		
IPI00028449	HS6ST1	HEPARAN SULFATE 6-O-SULFOTRANSFERASE 1.	X		
IPI00029013	FAM53B	ISOFORM 1 OF PROTEIN FAM53B.	X		
IPI00029091		PUTATIVE NUCLEOSIDE DIPHOSPHATE KINASE.	X		
IPI00029278	ADRB1	BETA-1 ADRENERGIC RECEPTOR.	X		
IPI00029502	CACNA1B	CALCIUM CHANNEL ALPHA12.2 SUBUNIT (FRAGMENT).	X		
IPI00029620	OR1I1	OLFACTORY RECEPTOR 1I1.	X		
IPI00029778	TP53BP1	ISOFORM 1 OF TUMOR SUPPRESSOR P53-BINDING PROTEIN 1.	X		
IPI00029822	SMARCA4	SMARCA4 ISOFORM 2.	X		
IPI00030208	SLC10A2	ILEAL SODIUM/BILE ACID COTRANSPORTER.	X		

Table S1. Proteins Identified or Not in MS Patients Compared to Normals and Other Neurologic Disease (OND)					
**IPI**	**Gene**	**Protein Name**	**MS**	**Normal**	**OND**
IPI00030241	CD164	PUTATIVE MUCIN CORE PROTEIN 24 PRECURSOR.	X		
IPI00030282	BFSP1	ISOFORM 1 OF FILENSIN.	X		
IPI00030357	DHFR	DIHYDROFOLATE REDUCTASE.	X		
IPI00030415	SH3BP1	CDNA: FLJ21318 FIS, CLONE COL02295.	X		
IPI00030652	ZIC2	ZINC FINGER PROTEIN ZIC 2.	X		
IPI00030774	TBCD	ISOFORM 4 OF TUBULIN-SPECIFIC CHAPERONE D.	X		
IPI00030917	GRB14	GROWTH FACTOR RECEPTOR-BOUND PROTEIN 14.	X		
IPI00031015	SLBP	HISTONE RNA HAIRPIN-BINDING PROTEIN.	X		
IPI00031100	SYT3	SYNAPTOTAGMIN-3.	X		
IPI00031282	KIAA1683	ISOFORM 1 OF UNCHARACTERIZED PROTEIN KIAA1683.	X		
IPI00031388	PIK3CB	PHOSPHATIDYLINOSITOL-4,5-BISPHOSPHATE 3-KINASE CATALYTIC SUBUNIT BETA ISOFORM.	X		
IPI00031526	C19orf43	UNCHARACTERIZED PROTEIN C19ORF43.	X		
IPI00031545	ITPR2	ISOFORM LONG OF INOSITOL 1,4,5-TRISPHOSPHATE RECEPTOR TYPE 2.	X		
IPI00031608	SPATA5L1	SPERMATOGENESIS-ASSOCIATED PROTEIN 5-LIKE PROTEIN 1.	X		
IPI00031756	KCNK6	ISOFORM 1 OF POTASSIUM CHANNEL SUBFAMILY K MEMBER 6.	X		
IPI00032061	SIRPG	ISOFORM 1 OF SIGNAL REGULATORY PROTEIN GAMMA PRECURSOR.	X		
IPI00032138	POFUT2	ISOFORM A OF GDP-FUCOSE PROTEIN O-FUCOSYLTRANSFERASE 2 PRECURSOR.	X		
IPI00032140	SERPINH1	SERPIN H1 PRECURSOR.	X		
IPI00032195	HSPB2	HEAT-SHOCK PROTEIN BETA-2.	X		
IPI00032236	AMOTL2	ISOFORM 2 OF ANGIOMOTIN-LIKE PROTEIN 2.	X		
IPI00032325	CSTA	CYSTATIN-A.	X		
IPI00032402	ATP8A1	ISOFORM LONG OF PROBABLE PHOSPHOLIPID-TRANSPORTING ATPASE IA.	X		
IPI00032563	ZNF281	ZINC FINGER PROTEIN 281.	X		
IPI00032630	MTO1	ISOFORM 3 OF PROTEIN MTO1 HOMOLOG, MITOCHONDRIAL PRECURSOR.	X		
IPI00033009	RIMBP3B	SIMILAR TO PERIPHERAL-TYPE BENZODIAZEPINE RECEPTOR-ASSOCIATED PROTEIN 1.	X		
IPI00033023	KCNAB1	POTASSIUM VOLTAGE-GATED CHANNEL BETA SUBUNIT.	X		
IPI00033217	AASS	ALPHA-AMINOADIPIC SEMIALDEHYDE SYNTHASE, MITOCHONDRIAL PRECURSOR.	X		
IPI00033553	TMEM16B	TRANSMEMBRANE PROTEIN 16B.	X		
IPI00034010	RWDD1	RWD DOMAIN-CONTAINING PROTEIN 1.	X		
IPI00034099	RBM35B	ISOFORM 2 OF RNA-BINDING PROTEIN 35B.	X		
IPI00041868	GALNT14	ISOFORM 3 OF POLYPEPTIDE N-ACETYLGALACTOSAMINYLTRANSFERASE 14.	X		
IPI00042099	PYGO2	PYGOPUS HOMOLOG 2.	X		

Table S1. Proteins Identified or Not in MS Patients Compared to Normals and Other Neurologic Disease (OND)					
IPI	Gene	Protein Name	MS	Normal	OND
IPI00043430	TMTC1	ISOFORM 2 OF TRANSMEMBRANE AND TPR REPEAT-CONTAINING PROTEIN 1.	X		
IPI00043553	GPR63	PROBABLE G-PROTEIN COUPLED RECEPTOR 63.	X		
IPI00043613		CDNA FLJ31021 FIS, CLONE HLUNG2000412.	X		
IPI00043622	FRMD6	ISOFORM 1 OF FERM DOMAIN-CONTAINING PROTEIN 6.	X		
IPI00043735	ZNF599	ISOFORM 1 OF ZINC FINGER PROTEIN 599.	X		
IPI00043760	AIFM3	ISOFORM 1 OF APOPTOSIS-INDUCING FACTOR 3.	X		
IPI00043909		CDNA FLJ30064 FIS, CLONE ADRGL2000323.	X		
IPI00044353	MED12L	ISOFORM 4 OF MEDIATOR OF RNA POLYMERASE II TRANSCRIPTION SUBUNIT 12- LIKE PROTEIN.	X		
IPI00044790	MICB	MHC CLASS I POLYPEPTIDE-RELATED SEQUENCE B.	X		
IPI00044823	SLC2A13	PROTON MYO-INOSITOL COTRANSPORTER.	X		
IPI00045528	IFT140	PUTATIVE UNCHARACTERIZED PROTEIN GS114.	X		
IPI00045531	LYSMD1	LYSM AND PUTATIVE PEPTIDOGLYCAN-BINDING DOMAIN-CONTAINING PROTEIN 1.	X		
IPI00045764	FLJ14803	HYPOTHETICAL PROTEIN LOC84928.	X		
IPI00045865	ATOH8	PROTEIN ATONAL HOMOLOG 8.	X		
IPI00045912	KCNK16	POTASSIUM CHANNEL SUBFAMILY K MEMBER 16.	X		
IPI00045914	SPEN	MSX2-INTERACTING PROTEIN.	X		
IPI00045938	ZSCAN10	ZINC FINGER AND SCAN DOMAIN-CONTAINING PROTEIN 10.	X		
IPI00047476	ZFP92	UNCHARACTERIZED PROTEIN ENSP00000345765.	X		
IPI00053232	AGBL1	ISOFORM 3 OF CYTOSOLIC CARBOXYPEPTIDASE 4.	X		
IPI00053621	ZNF99	ZINC FINGER PROTEIN 99.	X		
IPI00056522	RILPL2	RAB INTERACTING LYSOSOMAL PROTEIN-LIKE 2.	X		
IPI00059169	CCDC102A	COILED-COIL DOMAIN-CONTAINING PROTEIN 102A.	X		
IPI00059308	LOC390667	SIMILAR TO PORTION OF NEURONAL PENTRAXIN I NPX1 OR NP1.	X		
IPI00059708	LRRC38	SIMILAR TO SLIT CG8355-PC, ISOFORM C.	X		
IPI00060201	SYTL4	ENDOCRINE TRANSMITTER REGULATORY PROTEIN.	X		
IPI00061178	CCBL2	KYNURENINE AMINOTRANSFERASE III ISOFORM 3.	X		
IPI00061277	MTBP	PROTEIN MDM2-BINDING PROTEIN.	X		
IPI00061521	PHF21B	ISOFORM 2 OF PHD FINGER PROTEIN 21B.	X		
IPI00061988	GLB1L3	GALACTOSIDASE, BETA 1 LIKE 3.	X		
IPI00062124	RPLP0P2	SIMILAR TO ACIDIC RIBOSOMAL PHOSPHOPROTEIN P0.	X		
IPI00062810	MGC16169	ISOFORM 3 OF TBC DOMAIN-CONTAINING PROTEIN KINASE-LIKE PROTEIN.	X		
IPI00063050	TMEM80	TRANSMEMBRANE PROTEIN 80.	X		
IPI00063183	DUSP3	DUSP3 PROTEIN.	X		

Table S1. Proteins Identified or Not in MS Patients Compared to Normals and Other Neurologic Disease (OND)					
**IPI**	**Gene**	**Protein Name**	**MS**	**Normal**	**OND**
IPI00063605	JUB	PROTEIN AJUBA.	X		
IPI00064158	TTBK1	ISOFORM 1 OF TAU-TUBULIN KINASE 1.	X		
IPI00064162	VCPIP1	DEUBIQUITINATING PROTEIN VCIP135.	X		
IPI00064219	KIAA1787	ISOFORM 1 OF NHR DOMAIN-CONTAINING PROTEIN KIAA1787.	X		
IPI00064351	C8orf76	UNCHARACTERIZED PROTEIN C8ORF76.	X		
IPI00064741	LMLN	ISOFORM 1 OF LEISHMANOLYSIN-LIKE PEPTIDASE.	X		
IPI00065371	OR1E3P	OLFACTORY RECEPTOR 1E3.	X		
IPI00065428	CCDC11	COILED-COIL DOMAIN-CONTAINING PROTEIN 11.	X		
IPI00065486	ABCB6	ISOFORM 4 OF MITOCHONDRIAL ATP-BINDING CASSETTE SUB-FAMILY B MEMBER 6.	X		
IPI00066317	SIGLEC11	SIALIC ACID-BINDING IG-LIKE LECTIN 11 PRECURSOR.	X		
IPI00071318	LUC7L	ISOFORM 1 OF PUTATIVE RNA-BINDING PROTEIN LUC7-LIKE 1.	X		
IPI00071703	GFM2	ELONGATION FACTOR G 2, MITOCHONDRIAL PRECURSOR.	X		
IPI00073196	LTBP3	ISOFORM 1 OF LATENT-TRANSFORMING GROWTH FACTOR BETA-BINDING PROTEIN 3 PRECURSOR.	X		
IPI00074876	PHF20	ISOFORM 1 OF PHD FINGER PROTEIN 20.	X		
IPI00074957	EGLN2	EGL NINE HOMOLOG 2.	X		
IPI00094507	UBXD4	UBX DOMAIN-CONTAINING PROTEIN 4.	X		
IPI00098952	NET1	62 KDA PROTEIN.	X		
IPI00099395	FBXL5	ISOFORM 1 OF F-BOX/LRR-REPEAT PROTEIN 5.	X		
IPI00099834	UBN1	UBINUCLEIN.	X		
IPI00100298	PIGV	GPI MANNOSYLTRANSFERASE 2.	X		
IPI00100369	UBQLN3	UBIQUILIN-3.	X		
IPI00100431	KCNF1	POTASSIUM VOLTAGE-GATED CHANNEL SUBFAMILY F MEMBER 1.	X		
IPI00100460	DARS2	ASPARTYL-TRNA SYNTHETASE, MITOCHONDRIAL PRECURSOR.	X		
IPI00100559	AP1S1	ISOFORM 2 OF AP-1 COMPLEX SUBUNIT SIGMA-1A.	X		
IPI00101652	SCLY	SELENOCYSTEINE LYASE.	X		
IPI00102065	KCNS1	POTASSIUM VOLTAGE-GATED CHANNEL SUBFAMILY S MEMBER 1.	X		
IPI00102377	ANKRD27	ANKYRIN REPEAT DOMAIN-CONTAINING PROTEIN 27.	X		
IPI00102423	BIRC8	BACULOVIRAL IAP REPEAT-CONTAINING PROTEIN 8.	X		
IPI00103036	PTCD2	PENTATRICOPEPTIDE REPEAT DOMAIN 2.	X		
IPI00103263	WAPAL	WAPAL PROTEIN.	X		
IPI00103335	GRIK5	KAINATE RECEPTOR SUBUNIT KA2A.	X		
IPI00103481	KRT72	ISOFORM 1 OF KERATIN, TYPE II CYTOSKELETAL 72.	X		
IPI00103528	RASSF5	ISOFORM 2 OF RAS ASSOCIATION DOMAIN-CONTAINING FAMILY PROTEIN 5.	X		

Table S1. Proteins Identified or Not in MS Patients Compared to Normals and Other Neurologic Disease (OND)					
IPI	Gene	Protein Name	MS	Normal	OND
IPI00103554	GATAD2B	TRANSCRIPTIONAL REPRESSOR P66 BETA.	X		
IPI00103563	ASB15	ANKYRIN REPEAT AND SOCS BOX PROTEIN 15.	X		
IPI00103636	WFDC2	ISOFORM 2 OF WAP FOUR-DISULFIDE CORE DOMAIN PROTEIN 2 PRECURSOR.	X		
IPI00103741	SSH1	ISOFORM 3 OF PROTEIN PHOSPHATASE SLINGSHOT HOMOLOG 1.	X		
IPI00103847	TMEM190	TRANSMEMBRANE PROTEIN 190 PRECURSOR.	X		
IPI00103879	DNASE2B	ISOFORM 1 OF DEOXYRIBONUCLEASE-2-BETA PRECURSOR.	X		
IPI00104219	TM2D1	BETA-AMYLOID BINDING PROTEIN PRECURSOR.	X		
IPI00104698		UNCHARACTERIZED PROTEIN ENSP00000301171.	X		
IPI00106552	AMBRA1	ISOFORM 2 OF ACTIVATING MOLECULE IN BECN1-REGULATED AUTOPHAGY PROTEIN 1.	X		
IPI00106663	CYLD	ISOFORM 1 OF PROBABLE UBIQUITIN CARBOXYL-TERMINAL HYDROLASE CYLD.	X		
IPI00107714	hCG_1998957; HLA-DRB1; HLA-DRB2; HLA-DRB3; HLA-DRB4; HLA-DRB5; LOC730415	MAJOR HISTOCOMPATIBILITY COMPLEX, CLASS II, DR BETA 5 PRECURSOR.	X		
IPI00107745	CROP	ISOFORM 1 OF CISPLATIN RESISTANCE-ASSOCIATED OVEREXPRESSED PROTEIN.	X		
IPI00140246	LRRC55	LEUCINE RICH REPEAT CONTAINING 55.	X		
IPI00145311	ZNF389	SIMILAR TO ZINC FINGER PROTEIN 192.	X		
IPI00147644	DOM3Z	ISOFORM 1 OF PROTEIN DOM3Z.	X		
IPI00151988	ZNF532	ZINC FINGER PROTEIN 532.	X		
IPI00152022	FAM47A	HYPOTHETICAL PROTEIN LOC158724.	X		
IPI00152152	CCDC33	ISOFORM 3 OF COILED-COIL DOMAIN-CONTAINING PROTEIN 33.	X		
IPI00152156	CXXC5	CXXC FINGER 5.	X		
IPI00152304	RBM46	PROBABLE RNA-BINDING PROTEIN 46.	X		
IPI00152380	MYO15A	MYOSIN-XV.	X		
IPI00152488	CD99L2	CD99 ANTIGEN-LIKE 2 ISOFORM E4.	X		
IPI00152491	CD99L2	CD99 ANTIGEN-LIKE 2 ISOFORM E3'-E4'-E3-E4.	X		
IPI00152661	PKD1L1	ISOFORM 1 OF POLYCYSTIC KIDNEY DISEASE 1-LIKE 1 PROTEIN.	X		
IPI00152875	ZNF384	ISOFORM 1 OF ZINC FINGER PROTEIN 384.	X		
IPI00152890	NOL6	ISOFORM 1 OF NUCLEOLAR PROTEIN 6.	X		
IPI00152946	RACGAP1	RAC GTPASE-ACTIVATING PROTEIN 1.	X		

Table S1. Proteins Identified or Not in MS Patients Compared to Normals and Other Neurologic Disease (OND)					
**IPI**	**Gene**	**Protein Name**	**MS**	**Normal**	**OND**
IPI00152990		253 KDA PROTEIN.	X		
IPI00154752	RHPN1	ISOFORM 1 OF RHOPHILIN-1.	X		
IPI00154766	TMEM174	ISOFORM 1 OF TRANSMEMBRANE PROTEIN 174.	X		
IPI00154813	TRAF3IP1	ISOFORM 1 OF TRAF3-INTERACTING PROTEIN 1.	X		
IPI00156982		UNCHARACTERIZED PROTEIN ENSP00000375469.	X		
IPI00157417	SEZ6L	ISOFORM 4 OF SEIZURE 6-LIKE PROTEIN PRECURSOR.	X		
IPI00157535	EPS8L1	ISOFORM 3 OF EPIDERMAL GROWTH FACTOR RECEPTOR KINASE SUBSTRATE 8-LIKE PROTEIN 1.	X		
IPI00158296	KIAA0564	HYPOTHETICAL PROTEIN LOC23078 ISOFORM A.	X		
IPI00158615	THOC2	THO COMPLEX 2 ISOFORM 1.	X		
IPI00159494	KIAA1377	UNCHARACTERIZED PROTEIN KIAA1377.	X		
IPI00159770	GMEB2	GLUCOCORTICOID MODULATORY ELEMENT-BINDING PROTEIN 2.	X		
IPI00159969	REST	ISOFORM 1 OF RE1-SILENCING TRANSCRIPTION FACTOR.	X		
IPI00160131	MYO9A	MYOSIN IXA.	X		
IPI00160290	BCL9	B-CELL CLL/LYMPHOMA 9 PROTEIN.	X		
IPI00160340	C20orf32	ISOFORM 1 OF HEF-LIKE PROTEIN.	X		
IPI00160622	CEP250	ISOFORM 1 OF CENTROSOME-ASSOCIATED PROTEIN CEP250.	X		
IPI00160775	SIDT1	SIDT1 PROTEIN.	X		
IPI00162664	ZNF323	ZINC FINGER PROTEIN 323.	X		
IPI00163230	COPS6	COP9 SIGNALOSOME COMPLEX SUBUNIT 6.	X		
IPI00163384	RDH12	RETINOL DEHYDROGENASE 12.	X		
IPI00163395	ZIC5	ZINC FINGER PROTEIN OF THE CEREBELLUM 5.	X		
IPI00163493	C9orf3	ISOFORM 1 OF AMINOPEPTIDASE O.	X		
IPI00163496	PPFIA1	ISOFORM 1 OF LIPRIN-ALPHA-1.	X		
IPI00163729	MKL1	MKL/MYOCARDIN-LIKE PROTEIN 1.	X		
IPI00164782	SLC7A10	ASC-TYPE AMINO ACID TRANSPORTER 1.	X		
IPI00165004	AHI1	ISOFORM 2 OF JOUBERIN.	X		
IPI00165249	PFTK1	ISOFORM 1 OF SERINE/THREONINE-PROTEIN KINASE PFTAIRE-1.	X		
IPI00165528	USP47	ISOFORM 2 OF UBIQUITIN CARBOXYL-TERMINAL HYDROLASE 47.	X		
IPI00165934	GPR98	ISOFORM 1 OF G-PROTEIN COUPLED RECEPTOR 98 PRECURSOR.	X		
IPI00166009	FBXL11	ISOFORM 1 OF JMJC DOMAIN-CONTAINING HISTONE DEMETHYLATION PROTEIN 1A.	X		
IPI00166031	CAMTA1	CALMODULIN-BINDING TRANSCRIPTION ACTIVATOR 1.	X		
IPI00166053	SLC34A3	SODIUM-DEPENDENT PHOSPHATE TRANSPORT PROTEIN 2C.	X		
IPI00166086	GCNT2	GLUCOSAMINYL (N-ACETYL) TRANSFERASE 2, I-BRANCHING ENZYME ISOFORM A.	X		

Table S1. Proteins Identified or Not in MS Patients Compared to Normals and Other Neurologic Disease (OND)					
**IPI**	**Gene**	**Protein Name**	**MS**	**Normal**	**OND**
IPI00166157	OR5T2	CDNA FLJ25625 FIS, CLONE STM02974.	X		
IPI00166304	FLJ37357	HYPOTHETICAL PROTEIN LOC284944.	X		
IPI00166533	C10orf140	HYPOTHETICAL PROTEIN LOC387640.	X		
IPI00166694	SLC22A24	SIMILAR TO SOLUTE CARRIER FAMILY 22 MEMBER 9.	X		
IPI00166698	CA6	CA6 PROTEIN.	X		
IPI00166711	ARHGEF1	FLJ00369 PROTEIN (FRAGMENT).	X		
IPI00166767	FBXL6	ISOFORM 1 OF F-BOX/LRR-REPEAT PROTEIN 6.	X		
IPI00166847	GK5	GLYCEROL KINASE 5.	X		
IPI00166861	C12orf45	UNCHARACTERIZED PROTEIN C12ORF45.	X		
IPI00166915	MS4A15	HYPOTHETICAL PROTEIN LOC219995 ISOFORM 2.	X		
IPI00166966	ESX1	HOMEOBOX PROTEIN ESX1.	X		
IPI00167124	CXorf22	ISOFORM 1 OF UNCHARACTERIZED PROTEIN CXORF22.	X		
IPI00167222	FAM123A	ISOFORM 2 OF PROTEIN FAM123A.	X		
IPI00167402		CDNA FLJ39811 FIS, CLONE SPLEN2009581.	X		
IPI00167403	LOC440320; LOC645202	SIMILAR TO CIS-GOLGI MATRIX PROTEIN GM130.	X		
IPI00167438		CDNA FLJ39672 FIS, CLONE SMINT2009233.	X		
IPI00167541	C15orf56	HYPOTHETICAL PROTEIN LOC644809.	X		
IPI00167616	ZDHHC22	PUTATIVE PALMITOYLTRANSFERASE ZDHHC22.	X		
IPI00167617	KLHDC7A	KELCH DOMAIN-CONTAINING PROTEIN 7A PRECURSOR.	X		
IPI00167681	FLJ37786	CDNA FLJ37786 FIS, CLONE BRHIP2028480.	X		
IPI00167732		CDNA FLJ37098 FIS, CLONE BRACE2019004.	X		
IPI00167747		CDNA FLJ37010 FIS, CLONE BRACE2009732.	X		
IPI00167778		CDNA FLJ36796 FIS, CLONE ADRGL2006817.	X		
IPI00167781	CYP11B1	CYTOCHROME P450, FAMILY 11, SUBFAMILY B, POLYPEPTIDE 1 ISOFORM 2 PRECURSOR.	X		
IPI00167858	IFLTD1	INTERMEDIATE FILAMENT TAIL DOMAIN CONTAINING 1.	X		
IPI00167867	KIAA1024	UPF0258 PROTEIN KIAA1024.	X		
IPI00167904	CCDC63	COILED-COIL DOMAIN-CONTAINING PROTEIN 63.	X		
IPI00168047	FAM73A	ISOFORM 1 OF PROTEIN FAM73A.	X		
IPI00168117	MGC16291	CDNA FLJ34785 FIS, CLONE NT2NE2004519.	X		
IPI00168150		CDNA FLJ34659 FIS, CLONE KIDNE2018863.	X		
IPI00168279	NLRP6	NACHT, LRR AND PYD DOMAINS-CONTAINING PROTEIN 6.	X		
IPI00168291	UGT3A2	UDP-GLUCURONOSYLTRANSFERASE 3A2 PRECURSOR.	X		

Table S1. Proteins Identified or Not in MS Patients Compared to Normals and Other Neurologic Disease (OND)					
**IPI**	**Gene**	**Protein Name**	**MS**	**Normal**	**OND**
IPI00168341	NETO2	ISOFORM 1 OF NEUROPILIN AND TOLLOID-LIKE PROTEIN 2 PRECURSOR.	X		
IPI00168603	CHDH	CHOLINE DEHYDROGENASE, MITOCHONDRIAL PRECURSOR.	X		
IPI00168915	RDM1	ISOFORM 1 OF RAD52 MOTIF-CONTAINING PROTEIN 1.	X		
IPI00168953	TNIP2	TNFAIP3-INTERACTING PROTEIN 2.	X		
IPI00168984	OR4K2	OLFACTORY RECEPTOR 4K2.	X		
IPI00169206	OR4C12	OLFACTORY RECEPTOR 4C12.	X		
IPI00169239	GRM6	SEVEN TRANSMEMBRANE HELIX RECEPTOR.	X		
IPI00170605	STRC	STEREOCILIN PRECURSOR.	X		
IPI00170641	DENND1A	ISOFORM 1 OF DENN DOMAIN-CONTAINING PROTEIN 1A.	X		
IPI00170706	TMEM2	TRANSMEMBRANE PROTEIN 2.	X		
IPI00170791	INADL	ISOFORM 2 OF INAD-LIKE PROTEIN.	X		
IPI00170865	MAGI3	MEMBRANE ASSOCIATED GUANYLATE KINASE, WW AND PDZ DOMAIN CONTAINING 3.	X		
IPI00170999	TMEM16G	ISOFORM 1 OF TRANSMEMBRANE PROTEIN 16G.	X		
IPI00171145	SPIRE1	ISOFORM 1 OF PROTEIN SPIRE HOMOLOG 1.	X		
IPI00171186	PDCD7	PROGRAMMED CELL DEATH PROTEIN 7.	X		
IPI00171614	SLC16A7	MONOCARBOXYLATE TRANSPORTER 2.	X		
IPI00171636	NAV1	ISOFORM 1 OF NEURON NAVIGATOR 1.	X		
IPI00171720	PIGP	ISOFORM A OF PHOSPHATIDYLINOSITOL N-ACETYLGLUCOSAMINYLTRANSFERASE SUBUNIT P.	X		
IPI00172460	AK2	ISOFORM 3 OF ADENYLATE KINASE ISOENZYME 2, MITOCHONDRIAL.	X		
IPI00172559	PALB2	PARTNER AND LOCALIZER OF BRCA2.	X		
IPI00172611		UNCHARACTERIZED PROTEIN ENSP00000378685.	X		
IPI00173449	FLJ35848	FLJ35848 PROTEIN.	X		
IPI00174859	XKR4	XK-RELATED PROTEIN 4.	X		
IPI00175092	RNF149	E3 UBIQUITIN-PROTEIN LIGASE RNF149 PRECURSOR.	X		
IPI00175096	TTC9C	TETRATRICOPEPTIDE REPEAT PROTEIN 9C.	X		
IPI00175146	CCDC131	ISOFORM 2 OF COILED-COIL DOMAIN-CONTAINING PROTEIN 131.	X		
IPI00176469	CABC1	ISOFORM 1 OF CHAPERONE ACTIVITY OF BC1 COMPLEX-LIKE, MITOCHONDRIAL PRECURSOR.	X		
IPI00176709	PHLPPL	ISOFORM 1 OF PH DOMAIN LEUCINE-RICH REPEAT PROTEIN PHOSPHATASE-LIKE.	X		
IPI00176829	RASSF3	ISOFORM 1 OF RAS ASSOCIATION DOMAIN-CONTAINING PROTEIN 3.	X		
IPI00177437	CRNKL1	ISOFORM 1 OF CROOKED NECK-LIKE PROTEIN 1.	X		
IPI00177856	C14orf172	ISOFORM 2 OF TRNA.	X		

Table S1. Proteins Identified or Not in MS Patients Compared to Normals and Other Neurologic Disease (OND)					
**IPI**	**Gene**	**Protein Name**	**MS**	**Normal**	**OND**
IPI00177938	TLE3	ISOFORM 2 OF TRANSDUCIN-LIKE ENHANCER PROTEIN 3.	X		
IPI00178072	SPECC1L	CYTOSPIN-A.	X		
IPI00178187	EHBP1	ISOFORM 1 OF EH DOMAIN-BINDING PROTEIN 1.	X		
IPI00178203	CHTF18	PROTEIN CTF18 HOMOLOG.	X		
IPI00178375	HISPPD1	ISOFORM 2 OF INOSITOL HEXAKISPHOSPHATE AND DIPHOSPHOINOSITOL-PENTAKISPHOSPHATE KINASE 2.	X		
IPI00178854	CNTN4	ISOFORM 1 OF CONTACTIN-4 PRECURSOR.	X		
IPI00178972	WDR23	ISOFORM 1 OF WD REPEAT-CONTAINING PROTEIN 23.	X		
IPI00179169	RPGRIP1	ISOFORM 1 OF X-LINKED RETINITIS PIGMENTOSA GTPASE REGULATOR- INTERACTING PROTEIN 1.	X		
IPI00180154	ATXN2	ATAXIN 2.	X		
IPI00180230	GRIP2	GLUTAMATE RECEPTOR INTERACTING PROTEIN 2.	X		
IPI00180325	IGSF22	IMMUNOGLOBULIN SUPERFAMILY MEMBER 22.	X		
IPI00180408	MYH15	MYOSIN-15.	X		
IPI00180428	FAM19A5	UNCHARACTERIZED PROTEIN FAM19A5 (FRAGMENT).	X		
IPI00180922	FAM48A	ISOFORM 2 OF PROTEIN FAM48A.	X		
IPI00181352	DNM2	DYNAMIN 2 ISOFORM 4.	X		
IPI00181359	SFRS2IP	SFRS2-INTERACTING PROTEIN.	X		
IPI00181556	ZNF777	CDNA FLJ90551 FIS, CLONE OVARC1000779, WEAKLY SIMILAR TO ZINC FINGER PROTEIN 157.	X		
IPI00181670	GRHL1	ISOFORM 2 OF GRAINYHEAD-LIKE PROTEIN 1 HOMOLOG.	X		
IPI00181896	SPO11	ISOFORM 2 OF MEIOTIC RECOMBINATION PROTEIN SPO11.	X		
IPI00182180	OTUD6B	OTU DOMAIN CONTAINING 6B.	X		
IPI00182427	C17orf47	UNCHARACTERIZED PROTEIN C17ORF47.	X		
IPI00182757	KIAA1967	ISOFORM 1 OF PROTEIN KIAA1967.	X		
IPI00182798	PAXIP1	ISOFORM 1 OF PAX-INTERACTING PROTEIN 1.	X		
IPI00183229	KIAA1545	SIMILAR TO AUTISM SUSCEPTIBILITY CANDIDATE 2.	X		
IPI00183349	ZNF667	R31155_1.	X		
IPI00183462	DIS3	ISOFORM 2 OF EXOSOME COMPLEX EXONUCLEASE RRP44.	X		
IPI00183913	IGSF10	ISOFORM 1 OF IMMUNOGLOBULIN SUPERFAMILY MEMBER 10 PRECURSOR.	X		
IPI00183933	SLC9A8	NA+/H+ EXCHANGER ISOFORM 8.	X		
IPI00183964	KIDINS220	PUTATIVE UNCHARACTERIZED PROTEIN DKFZP434F0621.	X		
IPI00183965	CACNB1	ISOFORM 1 OF VOLTAGE-DEPENDENT L-TYPE CALCIUM CHANNEL SUBUNIT BETA-1.	X		
IPI00184296		UNCHARACTERIZED PROTEIN ENSP00000305613 (FRAGMENT).	X		

Table S1. Proteins Identified or Not in MS Patients Compared to Normals and Other Neurologic Disease (OND)					
**IPI**	**Gene**	**Protein Name**	**MS**	**Normal**	**OND**
IPI00184572	PRKCH	PROTEIN KINASE C, ETA.	X		
IPI00184699	ZNF641	ISOFORM 1 OF ZINC FINGER PROTEIN 641.	X		
IPI00184772	DOCK6	DEDICATOR OF CYTOKINESIS PROTEIN 6.	X		
IPI00185084		SEVEN TRANSMEMBRANE HELIX RECEPTOR.	X		
IPI00185097	GINS3	CDNA FLJ13912 FIS, CLONE Y79AA1000230.	X		
IPI00215851	VILL	ISOFORM 2 OF VILLIN-LIKE PROTEIN.	X		
IPI00215977	IGF2	ISOFORM 2 OF INSULIN-LIKE GROWTH FACTOR II PRECURSOR.	X		
IPI00216184	PICALM	ISOFORM 2 OF PHOSPHATIDYLINOSITOL-BINDING CLATHRIN ASSEMBLY PROTEIN.	X		
IPI00216206	SLC8A3	103 KDA PROTEIN.	X		
IPI00216251	ABCC9	ISOFORM SUR2B OF ATP-BINDING CASSETTE TRANSPORTER SUB-FAMILY C MEMBER 9.	X		
IPI00216310	WNT16	ISOFORM WNT-16A OF PROTEIN WNT-16 PRECURSOR.	X		
IPI00216412	C6orf97	ISOFORM 2 OF COILED-COIL DOMAIN-CONTAINING PROTEIN C6ORF97.	X		
IPI00216433	LIMK1	ISOFORM 2 OF LIM DOMAIN KINASE 1.	X		
IPI00216546	ZDHHC5	ISOFORM 2 OF PROBABLE PALMITOYLTRANSFERASE ZDHHC5.	X		
IPI00216773	ALB	ALB PROTEIN.	X		
IPI00216813	ATXN7L2	ATAXIN-7-LIKE PROTEIN 2.	X		
IPI00216819	GJD4	GJD4 PROTEIN (FRAGMENT).	X		
IPI00216890	RELL1	RECEPTOR EXPRESSED IN LYMPHOID TISSUES LIKE 1.	X		
IPI00216905	CNGA4	CYCLIC NUCLEOTIDE GATED CHANNEL ALPHA 4.	X		
IPI00216932	ACSS1	ISOFORM 1 OF ACETYL-COENZYME A SYNTHETASE 2-LIKE, MITOCHONDRIAL PRECURSOR.	X		
IPI00216951	DARS	ASPARTYL-TRNA SYNTHETASE, CYTOPLASMIC.	X		
IPI00216984	CALML3	CALMODULIN-LIKE PROTEIN 3.	X		
IPI00217067	SLC22A9	SOLUTE CARRIER FAMILY 22 MEMBER 9.	X		
IPI00217121	C19orf21	UNCHARACTERIZED PROTEIN C19ORF21.	X		
IPI00217232	SUCLA2	ISOFORM 2 OF SUCCINYL-COA LIGASE [ADP-FORMING] BETA-CHAIN, MITOCHONDRIAL PRECURSOR.	X		
IPI00217269	GNAT2	GUANINE NUCLEOTIDE-BINDING PROTEIN G(T) SUBUNIT ALPHA-2.	X		
IPI00217423	BCAN	HYALURONAN BINDING PROTEIN (FRAGMENT).	X		
IPI00217481	GPR126	DEVELOPMENTALLY REGULATED G-PROTEIN-COUPLED RECEPTOR BETA 1.	X		
IPI00217492	TEK	RECEPTOR PROTEIN TYROSINE KINASE.	X		
IPI00217542	LOC130576	PUTATIVE UNCHARACTERIZED PROTEIN LOC130576.	X		
IPI00217554	SLC5A10	ISOFORM 3 OF SODIUM/GLUCOSE COTRANSPORTER 5.	X		

Table S1. Proteins Identified or Not in MS Patients Compared to Normals and Other Neurologic Disease (OND)					
**IPI**	**Gene**	**Protein Name**	**MS**	**Normal**	**OND**
IPI00217561	ITGB1	ISOFORM BETA-1C OF INTEGRIN BETA-1 PRECURSOR.	X		
IPI00217605	C6orf146	UNCHARACTERIZED PROTEIN C6ORF146.	X		
IPI00217620	C11orf82	ISOFORM 1 OF NITRIC OXIDE-INDUCIBLE GENE PROTEIN.	X		
IPI00217686	FTSJ3	PUTATIVE RRNA METHYLTRANSFERASE 3.	X		
IPI00217814	AGPAT3	ISOFORM 2 OF 1-ACYL-SN-GLYCEROL-3-PHOSPHATE ACYLTRANSFERASE GAMMA.	X		
IPI00217920	ALDH16A1	ISOFORM 1 OF ALDEHYDE DEHYDROGENASE FAMILY 16 MEMBER A1.	X		
IPI00218000	GPR113	ISOFORM 1 OF PROBABLE G-PROTEIN COUPLED RECEPTOR 113 PRECURSOR.	X		
IPI00218078	SYCP3	SYNAPTONEMAL COMPLEX PROTEIN 3.	X		
IPI00218270	TYR	ISOFORM 2 OF TYROSINASE PRECURSOR.	X		
IPI00218369	SHOX2	ISOFORM 2 OF SHORT STATURE HOMEOBOX PROTEIN 2.	X		
IPI00218648	ARID4A	ISOFORM III OF AT-RICH INTERACTIVE DOMAIN-CONTAINING PROTEIN 4A.	X		
IPI00218659	ITPR1	ISOFORM 3 OF INOSITOL 1,4,5-TRISPHOSPHATE RECEPTOR TYPE 1.	X		
IPI00218851	LYNX1	LY-6 NEUROTOXIN-LIKE PROTEIN 1 ISOFORM A.	X		
IPI00218852	VIL1	VILLIN-1.	X		
IPI00218971	PPM2C	[PYRUVATE DEHYDROGENASE [LIPOAMIDE]]-PHOSPHATASE 1, MITOCHONDRIAL PRECURSOR.	X		
IPI00219018	GAPDH	GLYCERALDEHYDE-3-PHOSPHATE DEHYDROGENASE.	X		
IPI00219065	AGL	ISOFORM 5 OF GLYCOGEN DEBRANCHING ENZYME.	X		
IPI00219068	NAT12	N-ACETYLTRANSFERASE 12.	X		
IPI00219168	SPTBN5	SPECTRIN BETA CHAIN, BRAIN 4.	X		
IPI00219299	TLN2	TALIN-2.	X		
IPI00219303	PGDS	GLUTATHIONE-REQUIRING PROSTAGLANDIN D SYNTHASE.	X		
IPI00219352	CBS	ISOFORM 1 OF CYSTATHIONINE BETA-SYNTHASE.	X		
IPI00219381	NDUFA2	NADH DEHYDROGENASE [UBIQUINONE] 1 ALPHA SUBCOMPLEX SUBUNIT 2.	X		
IPI00219462	TCFL5	ISOFORM 1 OF TRANSCRIPTION FACTOR-LIKE 5 PROTEIN.	X		
IPI00219585	PFKM	ISOFORM 2 OF 6-PHOSPHOFRUCTOKINASE, MUSCLE TYPE.	X		
IPI00219592	PRKACG	CAMP-DEPENDENT PROTEIN KINASE, GAMMA-CATALYTIC SUBUNIT.	X		
IPI00219849	RTEL1	ISOFORM 2 OF REGULATOR OF TELOMERE ELONGATION HELICASE 1.	X		
IPI00220006	DNMT3A	DNA CYTOSINE METHYLTRANSFERASE 3 ALPHA ISOFORM B.	X		
IPI00220219	COPB2	COATOMER SUBUNIT BETA'.	X		
IPI00220343	OMP	OLFACTORY MARKER PROTEIN.	X		
IPI00220373	IDE	INSULIN-DEGRADING ENZYME.	X		
IPI00220421	CENTD2	ISOFORM 2 OF CENTAURIN-DELTA-2.	X		
IPI00220477	MCRS1	ISOFORM 2 OF MICROSPHERULE PROTEIN 1.	X		

Table S1. Proteins Identified or Not in MS Patients Compared to Normals and Other Neurologic Disease (OND)					
**IPI**	**Gene**	**Protein Name**	**MS**	**Normal**	**OND**
IPI00220592	ITSN1	ISOFORM 2 OF INTERSECTIN-1.	X		
IPI00220618	SLCO1A2	ISOFORM OATP1B OF SOLUTE CARRIER ORGANIC ANION TRANSPORTER FAMILY MEMBER 1A2.	X		
IPI00220737	NCAM1	ISOFORM N-CAM 120 OF NEURAL CELL ADHESION MOLECULE 1, 120 KDA ISOFORM PRECURSOR.	X		
IPI00220878	SREBF1	ISOFORM SREBP-1C OF STEROL REGULATORY ELEMENT-BINDING PROTEIN 1.	X		
IPI00221067	PTPRA	ISOFORM 2 OF RECEPTOR-TYPE TYROSINE-PROTEIN PHOSPHATASE ALPHA PRECURSOR.	X		
IPI00221090	ZFHX2	ZINC FINGER HOMEOBOX PROTEIN 2.	X		
IPI00221199	GCN5L2	GCN5 GENERAL CONTROL OF AMINO-ACID SYNTHESIS 5-LIKE 2.	X		
IPI00221203	NLRP12	ISOFORM 2 OF NACHT, LRR AND PYD DOMAINS-CONTAINING PROTEIN 12.	X		
IPI00234679	SLC10A6	SOLUTE CARRIER FAMILY 10 MEMBER 6.	X		
IPI00235708	LOC651746	SIMILAR TO ANKYRIN REPEAT DOMAIN 33.	X		
IPI00238277	KIAA1086	UNCHARACTERIZED PROTEIN KIAA1086.	X		
IPI00238575	C10orf112	SIMILAR TO APICAL EARLY ENDOSOMAL GLYCOPROTEIN.	X		
IPI00239657	EPB41L4B	ISOFORM 3 OF BAND 4.1-LIKE PROTEIN 4B.	X		
IPI00240675	PDCD4	PROGRAMMED CELL DEATH 4 ISOFORM 2.	X		
IPI00246842	TAF1C	ISOFORM 1 OF TATA BOX-BINDING PROTEIN-ASSOCIATED FACTOR RNA POLYMERASE I SUBUNIT C.	X		
IPI00248651	REV3L	ISOFORM 1 OF DNA POLYMERASE ZETA CATALYTIC SUBUNIT.	X		
IPI00249970	LOC338667	SIMILAR TO CG32387-PB, ISOFORM B.	X		
IPI00251837	CECR2	CDNA FLJ34435 FIS, CLONE HLUNG2000955.	X		
IPI00252929	HOM-TES-103	CDNA FLJ20703 FIS, CLONE KAIA1965.	X		
IPI00288954	C12orf55	HYPOTHETICAL PROTEIN.	X		
IPI00289275	CILP	CARTILAGE INTERMEDIATE LAYER PROTEIN 1 PRECURSOR.	X		
IPI00289547	JUND	TRANSCRIPTION FACTOR JUN-D.	X		
IPI00289776	MYCBP2	MYC BINDING PROTEIN 2.	X		
IPI00289807	TRNT1	ISOFORM 1 OF TRNA-NUCLEOTIDYLTRANSFERASE 1, MITOCHONDRIAL PRECURSOR.	X		
IPI00289866	FOXO1	FORKHEAD BOX PROTEIN O1.	X		
IPI00290032	TRPM7	TRANSIENT RECEPTOR POTENTIAL CATION CHANNEL SUBFAMILY M MEMBER 7.	X		
IPI00290377	FOXC1	FORKHEAD BOX PROTEIN C1.	X		
IPI00290410	DNTTIP2	DEOXYNUCLEOTIDYLTRANSFERASE, TERMINAL, INTERACTING PROTEIN 2.	X		
IPI00290547	NPAT	NUCLEAR PROTEIN, ATAXIA-TELANGIECTASIA LOCUS.	X		
IPI00290799	C18orf19	UNCHARACTERIZED PROTEIN C18ORF19.	X		

Table S1. Proteins Identified or Not in MS Patients Compared to Normals and Other Neurologic Disease (OND)					
IPI	Gene	Protein Name	MS	Normal	OND
IPI00290916	MEI1	NOVEL PROTEIN SIMILAR TO MOUSE MEIOSIS DEFECTIVE 1 GENE.	X		
IPI00290949	ENOX1	ECTO-NOX DISULFIDE-THIOL EXCHANGER 1 (CONSTITUTIVE ECTO-NOX) (CNOX) (CANDIDATE GROWTH-RELATED AND TIME KEEPING CONSTITUTIVE HYDROQUINONE.	X		
IPI00291010	C17orf63	UNCHARACTERIZED PROTEIN C17ORF63.	X		
IPI00291624	SRrp35	OTTHUMP00000016846.	X		
IPI00291711	PLEKHG3	ISOFORM 1 OF PLECKSTRIN HOMOLOGY DOMAIN-CONTAINING FAMILY G MEMBER 3.	X		
IPI00291802	LMO7	ISOFORM 3 OF LIM DOMAIN ONLY PROTEIN 7.	X		
IPI00291851	AMZ1	ARCHAEMETZINCIN-1.	X		
IPI00291997	EYA1	EYES ABSENT 1 ISOFORM C.	X		
IPI00292009	PALLD	ISOFORM 2 OF PALLADIN.	X		
IPI00292208	IER5	IMMEDIATE EARLY RESPONSE GENE 5 PROTEIN.	X		
IPI00292228	GSK3A	GLYCOGEN SYNTHASE KINASE-3 ALPHA.	X		
IPI00292468	KLHL6	KELCH-LIKE PROTEIN 6.	X		
IPI00292567	POMGNT1	PROTEIN O-LINKED MANNOSE BETA1,2-N-ACETYLGLUCOSAMINYLTRANSFERASE.	X		
IPI00292794	LRRC49	74 KDA PROTEIN.	X		
IPI00292880	E2F4	TRANSCRIPTION FACTOR E2F4.	X		
IPI00293038	NR4A3	ISOFORM BETA OF NUCLEAR RECEPTOR SUBFAMILY 4 GROUP A MEMBER 3.	X		
IPI00293078	DDX27	PROBABLE ATP-DEPENDENT RNA HELICASE DDX27.	X		
IPI00293080	FAM75A7	PROTEIN FAM75A7.	X		
IPI00293125	ACOX2	ACYL-COENZYME A OXIDASE 2, PEROXISOMAL.	X		
IPI00293235	BBS7	ISOFORM 1 OF BARDET-BIEDL SYNDROME 7 PROTEIN.	X		
IPI00293312	ZC3H18	ISOFORM 2 OF ZINC FINGER CCCH DOMAIN-CONTAINING PROTEIN 18.	X		
IPI00293336	LOC153364	SIMILAR TO METALLO-BETA-LACTAMASE SUPERFAMILY PROTEIN.	X		
IPI00293381	TMEM183A	ISOFORM 1 OF TRANSMEMBRANE PROTEIN 183A.	X		
IPI00293593	TCTE1	OTTHUMP00000016524.	X		
IPI00293655	DDX1	ATP-DEPENDENT RNA HELICASE DDX1.	X		
IPI00293665	KRT6B	KERATIN, TYPE II CYTOSKELETAL 6B.	X		
IPI00293887	STARD8	PUTATIVE UNCHARACTERIZED PROTEIN DKFZP686H1668.	X		
IPI00294173	RP4-691N24.1	NINEIN-LIKE PROTEIN.	X		
IPI00294435	SLU7	PRE-MRNA-SPLICING FACTOR SLU7.	X		
IPI00294603	ZMYM2	SIMILAR TO ZINC FINGER MYM-TYPE PROTEIN 5.	X		
IPI00294649	KRT35	KERATIN 35.	X		
IPI00294744	PARN	POLY(A)-SPECIFIC RIBONUCLEASE PARN.	X		

Table S1. Proteins Identified or Not in MS Patients Compared to Normals and Other Neurologic Disease (OND)					
**IPI**	**Gene**	**Protein Name**	**MS**	**Normal**	**OND**
IPI00294749	KIF27	ISOFORM 1 OF KINESIN-LIKE PROTEIN KIF27.	X		
IPI00294840	AIM1	ABSENT IN MELANOMA 1 PROTEIN.	X		
IPI00295313	MRVI1	ISOFORM 3 OF PROTEIN MRVI1.	X		
IPI00295363	OTC	ORNITHINE CARBAMOYLTRANSFERASE, MITOCHONDRIAL PRECURSOR.	X		
IPI00295388	GLDC	115 KDA PROTEIN.	X		
IPI00295401	MAP3K10	MITOGEN-ACTIVATED PROTEIN KINASE KINASE KINASE 10.	X		
IPI00295425	CCDC61; LOC388554	COILED-COIL DOMAIN CONTAINING 61.	X		
IPI00295774	SLC23A2	SOLUTE CARRIER FAMILY 23 MEMBER 2.	X		
IPI00295898	CCDC144A; CCDC144B; LOC652491; LOC731479	ISOFORM 1 OF COILED-COIL DOMAIN-CONTAINING PROTEIN 144B.	X		
IPI00296022	UQCRH	CYTOCHROME B-C1 COMPLEX SUBUNIT 6, MITOCHONDRIAL PRECURSOR.	X		
IPI00296180	PLAU	UROKINASE-TYPE PLASMINOGEN ACTIVATOR PRECURSOR.	X		
IPI00296432	IWS1	ISOFORM 1 OF IWS1 HOMOLOG.	X		
IPI00296789	FBXO3	ISOFORM 1 OF F-BOX ONLY PROTEIN 3.	X		
IPI00297160	CD44	ISOFORM 12 OF CD44 ANTIGEN PRECURSOR.	X		
IPI00297191	MED14	MEDIATOR OF RNA POLYMERASE II TRANSCRIPTION SUBUNIT 14.	X		
IPI00297349	SOCS3	SUPPRESSOR OF CYTOKINE SIGNALING 3.	X		
IPI00297572	AQR	INTRON-BINDING PROTEIN AQUARIUS.	X		
IPI00297587	LOC202051	CDNA FLJ25874 FIS, CLONE CBR02446 (FRAGMENT).	X		
IPI00297593	USP34	UBIQUITIN SPECIFIC PROTEASE 34.	X		
IPI00297656	CNGB1	CYCLIC NUCLEOTIDE GATED CHANNEL BETA 1.	X		
IPI00298136	FLJ39653	CDNA FLJ39653 FIS, CLONE SMINT2005387.	X		
IPI00298235	POMT1	ISOFORM 1 OF PROTEIN O-MANNOSYL-TRANSFERASE 1.	X		
IPI00298301	MYH3	MYOSIN-3.	X		
IPI00298417	GYLTL1B	ISOFORM 1 OF GLYCOSYLTRANSFERASE-LIKE PROTEIN LARGE2.	X		
IPI00298558	PDCD10	PROGRAMMED CELL DEATH PROTEIN 10.	X		
IPI00298731	PPP1R10	SERINE/THREONINE-PROTEIN PHOSPHATASE 1 REGULATORY SUBUNIT 10.	X		
IPI00298817	RUNDC3A	SIMILAR TO RAP2 INTERACTING PROTEIN 8.	X		
IPI00298912	CORO2B	CORONIN, ACTIN BINDING PROTEIN, 2B.	X		
IPI00299921	SLC35B4	ISOFORM 1 OF UDP-XYLOSE AND UDP-N-ACETYLGLUCOSAMINE TRANSPORTER.	X		
IPI00300074	FARSB	PHENYLALANYL-TRNA SYNTHETASE BETA CHAIN.	X		

Table S1. Proteins Identified or Not in MS Patients Compared to Normals and Other Neurologic Disease (OND)					
**IPI**	**Gene**	**Protein Name**	**MS**	**Normal**	**OND**
IPI00300213	MEX3B	ISOFORM 1 OF RNA-BINDING PROTEIN MEX3B.	X		
IPI00300332	BCL11A	ISOFORM 1 OF B-CELL LYMPHOMA/LEUKEMIA 11A.	X		
IPI00300384	ERBB2	RECEPTOR TYROSINE-PROTEIN KINASE ERBB-2 PRECURSOR.	X		
IPI00300860	PDZD4	PDZD4 PROTEIN.	X		
IPI00300905		CDNA FLJ34460 FIS, CLONE HLUNG2002942.	X		
IPI00301112		HOR5'BETA13.	X		
IPI00301139	MED17	ISOFORM 1 OF MEDIATOR OF RNA POLYMERASE II TRANSCRIPTION SUBUNIT 17.	X		
IPI00301163	KTELC1	KTEL MOTIF-CONTAINING PROTEIN 1 PRECURSOR.	X		
IPI00301263	CAD	CAD PROTEIN.	X		
IPI00301711	FAM148A	NUCLEAR LOCALIZED FACTOR 1.	X		
IPI00301907	HLCS	BIOTIN--PROTEIN LIGASE.	X		
IPI00301994	FAHD2B	FUMARYLACETOACETATE HYDROLASE DOMAIN-CONTAINING PROTEIN 2B.	X		
IPI00302151	CCDC87	COILED-COIL DOMAIN-CONTAINING PROTEIN 87.	X		
IPI00302309	TTC17	TTC17 PROTEIN.	X		
IPI00302383	CFTR	CYSTIC FIBROSIS TRANSMEMBRANE CONDUCTANCE REGULATOR ATP-BINDING CASSETTE SUB-FAMILY C MEMBER 7.	X		
IPI00302453	DNAH9	CILIARY DYNEIN HEAVY CHAIN 9.	X		
IPI00302688	ECHDC1	ISOFORM 1 OF ENOYL-COA HYDRATASE DOMAIN-CONTAINING PROTEIN 1.	X		
IPI00302829	RB1	RETINOBLASTOMA-ASSOCIATED PROTEIN.	X		
IPI00303053	EIF2AK3	EUKARYOTIC TRANSLATION INITIATION FACTOR 2-ALPHA KINASE 3.	X		
IPI00303063	PDS5A	ISOFORM 2 OF SISTER CHROMATID COHESION PROTEIN PDS5 HOMOLOG A.	X		
IPI00303258	LMCD1	LIM AND CYSTEINE-RICH DOMAINS PROTEIN 1.	X		
IPI00303481	OR51G1	OLFACTORY RECEPTOR 51G1.	X		
IPI00303746	DHX58	PROBABLE ATP-DEPENDENT RNA HELICASE DHX58.	X		
IPI00304028	RBL2	RETINOBLASTOMA-LIKE PROTEIN 2.	X		
IPI00304232	WDR12	WD REPEAT-CONTAINING PROTEIN 12.	X		
IPI00304493	SRFBP1	SERUM RESPONSE FACTOR BINDING PROTEIN 1.	X		
IPI00304549	KIAA1755	HYPOTHETICAL PROTEIN LOC85449.	X		
IPI00304578	FGFR4	FIBROBLAST GROWTH FACTOR RECEPTOR 4 PRECURSOR.	X		
IPI00304895	MOCOS	MOLYBDENUM COFACTOR SULFURASE.	X		
IPI00305020	SH2D3A	SH2 DOMAIN-CONTAINING PROTEIN 3A.	X		
IPI00305042	MYEOV	MYELOMA-OVEREXPRESSED GENE PROTEIN.	X		
IPI00305068	PRPF6	PRE-MRNA-PROCESSING FACTOR 6.	X		
IPI00305348	ZNF2	ZINC FINGER PROTEIN 2.	X		

Table S1. Proteins Identified or Not in MS Patients Compared to Normals and Other Neurologic Disease (OND)					
**IPI**	**Gene**	**Protein Name**	**MS**	**Normal**	**OND**
IPI00305416	TMEM100	TRANSMEMBRANE PROTEIN 100.	X		
IPI00306369	NSUN2	TRNA.	X		
IPI00306718	SYCP2	SYNAPTONEMAL COMPLEX PROTEIN 2.	X		
IPI00306774	TAPBPL	TAPASIN-RELATED PROTEIN PRECURSOR.	X		
IPI00306903	PPP1R13B	APOPTOSIS-STIMULATING OF P53 PROTEIN 1.	X		
IPI00306984	KCNH8	POTASSIUM VOLTAGE-GATED CHANNEL SUBFAMILY H MEMBER 8.	X		
IPI00307009	POM121L2	POM121-LIKE PROTEIN 2.	X		
IPI00307352	ZNF474	ZINC FINGER PROTEIN 474.	X		
IPI00307446	PCDH24	PROTOCADHERIN LKC PRECURSOR.	X		
IPI00307545	TNS1	TENSIN-1.	X		
IPI00328144	TAF2	TRANSCRIPTION INITIATION FACTOR TFIID SUBUNIT 2.	X		
IPI00328149	EIF2AK1	ISOFORM 1 OF EUKARYOTIC TRANSLATION INITIATION FACTOR 2-ALPHA KINASE 1.	X		
IPI00328254	PAN2	ISOFORM 2 OF PAB-DEPENDENT POLY(A)-SPECIFIC RIBONUCLEASE SUBUNIT 2.	X		
IPI00328350	FAM129A	NIBAN PROTEIN.	X		
IPI00328354	MAGED1	ISOFORM 1 OF MELANOMA-ASSOCIATED ANTIGEN D1.	X		
IPI00328379	GPSM2	G-PROTEIN SIGNALING MODULATOR 2.	X		
IPI00328573	ATG4D	CYSTEINE PROTEASE ATG4D.	X		
IPI00328644	FPR1	FMET-LEU-PHE RECEPTOR.	X		
IPI00328719	SLC15A2	OLIGOPEPTIDE TRANSPORTER, KIDNEY ISOFORM.	X		
IPI00328722	SLC9A11	SODIUM/HYDROGEN EXCHANGER 11.	X		
IPI00328815	USP48	ISOFORM 1 OF UBIQUITIN CARBOXYL-TERMINAL HYDROLASE 48.	X		
IPI00328867	SRC	ISOFORM 2 OF PROTO-ONCOGENE TYROSINE-PROTEIN KINASE SRC.	X		
IPI00328911	HECTD1	E3 UBIQUITIN-PROTEIN LIGASE HECTD1.	X		
IPI00329104	LILRA3	LEUKOCYTE IMMUNOGLOBULIN-LIKE RECEPTOR SUBFAMILY A MEMBER 3 PRECURSOR.	X		
IPI00329181	TMEM39A	ISOFORM 1 OF TRANSMEMBRANE PROTEIN 39A.	X		
IPI00329260	C13orf23	UNCHARACTERIZED PROTEIN KIAA2032.	X		
IPI00329535	THBS3	THROMBOSPONDIN-3 PRECURSOR.	X		
IPI00329560	RAPGEF4	ISOFORM 1 OF RAP GUANINE NUCLEOTIDE EXCHANGE FACTOR 4.	X		
IPI00329638	ZAK	ISOFORM 1 OF MITOGEN-ACTIVATED PROTEIN KINASE KINASE KINASE MLT.	X		
IPI00329707	BCORL1	ISOFORM 3 OF BCOR-LIKE PROTEIN 1.	X		
IPI00329721	ABCC5	ATP-BINDING CASSETTE C5 SPLICING VARIANT A.	X		
IPI00332095	BCAS3	ISOFORM 2 OF BREAST CARCINOMA-AMPLIFIED SEQUENCE 3.	X		
IPI00332155	GPRIN1	ISOFORM 1 OF G PROTEIN-REGULATED INDUCER OF NEURITE OUTGROWTH 1.	X		

Table S1. Proteins Identified or Not in MS Patients Compared to Normals and Other Neurologic Disease (OND)					
**IPI**	**Gene**	**Protein Name**	**MS**	**Normal**	**OND**
IPI00333325	GRASP	ISOFORM 1 OF GENERAL RECEPTOR FOR PHOSPHOINOSITIDES 1-ASSOCIATED SCAFFOLD PROTEIN.	X		
IPI00333420	SRPK2	SERINE/THREONINE-PROTEIN KINASE SRPK2.	X		
IPI00333449	ZNF605	ZINC FINGER PROTEIN 605.	X		
IPI00334186	VWA3B	CDNA FLJ25975 FIS, CLONE TST07107.	X		
IPI00334190	STOML2	STOMATIN-LIKE PROTEIN 2.	X		
IPI00334930	LOC728008	UNCHARACTERIZED PROTEIN ENSP00000375311.	X		
IPI00335355	SLC6A17	ORPHAN SODIUM- AND CHLORIDE-DEPENDENT NEUROTRANSMITTER TRANSPORTER NTT4.	X		
IPI00335509	DPYSL5	DIHYDROPYRIMIDINASE-RELATED PROTEIN 5.	X		
IPI00336000	ZCCHC6	ISOFORM 6 OF ZINC FINGER CCHC DOMAIN-CONTAINING PROTEIN 6.	X		
IPI00337307	HTF9C	ISOFORM 1 OF HPAII TINY FRAGMENTS LOCUS 9C PROTEIN (FRAGMENT).	X		
IPI00337315	RBBP6	ISOFORM 1 OF RETINOBLASTOMA-BINDING PROTEIN 6.	X		
IPI00337541	NNT	NAD(P) TRANSHYDROGENASE, MITOCHONDRIAL PRECURSOR.	X		
IPI00337549	OSGIN1	ISOFORM 2 OF OXIDATIVE STRESS-INDUCED GROWTH INHIBITOR 1.	X		
IPI00373894	KIAA0100	ISOFORM 1 OF UPF0378 FAMILY PROTEIN KIAA0100 PRECURSOR.	X		
IPI00374218	FLJ36157	HYPOTHETICAL PROTEIN.	X		
IPI00374317	SAMD5	STERILE ALPHA MOTIF DOMAIN-CONTAINING PROTEIN 5.	X		
IPI00374337	GRID1	GLUTAMATE RECEPTOR DELTA-1 SUBUNIT PRECURSOR.	X		
IPI00374550	LOC389813	SIMILAR TO CG15216-PA.	X		
IPI00374777	C1orf120	PUTATIVE UNCHARACTERIZED PROTEIN C1ORF120.	X		
IPI00375220	NHS	ISOFORM 1 OF NANCE-HORAN SYNDROME PROTEIN.	X		
IPI00375254	FBXO18	F-BOX ONLY PROTEIN, HELICASE, 18 ISOFORM 1.	X		
IPI00375441	FUBP1	ISOFORM 1 OF FAR UPSTREAM ELEMENT-BINDING PROTEIN 1.	X		
IPI00375617	ABHD12B	ISOFORM 2 OF ABHYDROLASE DOMAIN-CONTAINING PROTEIN 12B.	X		
IPI00375682	NRK	ISOFORM 2 OF NIK-RELATED PROTEIN KINASE.	X		
IPI00375793	RGPD4	ISOFORM 1 OF RANBP2-LIKE AND GRIP DOMAIN-CONTAINING PROTEIN 4.	X		
IPI00375947	RTL1	SIMILAR TO RETROTRANSPOSON-LIKE 1.	X		
IPI00376002	TRIM46	ISOFORM 1 OF TRIPARTITE MOTIF-CONTAINING PROTEIN 46.	X		
IPI00376221	EPB41L5	ERYTHROCYTE MEMBRANE PROTEIN BAND 4.1 LIKE 5.	X		
IPI00376229	PACS1	ISOFORM 1 OF PHOSPHOFURIN ACIDIC CLUSTER SORTING PROTEIN 1.	X		
IPI00376299		OLFACTORY RECEPTOR.	X		
IPI00376788	C7orf51	CDNA FLJ42783 FIS, CLONE BRAWH3005981.	X		
IPI00376791	OR1L1	OLFACTORY RECEPTOR, FAMILY 1, SUBFAMILY L, MEMBER 1.	X		

Table S1. Proteins Identified or Not in MS Patients Compared to Normals and Other Neurologic Disease (OND)					
**IPI**	**Gene**	**Protein Name**	**MS**	**Normal**	**OND**
IPI00376955	PCTK2	SERINE/THREONINE-PROTEIN KINASE PCTAIRE-2.	X		
IPI00377022	KIF17	ISOFORM 2 OF KINESIN-LIKE PROTEIN KIF17.	X		
IPI00377041	CSMD3	CUB AND SUSHI MULTIPLE DOMAINS 3 ISOFORM 1.	X		
IPI00377187	AKNA	ISOFORM 1 OF AT-HOOK-CONTAINING TRANSCRIPTION FACTOR.	X		
IPI00382447		IG LAMBDA CHAIN V-VI REGION AR.	X		
IPI00382455		IG HEAVY CHAIN V-I REGION EU.	X		
IPI00382577		KAPPA 1 LIGHT CHAIN VARIABLE REGION (FRAGMENT).	X		
IPI00382589	RREB1	194 KDA PROTEIN.	X		
IPI00382748	HYI	ISOFORM 3 OF PUTATIVE HYDROXYPYRUVATE ISOMERASE.	X		
IPI00382752	WWOX	OXIDOREDUCTASE.	X		
IPI00382990		DERP12.	X		
IPI00383010		PUTATIVE G-PROTEIN COUPLED RECEPTOR.	X		
IPI00383105	CHD9	ISOFORM 1 OF CHROMODOMAIN-HELICASE-DNA-BINDING PROTEIN 9.	X		
IPI00383295		PUTATIVE UNCHARACTERIZED PROTEIN.	X		
IPI00383444	TCP11L1	T-COMPLEX PROTEIN 11-LIKE 1.	X		
IPI00383584	INSIG1	INSULIN INDUCED GENE 1 ISOFORM 3.	X		
IPI00383597	SLC4A2	ISOFORM A OF ANION EXCHANGE PROTEIN 2.	X		
IPI00383649	CEBPA	HP8 PEPTIDE.	X		
IPI00383975	CHGA	FULL-LENGTH CDNA CLONE CS0DN001YP04 OF ADULT BRAIN OF HOMO SAPIENS (FRAGMENT).	X		
IPI00383995	C10orf93	ISOFORM 2 OF UNCHARACTERIZED PROTEIN C10ORF93.	X		
IPI00384028	PAPOLA	ISOFORM 1 OF POLY(A) POLYMERASE ALPHA.	X		
IPI00384096		PROSTATE APOPTOSIS RESPONSE-4.	X		
IPI00384127	PPP2R5C	FULL-LENGTH CDNA CLONE CS0DF017YJ08 OF FETAL BRAIN OF HOMO SAPIENS (FRAGMENT).	X		
IPI00384397		MYOSIN-REACTIVE IMMUNOGLOBULIN LIGHT CHAIN VARIABLE REGION (FRAGMENT).	X		
IPI00384401		MYOSIN-REACTIVE IMMUNOGLOBULIN KAPPA CHAIN VARIABLE REGION (FRAGMENT).	X		
IPI00384443	BRD9	SARCOMA ANTIGEN NY-SAR-29.	X		
IPI00384458	INTU	ISOFORM 1 OF PDZ DOMAIN-CONTAINING PROTEIN 6.	X		
IPI00384576		IG KAPPA CHAIN V-III REGION HIC PRECURSOR.	X		
IPI00384657	FLJ36144	CDNA FLJ40095 FIS, CLONE TESTI2003940, WEAKLY SIMILAR TO EBNA-1 NUCLEAR PROTEIN.	X		
IPI00384709	ZNF610	ISOFORM 2 OF ZINC FINGER PROTEIN 610.	X		

Table S1. Proteins Identified or Not in MS Patients Compared to Normals and Other Neurologic Disease (OND)					
**IPI**	**Gene**	**Protein Name**	**MS**	**Normal**	**OND**
IPI00384972	KIAA1267	ISOFORM 1 OF UNCHARACTERIZED PROTEIN KIAA1267.	X		
IPI00385001	NUP188	ISOFORM 2 OF NUCLEOPORIN NUP188 HOMOLOG.	X		
IPI00385059		REARRANGED VH4-34 V GENE SEGMENT (FRAGMENT).	X		
IPI00385151	ASB7	ISOFORM 1 OF ANKYRIN REPEAT AND SOCS BOX PROTEIN 7.	X		
IPI00385206	PCBP4	CBP.	X		
IPI00385254		IG LAMBDA CHAIN V-I REGION WAH.	X		
IPI00385305	EMR2	EMR2 PROTEIN.	X		
IPI00385341	FNDC5	FNDC5 PROTEIN.	X		
IPI00385461	FUCA2	OTTHUMP00000017333.	X		
IPI00385555		IG KAPPA CHAIN V-I REGION BAN.	X		
IPI00385631	ZZEF1	ISOFORM 1 OF ZINC FINGER ZZ-TYPE AND EF-HAND DOMAIN-CONTAINING PROTEIN 1.	X		
IPI00385686		IG GAMMA LAMBDA CHAIN V-II REGION DOT.	X		
IPI00385772		CDNA FLJ35331 FIS, CLONE PROST2014659.	X		
IPI00385900		SYNOVIAL STIMULATORY PROTEIN P205 (FRAGMENT).	X		
IPI00385938		PRO0898.	X		
IPI00385995	PRKAG2	CDNA FLJ90194 FIS, CLONE MAMMA1001284.	X		
IPI00386035		PROFILAGGRIN (FRAGMENT).	X		
IPI00386197		FLJ00291 PROTEIN (FRAGMENT).	X		
IPI00386271	SLC25A12	CALCIUM-BINDING MITOCHONDRIAL CARRIER PROTEIN ARALAR1.	X		
IPI00386383	GPR109A	SEVEN TRANSMEMBRANE HELIX RECEPTOR.	X		
IPI00386501	GABBR2	HRIHFB2099 PROTEIN (FRAGMENT).	X		
IPI00386687	LRRFIP1	LRRFIP1 PROTEIN.	X		
IPI00387027		IG KAPPA CHAIN V-I REGION GAL.	X		
IPI00387037	ZNF548	ISOFORM 1 OF ZINC FINGER PROTEIN 548.	X		
IPI00394790	TEX9	TESTIS-EXPRESSED SEQUENCE 9 PROTEIN.	X		
IPI00394793	PLEKHG5	PLECKSTRIN HOMOLOGY DOMAIN CONTAINING FAMILY G MEMBER 5 ISOFORM A.	X		
IPI00394805	C1orf122	UNCHARACTERIZED PROTEIN C1ORF122.	X		
IPI00394833	ZNF642	ZINC FINGER PROTEIN 642.	X		
IPI00394847	SMTNL2	50 KDA PROTEIN.	X		
IPI00394855	C12orf63	PUTATIVE UNCHARACTERIZED PROTEIN C12ORF63.	X		
IPI00394856	KIF7	KINESIN-LIKE PROTEIN KIF7.	X		
IPI00394927	LOC727857	SIMILAR TO PANCREAS SPECIFIC TRANSCRIPTION FACTOR, 1A.	X		
IPI00395474	SAMD1	ATHERIN.	X		

Table S1. Proteins Identified or Not in MS Patients Compared to Normals and Other Neurologic Disease (OND)					
**IPI**	**Gene**	**Protein Name**	**MS**	**Normal**	**OND**
IPI00395604	LIPI	ISOFORM 2 OF LIPASE MEMBER I PRECURSOR.	X		
IPI00395803	C7orf46	ISOFORM 1 OF UNCHARACTERIZED PROTEIN C7ORF46.	X		
IPI00396069	ZNF571	ZINC FINGER PROTEIN 571.	X		
IPI00396218	SCYL2	SCY1-LIKE PROTEIN 2.	X		
IPI00396234	ATP11C	ISOFORM 1 OF PROBABLE PHOSPHOLIPID-TRANSPORTING ATPASE IG.	X		
IPI00396310	AFF1	AF4/FMR2 FAMILY, MEMBER 1.	X		
IPI00396634	CTA-221G9.4	ISOFORM 1 OF UNCHARACTERIZED PROTEIN KIAA1671.	X		
IPI00397016		UNCHARACTERIZED PROTEIN ENSP00000361672.	X		
IPI00397575	LRRC41	92 KDA PROTEIN.	X		
IPI00397764	LOC388630	SIMILAR TO C05G5.5.	X		
IPI00397801	FLG2	IFAPSORIASIN.	X		
IPI00397820		185 KDA PROTEIN.	X		
IPI00397891	LOC388963	SIMILAR TO SHORT-CHAIN DEHYDROGENASE/REDUCTASE 1.	X		
IPI00397977	ZXDC	ISOFORM 1 OF ZINC FINGER PROTEIN ZXDC.	X		
IPI00398220		MYOSIN-REACTIVE IMMUNOGLOBULIN LIGHT CHAIN VARIABLE REGION (FRAGMENT).	X		
IPI00398347	LOC387763	HYPOTHETICAL PROTEIN.	X		
IPI00398505	USP24	UBIQUITIN CARBOXYL-TERMINAL HYDROLASE 24.	X		
IPI00398607	LOC283755	HYPOTHETICAL PROTEIN LOC283755.	X		
IPI00398625	HRNR	HORNERIN.	X		
IPI00398808	WNK2	ISOFORM 1 OF SERINE/THREONINE-PROTEIN KINASE WNK2.	X		
IPI00399007	IGHG2	PUTATIVE UNCHARACTERIZED PROTEIN DKFZP686I04196 (FRAGMENT).	X		
IPI00399037	HNRPA1P4	SIMILAR TO HETEROGENEOUS NUCLEAR RIBONUCLEOPROTEIN A1.	X		
IPI00400774	DACH1	UNCHARACTERIZED PROTEIN DACH1.	X		
IPI00400838		HERV-H_3Q26 PROVIRUS ANCESTRAL ENV POLYPROTEIN PRECURSOR.	X		
IPI00400923	SH3PXD2A	SH3 MULTIPLE DOMAINS 1.	X		
IPI00401142	PHACTR2	ISOFORM 1 OF PHOSPHATASE AND ACTIN REGULATOR 2.	X		
IPI00401195	FLJ32549	FLJ32549 PROTEIN.	X		
IPI00401203	LOC401433	CDNA FLJ45817 FIS, CLONE NT2RP7020343.	X		
IPI00401476	LOC145757	HYPOTHETICAL LOC145757.	X		
IPI00401758	ANKS3	ANKYRIN REPEAT AND SAM DOMAIN-CONTAINING PROTEIN 3.	X		
IPI00401831	PLEKHH1	ISOFORM 1 OF PLECKSTRIN HOMOLOGY DOMAIN-CONTAINING FAMILY H MEMBER 1.	X		
IPI00401956	AATK	APOPTOSIS-ASSOCIATED TYROSINE KINASE.	X		
IPI00402077	SHE	SH2 DOMAIN-CONTAINING ADAPTER PROTEIN E.	X		
IPI00402189		UNCHARACTERIZED PROTEIN ENSP00000334201.	X		

Table S1. Proteins Identified or Not in MS Patients Compared to Normals and Other Neurologic Disease (OND)					
**IPI**	**Gene**	**Protein Name**	**MS**	**Normal**	**OND**
IPI00402559	LOC388210	SIMILAR TO CG15828-PA, ISOFORM A.	X		
IPI00402657	RPAP1	ISOFORM 1 OF RNA POLYMERASE II-ASSOCIATED PROTEIN 1.	X		
IPI00409578	GRIP1	ISOFORM 1 OF GLUTAMATE RECEPTOR-INTERACTING PROTEIN 1.	X		
IPI00409607	CAMSAP1L1	CAMSAP1L1 PROTEIN.	X		
IPI00409652		HERV-F(C)2_7Q36.2 PROVIRUS ANCESTRAL ENV POLYPROTEIN.	X		
IPI00410344	PLK4	SERINE/THREONINE-PROTEIN KINASE PLK4.	X		
IPI00410436	C2orf55	UNCHARACTERIZED PROTEIN C2ORF55.	X		
IPI00410478	FLJ43582	CDNA FLJ43582 FIS, CLONE SKNMC2004643.	X		
IPI00410523	FLJ44815	HYPOTHETICAL PROTEIN LOC400591.	X		
IPI00410550	FLJ45964	CDNA FLJ45964 FIS, CLONE PLACE7014396.	X		
IPI00410687	ZDHHC17	ISOFORM 1 OF PALMITOYLTRANSFERASE ZDHHC17.	X		
IPI00411356	VPS4A	VACUOLAR PROTEIN SORTING-ASSOCIATING PROTEIN 4A.	X		
IPI00411817	ACCS	1-AMINOCYCLOPROPANE-1-CARBOXYLATE SYNTHASE HOMOLOG.	X		
IPI00411886	NOC2L	NUCLEOLAR COMPLEX PROTEIN 2 HOMOLOG.	X		
IPI00412224	BRWD2	BROMODOMAIN AND WD REPEAT-CONTAINING PROTEIN 2.	X		
IPI00412404	SUPV3L1	ATP-DEPENDENT RNA HELICASE SUPV3L1, MITOCHONDRIAL PRECURSOR.	X		
IPI00412740	MAP3K15	ISOFORM 1 OF MITOGEN-ACTIVATED PROTEIN KINASE KINASE KINASE 15.	X		
IPI00412792	BTF3L4	TRANSCRIPTION FACTOR BTF3 HOMOLOG 4.	X		
IPI00413365	ZNF318	ISOFORM 1 OF ZINC FINGER PROTEIN 318.	X		
IPI00413447	APOBEC3D	PROBABLE DNA DC-]DU-EDITING ENZYME APOBEC-3D.	X		
IPI00413611	TOP1	DNA TOPOISOMERASE 1.	X		
IPI00413642	MMP23B	MIFR-2.	X		
IPI00413984	C10orf140	CDNA FLJ45187 FIS, CLONE BRAWH3048548.	X		
IPI00414021	TRIM41	ISOFORM 1 OF TRIPARTITE MOTIF-CONTAINING PROTEIN 41.	X		
IPI00414197	WDR26	ISOFORM 1 OF WD REPEAT-CONTAINING PROTEIN 26.	X		
IPI00414228	LOC139249	UNCHARACTERIZED PROTEIN ENSP00000344398.	X		
IPI00414231	LRP10	ISOFORM 1 OF LOW-DENSITY LIPOPROTEIN RECEPTOR-RELATED PROTEIN 10 PRECURSOR.	X		
IPI00414315	EPS8L2	ISOFORM 1 OF EPIDERMAL GROWTH FACTOR RECEPTOR KINASE SUBSTRATE 8-LIKE PROTEIN 2.	X		
IPI00414321	ADAMTS20	ISOFORM 2 OF ADAMTS-20 PRECURSOR.	X		
IPI00414455	CARD11	CASPASE RECRUITMENT DOMAIN FAMILY, MEMBER 11.	X		
IPI00414651	SLC22A16	SOLUTE CARRIER FAMILY 22 MEMBER 16.	X		
IPI00418142	KRIT1	ISOFORM 1 OF KREV INTERACTION TRAPPED PROTEIN 1.	X		

Table S1. Proteins Identified or Not in MS Patients Compared to Normals and Other Neurologic Disease (OND)					
**IPI**	**Gene**	**Protein Name**	**MS**	**Normal**	**OND**
IPI00418382	OPLAH	5-OXOPROLINASE.	X		
IPI00418422	IGHD	IGHD PROTEIN.	X		
IPI00418499	CMAH	ISOFORM 3 OF CYTIDINE MONOPHOSPHATE-N-ACETYLNEURAMINIC ACID HYDROXYLASE-LIKE PROTEIN.	X		
IPI00418544	LOC619207	SIMILAR TO SCAVENGER-RECEPTOR PROTEIN.	X		
IPI00418546	ZNF28	ISOFORM 1 OF ZINC FINGER PROTEIN 28.	X		
IPI00418591		73 KDA PROTEIN.	X		
IPI00418639	ACAD10	ISOFORM 1 OF ACYL-COA DEHYDROGENASE FAMILY MEMBER 10.	X		
IPI00418705	LOC401351	CDNA FLJ46332 FIS, CLONE TESTI4045470.	X		
IPI00418765	OTOG	ISOFORM 1 OF OTOGELIN PRECURSOR.	X		
IPI00418790	EML5	ECHINODERM MICROTUBULE ASSOCIATED PROTEIN LIKE 5.	X		
IPI00418890	LDLRAD2	LOW-DENSITY LIPOPROTEIN RECEPTOR CLASS A DOMAIN-CONTAINING PROTEIN 2 PRECURSOR.	X		
IPI00419100	FGD6	ISOFORM 1 OF FYVE, RHOGEF AND PH DOMAIN-CONTAINING PROTEIN 6.	X		
IPI00419172	MYL5	CDNA FLJ41980 FIS, CLONE SMINT2007391.	X		
IPI00419234	GK2	GLYCEROL KINASE, TESTIS SPECIFIC 2.	X		
IPI00419440	PODN	PODOCAN.	X		
IPI00419451	TRIM65	TRIPARTITE MOTIF-CONTAINING PROTEIN 65.	X		
IPI00419531	CPSF2	CLEAVAGE AND POLYADENYLATION SPECIFICITY FACTOR SUBUNIT 2.	X		
IPI00419731	CRELD2	ISOFORM 1 OF CYSTEINE-RICH WITH EGF-LIKE DOMAIN PROTEIN 2 PRECURSOR.	X		
IPI00423464	IGHG1	PUTATIVE UNCHARACTERIZED PROTEIN DKFZP686K03196.	X		
IPI00424178	SMOX	ISOFORM 1 OF SPERMINE OXIDASE.	X		
IPI00426060	IGHA1; IGHV3OR16-13	PUTATIVE UNCHARACTERIZED PROTEIN DKFZP686J11235 (FRAGMENT).	X		
IPI00427586	PRKG1	CGMP-DEPENDENT PROTEIN KINASE 1, ALPHA ISOZYME.	X		
IPI00428732	NRXN2	NEUREXIN-2-BETA PRECURSOR.	X		
IPI00429172		HERV-K_1Q22 PROVIRUS ANCESTRAL GAG POLYPROTEIN.	X		
IPI00430634	RIMS2	RAB3-INTERACTING PROTEIN.	X		
IPI00431405	C5orf33	ISOFORM 2 OF UPF0465 PROTEIN C5ORF33.	X		
IPI00432008	ATXN1	ATAXIN-1.	X		
IPI00433160	ZNF711	ZINC FINGER PROTEIN 711.	X		
IPI00434629	FHDC1	FH2 DOMAIN CONTAINING 1.	X		
IPI00435975		FP248.	X		
IPI00436705	MORC3	MORC FAMILY CW-TYPE ZINC FINGER PROTEIN 3.	X		

Table S1. Proteins Identified or Not in MS Patients Compared to Normals and Other Neurologic Disease (OND)					
IPI	Gene	Protein Name	MS	Normal	OND
IPI00437168	PAX5	BSAP SPLICE VARIANT DELTA789.	X		
IPI00442327	FLJ16126	CDNA FLJ16126 FIS, CLONE BRACE2029396.	X		
IPI00442507		CDNA FLJ27112 FIS, CLONE SPL05779.	X		
IPI00442568	LOC644686	CDNA FLJ26926 FIS, CLONE RCT05265.	X		
IPI00442882		CDNA FLJ26446 FIS, CLONE KDN02743.	X		
IPI00443010		CDNA FLJ26108 FIS, CLONE SPL05107.	X		
IPI00443024		CDNA FLJ26089 FIS, CLONE RCT06287.	X		
IPI00443413		CDNA FLJ46317 FIS, CLONE TESTI4041832.	X		
IPI00444086		CDNA FLJ45866 FIS, CLONE OCBBF3001616.	X		
IPI00444141		CDNA FLJ46069 FIS, CLONE TESOP2004110.	X		
IPI00444145	LOC440031	CDNA FLJ45820 FIS, CLONE NT2RP8001407.	X		
IPI00444540		PUTATIVE P150.	X		
IPI00444648		CDNA FLJ45038 FIS, CLONE BRAWH3020200.	X		
IPI00444656	RUSC2	IPORIN.	X		
IPI00444733		CDNA FLJ45387 FIS, CLONE BRHIP3024703.	X		
IPI00444792	C1orf86	33 KDA PROTEIN.	X		
IPI00445152	C18orf54	ISOFORM 2 OF UNCHARACTERIZED PROTEIN C18ORF54 PRECURSOR.	X		
IPI00445371	LOC259308	UNCHARACTERIZED PROTEIN C9ORF144B.	X		
IPI00445531	UPF1	CDNA FLJ43809 FIS, CLONE TESTI4001176, MODERATELY SIMILAR TO REGULATOR OF NONSENSE TRANSCRIPTS 1.	X		
IPI00445705	DENND3	CDNA FLJ43517 FIS, CLONE PERIC2008385.	X		
IPI00445707	MAEA	CDNA FLJ43512 FIS, CLONE PERIC2004028, MODERATELY SIMILAR TO MUS MUSCULUS ERYTHROBLAST MACROPHAGE PROTEIN EMP MRNA.	X		
IPI00445780		CDNA FLJ43098 FIS, CLONE CTONG1000488.	X		
IPI00445972	LOC165186	HYPOTHETICAL PROTEIN LOC165186.	X		
IPI00446376	TIAF1	CDNA FLJ42167 FIS, CLONE THYMU2023967.	X		
IPI00446610		CDNA FLJ41887 FIS, CLONE OCBBF2023643.	X		
IPI00446743	SPRN	SHADOW OF PRION PROTEIN HOMOLOG.	X		
IPI00446866	EVI1	ECOTROPIC VIRUS INTEGRATION SITE 1 PROTEIN HOMOLOG.	X		
IPI00447177		ANTIGEN MLAA-23.	X		
IPI00448734	IGHV7-81	IGHV7-81 PROTEIN.	X		
IPI00448938	IGHG1	IGHG1 PROTEIN.	X		
IPI00449906	ARHGEF7	ISOFORM 1 OF RHO GUANINE NUCLEOTIDE EXCHANGE FACTOR 7.	X		
IPI00451624	CRTAC1	ISOFORM 1 OF CARTILAGE ACIDIC PROTEIN 1 PRECURSOR.	X		

Table S1. Proteins Identified or Not in MS Patients Compared to Normals and Other Neurologic Disease (OND)					
**IPI**	**Gene**	**Protein Name**	**MS**	**Normal**	**OND**
IPI00452728	HES6	ISOFORM 2 OF TRANSCRIPTION COFACTOR HES-6.	X		
IPI00453476		UNCHARACTERIZED PROTEIN ENSP00000348237.	X		
IPI00454612	ERVK6	HERV-K_6Q14.1 PROVIRUS ANCESTRAL GAG-POL POLYPROTEIN.	X		
IPI00455167	AKR1C2	25 KDA PROTEIN.	X		
IPI00455451	NUDT17	NUDIX (NUCLEOSIDE DIPHOSPHATE LINKED MOIETY X)-TYPE MOTIF 17.	X		
IPI00455467	LOC440706	SIMILAR TO TITIN ISOFORM N2-A.	X		
IPI00455618	CLEC6A	C-TYPE LECTIN DOMAIN FAMILY 6 MEMBER A.	X		
IPI00455832	LOC728957	SIMILAR TO ZINC FINGER PROTEIN 75.	X		
IPI00455979	C18orf1	HYPOTHETICAL PROTEIN LOC753 ISOFORM GAMMA 1.	X		
IPI00456004	ATAD2B	ISOFORM 2 OF ATPASE FAMILY AAA DOMAIN-CONTAINING PROTEIN 2B.	X		
IPI00456149	GALNT12	ISOFORM 1 OF POLYPEPTIDE N-ACETYLGALACTOSAMINYLTRANSFERASE 12.	X		
IPI00456626	TXLNB	BETA-TAXILIN.	X		
IPI00456629	ZNF534	ZINC FINGER PROTEIN 534 (FRAGMENT).	X		
IPI00456630	FNDC3A	FIBRONECTIN TYPE III DOMAIN CONTAINING 3A ISOFORM 2.	X		
IPI00456649	KIAA1161	UNCHARACTERIZED FAMILY 31 GLUCOSIDASE KIAA1161.	X		
IPI00456680	KIAA1245; NBPF1; NBPF10; NBPF11; NBPF14; NBPF15; NBPF16; NBPF20; NBPF8; NBPF9	NEUROBLASTOMA BREAKPOINT FAMILY MEMBER 8.	X		
IPI00456707	ERC2	ISOFORM 2 OF ERC PROTEIN 2.	X		
IPI00456722	GARNL1	ISOFORM 2 OF GTPASE-ACTIVATING RAP/RAN-GAP DOMAIN-LIKE 1.	X		
IPI00456759		UNCHARACTERIZED PROTEIN ENSP00000346048.	X		
IPI00457035	LOC401677	SIMILAR TO EUKARYOTIC TRANSLATION ELONGATION FACTOR 1 ALPHA 2.	X		
IPI00464963	HEMGN	HEMOGEN.	X		
IPI00464999	HEATR6	HEAT REPEAT CONTAINING 6.	X		
IPI00465008	KIAA0240	UNCHARACTERIZED PROTEIN KIAA0240.	X		
IPI00465059	RHOT2	ISOFORM 1 OF MITOCHONDRIAL RHO GTPASE 2.	X		
IPI00465102	POLE	DNA POLYMERASE EPSILON CATALYTIC SUBUNIT A.	X		

Table S1. Proteins Identified or Not in MS Patients Compared to Normals and Other Neurologic Disease (OND)					
**IPI**	**Gene**	**Protein Name**	**MS**	**Normal**	**OND**
IPI00465113	EXDL2	EXONUCLEASE 3'-5' DOMAIN-LIKE-CONTAINING PROTEIN 2.	X		
IPI00465270	REP15	RAB15 EFFECTOR PROTEIN.	X		
IPI00470339	LOC653319	46 KDA PROTEIN.	X		
IPI00470388	CADM2	ISOFORM 2 OF CELL ADHESION MOLECULE 2 PRECURSOR.	X		
IPI00470470	AFMID	PROBABLE ARYLFORMAMIDASE.	X		
IPI00470473	NFIB	NUCLEAR FACTOR 1.	X		
IPI00470483	WDR62	WD REPEAT PROTEIN 62.	X		
IPI00470781	LOC90379	DDB1- AND CUL4-ASSOCIATED FACTOR 15.	X		
IPI00472011	NEO1	154 KDA PROTEIN.	X		
IPI00472610	IGHM	IGHM PROTEIN.	X		
IPI00472810	UBE3C	ISOFORM 2 OF UBIQUITIN-PROTEIN LIGASE E3C.	X		
IPI00473006	HLA-A; HLA-A29.1; HLA-B; HLA-C; LOC730410; MICA	HLA CLASS I HISTOCOMPATIBILITY ANTIGEN, CW-17 ALPHA CHAIN PRECURSOR.	X		
IPI00473066	MRC1L1	MANNOSE RECEPTOR, C TYPE 1-LIKE 1.	X		
IPI00477118	FLJ20254	HYPOTHETICAL PROTEIN LOC54867 ISOFORM 1.	X		
IPI00477285	SH3KBP1	SH3-DOMAIN KINASE BINDING PROTEIN 1.	X		
IPI00477535	ERCC5	DNA-REPAIR PROTEIN COMPLEMENTING XP-G CELLS.	X		
IPI00477577	SORBS2	SORBIN AND SH3 DOMAIN CONTAINING 2 ISOFORM 2.	X		
IPI00478254	SLC35A1	CMP-SIALIC ACID TRANSPORTER.	X		
IPI00478600	IGKV1-5	IGKV1-5 PROTEIN.	X		
IPI00478616	FAM111A	PROTEIN FAM111A.	X		
IPI00478685	PRAMEF8	PRAME FAMILY MEMBER 8.	X		
IPI00478832	FBF1	FAS-BINDING FACTOR 1.	X		
IPI00478866	KLF8	KRUEPPEL-LIKE FACTOR 8.	X		
IPI00478896	RPL7A	RIBOSOMAL PROTEIN L7A.	X		
IPI00479304	CNTN1	ISOFORM 3 OF CONTACTIN-1 PRECURSOR.	X		
IPI00479533	ZNF277	ZINC FINGER PROTEIN (C2H2 TYPE) 277.	X		
IPI00479640		CHROMOSOME 1 OPEN READING FRAME 113.	X		
IPI00479708	IGHM	IGHM PROTEIN.	X		
IPI00479760	AAK1	AP2 ASSOCIATED KINASE 1.	X		
IPI00480099	PLA2R1	ISOFORM 2 OF SECRETORY PHOSPHOLIPASE A2 RECEPTOR PRECURSOR.	X		

Table S1. Proteins Identified or Not in MS Patients Compared to Normals and Other Neurologic Disease (OND)					
**IPI**	**Gene**	**Protein Name**	**MS**	**Normal**	**OND**
IPI00480160	VEZT	VEZT PROTEIN.	X		
IPI00513799	TTC22	NOVEL PROTEIN.	X		
IPI00514002	STARD9	ISOFORM 1 OF STAR-RELATED LIPID TRANSFER PROTEIN 9.	X		
IPI00514115	NFAT5	NUCLEAR FACTOR OF ACTIVATED T-CELLS 5 ISOFORM B.	X		
IPI00514688	RABEPK	RAB9 EFFECTOR PROTEIN WITH KELCH MOTIFS.	X		
IPI00514759	GPATCH3	G PATCH DOMAIN-CONTAINING PROTEIN 3.	X		
IPI00514866	ZNF124	ISOFORM 2 OF ZINC FINGER PROTEIN 124.	X		
IPI00514887	GBGT1	GLOBOSIDE ALPHA-1,3-N-ACETYLGALACTOSAMINYLTRANSFERASE 1.	X		
IPI00514956	RABGGTB	RAB GERANYLGERANYLTRANSFERASE, BETA SUBUNIT.	X		
IPI00550020	PTMS	PARATHYMOSIN.	X		
IPI00550021	RPL3	60S RIBOSOMAL PROTEIN L3.	X		
IPI00550090	JMJD3	ISOFORM 1 OF JMJC DOMAIN-CONTAINING PROTEIN 3.	X		
IPI00550811	LBX2	ISOFORM 1 OF TRANSCRIPTION FACTOR LBX2.	X		
IPI00550821	FLJ12529	ISOFORM 1 OF CLEAVAGE AND POLYADENYLATION SPECIFICITY FACTOR SUBUNIT 7.	X		
IPI00552323	IGLV4-60	V5-4 PROTEIN.	X		
IPI00552587	GADD45GIP1	GROWTH ARREST AND DNA-DAMAGE-INDUCIBLE PROTEINS-INTERACTING PROTEIN 1.	X		
IPI00552637		G-PROTEIN COUPLED RECEPTOR.	X		
IPI00552701	DLG3	DISCS, LARGE HOMOLOG 3.	X		
IPI00553148		OTTHUMP00000028750.	X		
IPI00553175	ZGPAT	ISOFORM 1 OF ZINC FINGER CCCH-TYPE WITH G PATCH DOMAIN-CONTAINING PROTEIN.	X		
IPI00554597	ESRRG	ISOFORM 3 OF ESTROGEN-RELATED RECEPTOR GAMMA.	X		
IPI00554660	PIPOX	PEROXISOMAL SARCOSINE OXIDASE.	X		
IPI00554751	NEXN	PUTATIVE UNCHARACTERIZED PROTEIN DKFZP686A0568.	X		
IPI00556004		AAK1 PROTEIN (FRAGMENT).	X		
IPI00556087	SLC19A2	SOLUTE CARRIER FAMILY 19, MEMBER 2 VARIANT (FRAGMENT).	X		
IPI00556440		KV CHANNEL INTERACTING PROTEIN 3 VARIANT (FRAGMENT).	X		
IPI00556514	RBM14	RNA BINDING MOTIF PROTEIN 14 VARIANT (FRAGMENT).	X		
IPI00604504		PUTATIVE UNCHARACTERIZED PROTEIN.	X		
IPI00604624	NIF3L1	ISOFORM 1 OF NIF3-LIKE PROTEIN 1.	X		
IPI00604711	KIF1A	ISOFORM 1 OF KINESIN-LIKE PROTEIN KIF1A.	X		
IPI00640046		PUTATIVE UNCHARACTERIZED PROTEIN.	X		

Table S1. Proteins Identified or Not in MS Patients Compared to Normals and Other Neurologic Disease (OND)					
**IPI**	**Gene**	**Protein Name**	**MS**	**Normal**	**OND**
IPI00640533	HYDIN; HYDIN2	UNCHARACTERIZED PROTEIN HYDIN2.	X		
IPI00640556		UNCHARACTERIZED PROTEIN ENSP00000373106.	X		
IPI00640749	GPAM	GLYCEROL 3-PHOSPHATE ACYLTRANSFERASE, MITOCHONDRIAL.	X		
IPI00641831		15 KDA PROTEIN.	X		
IPI00641908	DBNDD1	25 KDA PROTEIN.	X		
IPI00642760	DNAI2	DNAI2 PROTEIN.	X		
IPI00643305		TRANSTHYRETIN FAMILY PROTEIN.	X		
IPI00643492	C18orf10	28 KDA PROTEIN.	X		
IPI00643605	ANKRD20B	NOVEL PROTEIN.	X		
IPI00644140	ELMO3	85 KDA PROTEIN.	X		
IPI00644286	MGC26718	12 KDA PROTEIN.	X		
IPI00644429		PROTEIN.	X		
IPI00644502	GARNL3	ISOFORM 1 OF GTPASE-ACTIVATING RAP/RAN-GAP DOMAIN-LIKE PROTEIN 3.	X		
IPI00644671	CC2D1B	ISOFORM 1 OF COILED-COIL AND C2 DOMAIN-CONTAINING PROTEIN 1B.	X		
IPI00644868	TNRC4	TRINUCLEOTIDE REPEAT-CONTAINING PROTEIN 4.	X		
IPI00645862	SLC35A2	UNCHARACTERIZED PROTEIN SLC35A2.	X		
IPI00645947	RTTN	ISOFORM 1 OF ROTATIN.	X		
IPI00646010		167 KDA PROTEIN.	X		
IPI00646107	AGER	UNCHARACTERIZED PROTEIN ENSP00000364210.	X		
IPI00647428	E2F3	E2F3 PROTEIN.	X		
IPI00647562	C1orf50	PROTEIN.	X		
IPI00651644	NAPB	CDNA FLJ39175 FIS, CLONE OCBBF2003593, HIGHLY SIMILAR TO BETA-SOLUBLE NSF ATTACHMENT PROTEIN.	X		
IPI00651646	LOC387790	LOC387790 PROTEIN.	X		
IPI00651749		CAPON SHORT FORM.	X		
IPI00657887	MUC19	PROTEIN.	X		
IPI00657953	TRIO	TRIPLE FUNCTIONAL DOMAIN.	X		
IPI00658004	PRTG	PROTOGENIN.	X		
IPI00658153		MUTANT BETA-GLOBIN.	X		
IPI00658196		54 KDA PROTEIN.	X		
IPI00719708	C11orf34	HYPOTHETICAL PROTEIN.	X		
IPI00735530	LOC644066	SIMILAR TO CG31901-PA.	X		
IPI00736435	LOC647654	SIMILAR TO CELL DIVISION CYCLE PROTEIN 27 HOMOLOG.	X		

Table S1. Proteins Identified or Not in MS Patients Compared to Normals and Other Neurologic Disease (OND)					
**IPI**	**Gene**	**Protein Name**	**MS**	**Normal**	**OND**
IPI00736556	KIAA1486	HYPOTHETICAL PROTEIN LOC57624.	X		
IPI00737214	LOC644717	SIMILAR TO SARCOMA ANTIGEN 1.	X		
IPI00737385	LOC647102	SIMILAR TO ELK1.	X		
IPI00737518	TMEM201	SIMILAR TO CG7744-PA.	X		
IPI00737545	SGK269	TYROSINE-PROTEIN KINASE SGK269.	X		
IPI00737871	KIAA0754	HYPOTHETICAL PROTEIN LOC643314.	X		
IPI00738052		SIMILAR TO RIBOSOMAL PROTEIN S3A; 40S RIBOSOMAL PROTEIN S3A; V-FOS TRANSFORMATION EFFECTOR PROTEIN 1.	X		
IPI00738367	ZC3H12C	ZINC FINGER CCCH-TYPE CONTAINING 12C.	X		
IPI00739386	PRAGMIN	TYROSINE-PROTEIN KINASE SGK223.	X		
IPI00739565	UNC13C	PROTEIN UNC-13 HOMOLOG C.	X		
IPI00739697	LOC645852	SIMILAR TO SPLICING FACTOR 3A, SUBUNIT 2, 66KDA.	X		
IPI00740057	ANKRD31	SIMILAR TO ANKYRIN REPEAT DOMAIN-CONTAINING PROTEIN 11.	X		
IPI00741637	LOC652455	SIMILAR TO TRANSCRIPTIONAL REGULATOR ATRX.	X		
IPI00742210	LOC647275	SIMILAR TO PURINE NUCLEOSIDE PHOSPHORYLASE.	X		
IPI00742661	COQ5	ISOFORM 2 OF UBIQUINONE BIOSYNTHESIS METHYLTRANSFERASE COQ5, MITOCHONDRIAL PRECURSOR.	X		
IPI00743102	LOC642648	CDNA FLJ41389 FIS, CLONE BRCAN2024563.	X		
IPI00743123		CONSERVED HYPOTHETICAL PROTEIN.	X		
IPI00743555		CDNA FLJ44898 FIS, CLONE BRAMY3002508.	X		
IPI00743623	AK5	PUTATIVE UNCHARACTERIZED PROTEIN.	X		
IPI00743663	TRAF6	TNF RECEPTOR-ASSOCIATED FACTOR 6.	X		
IPI00743691	PTPRU	FMI PROTEIN.	X		
IPI00744194		SIMILAR TO SODIUM/POTASSIUM-TRANSPORTING ATPASE ALPHA-1 CHAIN PRECURSOR.	X		
IPI00744317		UNCHARACTERIZED PROTEIN ENSP00000366447 (FRAGMENT).	X		
IPI00744423	LAYN	ISOFORM 2 OF LAYILIN PRECURSOR.	X		
IPI00744530		CONSERVED HYPOTHETICAL PROTEIN.	X		
IPI00744575	USP9Y	ISOFORM SHORT OF PROBABLE UBIQUITIN CARBOXYL-TERMINAL HYDROLASE FAF-Y.	X		
IPI00744742		CONSERVED HYPOTHETICAL PROTEIN.	X		
IPI00744762		CONSERVED HYPOTHETICAL PROTEIN.	X		
IPI00744933		SIMILAR TO SYNTAXIN 17.	X		
IPI00745149	CD2AP	CONSERVED HYPOTHETICAL PROTEIN.	X		

Table S1. Proteins Identified or Not in MS Patients Compared to Normals and Other Neurologic Disease (OND)					
**IPI**	**Gene**	**Protein Name**	**MS**	**Normal**	**OND**
IPI00745471	SYNE1	SPECTRIN-LIKE PROTEIN OF THE NUCLEAR ENVELOPE AND GOLGI.	X		
IPI00745746	PDZD2	ISOFORM 1 OF PDZ DOMAIN-CONTAINING PROTEIN 2.	X		
IPI00745839	LOC644925	CONSERVED HYPOTHETICAL PROTEIN.	X		
IPI00746124	SLC6A15	TRANSPORTER.	X		
IPI00746675	NANOS3	NANOS HOMOLOG 3.	X		
IPI00746965		CONSERVED HYPOTHETICAL PROTEIN.	X		
IPI00747084	MEIS3P1	UNCHARACTERIZED PROTEIN ENSP00000368940.	X		
IPI00747222		SIMILAR TO GRAINYHEAD-LIKE PROTEIN 1 HOMOLOG.	X		
IPI00747459	PDE6C	CONE CGMP-SPECIFIC 3',5'-CYCLIC PHOSPHODIESTERASE SUBUNIT ALPHA'.	X		
IPI00747470	JMJD1A	PUTATIVE ZINC FINGER PROTEIN.	X		
IPI00747501	OR2J4P	SEVEN TRANSMEMBRANE HELIX RECEPTOR.	X		
IPI00747950	TNC	PUTATIVE UNCHARACTERIZED PROTEIN DKFZP686B15117.	X		
IPI00748003	CCDC112	COILED-COIL DOMAIN CONTAINING 112 ISOFORM 1.	X		
IPI00748334		12 KDA PROTEIN.	X		
IPI00748360	KIAA1797	UNCHARACTERIZED PROTEIN KIAA1797.	X		
IPI00748705	LOC643988	CONSERVED HYPOTHETICAL PROTEIN.	X		
IPI00748708		UNCHARACTERIZED PROTEIN ENSP00000370274.	X		
IPI00748866	hCG_1983896	CDNA FLJ32790 FIS, CLONE TESTI2002361.	X		
IPI00748918	FLJ46358	SIMILAR TO ANKYRIN REPEAT DOMAIN-CONTAINING PROTEIN 36A.	X		
IPI00749116		CONSERVED HYPOTHETICAL PROTEIN.	X		
IPI00749433	DRD4	DOPAMINE RECEPTOR D4 (FRAGMENT).	X		
IPI00759479	IQSEC2	ISOFORM 1 OF IQ MOTIF AND SEC7 DOMAIN-CONTAINING PROTEIN 2.	X		
IPI00759493	SUCLG1	SUCCINATE-COA LIGASE, GDP-FORMING, ALPHA SUBUNIT.	X		
IPI00760547	MPZL1	ISOFORM 2 OF MYELIN PROTEIN ZERO-LIKE PROTEIN 1 PRECURSOR.	X		
IPI00760925	MYO18A	ISOFORM 3 OF MYOSIN-XVIIIA.	X		
IPI00761082	PRR6	ISOFORM 1 OF PROLINE-RICH PROTEIN 6.	X		
IPI00783185	FLJ39660	HYPOTHETICAL PROTEIN LOC284992.	X		
IPI00783234	PTGER3	PTGER3 PROTEIN.	X		
IPI00783290	EFCAB4B	SIMILAR TO 4R79.2.	X		
IPI00783411	DEAF1	SUPPRESSIN VARIANT.	X		
IPI00783641	TXNRD1	THIOREDOXIN REDUCTASE 1 ISOFORM 3.	X		
IPI00783847	SLC25A42	SOLUTE CARRIER FAMILY 25 MEMBER 42.	X		
IPI00784002	SACS	ISOFORM 2 OF SACSIN.	X		
IPI00784159	ANKRD13D	SIMILAR TO ANKYRIN REPEAT DOMAIN 13 FAMILY, MEMBER D.	X		

Table S1. Proteins Identified or Not in MS Patients Compared to Normals and Other Neurologic Disease (OND)					
**IPI**	**Gene**	**Protein Name**	**MS**	**Normal**	**OND**
IPI00784224	ZFR	ZINC FINGER RNA BINDING PROTEIN.	X		
IPI00784373	ACACB	277 KDA PROTEIN.	X		
IPI00784713		PUTATIVE UNCHARACTERIZED PROTEIN.	X		
IPI00784817	IGHV4-31	ANTI-RHD MONOCLONAL T125 GAMMA1 HEAVY CHAIN PRECURSOR.	X		
IPI00784935	IGL@	IGL@ PROTEIN.	X		
IPI00785100	MEF2D	MYOCYTE ENHANCER FACTOR 2D/DELETED IN AZOOSPERMIA ASSOCIATED PROTEIN 1 FUSION PROTEIN.	X		
IPI00786982	PGAM5	SIMILAR TO PHOSPHOGLYCERATE MUTASE FAMILY MEMBER 5.	X		
IPI00787002	LOC729670	SIMILAR TO BCL2-LIKE 12 ISOFORM 1.	X		
IPI00787142	LOC647259	SIMILAR TO SEQUESTOSOME 1 ISOFORM 1.	X		
IPI00787154	GUCY2E	SIMILAR TO OLFACTORY GUANYLYL CYCLASE GC-D PRECURSOR.	X		
IPI00787193	LOC729900	SIMILAR TO GOLGIN SUBFAMILY A MEMBER 2.	X		
IPI00787224	LOC731384	SIMILAR TO COLLAGEN ALPHA-1(II) CHAIN PRECURSOR.	X		
IPI00787356	LOC389654	SIMILAR TO PATCHED.	X		
IPI00787418	LOC731764	SIMILAR TO K08E3.2.	X		
IPI00787449	LOC729789	SIMILAR TO 40S RIBOSOMAL PROTEIN S15.	X		
IPI00787508	LOC732406	SIMILAR TO DOUBLE HOMEOBOX 4C.	X		
IPI00787693	LOC728169	SIMILAR TO DOUBLE HOMEOBOX, 4.	X		
IPI00787743	LOC731813	RHO GTPASE-ACTIVATING PROTEIN.	X		
IPI00787773	KIAA1549	HYPOTHETICAL PROTEIN LOC57670.	X		
IPI00787864	ECT2	EPITHELIAL CELL TRANSFORMING SEQUENCE 2 ONCOGENE PROTEIN SPLICE VARIANT B.	X		
IPI00788232		SIMILAR TO DESTRIN.	X		
IPI00788247	KIF26A	KINESIN-LIKE PROTEIN KIF26A.	X		
IPI00789092		7 KDA PROTEIN.	X		
IPI00789523	DSCR3	UNCHARACTERIZED PROTEIN DSCR3.	X		
IPI00789670	FAM109A	ISOFORM 3 OF PROTEIN FAM109A.	X		
IPI00789751		9 KDA PROTEIN.	X		
IPI00789949	GRM5	PROTEIN.	X		
IPI00790414	RAB8B	10 KDA PROTEIN.	X		
IPI00790928		11 KDA PROTEIN.	X		
IPI00790967	KLRC2	6 KDA PROTEIN.	X		
IPI00791079	C20orf174	UNCHARACTERIZED PROTEIN C20ORF174.	X		
IPI00791158		HYPOTHETICAL PROTEIN.	X		

Table S1. Proteins Identified or Not in MS Patients Compared to Normals and Other Neurologic Disease (OND)					
**IPI**	**Gene**	**Protein Name**	**MS**	**Normal**	**OND**
IPI00791573	SUGT1	ISOFORM 2 OF SUPPRESSOR OF G2 ALLELE OF SKP1 HOMOLOG.	X		
IPI00791634	PHB	PROHIBITIN VARIANT (FRAGMENT).	X		
IPI00792026	KIAA0692	9 KDA PROTEIN.	X		
IPI00792817	PKM2	24 KDA PROTEIN.	X		
IPI00792926		19 KDA PROTEIN.	X		
IPI00792956	CAMKV	35 KDA PROTEIN.	X		
IPI00793212	MVK	28 KDA PROTEIN.	X		
IPI00793233	CCDC144A; CCDC144B; LOC652491; LOC731479	37 KDA PROTEIN.	X		
IPI00793955	CCDC64	6 KDA PROTEIN.	X		
IPI00794143	C12orf65	HYPOTHETICAL SHORT PROTEIN.	X		
IPI00794205		26 KDA PROTEIN.	X		
IPI00794720	CAPNS1	34 KDA PROTEIN.	X		
IPI00794768	EDEM1	5 KDA PROTEIN.	X		
IPI00794948		8 KDA PROTEIN.	X		
IPI00795234	STX1A	UNCHARACTERIZED PROTEIN STX1A.	X		
IPI00795679	CD47	PROTEIN.	X		
IPI00795768	UQCRC1	10 KDA PROTEIN.	X		
IPI00795930	IL1RL2	INTERLEUKIN-1 RECEPTOR-LIKE 2 PRECURSOR.	X		
IPI00796198	PSMB6	12 KDA PROTEIN.	X		
IPI00796305		HYPOTHETICAL PROTEIN.	X		
IPI00796830	A2M	13 KDA PROTEIN.	X		
IPI00797033	SEC22A	4 KDA PROTEIN.	X		
IPI00797525	TDRD12	ISOFORM 1 OF TUDOR DOMAIN-CONTAINING PROTEIN 12.	X		
IPI00798359	SHANK2	35 KDA PROTEIN.	X		
IPI00798376	OSGIN2	62 KDA PROTEIN.	X		
IPI00807481	LOC442132	HYPOTHETICAL PROTEIN LOC442132.	X		
IPI00807497	LOC647979	HYPOTHETICAL PROTEIN ISOFORM 2.	X		
IPI00807519	LOC653056; LOC729028	HYPOTHETICAL PROTEIN.	X		
IPI00815801		RHEUMATOID FACTOR C6 HEAVY CHAIN (FRAGMENT).	X		
IPI00816314	IGHM	PUTATIVE UNCHARACTERIZED PROTEIN DKFZP686I15196.	X		

Table S1. Proteins Identified or Not in MS Patients Compared to Normals and Other Neurologic Disease (OND)					
**IPI**	**Gene**	**Protein Name**	**MS**	**Normal**	**OND**
IPI00827576		VH6DJ PROTEIN (FRAGMENT).	X		
IPI00827589		HEAVY CHAIN VARIABLE REGION (FRAGMENT).	X		
IPI00827646		RHEUMATOID FACTOR RF-IP16 (FRAGMENT).	X		
IPI00827776		VL87-2 PROTEIN (FRAGMENT).	X		
IPI00827826		COLD AGGLUTININ FS-2 L-CHAIN (FRAGMENT).	X		
IPI00827861	PCDH15	PROTOCADHERIN 15.	X		
IPI00828046	LOC728531	SIMILAR TO SEPTIN 7 ISOFORM 1.	X		
IPI00829606		UNCHARACTERIZED PROTEIN ENSP00000374871.	X		
IPI00829624		UNCHARACTERIZED PROTEIN ENSP00000375032 (FRAGMENT).	X		
IPI00829709		UNCHARACTERIZED PROTEIN ENSP00000374946.	X		
IPI00829739	ROBO1	ROBO1 PROTEIN.	X		
IPI00829902		UNCHARACTERIZED PROTEIN ENSP00000375037.	X		
IPI00829910		UNCHARACTERIZED PROTEIN ENSP00000374930.	X		
IPI00829914		PUTATIVE UNCHARACTERIZED PROTEIN.	X		
IPI00829944	IGHG1	IGHG1 PROTEIN.	X		
IPI00844088		LOC392621.	X		
IPI00844119		LOC401367.	X		
IPI00844510	VPS72	VACUOLAR PROTEIN SORTING 72 HOMOLOG.	X		
IPI00847513		CONSERVED HYPOTHETICAL PROTEIN.	X		
IPI00847590		CONSERVED HYPOTHETICAL PROTEIN.	X		
IPI00847597		CONSERVED HYPOTHETICAL PROTEIN.	X		
IPI00847760		RNA-DIRECTED DNA POLYMERASE (REVERSE TRANSCRIPTASE) DOMAIN CONTAINING PROTEIN.	X		
IPI00852573	HFM1	UNCHARACTERIZED PROTEIN HFM1.	X		
IPI00852643	KIAA1109	HYPOTHETICAL PROTEIN LOC84162.	X		
IPI00852806	EXOC6B	SEC15-LIKE 2.	X		
IPI00853083	LOC100101267	POM121 MEMBRANE GLYCOPROTEIN (RAT)-LIKE.	X		
IPI00853270	JAKMIP1	JANUS KINASE AND MICROTUBULE INTERACTING PROTEIN 1 ISOFORM 1.	X		
IPI00853627	KIAA1430	ISOFORM 1 OF UPF0501 PROTEIN KIAA1430.	X		
IPI00855758	LOC648999	SIMILAR TO CG3764-PA.	X		
IPI00855916		TRANSTHYRETIN.	X		
IPI00855985	MAP3K1	MITOGEN-ACTIVATED PROTEIN KINASE KINASE KINASE 1.	X		
IPI00856014	FLJ40298	HYPOTHETICAL PROTEIN LOC129852.	X		

Table S1. Proteins Identified or Not in MS Patients Compared to Normals and Other Neurologic Disease (OND)					
**IPI**	**Gene**	**Protein Name**	**MS**	**Normal**	**OND**
IPI00856095	LOC145814	UNCHARACTERIZED PROTEIN ENSP00000368199.	X		
IPI00856098		P180/RIBOSOME RECEPTOR.	X		
IPI00868787	TANC2	TETRATRICOPEPTIDE REPEAT, ANKYRIN REPEAT AND COILED-COIL CONTAINING 2.	X		
IPI00871695	DEK	48 KDA PROTEIN.	X		
IPI00871803		30 KDA PROTEIN.	X		
IPI00872262	SHANK3	SHANK3 (FRAGMENT).	X		
IPI00872274		UNCHARACTERIZED PROTEIN ENSP00000382267.	X		
IPI00872287	LOC729830	HYPOTHETICAL PROTEIN LOC729830.	X		
IPI00873112		NUCLEAR PORE MEMBRANE PROTEIN 121 (FRAGMENT).	X		
IPI00873155		UNCHARACTERIZED PROTEIN ENSP00000346720.	X		
IPI00873189	SLC35B2	51 KDA PROTEIN.	X		
IPI00873427		UNCHARACTERIZED PROTEIN ENSP00000351411.	X		
IPI00873576	LOC646262	HYPOTHETICAL PROTEIN.	X		
IPI00873735	OR10C1	UNCHARACTERIZED PROTEIN ENSP00000373045 (FRAGMENT).	X		
IPI00874167		UNCHARACTERIZED PROTEIN ENSP00000383207 (FRAGMENT).	X		
IPI00874282		564 KDA PROTEIN.	X		
IPI00876888		CDNA FLJ78387.	X		
IPI00876912	LOC644264	SIMILAR TO FETAL ALZHEIMER ANTIGEN ISOFORM 2.	X		
IPI00876992	MAGIX	MAGI FAMILY MEMBER, X-LINKED ISOFORM B.	X		
IPI00877821		29 KDA PROTEIN.	X		
IPI00878017		38 KDA PROTEIN.	X		
IPI00878025		3 KDA PROTEIN.	X		
IPI00878148		154 KDA PROTEIN.	X		
IPI00878214		12 KDA PROTEIN.	X		
IPI00878600	POLR2F	PROTEIN.	X		
IPI00878620		13 KDA PROTEIN.	X		
IPI00878838		37 KDA PROTEIN.	X		
IPI00878876	SNRPD3	13 KDA PROTEIN.	X		
IPI00879014		75 KDA PROTEIN.	X		
IPI00879148		18 KDA PROTEIN.	X		
IPI00879225	LOC440258	PUTATIVE UNCHARACTERIZED PROTEIN.	X		
IPI00879452		8 KDA PROTEIN.	X		
IPI00879559		17 KDA PROTEIN.	X		
IPI00879809		12 KDA PROTEIN.	X		

Table S1. Proteins Identified or Not in MS Patients Compared to Normals and Other Neurologic Disease (OND)					
**IPI**	**Gene**	**Protein Name**	**MS**	**Normal**	**OND**
IPI00879988		29 KDA PROTEIN.	X		
IPI00883599	MRC1	MANNOSE RECEPTOR.	X		
IPI00884133	LOC401561	CONSERVED HYPOTHETICAL PROTEIN.	X		
IPI00884135		SIMILAR TO VH-3 FAMILY (VH26)D/J PROTEIN.	X		
IPI00884147		CONSERVED HYPOTHETICAL PROTEIN.	X		
IPI00884179		PROTEIN OF UNKNOWN FUNCTION DUF1725 DOMAIN CONTAINING PROTEIN.	X		
IPI00884390		CONSERVED HYPOTHETICAL PROTEIN.	X		
IPI00884402		CONSERVED HYPOTHETICAL PROTEIN.	X		
IPI00884436		SIMILAR TO BETA-HEMOGLOBIN.	X		
IPI00884437		SIMILAR TO SYNTAXIN-BINDING PROTEIN 1.	X		
IPI00884440	MIA3	SIMILAR TO CTAGE FAMILY, MEMBER 5 ISOFORM 1 ISOFORM 11.	X		
IPI00000087	SCN2B	SODIUM CHANNEL SUBUNIT BETA-2 PRECURSOR.		X	X
IPI00000104	RNGTT	ISOFORM 1 OF MRNA-CAPPING ENZYME.		X	X
IPI00000265	C10orf38	UNCHARACTERIZED PROTEIN C10ORF38 PRECURSOR.		X	X
IPI00000760	DDAH2	N(G),N(G)-DIMETHYLARGININE DIMETHYLAMINOHYDROLASE 2.		X	X
IPI00000871	PRL	PROLACTIN.		X	X
IPI00000914	CALCA	ISOFORM 1 OF CALCITONIN PRECURSOR.		X	X
IPI00001120	LINGO2	CDNA FLJ31810 FIS, CLONE NT2RI2009289, WEAKLY SIMILAR TO CARBOXYPEPTIDASE N 83 KD CHAIN.		X	X
IPI00001755	GPC6	GLYPICAN-6 PRECURSOR.		X	X
IPI00001863	WIF1	WNT INHIBITORY FACTOR 1 PRECURSOR.		X	X
IPI00001893	PCDH7	ISOFORM A OF PROTOCADHERIN-7 PRECURSOR.		X	X
IPI00002283	PTCHD2	ISOFORM 2 OF PATCHED DOMAIN-CONTAINING PROTEIN 2.		X	X
IPI00002412	PPT1	PALMITOYL-PROTEIN THIOESTERASE 1 PRECURSOR.		X	X
IPI00002459	ANXA6	ANNEXIN VI ISOFORM 2.		X	X
IPI00002535	FKBP2	FK506-BINDING PROTEIN 2 PRECURSOR.		X	X
IPI00003031	ISOC2	ISOFORM 2 OF ISOCHORISMATASE DOMAIN-CONTAINING PROTEIN 2, MITOCHONDRIAL PRECURSOR.		X	X
IPI00003441	C1orf9	ISOFORM 1 OF PROTEIN C1ORF9 PRECURSOR.		X	X
IPI00003470		IG KAPPA CHAIN V-I REGION WES.		X	X
IPI00003527	SLC9A3R1	EZRIN-RADIXIN-MOESIN-BINDING PHOSPHOPROTEIN 50.		X	X
IPI00003799	HEBP2	ISOFORM 2 OF HEME-BINDING PROTEIN 2.		X	X
IPI00003933	HAGH	HYDROXYACYL GLUTATHIONE HYDROLASE ISOFORM 1.		X	X
IPI00003935	HIST2H2BE	HISTONE H2B TYPE 2-E.		X	X

Table S1. Proteins Identified or Not in MS Patients Compared to Normals and Other Neurologic Disease (OND)					
**IPI**	**Gene**	**Protein Name**	**MS**	**Normal**	**OND**
IPI00004047	EXT2	ISOFORM 1 OF EXOSTOSIN-2.		X	X
IPI00004084	CREBL1	ISOFORM 2 OF CYCLIC AMP-DEPENDENT TRANSCRIPTION FACTOR ATF-6 BETA.		X	X
IPI00004457	AOC3	MEMBRANE COPPER AMINE OXIDASE.		X	X
IPI00004480	ADAMDEC1	ADAM DEC1 PRECURSOR.		X	X
IPI00004494	SEMA3E	SEMAPHORIN-3E PRECURSOR.		X	X
IPI00005038	HRSP12	RIBONUCLEASE UK114.		X	X
IPI00005128	ANGPT2	ISOFORM 1 OF ANGIOPOIETIN-2 PRECURSOR.		X	X
IPI00005129	SCAMP1	ISOFORM 1 OF SECRETORY CARRIER-ASSOCIATED MEMBRANE PROTEIN 1.		X	X
IPI00005153	OBP2A	ISOFORM AA OF ODORANT-BINDING PROTEIN 2A PRECURSOR.		X	X
IPI00005158	LONP1	LON PROTEASE HOMOLOG, MITOCHONDRIAL PRECURSOR.		X	X
IPI00005159	ACTR2	ACTIN-RELATED PROTEIN 2.		X	X
IPI00005564	STC1	STANNIOCALCIN-1 PRECURSOR.		X	X
IPI00005605	NDRG3	ISOFORM 1 OF PROTEIN NDRG3.		X	X
IPI00005652	WSCD2	ISOFORM 1 OF WSC DOMAIN-CONTAINING PROTEIN 2.		X	X
IPI00005690	MATN3	MATRILIN-3 PRECURSOR.		X	X
IPI00005722	FLT3	TYROSINE-PROTEIN KINASE RECEPTOR.		X	X
IPI00006034	CRIP2	CYSTEINE-RICH PROTEIN 2.		X	X
IPI00006146	SAA1; SAA2	SERUM AMYLOID A2.		X	X
IPI00006470	HMP19	NEURON-SPECIFIC PROTEIN FAMILY MEMBER 2.		X	X
IPI00006510	TUBB1	TUBULIN BETA-1 CHAIN.		X	X
IPI00006556	KIAA0644	HYPOTHETICAL PROTEIN LOC9865.		X	X
IPI00006987	DDX24	ATP-DEPENDENT RNA HELICASE DDX24.		X	X
IPI00007193	ANKRD26	ISOFORM 2 OF ANKYRIN REPEAT DOMAIN-CONTAINING PROTEIN 26.		X	X
IPI00007240	F13B	COAGULATION FACTOR XIII B CHAIN PRECURSOR.		X	X
IPI00007249	ENPP4	ECTONUCLEOTIDE PYROPHOSPHATASE/PHOSPHODIESTERASE 4.		X	X
IPI00007425	DSC1	DESMOCOLLIN 1 ISOFORM DSC1B PREPROPROTEIN.		X	X
IPI00007702	HSPA2	HEAT SHOCK-RELATED 70 KDA PROTEIN 2.		X	X
IPI00007750	TUBA4A	TUBULIN ALPHA-4A CHAIN.		X	X
IPI00007752	TUBB2C	TUBULIN BETA-2C CHAIN.		X	X
IPI00007798	TRHDE	THYROTROPIN-RELEASING HORMONE-DEGRADING ECTOENZYME.		X	X
IPI00008164	PREP	PROLYL ENDOPEPTIDASE.		X	X
IPI00008215	ME1	NADP-DEPENDENT MALIC ENZYME.		X	X
IPI00008223	RAD23B	UV EXCISION REPAIR PROTEIN RAD23 HOMOLOG B.		X	X
IPI00008274	CAP1	ADENYLYL CYCLASE-ASSOCIATED PROTEIN 1.		X	X

Table S1. Proteins Identified or Not in MS Patients Compared to Normals and Other Neurologic Disease (OND)					
**IPI**	**Gene**	**Protein Name**	**MS**	**Normal**	**OND**
IPI00008504	CA14	CARBONIC ANHYDRASE 14 PRECURSOR.		X	X
IPI00008726	IREB2	IRON-RESPONSIVE ELEMENT-BINDING PROTEIN 2.		X	X
IPI00009111	TPBG	TROPHOBLAST GLYCOPROTEIN PRECURSOR.		X	X
IPI00009148	NUDT3	DIPHOSPHOINOSITOL POLYPHOSPHATE PHOSPHOHYDROLASE 1.		X	X
IPI00009365	C14orf112	COX16-LIKE PROTEIN C14ORF112, MITOCHONDRIAL PRECURSOR.		X	X
IPI00009771	LMNB2	LAMIN-B2.		X	X
IPI00009826	CPB1	CARBOXYPEPTIDASE B PRECURSOR.		X	X
IPI00009828	RXFP3	RELAXIN-3 RECEPTOR 1.		X	X
IPI00009890	SERPINE2	GLIA-DERIVED NEXIN PRECURSOR.		X	X
IPI00009943	TPT1	TUMOR PROTEIN, TRANSLATIONALLY-CONTROLLED 1.		X	X
IPI00010133	CORO1A	CORONIN-1A.		X	X
IPI00010207	UFM1	UBIQUITIN-FOLD MODIFIER 1 PRECURSOR.		X	X
IPI00010274	TPSAB1	ISOFORM 1 OF TRYPTASE ALPHA-1 PRECURSOR.		X	X
IPI00010369	TEX15	TESTIS-EXPRESSED SEQUENCE 15 PROTEIN.		X	X
IPI00010470	SNAP25	ISOFORM SNAP-25B OF SYNAPTOSOMAL-ASSOCIATED PROTEIN 25.		X	X
IPI00010810	ETFA	ELECTRON TRANSFER FLAVOPROTEIN SUBUNIT ALPHA, MITOCHONDRIAL PRECURSOR.		X	X
IPI00010863	ATOX1	COPPER TRANSPORT PROTEIN ATOX1.		X	X
IPI00010896	CLIC1	CHLORIDE INTRACELLULAR CHANNEL PROTEIN 1.		X	X
IPI00011094	C1QTNF4	COMPLEMENT C1Q TUMOR NECROSIS FACTOR-RELATED PROTEIN 4 PRECURSOR.		X	X
IPI00011400	TIAM1	T-LYMPHOMA INVASION AND METASTASIS-INDUCING PROTEIN 1.		X	X
IPI00011515	PACSIN1	PROTEIN KINASE C AND CASEIN KINASE SUBSTRATE IN NEURONS PROTEIN 1.		X	X
IPI00011592	DYNC1LI2	CYTOPLASMIC DYNEIN 1 LIGHT INTERMEDIATE CHAIN 2.		X	X
IPI00011781	C20orf86	ANKYRIN REPEAT-CONTAINING PROTEIN C20ORF86 PRECURSOR.		X	X
IPI00012007	AHCY	ADENOSYLHOMOCYSTEINASE.		X	X
IPI00012009	CSF2RA	ISOFORM 1 OF GRANULOCYTE-MACROPHAGE COLONY-STIMULATING FACTOR RECEPTOR ALPHA CHAIN PRECURSOR.		X	X
IPI00012048	NME1; NME2	NUCLEOSIDE DIPHOSPHATE KINASE A.		X	X
IPI00012283	SEMA3B	ISOFORM 1 OF SEMAPHORIN-3B PRECURSOR.		X	X
IPI00012792	CDH5	CADHERIN-5 PRECURSOR.		X	X
IPI00012877	IFNAR1	ISOFORM 1 OF INTERFERON-ALPHA/BETA RECEPTOR ALPHA CHAIN PRECURSOR.		X	X
IPI00012948	HBEGF	PROHEPARIN-BINDING EGF-LIKE GROWTH FACTOR PRECURSOR.		X	X
IPI00013216	ORC2L	ORIGIN RECOGNITION COMPLEX SUBUNIT 2.		X	X
IPI00013272	GOLGA4	ISOFORM 1 OF GOLGIN SUBFAMILY A MEMBER 4.		X	X

Table S1. Proteins Identified or Not in MS Patients Compared to Normals and Other Neurologic Disease (OND)					
**IPI**	**Gene**	**Protein Name**	**MS**	**Normal**	**OND**
IPI00013281	FKRP	FUKUTIN-RELATED PROTEIN.		X	X
IPI00013302	ADAM15	ADAM 15 PRECURSOR.		X	X
IPI00013319	RAPSN	ISOFORM 2 OF 43 KDA RECEPTOR-ASSOCIATED PROTEIN OF THE SYNAPSE.		X	X
IPI00013475	TUBB2A	TUBULIN BETA-2A CHAIN.		X	X
IPI00013860	HIBADH	3-HYDROXYISOBUTYRATE DEHYDROGENASE, MITOCHONDRIAL PRECURSOR.		X	X
IPI00013978	SPOP	SPECKLE-TYPE POZ PROTEIN.		X	X
IPI00014223	LRRC4C	NETRIN-G1 LIGAND PRECURSOR.		X	X
IPI00014398	FHL1	FOUR AND A HALF LIM DOMAINS 1 VARIANT.		X	X
IPI00014850	PEA15	ASTROCYTIC PHOSPHOPROTEIN PEA-15.		X	X
IPI00017334	PHB	PROHIBITIN.		X	X
IPI00017529	CD58	ISOFORM 1 OF LYMPHOCYTE FUNCTION-ASSOCIATED ANTIGEN 3 PRECURSOR.		X	X
IPI00017745	TIMP4	METALLOPROTEINASE INHIBITOR 4 PRECURSOR.		X	X
IPI00018246	HK1	ISOFORM 1 OF HEXOKINASE-1.		X	X
IPI00018311	NPTN	ISOFORM 2 OF NEUROPLASTIN PRECURSOR.		X	X
IPI00018342	AK1	ADENYLATE KINASE ISOENZYME 1.		X	X
IPI00018352	UCHL1	UBIQUITIN CARBOXYL-TERMINAL HYDROLASE ISOZYME L1.		X	X
IPI00018381	TLL1	ISOFORM 1 OF TOLLOID-LIKE PROTEIN 1 PRECURSOR.		X	X
IPI00018708	CEP63	ISOFORM 2 OF CENTROSOMAL PROTEIN OF 63 KDA.		X	X
IPI00018860	ULBP2	NKG2D LIGAND 2 PRECURSOR.		X	X
IPI00018980	SCN1B	SODIUM CHANNEL SUBUNIT BETA-1 PRECURSOR.		X	X
IPI00019180	GPC5	GLYPICAN-5 PRECURSOR.		X	X
IPI00019190	MYOC	MYOCILIN PRECURSOR.		X	X
IPI00019449	RNASE2	NON-SECRETORY RIBONUCLEASE PRECURSOR.		X	X
IPI00019485	ECHDC2	ISOFORM 2 OF ENOYL-COA HYDRATASE DOMAIN-CONTAINING PROTEIN 2, MITOCHONDRIAL PRECURSOR.		X	X
IPI00019530	TIE1	TYROSINE-PROTEIN KINASE RECEPTOR TIE-1 PRECURSOR.		X	X
IPI00019600	UBE2V2	UBIQUITIN-CONJUGATING ENZYME E2 VARIANT 2.		X	X
IPI00019812	PPP5C	SERINE/THREONINE-PROTEIN PHOSPHATASE 5.		X	X
IPI00019862	BTN2A1	BUTYROPHILIN, SUBFAMILY 2, MEMBER A1 ISOFORM 2 PRECURSOR.		X	X
IPI00019907	GPC3	GLYPICAN-3 PRECURSOR.		X	X
IPI00020201	ST8SIA4	CMP-N-ACETYLNEURAMINATE-POLY-ALPHA-2,8-SIALYLTRANSFERASE.		X	X
IPI00020329	KCNS2	POTASSIUM VOLTAGE-GATED CHANNEL SUBFAMILY S MEMBER 2.		X	X
IPI00020407	MGAT5	ALPHA-1,6-MANNOSYLGLYCOPROTEIN 6-BETA-N-ACETYLGLUCOSAMINYLTRANSFERASE A.		X	X

Table S1. Proteins Identified or Not in MS Patients Compared to Normals and Other Neurologic Disease (OND)					
**IPI**	**Gene**	**Protein Name**	**MS**	**Normal**	**OND**
IPI00020672	BBS1; DPP3	ISOFORM 1 OF DIPEPTIDYL-PEPTIDASE 3.		X	X
IPI00020692	SCN3A	ISOFORM 1 OF SODIUM CHANNEL PROTEIN TYPE 3 SUBUNIT ALPHA.		X	X
IPI00020906	IMPA1	INOSITOL MONOPHOSPHATASE.		X	X
IPI00021048	FER1L3	ISOFORM 1 OF MYOFERLIN.		X	X
IPI00021091	LGI1	ISOFORM 1 OF LEUCINE-RICH GLIOMA-INACTIVATED PROTEIN 1 PRECURSOR.		X	X
IPI00021119	CHST1	CARBOHYDRATE SULFOTRANSFERASE 1.		X	X
IPI00021199	STMN3	STATHMIN-3.		X	X
IPI00021274	EPHA8	EPHRIN TYPE-A RECEPTOR 8 PRECURSOR.		X	X
IPI00021327	GRB2	ISOFORM 1 OF GROWTH FACTOR RECEPTOR-BOUND PROTEIN 2.		X	X
IPI00021733	NDST4	BIFUNCTIONAL HEPARAN SULFATE N-DEACETYLASE/N-SULFOTRANSFERASE 4.		X	X
IPI00021794	CTSA	LYSOSOMAL PROTECTIVE PROTEIN PRECURSOR.		X	X
IPI00021812	AHNAK	NEUROBLAST DIFFERENTIATION-ASSOCIATED PROTEIN AHNAK.		X	X
IPI00022055	PCAF	HISTONE ACETYLTRANSFERASE PCAF.		X	X
IPI00022078	NDRG1	PROTEIN NDRG1.		X	X
IPI00022295	PF4V1	PLATELET FACTOR 4 VARIANT PRECURSOR.		X	X
IPI00022314	SOD2	SUPEROXIDE DISMUTASE [MN], MITOCHONDRIAL PRECURSOR.		X	X
IPI00022462	TFRC	TRANSFERRIN RECEPTOR PROTEIN 1.		X	X
IPI00022542	ROCK1	RHO-ASSOCIATED PROTEIN KINASE 1.		X	X
IPI00022674	OSMR	ISOFORM 1 OF ONCOSTATIN-M SPECIFIC RECEPTOR SUBUNIT BETA PRECURSOR.		X	X
IPI00022774	VCP	TRANSITIONAL ENDOPLASMIC RETICULUM ATPASE.		X	X
IPI00022890	IGLV7-43	IG LAMBDA CHAIN V REGION 4A PRECURSOR.		X	X
IPI00023191	TOM1	TARGET OF MYB1.		X	X
IPI00023598	TUBB4	TUBULIN BETA-4 CHAIN.		X	X
IPI00023754	NELL1	PROTEIN KINASE C-BINDING PROTEIN NELL1 PRECURSOR.		X	X
IPI00024032	TBC1D29	TBC1 DOMAIN FAMILY, MEMBER 29.		X	X
IPI00024067	CLTC	ISOFORM 1 OF CLATHRIN HEAVY CHAIN 1.		X	X
IPI00024107	SNCA	ISOFORM 1 OF ALPHA-SYNUCLEIN.		X	X
IPI00024138		UNCHARACTERIZED PROTEIN ENSP00000374816.		X	X
IPI00024248	SLC5A5	SODIUM/IODIDE COTRANSPORTER.		X	X
IPI00024273	VLDLR	ISOFORM LONG OF VERY LOW-DENSITY LIPOPROTEIN RECEPTOR PRECURSOR.		X	X
IPI00024580	MCCC1	METHYLCROTONOYL-COA CARBOXYLASE SUBUNIT ALPHA, MITOCHONDRIAL PRECURSOR.		X	X
IPI00024766	PLXNC1	PLEXIN-C1 PRECURSOR.		X	X
IPI00024887	BMP6	BONE MORPHOGENETIC PROTEIN 6 PRECURSOR.		X	X

Table S1. Proteins Identified or Not in MS Patients Compared to Normals and Other Neurologic Disease (OND)					
**IPI**	**Gene**	**Protein Name**	**MS**	**Normal**	**OND**
IPI00024919	PRDX3	THIOREDOXIN-DEPENDENT PEROXIDE REDUCTASE, MITOCHONDRIAL PRECURSOR.		X	X
IPI00024929	ASAM	ADIPOCYTE ADHESION MOLECULE PRECURSOR.		X	X
IPI00025311	BCAS1	ISOFORM 1 OF BREAST CARCINOMA-AMPLIFIED SEQUENCE 1.		X	X
IPI00025447	EEF1A1	ELONGATION FACTOR 1-ALPHA.		X	X
IPI00025473	B4GALNT1	BETA-1,4 N-ACETYLGALACTOSAMINYLTRANSFERASE 1.		X	X
IPI00025476	AMY1A; AMY1B; AMY1C; AMY2A	PANCREATIC ALPHA-AMYLASE PRECURSOR.		X	X
IPI00025647	FBXO21	ISOFORM 1 OF F-BOX ONLY PROTEIN 21.		X	X
IPI00025869	GLA	ALPHA-GALACTOSIDASE A PRECURSOR.		X	X
IPI00025992	HAMP	HEPCIDIN PRECURSOR.		X	X
IPI00026125	DNASE1L1	DEOXYRIBONUCLEASE I-LIKE 1 PRECURSOR.		X	X
IPI00026230	HNRPH2	HETEROGENEOUS NUCLEAR RIBONUCLEOPROTEIN H2.		X	X
IPI00026262	RASA1	ISOFORM 1 OF RAS GTPASE-ACTIVATING PROTEIN 1.		X	X
IPI00026285	ST8SIA3	SIA-ALPHA-2,3-GAL-BETA-1,4-GLCNAC-R:ALPHA 2,8-SIALYLTRANSFERASE.		X	X
IPI00026358	GABARAPL2	GAMMA-AMINOBUTYRIC ACID RECEPTOR-ASSOCIATED PROTEIN-LIKE 2.		X	X
IPI00026546	PAFAH1B2	PLATELET-ACTIVATING FACTOR ACETYLHYDROLASE IB SUBUNIT BETA.		X	X
IPI00027223	IDH1	ISOCITRATE DEHYDROGENASE [NADP] CYTOPLASMIC.		X	X
IPI00027239	TNFSF12; TNFSF12-TNFSF13; TNFSF13	ISOFORM ALPHA OF TUMOR NECROSIS FACTOR LIGAND SUPERFAMILY MEMBER 13 PRECURSOR.		X	X
IPI00027264	CALB2	CALRETININ.		X	X
IPI00027429	FABP7	PUTATIVE UNCHARACTERIZED PROTEIN DKFZP547J2313.		X	X
IPI00027444	SERPINB1	LEUKOCYTE ELASTASE INHIBITOR.		X	X
IPI00027457	C1QL1	C1Q-RELATED FACTOR PRECURSOR.		X	X
IPI00027463	S100A6	PROTEIN S100-A6.		X	X
IPI00027464	PPP3R1	CALCINEURIN SUBUNIT B ISOFORM 1.		X	X
IPI00027721	PDGFRA	ISOFORM 1 OF ALPHA-TYPE PLATELET-DERIVED GROWTH FACTOR RECEPTOR PRECURSOR.		X	X
IPI00027744	NR3C2	ISOFORM 1 OF MINERALOCORTICOID RECEPTOR.		X	X
IPI00027806	CRISPLD1	CYSTEINE-RICH SECRETORY PROTEIN LCCL DOMAIN-CONTAINING 1 PRECURSOR.		X	X
IPI00027875	SYT11	SYNAPTOTAGMIN-11.		X	X

Table S1. Proteins Identified or Not in MS Patients Compared to Normals and Other Neurologic Disease (OND)					
**IPI**	**Gene**	**Protein Name**	**MS**	**Normal**	**OND**
IPI00028082	RECK	REVERSION-INDUCING CYSTEINE-RICH PROTEIN WITH KAZAL MOTIFS PRECURSOR.		X	X
IPI00028448	BAI3	BRAIN-SPECIFIC ANGIOGENESIS INHIBITOR 3 PRECURSOR.		X	X
IPI00028561	KIF5C	KINESIN HEAVY CHAIN ISOFORM 5C.		X	X
IPI00028600	KLK7	ISOFORM 1 OF KALLIKREIN-7 PRECURSOR.		X	X
IPI00028786	PKD1	ISOFORM 3 OF POLYCYSTIN-1 PRECURSOR.		X	X
IPI00029275	MFI2	ISOFORM 1 OF MELANOTRANSFERRIN PRECURSOR.		X	X
IPI00029591	SELPLG	P-SELECTIN GLYCOPROTEIN LIGAND 1 PRECURSOR.		X	X
IPI00029699	RNASE4	RIBONUCLEASE 4 PRECURSOR.		X	X
IPI00029700	DSCAM	ISOFORM LONG OF DOWN SYNDROME CELL ADHESION MOLECULE PRECURSOR.		X	X
IPI00029928	ELN	ELASTIN.		X	X
IPI00030205	IGKV3-20	IG KAPPA CHAIN V-III REGION HAH PRECURSOR.		X	X
IPI00030431	ANTXR1	ISOFORM 1 OF ANTHRAX TOXIN RECEPTOR 1 PRECURSOR.		X	X
IPI00030757	ADAMTS2	ISOFORM LPNPI OF ADAMTS-2 PRECURSOR.		X	X
IPI00030847	TM9SF3	TRANSMEMBRANE 9 SUPERFAMILY MEMBER 3 PRECURSOR.		X	X
IPI00030871	VNN1	PANTETHEINASE PRECURSOR.		X	X
IPI00030877	SEP15	15 KDA SELENOPROTEIN ISOFORM 1 PRECURSOR.		X	X
IPI00030882	GRIA2	ISOFORM FLOP OF GLUTAMATE RECEPTOR 2 PRECURSOR.		X	X
IPI00031019	CST8	CYSTATIN-8 PRECURSOR.		X	X
IPI00031131	C20orf3	ADIPOCYTE PLASMA MEMBRANE-ASSOCIATED PROTEIN.		X	X
IPI00031411	FAT	CADHERIN-RELATED TUMOR SUPPRESSOR HOMOLOG PRECURSOR.		X	X
IPI00031506	HCN1	POTASSIUM/SODIUM HYPERPOLARIZATION-ACTIVATED CYCLIC NUCLEOTIDE-GATED CHANNEL 1.		X	X
IPI00031534	ST6GALNAC1	ALPHA-N-ACETYLGALACTOSAMINIDE ALPHA-2,6-SIALYLTRANSFERASE 1.		X	X
IPI00031696	FASTKD3	FAST KINASE DOMAIN-CONTAINING PROTEIN 3.		X	X
IPI00031765	PCDHGC4	ISOFORM 2 OF PROTOCADHERIN GAMMA C4 PRECURSOR.		X	X
IPI00032050	WBP2	WW DOMAIN-BINDING PROTEIN 2.		X	X
IPI00032227	RPH3A	ISOFORM 1 OF RABPHILIN-3A.		X	X
IPI00032311	LBP	LIPOPOLYSACCHARIDE-BINDING PROTEIN PRECURSOR.		X	X
IPI00032338	KLHL20	KELCH-LIKE 20.		X	X
IPI00032830	REXO2	ISOFORM 1 OF OLIGORIBONUCLEASE, MITOCHONDRIAL PRECURSOR (FRAGMENT).		X	X
IPI00032904	SNCB	BETA-SYNUCLEIN.		X	X
IPI00033030	ADRM1	PROTEIN ADRM1.		X	X
IPI00033419	FEM1B	PROTEIN FEM-1 HOMOLOG B.		X	X
IPI00033600	PPP1R7	ISOFORM 1 OF PROTEIN PHOSPHATASE 1 REGULATORY SUBUNIT 7.		X	X

Table S1. Proteins Identified or Not in MS Patients Compared to Normals and Other Neurologic Disease (OND)					
**IPI**	**Gene**	**Protein Name**	**MS**	**Normal**	**OND**
IPI00038378	ENOPH1	ISOFORM 1 OF ENOLASE-PHOSPHATASE E1.		X	X
IPI00044326	CHST14	CARBOHYDRATE SULFOTRANSFERASE 14.		X	X
IPI00044707	HAPLN4; TM6SF2	HYALURONAN AND PROTEOGLYCAN LINK PROTEIN 4 PRECURSOR.		X	X
IPI00045839	LEPRE1	ISOFORM 3 OF PROLYL 3-HYDROXYLASE 1 PRECURSOR.		X	X
IPI00059164	GAL3ST3	GALACTOSE-3-O-SULFOTRANSFERASE 3.		X	X
IPI00059395	KIFC2	KINESIN-LIKE PROTEIN KIFC2.		X	X
IPI00060308	PDLIM7	ISOFORM 6 OF PDZ AND LIM DOMAIN PROTEIN 7.		X	X
IPI00061448		13 KDA PROTEIN.		X	X
IPI00061520	MGC4655	HYPOTHETICAL PROTEIN LOC84752.		X	X
IPI00062037	DYNLL2	DYNEIN LIGHT CHAIN 2, CYTOPLASMIC.		X	X
IPI00063827	ABHD14B	ISOFORM 1 OF ABHYDROLASE DOMAIN-CONTAINING PROTEIN 14B.		X	X
IPI00064652	VEGFA	VASCULAR ENDOTHELIAL GROWTH FACTOR A ISOFORM E PRECURSOR.		X	X
IPI00065312	B4GALNT4	N-ACETYL-BETA-GLUCOSAMINYL-GLYCOPROTEIN 4-BETA-N-ACETYLGALACTOSAMINYLTRANSFERASE 1.		X	X
IPI00075013	C1QTNF1	COMPLEMENT C1Q TUMOR NECROSIS FACTOR-RELATED PROTEIN 1 PRECURSOR.		X	X
IPI00099650	JAG1	PROTEIN JAGGED-1 PRECURSOR.		X	X
IPI00099838	ACP6	ISOFORM 1 OF LYSOPHOSPHATIDIC ACID PHOSPHATASE TYPE 6 PRECURSOR.		X	X
IPI00103853	C18orf22	ISOFORM 1 OF PUTATIVE RIBOSOME-BINDING FACTOR A, MITOCHONDRIAL PRECURSOR.		X	X
IPI00103871	ROBO4	ISOFORM 1 OF ROUNDABOUT HOMOLOG 4 PRECURSOR.		X	X
IPI00106646	SDF4	45 KDA CALCIUM-BINDING PROTEIN PRECURSOR.		X	X
IPI00107886	SEMA6B	SEMAPHORIN 6B ISOFORM 2.		X	X
IPI00140827	LOC728825	SIMILAR TO SMT3 SUPPRESSOR OF MIF TWO 3 HOMOLOG 2.		X	X
IPI00141938	H2AFV	H2A HISTONE FAMILY, MEMBER V ISOFORM 2.		X	X
IPI00143753	SR140	ISOFORM 1 OF U2-ASSOCIATED PROTEIN SR140.		X	X
IPI00151036	RNF13	RING FINGER PROTEIN 13.		X	X
IPI00152182	KLHDC4	ISOFORM 1 OF KELCH DOMAIN-CONTAINING PROTEIN 4.		X	X
IPI00152216	RIC3	ISOFORM 1 OF PROTEIN RIC-3 PRECURSOR.		X	X
IPI00152326	GSTM1	GLUTATHIONE S-TRANSFERASE M1 ISOFORM 2.		X	X
IPI00152769	TRPC4AP	ISOFORM 1 OF TRPC4-ASSOCIATED PROTEIN.		X	X
IPI00157454	HS6ST2	ISOFORM 1 OF HEPARAN-SULFATE 6-O-SULFOTRANSFERASE 2.		X	X
IPI00162735	ATRN	ISOFORM 2 OF ATTRACTIN PRECURSOR.		X	X
IPI00163187	FSCN1	FASCIN.		X	X

Table S1. Proteins Identified or Not in MS Patients Compared to Normals and Other Neurologic Disease (OND)					
**IPI**	**Gene**	**Protein Name**	**MS**	**Normal**	**OND**
IPI00165229	PCSK5	PROPROTEIN CONVERTASE SUBTILISIN/KEXIN TYPE 5 PREPROPROTEIN.		X	X
IPI00165931	PLXNA4	ISOFORM 1 OF PLEXIN-A4 PRECURSOR.		X	X
IPI00165936	CLIC6	ISOFORM A OF CHLORIDE INTRACELLULAR CHANNEL 6.		X	X
IPI00166553	FAM19A2	ISOFORM 1 OF PROTEIN FAM19A2 PRECURSOR.		X	X
IPI00166807	OXR1	ISOFORM 3 OF OXIDATION RESISTANCE PROTEIN 1.		X	X
IPI00166892	DPP10	DPPY SPLICE VARIANT C.		X	X
IPI00167254	PLD5	ISOFORM 4 OF INACTIVE PHOSPHOLIPASE D5.		X	X
IPI00168520	MATN2	ISOFORM 2 OF MATRILIN-2 PRECURSOR.		X	X
IPI00168862	PXT1	CONSERVED HYPOTHETICAL PROTEIN.		X	X
IPI00168885	DHX57	ISOFORM 1 OF PUTATIVE ATP-DEPENDENT RNA HELICASE DHX57.		X	X
IPI00168920	COL24A1	COLLAGEN, TYPE XXIV, ALPHA 1.		X	X
IPI00168921	WBSCR17	PUTATIVE POLYPEPTIDE N-ACETYLGALACTOSAMINYLTRANSFERASE-LIKE PROTEIN 3.		X	X
IPI00169115	OR13C3	OLFACTORY RECEPTOR OR9-8.		X	X
IPI00170635	SECTM1	SECRETED AND TRANSMEMBRANE PROTEIN 1 PRECURSOR.		X	X
IPI00170766	CASC5	ISOFORM 2 OF PROTEIN CASC5.		X	X
IPI00171230	ERC1	ISOFORM 2 OF ELKS/RAB6-INTERACTING/CAST FAMILY MEMBER 1.		X	X
IPI00171611	HIST2H3A; HIST2H3C	HISTONE H3.2.		X	X
IPI00171678	DBH	DOPAMINE BETA-HYDROXYLASE.		X	X
IPI00171874	RASGRP3	RAS GUANYL-RELEASING PROTEIN 3.		X	X
IPI00174794	PTK7	TRANSMEMBRANE RECEPTOR PTK7-5.		X	X
IPI00174976	MPP5	ISOFORM 1 OF MAGUK P55 SUBFAMILY MEMBER 5.		X	X
IPI00175654	C17orf60	PROBABLE MAST CELL ANTIGEN 32 HOMOLOG PRECURSOR.		X	X
IPI00176104	SLITRK2	ISOFORM 1 OF SLIT AND NTRK-LIKE PROTEIN 2 PRECURSOR.		X	X
IPI00176398	SLITRK6	ISOFORM 1 OF SLIT AND NTRK-LIKE PROTEIN 6 PRECURSOR.		X	X
IPI00176920	NPHP4	NEPHROCYSTIN-4.		X	X
IPI00178302	SEMA6D	ISOFORM 4 OF SEMAPHORIN-6D PRECURSOR.		X	X
IPI00178352	FLNC	ISOFORM 1 OF FILAMIN-C.		X	X
IPI00178767	SMPDL3A	ACID SPHINGOMYELINASE-LIKE PHOSPHODIESTERASE 3A PRECURSOR.		X	X
IPI00179473	SQSTM1	ISOFORM 1 OF SEQUESTOSOME-1.		X	X
IPI00179851	C11orf9	NDT80/PHOG LIKE DNA-BINDING FAMILY PROTEIN.		X	X
IPI00180384	DNAH7	DYNEIN, AXONEMAL, HEAVY CHAIN 7.		X	X
IPI00180707	FREM2	ISOFORM 1 OF FRAS1-RELATED EXTRACELLULAR MATRIX PROTEIN 2 PRECURSOR.		X	X

Table S1. Proteins Identified or Not in MS Patients Compared to Normals and Other Neurologic Disease (OND)					
**IPI**	**Gene**	**Protein Name**	**MS**	**Normal**	**OND**
IPI00181174	NLGN4X	ISOFORM 1 OF NEUROLIGIN-4, X-LINKED PRECURSOR.		X	X
IPI00185088	IGSF11	IMMUNOGLOBULIN SUPERFAMILY, MEMBER 11 ISOFORM B.		X	X
IPI00186004	KIAA1641	HYPOTHETICAL PROTEIN LOC57730.		X	X
IPI00187143	RAB4B	ISOFORM 2 OF RAS-RELATED PROTEIN RAB-4B.		X	X
IPI00215894	KNG1	ISOFORM LMW OF KININOGEN-1 PRECURSOR.		X	X
IPI00215899	SRPX	ISOFORM 2 OF SUSHI REPEAT-CONTAINING PROTEIN SRPX PRECURSOR.		X	X
IPI00215979	BPGM	BISPHOSPHOGLYCERATE MUTASE.		X	X
IPI00215980	PVRL2	ISOFORM ALPHA OF POLIOVIRUS RECEPTOR-RELATED PROTEIN 2 PRECURSOR.		X	X
IPI00216049	HNRPK	ISOFORM 1 OF HETEROGENEOUS NUCLEAR RIBONUCLEOPROTEIN K.		X	X
IPI00216106	OLA1	ISOFORM 3 OF OBG-LIKE ATPASE 1.		X	X
IPI00216457	HIST2H2AA3; HIST2H2AA4	HISTONE H2A TYPE 2-A.		X	X
IPI00216592	HNRNPC	ISOFORM C1 OF HETEROGENEOUS NUCLEAR RIBONUCLEOPROTEINS C1/C2.		X	X
IPI00216694	PLS3	PLASTIN 3.		X	X
IPI00216780	CILP2	CARTILAGE INTERMEDIATE LAYER PROTEIN 2 PRECURSOR.		X	X
IPI00217005	ANKRD18A	ANKYRIN REPEAT DOMAIN-CONTAINING PROTEIN 18A.		X	X
IPI00217236	TBCA	TUBULIN-SPECIFIC CHAPERONE A.		X	X
IPI00217264	MAP7D3	ISOFORM 3 OF MAP7 DOMAIN-CONTAINING PROTEIN 3.		X	X
IPI00217376	SCN4B	ISOFORM 1 OF SODIUM CHANNEL SUBUNIT BETA-4 PRECURSOR.		X	X
IPI00217435	SCUBE1	SIGNAL PEPTIDE, CUB AND EGF-LIKE DOMAIN-CONTAINING PROTEIN 1 PRECURSOR.		X	X
IPI00217537	ASXL1	ISOFORM 1 OF PUTATIVE POLYCOMB GROUP PROTEIN ASXL1.		X	X
IPI00217652	GLT8D3	ISOFORM 1 OF GLYCOSYLTRANSFERASE 8 DOMAIN-CONTAINING PROTEIN 3.		X	X
IPI00217781	LOC399947	SIMILAR TO EXPRESSED SEQUENCE AI593442.		X	X
IPI00217966	LDHA	ISOFORM 1 OF L-LACTATE DEHYDROGENASE A CHAIN.		X	X
IPI00218292	UFD1L	ISOFORM SHORT OF UBIQUITIN FUSION DEGRADATION PROTEIN 1 HOMOLOG.		X	X
IPI00218345	LOC654264; TUBA3C; TUBA3D	ISOFORM 2 OF TUBULIN ALPHA-3C/D CHAIN.		X	X
IPI00218465	PLAA	PHOSPHOLIPASE A-2-ACTIVATING PROTEIN.		X	X
IPI00218493	HPRT1	HYPOXANTHINE-GUANINE PHOSPHORIBOSYLTRANSFERASE.		X	X
IPI00218667	STMN2	STATHMIN-2.		X	X
IPI00218874	SPP1	ISOFORM B OF OSTEOPONTIN PRECURSOR.		X	X
IPI00218914	ALDH1A1	RETINAL DEHYDROGENASE 1.		X	X

Table S1. Proteins Identified or Not in MS Patients Compared to Normals and Other Neurologic Disease (OND)					
**IPI**	**Gene**	**Protein Name**	**MS**	**Normal**	**OND**
IPI00218946	HCN2	POTASSIUM/SODIUM HYPERPOLARIZATION-ACTIVATED CYCLIC NUCLEOTIDE-GATED CHANNEL 2.		X	X
IPI00218999	CFH	ISOFORM 2 OF COMPLEMENT FACTOR H PRECURSOR.		X	X
IPI00219005	FKBP4	FK506-BINDING PROTEIN 4.		X	X
IPI00219042	PAM	ISOFORM 3 OF PEPTIDYL-GLYCINE ALPHA-AMIDATING MONOOXYGENASE PRECURSOR.		X	X
IPI00219067	GSTM2	GLUTATHIONE S-TRANSFERASE MU 2.		X	X
IPI00219525	PGD	6-PHOSPHOGLUCONATE DEHYDROGENASE, DECARBOXYLATING.		X	X
IPI00219526	PGM1	ISOFORM 1 OF PHOSPHOGLUCOMUTASE-1.		X	X
IPI00219575	BLMH	BLEOMYCIN HYDROLASE.		X	X
IPI00219684	FABP3	FATTY ACID-BINDING PROTEIN, HEART.		X	X
IPI00219910		22 KDA PROTEIN.		X	X
IPI00220281	GNAO1	GUANINE NUCLEOTIDE-BINDING PROTEIN G(O) SUBUNIT ALPHA 1.		X	X
IPI00220292	ART3	ISOFORM 1 OF ECTO-ADP-RIBOSYLTRANSFERASE 3 PRECURSOR.		X	X
IPI00220334	SEZ6L	ISOFORM 3 OF SEIZURE 6-LIKE PROTEIN PRECURSOR.		X	X
IPI00220361	CALB1	CALBINDIN.		X	X
IPI00220741	SPTA1	SPECTRIN, ALPHA, ERYTHROCYTIC 1.		X	X
IPI00220748	ITGA7	ISOFORM ALPHA-7X1A OF INTEGRIN ALPHA-7 PRECURSOR.		X	X
IPI00220828	TMSB4X	THYMOSIN BETA-4.		X	X
IPI00221006	TCF7L2	ISOFORM 4 OF TRANSCRIPTION FACTOR 7-LIKE 2.		X	X
IPI00221034	RELB	TRANSCRIPTION FACTOR RELB.		X	X
IPI00221080	PTHLH	ISOFORM 2 OF PARATHYROID HORMONE-RELATED PROTEIN PRECURSOR.		X	X
IPI00221117	ACYP1; C17orf13	ACYLPHOSPHATASE-1.		X	X
IPI00243221	NRD1	NARDILYSIN (N-ARGININE DIBASIC CONVERTASE) ISOFORM A.		X	X
IPI00243451	LST-3TM12; SLCO1B1; SLCO1B3	LIVER-SPECIFIC ORGANIC ANION TRANSPORTER 3TM12.		X	X
IPI00243995	NEK5	SERINE/THREONINE-PROTEIN KINASE NEK5.		X	X
IPI00248596	ELFN1	SIMILAR TO SLIT HOMOLOG 1.		X	X
IPI00252731	DPP6	ISOFORM DPPX-S OF DIPEPTIDYL AMINOPEPTIDASE-LIKE PROTEIN 6.		X	X
IPI00253036	CD99	ISOFORM I OF CD99 ANTIGEN PRECURSOR.		X	X
IPI00289329	EPHB3	EPHRIN TYPE-B RECEPTOR 3 PRECURSOR.		X	X
IPI00289334	FLNB	ISOFORM 1 OF FILAMIN-B.		X	X

Table S1. Proteins Identified or Not in MS Patients Compared to Normals and Other Neurologic Disease (OND)					
**IPI**	**Gene**	**Protein Name**	**MS**	**Normal**	**OND**
IPI00289746	PAK1	ISOFORM 2 OF SERINE/THREONINE-PROTEIN KINASE PAK 1.		X	X
IPI00289861	ZCCHC11	ISOFORM 1 OF ZINC FINGER CCHC DOMAIN-CONTAINING PROTEIN 11.		X	X
IPI00289870	PCDH7	ISOFORM C OF PROTOCADHERIN-7 PRECURSOR.		X	X
IPI00290857	KRT3	KERATIN, TYPE II CYTOSKELETAL 3.		X	X
IPI00291395	FLRT1	FIBRONECTIN LEUCINE RICH TRANSMEMBRANE PROTEIN 1.		X	X
IPI00291488	WFDC2	ISOFORM 1 OF WAP FOUR-DISULFIDE CORE DOMAIN PROTEIN 2 PRECURSOR.		X	X
IPI00292043	SDK2	ISOFORM 1 OF PROTEIN SIDEKICK-2 PRECURSOR.		X	X
IPI00292496	TUBB8	BETA-TUBULIN 4Q.		X	X
IPI00292657	LTB4DH	NADP-DEPENDENT LEUKOTRIENE B4 12-HYDROXYDEHYDROGENASE.		X	X
IPI00293128	EXT1	EXOSTOSIN-1.		X	X
IPI00293530	C3AR1	C3A ANAPHYLATOXIN CHEMOTACTIC RECEPTOR.		X	X
IPI00293539	CDH11	ISOFORM 2 OF CADHERIN-11 PRECURSOR.		X	X
IPI00294193	ITIH4	ISOFORM 1 OF INTER-ALPHA-TRYPSIN INHIBITOR HEAVY CHAIN H4 PRECURSOR.		X	X
IPI00294705	PAPLN	PAPILIN.		X	X
IPI00294834	ASPH	ASPARTYL/ASPARAGINYL BETA-HYDROXYLASE.		X	X
IPI00295172	NINJ1	NINJURIN-1.		X	X
IPI00296099	THBS1	THROMBOSPONDIN-1 PRECURSOR.		X	X
IPI00296197	SIL1	NUCLEOTIDE EXCHANGE FACTOR SIL1 PRECURSOR.		X	X
IPI00296259	TMED4	TRANSMEMBRANE EMP24 DOMAIN-CONTAINING PROTEIN 4 PRECURSOR.		X	X
IPI00296337	PRKDC	ISOFORM 1 OF DNA-DEPENDENT PROTEIN KINASE CATALYTIC SUBUNIT.		X	X
IPI00296461	SMPD1	ISOFORM 1 OF SPHINGOMYELIN PHOSPHODIESTERASE PRECURSOR.		X	X
IPI00296558	CPXM2	CARBOXYPEPTIDASE-LIKE PROTEIN X2 PRECURSOR.		X	X
IPI00296713	GRN	ISOFORM 1 OF GRANULINS PRECURSOR.		X	X
IPI00297277	RNF150	ISOFORM 1 OF RING FINGER PROTEIN 150 PRECURSOR.		X	X
IPI00297288	CDGAP	CDC42 GTPASE-ACTIVATING PROTEIN.		X	X
IPI00297550	F13A1	COAGULATION FACTOR XIII A CHAIN PRECURSOR.		X	X
IPI00297779	CCT2	T-COMPLEX PROTEIN 1 SUBUNIT BETA.		X	X
IPI00298285	ERBB3	ISOFORM 1 OF RECEPTOR TYROSINE-PROTEIN KINASE ERBB-3 PRECURSOR.		X	X
IPI00298650	ADAMTS8	ADAMTS-8 PRECURSOR.		X	X
IPI00298994	TLN1	TALIN-1.		X	X
IPI00299399	S100B	PROTEIN S100-B.		X	X
IPI00299435	APOF	APOLIPOPROTEIN F PRECURSOR.		X	X
IPI00299571	PDIA6	ISOFORM 2 OF PROTEIN DISULFIDE-ISOMERASE A6 PRECURSOR.		X	X
IPI00299627	DUOX2	DUAL OXIDASE 2 PRECURSOR.		X	X

Table S1. Proteins Identified or Not in MS Patients Compared to Normals and Other Neurologic Disease (OND)					
**IPI**	**Gene**	**Protein Name**	**MS**	**Normal**	**OND**
IPI00299758	CHST12	CARBOHYDRATE SULFOTRANSFERASE 12.		X	X
IPI00299778	PON3	SERUM PARAOXONASE/LACTONASE 3.		X	X
IPI00300207	LOC91431	ISOFORM 1 OF UNCHARACTERIZED PROTEIN FLJ44066.		X	X
IPI00301098	C1orf187	UNCHARACTERIZED PROTEIN C1ORF187 PRECURSOR.		X	X
IPI00301288	SVEP1	POLYDOM.		X	X
IPI00301465	HJURP	14-3-3-ASSOCIATED AKT SUBSTRATE.		X	X
IPI00301631	TOR3A	ISOFORM 1 OF TORSIN-3A PRECURSOR.		X	X
IPI00302133	TRPV5	TRANSIENT RECEPTOR POTENTIAL CATION CHANNEL SUBFAMILY V MEMBER 5.		X	X
IPI00302944	COL12A1	ISOFORM 4 OF COLLAGEN ALPHA-1(XII) CHAIN PRECURSOR.		X	X
IPI00302962	AMPH	AMPHIPHYSIN I VARIANT CT4 (FRAGMENT).		X	X
IPI00303210	ENPP2	ISOFORM 2 OF ECTONUCLEOTIDE PYROPHOSPHATASE/PHOSPHODIESTERASE FAMILY MEMBER 2 PRECURSOR.		X	X
IPI00303476	ATP5B	ATP SYNTHASE SUBUNIT BETA, MITOCHONDRIAL PRECURSOR.		X	X
IPI00303882	M6PRBP1	ISOFORM B OF MANNOSE-6-PHOSPHATE RECEPTOR-BINDING PROTEIN 1.		X	X
IPI00304331	B3GAT3	GALACTOSYLGALACTOSYLXYLOSYLPROTEIN 3-BETA-GLUCURONOSYLTRANSFERASE 3.		X	X
IPI00304577	AP2A1	ISOFORM A OF AP-2 COMPLEX SUBUNIT ALPHA-1.		X	X
IPI00304596	NONO	NON-POU DOMAIN-CONTAINING OCTAMER-BINDING PROTEIN.		X	X
IPI00304925	HSPA1A; HSPA1B	HEAT SHOCK 70 KDA PROTEIN 1.		X	X
IPI00306046	EDIL3	ISOFORM 1 OF EGF-LIKE REPEAT AND DISCOIDIN I-LIKE DOMAIN-CONTAINING PROTEIN 3 PRECURSOR.		X	X
IPI00306850	MEGF6	EGF-LIKE-DOMAIN, MULTIPLE 3.		X	X
IPI00306884	ST3GAL4	CDNA FLJ11867 FIS, CLONE HEMBA1006976, WEAKLY SIMILAR TO H.SAPIENS GAL-BETA(1-3/1-4)GLCNAC ALPHA-2.3-SIALYLTRANSFERASE.		X	X
IPI00306959	KRT7	KERATIN, TYPE II CYTOSKELETAL 7.		X	X
IPI00307612	CDH20	CADHERIN-20 PRECURSOR.		X	X
IPI00307729	ADAMTS3	ADAMTS-3 PRECURSOR.		X	X
IPI00328587	ENO1P	ENOLASE.		X	X
IPI00328680	MCFD2	MULTIPLE COAGULATION FACTOR DEFICIENCY PROTEIN 2 PRECURSOR.		X	X
IPI00328709	GREM2	GREMLIN-2 PRECURSOR.		X	X
IPI00328829	ITIH5	INTER-ALPHA TRYPSIN INHIBITOR HEAVY CHAIN PRECURSOR 5 ISOFORM 1.		X	X
IPI00329538	PRSS8	PROSTASIN PRECURSOR.		X	X
IPI00329593	ADPGK	ISOFORM 2 OF ADP-DEPENDENT GLUCOKINASE.		X	X

Table S1. Proteins Identified or Not in MS Patients Compared to Normals and Other Neurologic Disease (OND)					
**IPI**	**Gene**	**Protein Name**	**MS**	**Normal**	**OND**
IPI00329605	MSH3	DNA MISMATCH REPAIR PROTEIN MSH3.		X	X
IPI00329801	ANXA5	ANNEXIN A5.		X	X
IPI00333126	LRRC56	LEUCINE-RICH REPEAT-CONTAINING PROTEIN 56.		X	X
IPI00333197	GCC2	ISOFORM 2 OF GRIP AND COILED-COIL DOMAIN-CONTAINING PROTEIN 2.		X	X
IPI00334254	EGFLAM	ISOFORM 4 OF EGF-LIKE, FIBRONECTIN TYPE-III AND LAMININ G-LIKE DOMAIN-CONTAINING PROTEIN PRECURSOR.		X	X
IPI00334667	PTPRN2	ISOFORM 2 OF RECEPTOR-TYPE TYROSINE-PROTEIN PHOSPHATASE N2 PRECURSOR.		X	X
IPI00335946	FAM120B	FAMILY WITH SEQUENCE SIMILARITY 120B.		X	X
IPI00337612	DCBLD1	DISCOIDIN, CUB AND LCCL DOMAIN-CONTAINING PROTEIN 1 PRECURSOR.		X	X
IPI00374732	PPIAP19	SIMILAR TO PEPTIDYLPROLYL ISOMERASE A ISOFORM 1.		X	X
IPI00375205	GALNT10	ISOFORM 1 OF POLYPEPTIDE N-ACETYLGALACTOSAMINYLTRANSFERASE 10.		X	X
IPI00375746	GBP6	ISOFORM 1 OF GUANYLATE-BINDING PROTEIN 6.		X	X
IPI00375879	KIAA1467	UNCHARACTERIZED PROTEIN KIAA1467.		X	X
IPI00375881	PKD1L3	POLYCYSTIC KIDNEY DISEASE 1-LIKE PROTEIN 3.		X	X
IPI00377045	LAMA3	ALPHA3A.		X	X
IPI00377077	ASTN2	ISOFORM 3 OF ASTROTACTIN-2 PRECURSOR.		X	X
IPI00382421		IG LAMBDA CHAIN V-I REGION NEW.		X	X
IPI00382478		IG HEAVY CHAIN V-III REGION TIL.		X	X
IPI00382481		IG HEAVY CHAIN V-III REGION BUT.		X	X
IPI00382482		IG HEAVY CHAIN V-III REGION CAM.		X	X
IPI00382493		IG HEAVY CHAIN V-III REGION WAS.		X	X
IPI00382500		IG HEAVY CHAIN V-III REGION GAL.		X	X
IPI00382606	F7	FACTOR VII ACTIVE SITE MUTANT IMMUNOCONJUGATE.		X	X
IPI00382938	IGLV4-3	IGLV4-3 PROTEIN.		X	X
IPI00383016		IMMUNGLOBULIN LIGHT CHAIN VARIABLE REGION (FRAGMENT).		X	X
IPI00383581	GANAB	ISOFORM 1 OF NEUTRAL ALPHA-GLUCOSIDASE AB PRECURSOR.		X	X
IPI00383594	MUM1	MELANOMA UBIQUITOUS MUTATED PROTEIN.		X	X
IPI00383680	RPN2	RIBOPHORIN II.		X	X
IPI00383808		IG KAPPA CHAIN V-IV REGION STH (FRAGMENT).		X	X
IPI00383887		IMMUNOBLOBULIN HEAVY CHAIN (FRAGMENT).		X	X
IPI00384400		MYOSIN-REACTIVE IMMUNOGLOBULIN HEAVY CHAIN VARIABLE REGION (FRAGMENT).		X	X

Table S1. Proteins Identified or Not in MS Patients Compared to Normals and Other Neurologic Disease (OND)					
**IPI**	**Gene**	**Protein Name**	**MS**	**Normal**	**OND**
IPI00384407		MYOSIN-REACTIVE IMMUNOGLOBULIN HEAVY CHAIN VARIABLE REGION (FRAGMENT).		X	X
IPI00384542	NID1	ISOFORM 2 OF NIDOGEN-1 PRECURSOR.		X	X
IPI00384952		PUTATIVE UNCHARACTERIZED PROTEIN DKFZP686K04218 (FRAGMENT).		X	X
IPI00385007	AP1B1	PUTATIVE UNCHARACTERIZED PROTEIN DKFZP686A01208.		X	X
IPI00385264		IG MU HEAVY CHAIN DISEASE PROTEIN.		X	X
IPI00386575		IG LAMBDA CHAIN V-I REGION EPS.		X	X
IPI00387095		IG KAPPA CHAIN V-I REGION KA.		X	X
IPI00387096		IG KAPPA CHAIN V-I REGION KUE.		X	X
IPI00387101		IG KAPPA CHAIN V-I REGION SCW.		X	X
IPI00387118		IG KAPPA CHAIN V-III REGION WOL.		X	X
IPI00387119		IG KAPPA CHAIN V-III REGION POM.		X	X
IPI00394712		GRANULOCYTE INHIBITORY PROTEIN.		X	X
IPI00394820	OLFML1	OLFACTOMEDIN-LIKE PROTEIN 1 PRECURSOR.		X	X
IPI00394992	PGLYRP2	ISOFORM 2 OF N-ACETYLMURAMOYL-L-ALANINE AMIDASE PRECURSOR.		X	X
IPI00395866	SCUBE1	SCUBE1 PROTEIN.		X	X
IPI00396077	TOPORS	ISOFORM 1 OF E3 UBIQUITIN-PROTEIN LIGASE TOPORS.		X	X
IPI00396378	HNRNPA2B1	ISOFORM B1 OF HETEROGENEOUS NUCLEAR RIBONUCLEOPROTEINS A2/B1.		X	X
IPI00396383	VWA1	ISOFORM 1 OF VON WILLEBRAND FACTOR A DOMAIN-CONTAINING PROTEIN 1 PRECURSOR.		X	X
IPI00398715	NRP1	NEUROPILIN 1.		X	X
IPI00398918	DKFZp686O24166	PUTATIVE UNCHARACTERIZED PROTEIN DKFZP686I21167.		X	X
IPI00399089	MESDC2	MESODERM DEVELOPMENT CANDIDATE 2.		X	X
IPI00400935	COL16A1	ISOFORM 1 OF COLLAGEN ALPHA-1(XVI) CHAIN PRECURSOR.		X	X
IPI00400967	KIAA1843	KIAA1843 PROTEIN (FRAGMENT).		X	X
IPI00402293	ARSG	ARYLSULFATASE G PRECURSOR.		X	X
IPI00409640	LSR	ISOFORM 1 OF LIPOLYSIS-STIMULATED LIPOPROTEIN RECEPTOR.		X	X
IPI00410013	ZC3H3	ISOFORM 1 OF ZINC FINGER CCCH DOMAIN-CONTAINING PROTEIN 3.		X	X
IPI00410210	LPHN1	ISOFORM 2 OF LATROPHILIN-1 PRECURSOR.		X	X
IPI00410488	CD276	ISOFORM 1 OF CD276 ANTIGEN PRECURSOR.		X	X
IPI00410588	ADAMTSL3	ADAMTS-LIKE PROTEIN 3 PRECURSOR.		X	X
IPI00410675	STX1B	SYNTAXIN-1B.		X	X
IPI00412492	PLXND1	ISOFORM 1 OF PLEXIN-D1 PRECURSOR.		X	X

Table S1. Proteins Identified or Not in MS Patients Compared to Normals and Other Neurologic Disease (OND)					
**IPI**	**Gene**	**Protein Name**	**MS**	**Normal**	**OND**
IPI00413587	BID	ISOFORM 1 OF BH3-INTERACTING DOMAIN DEATH AGONIST.		X	X
IPI00413781	CXCL12	CHEMOKINE (C-X-C MOTIF) LIGAND 12 (STROMAL CELL-DERIVED FACTOR 1) ISOFORM GAMMA.		X	X
IPI00413826	LOC644914	SIMILAR TO H3 HISTONE, FAMILY 3B.		X	X
IPI00413912	TMEM132E	TRANSMEMBRANE PROTEIN 132E PRECURSOR.		X	X
IPI00413959	CLSTN1	CALSYNTENIN-1 PRECURSOR.		X	X
IPI00414676	HSP90AB1	HEAT SHOCK PROTEIN HSP 90-BETA.		X	X
IPI00418169	ANXA2	ANNEXIN A2 ISOFORM 1.		X	X
IPI00418446	ASAH1	N-ACYLSPHINGOSINE AMIDOHYDROLASE (ACID CERAMIDASE) 1 ISOFORM B.		X	X
IPI00419442	IGLV6-57	IGLV6-57 PROTEIN.		X	X
IPI00419565	STAB1	ISOFORM 1 OF STABILIN-1 PRECURSOR.		X	X
IPI00419820	OLFM1	NOELIN1_V3.		X	X
IPI00419836	DCBLD2	ISOFORM 1 OF DISCOIDIN, CUB AND LCCL DOMAIN-CONTAINING PROTEIN 2 PRECURSOR.		X	X
IPI00419908	GPR179	UNCHARACTERIZED PROTEIN GPR179.		X	X
IPI00420071	MAP6	MICROTUBULE-ASSOCIATED PROTEIN 6 ISOFORM 1.		X	X
IPI00423461	IGHA2	PUTATIVE UNCHARACTERIZED PROTEIN DKFZP686C02220 (FRAGMENT).		X	X
IPI00423466	IGHG1	PUTATIVE UNCHARACTERIZED PROTEIN DKFZP686H20196.		X	X
IPI00430291	CAMK2D	ISOFORM DELTA 2 OF CALCIUM/CALMODULIN-DEPENDENT PROTEIN KINASE TYPE II DELTA CHAIN.		X	X
IPI00430808	IGKC	IMMUNOBLOBULIN LIGHT CHAIN (FRAGMENT).		X	X
IPI00430842	IGHA1; IGHV3OR16-13	IGHA1 PROTEIN.		X	X
IPI00432592		126 KDA PROTEIN.		X	X
IPI00432707	CASP12	CASPASE-12.		X	X
IPI00432766	NTNG2	ISOFORM 2 OF NETRIN-G2 PRECURSOR.		X	X
IPI00437751	ACE	ISOFORM SOMATIC-1 OF ANGIOTENSIN-CONVERTING ENZYME, SOMATIC ISOFORM PRECURSOR.		X	X
IPI00438286	ERBB2IP	ISOFORM 1 OF PROTEIN LAP2.		X	X
IPI00440153	XRRA1	68 KDA PROTEIN.		X	X
IPI00440493	ATP5A1	ATP SYNTHASE SUBUNIT ALPHA, MITOCHONDRIAL PRECURSOR.		X	X
IPI00440580	MANEAL	ISOFORM 1 OF GLYCOPROTEIN ENDO-ALPHA-1,2-MANNOSIDASE-LIKE PROTEIN.		X	X
IPI00440932	ADAM9	ISOFORM 1 OF ADAM 9 PRECURSOR.		X	X
IPI00442294	HNT	NEUROTRIMIN VARIANT 3.		X	X

Table S1. Proteins Identified or Not in MS Patients Compared to Normals and Other Neurologic Disease (OND)					
**IPI**	**Gene**	**Protein Name**	**MS**	**Normal**	**OND**
IPI00442297	HNT	ISOFORM 2 OF NEUROTRIMIN PRECURSOR.		X	X
IPI00444272	LIFR	LEUKEMIA INHIBITORY FACTOR RECEPTOR PRECURSOR.		X	X
IPI00444605		CDNA FLJ45296 FIS, CLONE BRHIP3003340, MODERATELY SIMILAR TO ACTIN, ALPHA SKELETAL MUSCLE 2.		X	X
IPI00453473	HIST1H4A; HIST1H4B; HIST1H4C; HIST1H4D; HIST1H4E; HIST1H4F; HIST1H4H; HIST1H4I; HIST1H4J; HIST1H4K; HIST1H4L; HIST2H4A; HIST2H4B; HIST4H4	HISTONE H4.		X	X
IPI00454910	CDC42BPG	SERINE/THREONINE-PROTEIN KINASE MRCK GAMMA.		X	X
IPI00455521	C22:CTA-250D10.9	SIMILAR TO TRANSMEMBRANE PROTEIN 46.		X	X
IPI00455967		UNCHARACTERIZED PROTEIN ENSP00000353619 (FRAGMENT).		X	X
IPI00456589	GALNT11	ISOFORM 1 OF POLYPEPTIDE N-ACETYLGALACTOSAMINYLTRANSFERASE 11.		X	X
IPI00456683	SUPT6H	ISOFORM 3 OF TRANSCRIPTION ELONGATION FACTOR SPT6.		X	X
IPI00465016	QSOX1	ISOFORM 2 OF SULFHYDRYL OXIDASE 1 PRECURSOR.		X	X
IPI00465261	ERAP2	ISOFORM 1 OF ENDOPLASMIC RETICULUM AMINOPEPTIDASE 2.		X	X
IPI00465363	HIST1H2BA	HISTONE H2B TYPE 1-A.		X	X
IPI00465377	MXRA7	ISOFORM 1 OF MATRIX-REMODELING-ASSOCIATED PROTEIN 7.		X	X
IPI00470838	DENND2C	ISOFORM 1 OF DENN DOMAIN-CONTAINING PROTEIN 2C.		X	X
IPI00470937	PTPRK	PROTEIN TYROSINE PHOSPHATASE, RECEPTOR TYPE, K.		X	X
IPI00472249	PTPRN2	PROTEIN TYROSINE PHOSPHATASE, RECEPTOR TYPE, N POLYPEPTIDE 2 ISOFORM 2 PRECURSOR.		X	X
IPI00473033	ZNF69	ISOFORM 1 OF ZINC FINGER PROTEIN 69.		X	X
IPI00477616	PPP2R4	PROTEIN PHOSPHATASE 2A ACTIVATOR, REGULATORY SUBUNIT 4.		X	X

Table S1. Proteins Identified or Not in MS Patients Compared to Normals and Other Neurologic Disease (OND)					
**IPI**	**Gene**	**Protein Name**	**MS**	**Normal**	**OND**
IPI00478414	CHRDL1	VENTROPTIN (FRAGMENT).		X	X
IPI00478809	F5	COAGULATION FACTOR V PRECURSOR.		X	X
IPI00478816	SPINK5	SERINE PROTEASE INHIBITOR KAZAL-TYPE 5 PRECURSOR.		X	X
IPI00478860	MANEA	GLYCOPROTEIN ENDO-ALPHA-1,2-MANNOSIDASE.		X	X
IPI00479083	C10orf137	ISOFORM 2 OF ERYTHROID DIFFERENTIATION-RELATED FACTOR 1.		X	X
IPI00479116	CPN2	CARBOXYPEPTIDASE N SUBUNIT 2 PRECURSOR.		X	X
IPI00479217	HNRNPU	ISOFORM SHORT OF HETEROGENEOUS NUCLEAR RIBONUCLEOPROTEIN U.		X	X
IPI00479514	CACNA2D1	VOLTAGE-DEPENDENT CALCIUM CHANNEL SUBUNIT ALPHA-2/DELTA-1 PRECURSOR.		X	X
IPI00480183	PTPRF	PROTEIN.		X	X
IPI00514285	PTGDS	PROSTAGLANDIN D2 SYNTHASE 21KDA.		X	X
IPI00514517	IGLV5-37	V4-1 PROTEIN.		X	X
IPI00514676	MOG	MYELIN OLIGODENDROCYTE GLYCOPROTEIN ISOFORM BETA2 PRECURSOR.		X	X
IPI00514893	DAAM2	DISHEVELED-ASSOCIATED ACTIVATOR OF MORPHOGENESIS 2.		X	X
IPI00515041	CFH	UNCHARACTERIZED PROTEIN CFH.		X	X
IPI00550162	IGLV3-25	IGLV3-25 PROTEIN.		X	X
IPI00550232	XIRP2	CARDIOMYOPATHY ASSOCIATED 3 ISOFORM 1.		X	X
IPI00550263	CDC42BPA	ISOFORM 5 OF SERINE/THREONINE-PROTEIN KINASE MRCK ALPHA.		X	X
IPI00550363	TAGLN2	TRANSGELIN-2.		X	X
IPI00550533	C1orf56	ISOFORM 1 OF UNCHARACTERIZED PROTEIN C1ORF56 PRECURSOR.		X	X
IPI00550558	POMGNT1	PROTEIN O-LINKED-MANNOSE BETA-1,2-N-ACETYLGLUCOSAMINYLTRANSFERASE 1.		X	X
IPI00550677	WSCD1	WSC DOMAIN-CONTAINING PROTEIN 1.		X	X
IPI00550792	BIN2	ISOFORM 1 OF BRIDGING INTEGRATOR 2.		X	X
IPI00550949	BMP7	BONE MORPHOGENETIC PROTEIN 7 PRECURSOR.		X	X
IPI00552267	IGLV3-19	SIMILAR TO V2-13 PROTEIN.		X	X
IPI00552578	SAA1; SAA2	SERUM AMYLOID A PROTEIN PRECURSOR.		X	X
IPI00552735	IGLV3-12	V2-8 PROTEIN.		X	X
IPI00552771	IGLV3-16	V2-11 PROTEIN.		X	X
IPI00552852	IGLV3-27	V2-19 PROTEIN.		X	X
IPI00552937	NHLRC3	NHL REPEAT CONTAINING 3 ISOFORM A.		X	X
IPI00552939	C1QL3	ISOFORM 1 OF COMPLEMENT C1Q-LIKE PROTEIN 3 PRECURSOR.		X	X
IPI00552943	IGLV1-36	V1-11 PROTEIN.		X	X
IPI00553092	IGLV7-46	V3-3 PROTEIN.		X	X
IPI00554521	FTH1	FERRITIN HEAVY CHAIN.		X	X
IPI00555614	HSP90AB3P	HEAT SHOCK PROTEIN 90BC.		X	X

Table S1. Proteins Identified or Not in MS Patients Compared to Normals and Other Neurologic Disease (OND)					
**IPI**	**Gene**	**Protein Name**	**MS**	**Normal**	**OND**
IPI00556391		ACTIN-LIKE PROTEIN (FRAGMENT).		X	X
IPI00556643	SEMA3F	SEMAPHORIN 3F VARIANT.		X	X
IPI00604599	TMED3	TRANSMEMBRANE EMP24 DOMAIN-CONTAINING PROTEIN 3 PRECURSOR.		X	X
IPI00607655	EPHA7	ISOFORM 2 OF EPHRIN TYPE-A RECEPTOR 7 PRECURSOR.		X	X
IPI00639937	CFB	B-FACTOR, PROPERDIN.		X	X
IPI00640292	C6orf27	ISOFORM 1 OF PROTEIN G7C PRECURSOR.		X	X
IPI00640818	PNPLA6	ISOFORM 3 OF NEUROPATHY TARGET ESTERASE.		X	X
IPI00642861	CXorf36	CDNA FLJ37558 FIS, CLONE BRCOC1000087.		X	X
IPI00643034	PLTP	ISOFORM 1 OF PHOSPHOLIPID TRANSFER PROTEIN PRECURSOR.		X	X
IPI00643115	STMN1	STATHMIN 1/ONCOPROTEIN 18.		X	X
IPI00643348	COMP	80 KDA PROTEIN.		X	X
IPI00643667	C1QTNF3	C1Q AND TUMOR NECROSIS FACTOR RELATED PROTEIN 3 ISOFORM B.		X	X
IPI00643920	TKT	TRANSKETOLASE.		X	X
IPI00644025	SV2A	ISOFORM 1 OF SYNAPTIC VESICLE GLYCOPROTEIN 2A.		X	X
IPI00644191	ZNF90	70 KDA PROTEIN.		X	X
IPI00644472	HDHD2	ISOFORM 2 OF HALOACID DEHALOGENASE-LIKE HYDROLASE DOMAIN-CONTAINING PROTEIN 2.		X	X
IPI00645078	UBA1	UBIQUITIN-LIKE MODIFIER-ACTIVATING ENZYME 1.		X	X
IPI00645194	ITGB1	INTEGRIN BETA 1 ISOFORM 1A PRECURSOR.		X	X
IPI00646281	L1CAM	L1 CELL ADHESION MOLECULE.		X	X
IPI00647217	SKIV2L2	SUPERKILLER VIRALICIDIC ACTIVITY 2-LIKE 2.		X	X
IPI00647357	OSCAR	ISOFORM 1 OF OSTEOCLAST ASSOCIATED IMMUNOGLOBULIN-LIKE RECEPTOR PRECURSOR.		X	X
IPI00651762	TTLL9	TTLL9 PROTEIN.		X	X
IPI00655702	NFASC	ISOFORM 5 OF NEUROFASCIN PRECURSOR.		X	X
IPI00657742	HLA-F	MAJOR HISTOCOMPATIBILITY COMPLEX, CLASS I, F.		X	X
IPI00657936	COL28A1	COLLAGEN, TYPE XXVIII PRECURSOR.		X	X
IPI00719621	PLXNA2	ISOFORM 1 OF PLEXIN-A2 PRECURSOR.		X	X
IPI00736885	LOC440786	IG KAPPA CHAIN V-II REGION TEW.		X	X
IPI00737429	ODZ4	TENEURIN-4.		X	X
IPI00739237	LOC653879	SIMILAR TO COMPLEMENT C3 PRECURSOR.		X	X
IPI00740545	LOC653269	SIMILAR TO PROSTATE, OVARY, TESTIS EXPRESSED PROTEIN ON CHROMOSOME 2 ISOFORM 2.		X	X
IPI00741005	MGA	SIMILAR TO MAX-INTERACTING PROTEIN ISOFORM 4.		X	X

Table S1. Proteins Identified or Not in MS Patients Compared to Normals and Other Neurologic Disease (OND)					
IPI	Gene	Protein Name	MS	Normal	OND
IPI00741608	EIF5AL3	SIMILAR TO EUKARYOTIC TRANSLATION INITIATION FACTOR 5A.		X	X
IPI00743302	ICAM5	INTERCELLULAR ADHESION MOLECULE 5 PRECURSOR.		X	X
IPI00743898		UNCHARACTERIZED PROTEIN ENSP00000357890 (FRAGMENT).		X	X
IPI00744561	IGHA1; IGHV3OR16-13	IGHA1 PROTEIN.		X	X
IPI00744706	SPTAN1	282 KDA PROTEIN.		X	X
IPI00744835	PSAP	ISOFORM SAP-MU-9 OF PROACTIVATOR POLYPEPTIDE PRECURSOR.		X	X
IPI00745103	LOC728307	SIMILAR TO MELANOMA ASSOCIATED ANTIGEN (MUTATED) 1-LIKE 1.		X	X
IPI00745251	MAN1C1	MANNOSYL-OLIGOSACCHARIDE 1,2-ALPHA-MANNOSIDASE IC.		X	X
IPI00745313	AEBP1	ADIPOCYTE ENHANCER BINDING PROTEIN 1 PRECURSOR.		X	X
IPI00745363	LOC652113	IMMUNGLOBULIN HEAVY CHAIN VARIABLE REGION (FRAGMENT).		X	X
IPI00746177	LOC730222	SIMILAR TO TUBULIN ALPHA-2 CHAIN.		X	X
IPI00746388	EZR	EZRIN.		X	X
IPI00748890	LUZP2	ISOFORM 1 OF LEUCINE ZIPPER PROTEIN 2 PRECURSOR.		X	X
IPI00749245	SFRP1	SECRETED FRIZZLED-RELATED PROTEIN 1 PRECURSOR.		X	X
IPI00749440		UNCHARACTERIZED PROTEIN ENSP00000368180.		X	X
IPI00783156	BMPR2	BONE MORPHOGENETIC PROTEIN RECEPTOR TYPE-2 PRECURSOR.		X	X
IPI00783184		IMMUNGLOBULIN HEAVY CHAIN VARIABLE REGION (FRAGMENT).		X	X
IPI00783313	PYGL	GLYCOGEN PHOSPHORYLASE, LIVER FORM.		X	X
IPI00783464	DNAH2	DYNEIN HEAVY CHAIN DOMAIN 3.		X	X
IPI00783471		IMMUNGLOBULIN HEAVY CHAIN VARIABLE REGION (FRAGMENT).		X	X
IPI00783492	LTBP4	ISOFORM 2 OF LATENT-TRANSFORMING GROWTH FACTOR BETA-BINDING PROTEIN 4 PRECURSOR.		X	X
IPI00783604	EPHA6	EPH RECEPTOR A6 ISOFORM A.		X	X
IPI00783689		IMMUNGLOBULIN HEAVY CHAIN VARIABLE REGION (FRAGMENT).		X	X
IPI00783818		IMMUNGLOBULIN HEAVY CHAIN VARIABLE REGION (FRAGMENT).		X	X
IPI00784044	MCCC2	ISOFORM 1 OF METHYLCROTONOYL-COA CARBOXYLASE BETA CHAIN, MITOCHONDRIAL PRECURSOR.		X	X
IPI00784258	LTBP1	LATENT TRANSFORMING GROWTH FACTOR BETA BINDING PROTEIN 1 ISOFORM LTBP-1L.		X	X
IPI00784368	NDST1	ISOFORM 1 OF BIFUNCTIONAL HEPARAN SULFATE N-DEACETYLASE/N-SULFOTRANSFERASE 1.		X	X
IPI00784773		PUTATIVE UNCHARACTERIZED PROTEIN.		X	X
IPI00784894		PUTATIVE UNCHARACTERIZED PROTEIN.		X	X

Table S1. Proteins Identified or Not in MS Patients Compared to Normals and Other Neurologic Disease (OND)					
**IPI**	**Gene**	**Protein Name**	**MS**	**Normal**	**OND**
IPI00784985	IGK@	IGK@ PROTEIN.		X	X
IPI00785084	IGHV4-31	IMMUNOGLOBULIN HEAVY VARIABLE 4-31.		X	X
IPI00785196		PUTATIVE UNCHARACTERIZED PROTEIN.		X	X
IPI00785200		PUTATIVE UNCHARACTERIZED PROTEIN.		X	X
IPI00786893	LOC728998; LOC730296	SIMILAR TO LYRIC/3D3.		X	X
IPI00787050	NPTX1	SIMILAR TO NEURONAL PENTRAXIN I PRECURSOR.		X	X
IPI00787265	LOC729034	SIMILAR TO AMINOPEPTIDASE PUROMYCIN SENSITIVE.		X	X
IPI00787781	TIMP2	SIMILAR TO METALLOPROTEINASE INHIBITOR 2 PRECURSOR.		X	X
IPI00787936	LOC644021	SIMILAR TO CATHEPSIN L-LIKE PROTEIN.		X	X
IPI00788189	FCGBP	SIMILAR TO FC FRAGMENT OF IGG BINDING PROTEIN.		X	X
IPI00788786	VWF	309 KDA PROTEIN.		X	X
IPI00788824	IGLV1-44	LIGHT CHAIN FAB.		X	X
IPI00788835	PENK	25 KDA PROTEIN.		X	X
IPI00789398	LY75	ISOFORM 3 OF LYMPHOCYTE ANTIGEN 75 PRECURSOR.		X	X
IPI00789477	LTF	SIMILAR TO LACTOTRANSFERRIN PRECURSOR.		X	X
IPI00789847		PROTEIN.		X	X
IPI00789954	TF	7 KDA PROTEIN.		X	X
IPI00790473	SERPINF1	12 KDA PROTEIN.		X	X
IPI00790775	CACNA2D3	ISOFORM 3 OF VOLTAGE-DEPENDENT CALCIUM CHANNEL SUBUNIT ALPHA-2/DELTA-3 PRECURSOR.		X	X
IPI00790899		55 KDA PROTEIN.		X	X
IPI00791134	CLSTN2	CALSYNTENIN 2.		X	X
IPI00791228	GRIA4	GLUTAMATE RECEPTOR, IONOTROPHIC, AMPA 4 ISOFORM 3 PRECURSOR.		X	X
IPI00791343		261 KDA PROTEIN.		X	X
IPI00791479	ROBO2	CDNA FLJ90299 FIS, CLONE NT2RP2000514, HIGHLY SIMILAR TO HOMO SAPIENS ROUNDABOUT 2 (ROBO2) MRNA.		X	X
IPI00792115	CLEC3B	PUTATIVE UNCHARACTERIZED PROTEIN DKFZP686H17246.		X	X
IPI00792626	TF	14 KDA PROTEIN.		X	X
IPI00794070	CFI	CFI PROTEIN.		X	X
IPI00794450	LYNX1	9 KDA PROTEIN.		X	X
IPI00795013	LPHN3	149 KDA PROTEIN.		X	X
IPI00795055	C1RL	CDNA FLJ14022 FIS, CLONE HEMBA1003538, WEAKLY SIMILAR TO COMPLEMENT C1R COMPONENT.		X	X

Table S1. Proteins Identified or Not in MS Patients Compared to Normals and Other Neurologic Disease (OND)					
IPI	Gene	Protein Name	MS	Normal	OND
IPI00795481	LYPD1	ISOFORM 1 OF LY6/PLAUR DOMAIN-CONTAINING PROTEIN 1 PRECURSOR.		X	X
IPI00796279	SERPINF1	25 KDA PROTEIN.		X	X
IPI00797310	CLSTN3	14 KDA PROTEIN.		X	X
IPI00798430	TF	TRANSFERRIN VARIANT (FRAGMENT).		X	X
IPI00807609	CLEC11A	ABERRANT LSLCL.		X	X
IPI00815938	IGLV3-21	IGLV3-21 PROTEIN.		X	X
IPI00816155	C1QL3	ISOFORM 2 OF COMPLEMENT C1Q-LIKE PROTEIN 3 PRECURSOR.		X	X
IPI00816741	C5	COMPLEMENT COMPONENT 5 VARIANT (FRAGMENT).		X	X
IPI00816799		RHEUMATOID FACTOR D5 LIGHT CHAIN (FRAGMENT).		X	X
IPI00827482		UNCHARACTERIZED PROTEIN ENSP00000348964 (FRAGMENT).		X	X
IPI00827650	CD44	ISOFORM 3 OF CD44 ANTIGEN PRECURSOR.		X	X
IPI00827788		VH-3 FAMILY (VH26)D/J PROTEIN (FRAGMENT).		X	X
IPI00827829		HRV FAB N8-VL (FRAGMENT).		X	X
IPI00827846		ANTI-MUCIN1 HEAVY CHAIN VARIABLE REGION (FRAGMENT).		X	X
IPI00827891		COLD AGGLUTININ FS-2 H-CHAIN (FRAGMENT).		X	X
IPI00827929		VH-3 FAMILY (VH26)D/J PROTEIN (FRAGMENT).		X	X
IPI00827940		MU-CHAIN PRECURSOR (FRAGMENT).		X	X
IPI00828037		HEAVY CHAIN FAB (FRAGMENT).		X	X
IPI00828156		NANUC-1 HEAVY CHAIN (FRAGMENT).		X	X
IPI00828191		NANUC-2 HEAVY CHAIN (FRAGMENT).		X	X
IPI00829590		UNCHARACTERIZED PROTEIN ENSP00000375044.		X	X
IPI00829626	IGL@	IGL@ PROTEIN.		X	X
IPI00829640	IGL@	IGL@ PROTEIN.		X	X
IPI00829663		UNCHARACTERIZED PROTEIN ENSP00000374801.		X	X
IPI00829701		UNCHARACTERIZED PROTEIN ENSP00000375014.		X	X
IPI00829740		V2-6 PROTEIN.		X	X
IPI00829752		UNCHARACTERIZED PROTEIN ENSP00000375029.		X	X
IPI00829759		UNCHARACTERIZED PROTEIN ENSP00000375040.		X	X
IPI00829841		13 KDA PROTEIN.		X	X
IPI00829947		13 KDA PROTEIN.		X	X
IPI00830044		UNCHARACTERIZED PROTEIN ENSP00000374806.		X	X
IPI00830047		UNCHARACTERIZED PROTEIN ENSP00000374858 (FRAGMENT).		X	X
IPI00830107	IGLV5-45	V4-2 PROTEIN.		X	X
IPI00830132	IGHG4	UNCHARACTERIZED PROTEIN IGHG4 (FRAGMENT).		X	X

Table S1. Proteins Identified or Not in MS Patients Compared to Normals and Other Neurologic Disease (OND)					
IPI	Gene	Protein Name	MS	Normal	OND
IPI00845229	DEPDC2	ISOFORM 2 OF DEP DOMAIN-CONTAINING PROTEIN 2.		X	X
IPI00847179	APOA4	APOLIPOPROTEIN A-IV PRECURSOR.		X	X
IPI00847652	LOC400891	CDNA FLJ46805 FIS, CLONE TRACH3033535.		X	X
IPI00847670	LOC440043	SIMILAR TO PHOSPHOGLYCERATE MUTASE 1.		X	X
IPI00847723		SIMILAR TO VH4 HEAVY CHAIN VARIABLE REGION PRECURSOR.		X	X
IPI00853073	GPX3	PROTEIN.		X	X
IPI00853400	FKBP15	ISOFORM 1 OF FK506-BINDING PROTEIN 15.		X	X
IPI00853454	LAMB1	200 KDA PROTEIN.		X	X
IPI00853525	APOA1	APOLIPOPROTEIN A1.		X	X
IPI00854667		UNCHARACTERIZED PROTEIN ENSP00000375015.		X	X
IPI00854707		IMMUNGLOBULIN HEAVY CHAIN VARIABLE REGION (FRAGMENT).		X	X
IPI00854709		UNCHARACTERIZED PROTEIN ENSP00000374799 (FRAGMENT).		X	X
IPI00854743		UNCHARACTERIZED PROTEIN ENSP00000375034.		X	X
IPI00854806	IGKV1-5	IGKV1-5 PROTEIN.		X	X
IPI00855725	TGOLN2	ISOFORM 4 OF TRANS-GOLGI NETWORK INTEGRAL MEMBRANE PROTEIN 2 PRECURSOR.		X	X
IPI00867509	CORO1C	CORONIN-1C_I3 PROTEIN.		X	X
IPI00867665		SIMILAR TO PROTEIN DISULFIDE-ISOMERASE PRECURSOR.		X	X
IPI00871227	HMCN1	ISOFORM 1 OF HEMICENTIN-1 PRECURSOR.		X	X
IPI00872555	CFI	CDNA FLJ76262, HIGHLY SIMILAR TO HOMO SAPIENS I FACTOR (COMPLEMENT) (IF), MRNA.		X	X
IPI00873344	TPD52	N8 PROTEIN LONG ISOFORM (FRAGMENT).		X	X
IPI00873863	BDNF	BRAIN-DERIVED NEUROTROPHIC FACTOR TRANSCRIPT VARIANT 5.		X	X
IPI00874156	OTUB1	ISOFORM 1 OF UBIQUITIN THIOESTERASE OTUB1.		X	X
IPI00877029	FGA	FGA PROTEIN.		X	X
IPI00877800		32 KDA PROTEIN.		X	X
IPI00878517	ALB	56 KDA PROTEIN.		X	X
IPI00878576		AUTOTAXIN ISOFORM GAMMA.		X	X
IPI00879084	CP	20 KDA PROTEIN.		X	X
IPI00879309	NRXN2	PROTEIN.		X	X
IPI00879575		71 KDA PROTEIN.		X	X
IPI00879665	SEZ6L	112 KDA PROTEIN.		X	X
IPI00883711		SIMILAR TO ANTI-(ED-B) SCFV.		X	X
IPI00883772	GAA	ACID ALPHA-GLUCOSIDASE PREPROPROTEIN.		X	X

Table S1. Proteins Identified or Not in MS Patients Compared to Normals and Other Neurologic Disease (OND)					
**IPI**	**Gene**	**Protein Name**	**MS**	**Normal**	**OND**
IPI00883879		SIMILAR TO ANTI-IFN-G SCFV.		X	X
IPI00884004		RHEUMATOID FACTOR RF-ET12 (FRAGMENT).		X	X
IPI00884092		ANTI-HER3 SCFV (FRAGMENT).		X	X
IPI00000076	NGFB	BETA-NERVE GROWTH FACTOR PRECURSOR.		X	
IPI00000792	CRYZ	QUINONE OXIDOREDUCTASE.		X	
IPI00000811	PSMB6	PROTEASOME SUBUNIT BETA TYPE-6 PRECURSOR.		X	
IPI00000977	MAP3K11	MITOGEN-ACTIVATED PROTEIN KINASE KINASE KINASE 11.		X	
IPI00001433	PCDHB15	PROTOCADHERIN BETA 15 PRECURSOR.		X	
IPI00001434	PCDHB14	PROTOCADHERIN BETA 14 PRECURSOR.		X	
IPI00001568	ATP6V1D	VACUOLAR PROTON PUMP SUBUNIT D.		X	
IPI00001786	USP36	ISOFORM 2 OF UBIQUITIN CARBOXYL-TERMINAL HYDROLASE 36.		X	
IPI00001796	TNFRSF18	TUMOR NECROSIS FACTOR RECEPTOR SUPERFAMILY, MEMBER 18 (FRAGMENT).		X	
IPI00001869	PAPPA	PAPPALYSIN-1 PRECURSOR.		X	
IPI00001872	PCDHGA12; PCDHGC3	ISOFORM 1 OF PROTOCADHERIN GAMMA C3 PRECURSOR.		X	
IPI00002191		PUTATIVE UNCHARACTERIZED PROTEIN FLJ12684.		X	
IPI00002243	GGTLA1	ISOFORM 1 OF GAMMA-GLUTAMYLTRANSFERASE 5 PRECURSOR.		X	
IPI00002478	ECE1	ISOFORM B OF ENDOTHELIN-CONVERTING ENZYME 1.		X	
IPI00002491	SORBS1	ISOFORM 9 OF SORBIN AND SH3 DOMAIN-CONTAINING PROTEIN 1.		X	
IPI00002511	ATF6	CYCLIC AMP-DEPENDENT TRANSCRIPTION FACTOR ATF-6 ALPHA.		X	
IPI00002884	FLJ22222	CDNA: FLJ22222 FIS, CLONE HRC01658.		X	
IPI00002966	HSPA4	HEAT SHOCK 70 KDA PROTEIN 4.		X	
IPI00002993	TAF9	TRANSCRIPTION INITIATION FACTOR TFIID SUBUNIT 9.		X	
IPI00003353	C5orf13	NEURONAL PROTEIN 3.1.		X	
IPI00003391	ODZ1	TENEURIN-1.		X	
IPI00003392	TMEM5	TRANSMEMBRANE PROTEIN 5.		X	
IPI00003406	DBN1	ISOFORM 1 OF DREBRIN.		X	
IPI00003814	MAP2K6	ISOFORM 1 OF DUAL SPECIFICITY MITOGEN-ACTIVATED PROTEIN KINASE KINASE 6.		X	
IPI00003921	EPB41	ISOFORM 1 OF PROTEIN 4.1.		X	
IPI00004488	ATP6V1F	VACUOLAR PROTON PUMP SUBUNIT F.		X	
IPI00004533	KIF3B	KINESIN-LIKE PROTEIN KIF3B.		X	
IPI00004534	PFAS	PHOSPHORIBOSYLFORMYLGLYCINAMIDINE SYNTHASE.		X	
IPI00005107	NPC1	NIEMANN-PICK C1 PROTEIN PRECURSOR.		X	
IPI00005132	GNL3L	GUANINE NUCLEOTIDE-BINDING PROTEIN-LIKE 3-LIKE PROTEIN.		X	

Table S1. Proteins Identified or Not in MS Patients Compared to Normals and Other Neurologic Disease (OND)					
**IPI**	**Gene**	**Protein Name**	**MS**	**Normal**	**OND**
IPI00005347	ZRANB1	ZINC FINGER RAN-BINDING DOMAIN-CONTAINING PROTEIN 1.		X	
IPI00005600	NDST2	ISOFORM 1 OF BIFUNCTIONAL HEPARAN SULFATE N-DEACETYLASE/N-SULFOTRANSFERASE 2.		X	
IPI00005607	DBC1	ISOFORM 1 OF DELETED IN BLADDER CANCER PROTEIN 1 PRECURSOR.		X	
IPI00005614	SPTBN1	ISOFORM LONG OF SPECTRIN BETA CHAIN, BRAIN 1.		X	
IPI00005705	PPP1CC	ISOFORM GAMMA-1 OF SERINE/THREONINE-PROTEIN PHOSPHATASE PP1-GAMMA CATALYTIC SUBUNIT.		X	
IPI00005776	NOD1	NUCLEOTIDE-BINDING OLIGOMERIZATION DOMAIN-CONTAINING PROTEIN 1.		X	
IPI00005809	SDPR	SERUM DEPRIVATION-RESPONSE PROTEIN.		X	
IPI00005837	ANGPTL1	ANGIOPOIETIN-RELATED PROTEIN 1 PRECURSOR.		X	
IPI00005969	CAPZA1	F-ACTIN-CAPPING PROTEIN SUBUNIT ALPHA-1.		X	
IPI00006054	SNPH	SYNTAPHILIN.		X	
IPI00006094	RIMS3	REGULATING SYNAPTIC MEMBRANE EXOCYTOSIS PROTEIN 3.		X	
IPI00006252	SCYE1	MULTISYNTHETASE COMPLEX AUXILIARY COMPONENT P43.		X	
IPI00006746	ERMN	ERMIN.		X	
IPI00006900	UTP3	SOMETHING ABOUT SILENCING PROTEIN 10.		X	
IPI00007010	LYZL6	LYSOZYME-LIKE PROTEIN 6 PRECURSOR.		X	
IPI00007040	ZNF222	ZINC FINGER PROTEIN 222.		X	
IPI00007047	S100A8	PROTEIN S100-A8.		X	
IPI00007277	LRRFIP2	ISOFORM 1 OF LEUCINE-RICH REPEAT FLIGHTLESS-INTERACTING PROTEIN 2.		X	
IPI00007321	LYPLA1	ISOFORM 1 OF ACYL-PROTEIN THIOESTERASE 1.		X	
IPI00007402	IPO7	IMPORTIN-7.		X	
IPI00007512	GSTO2	GLUTATHIONE TRANSFERASE OMEGA-2.		X	
IPI00007682	ATP6V1A	VACUOLAR ATP SYNTHASE CATALYTIC SUBUNIT A.		X	
IPI00007812	ATP6V1B2	VACUOLAR ATP SYNTHASE SUBUNIT B, BRAIN ISOFORM.		X	
IPI00007834	ANK2	ISOFORM 1 OF ANKYRIN-2.		X	
IPI00008202	HECA	HEADCASE PROTEIN HOMOLOG.		X	
IPI00008226	THSD3	73 KDA PROTEIN.		X	
IPI00008422	SMARCAD1	ISOFORM 2 OF SWI/SNF-RELATED MATRIX-ASSOCIATED ACTIN-DEPENDENT REGULATOR OF CHROMATIN SUBFAMILY A CONTAINING DEAD/H BOX 1.		X	
IPI00008433	RPS5	40S RIBOSOMAL PROTEIN S5.		X	
IPI00008438	RPS10	40S RIBOSOMAL PROTEIN S10.		X	
IPI00008497	ODC1	ORNITHINE DECARBOXYLASE.		X	
IPI00008580	SLPI	ANTILEUKOPROTEINASE PRECURSOR.		X	

Table S1. Proteins Identified or Not in MS Patients Compared to Normals and Other Neurologic Disease (OND)					
IPI	Gene	Protein Name	MS	Normal	OND
IPI00008756	DST	ISOFORM 1 OF BULLOUS PEMPHIGOID ANTIGEN 1, ISOFORMS 1/2/3/4/5/8 (FRAGMENT).		X	
IPI00008894	CPA4	CARBOXYPEPTIDASE A4 PRECURSOR.		X	
IPI00008905	UGT2B15	UDP-GLUCURONOSYLTRANSFERASE 2B15 PRECURSOR.		X	
IPI00008998	PTPLAD1	PROTEIN TYROSINE PHOSPHATASE-LIKE PROTEIN PTPLAD1.		X	
IPI00009070	HBS1L	ISOFORM 1 OF HBS1-LIKE PROTEIN.		X	
IPI00009203	SNX7	SORTING NEXIN-7.		X	
IPI00009335	C8orf30A	BRAIN PROTEIN 16.		X	
IPI00009377	C3orf19	HSPC212.		X	
IPI00009396	CNR1	ISOFORM 1 OF CANNABINOID RECEPTOR 1.		X	
IPI00009439	SYT1	SYNAPTOTAGMIN-1.		X	
IPI00009471	WDR3	WD REPEAT-CONTAINING PROTEIN 3.		X	
IPI00009532	ABAT	4-AMINOBUTYRATE AMINOTRANSFERASE, MITOCHONDRIAL PRECURSOR.		X	
IPI00009899	C5orf5	UNCHARACTERIZED PROTEIN C5ORF5.		X	
IPI00010118	PTOV1	ISOFORM 1 OF PROSTATE TUMOR OVEREXPRESSED GENE 1 PROTEIN.		X	
IPI00010346	NLN	NEUROLYSIN, MITOCHONDRIAL PRECURSOR.		X	
IPI00010442	FXYD1; FXYD7	PHOSPHOLEMMAN PRECURSOR.		X	
IPI00010575	KIAA1466	KIAA1466 PROTEIN.		X	
IPI00010737	THBD	THROMBOMODULIN PRECURSOR.		X	
IPI00010808	IFNGR1	INTERFERON-GAMMA RECEPTOR ALPHA CHAIN PRECURSOR.		X	
IPI00010895	TULP2	TUBBY-RELATED PROTEIN 2.		X	
IPI00010903	DOPEY1	DOPEY FAMILY MEMBER 1.		X	
IPI00011051	TLX1	T-CELL LEUKEMIA HOMEOBOX PROTEIN 1.		X	
IPI00011416	ECH1	DELTA(3,5)-DELTA(2,4)-DIENOYL-COA ISOMERASE, MITOCHONDRIAL PRECURSOR.		X	
IPI00011865	PDGFD	ISOFORM 2 OF PLATELET-DERIVED GROWTH FACTOR D PRECURSOR.		X	
IPI00012315	NME3	NUCLEOSIDE DIPHOSPHATE KINASE 3.		X	
IPI00012895	CA12	ISOFORM 1 OF CARBONIC ANHYDRASE 12 PRECURSOR.		X	
IPI00013004	PDXK	ISOFORM 1 OF PYRIDOXAL KINASE.		X	
IPI00013219	ILK	INTEGRIN-LINKED PROTEIN KINASE.		X	
IPI00013455	CLIP1	CLIP1 PROTEIN.		X	
IPI00013466	ASNA1	ARSENICAL PUMP-DRIVING ATPASE.		X	
IPI00013681	TRPC3	N-TERMINALLY EXTENDED TYPE 3 CANONICAL TRANSIENT RECEPTOR POTENTIAL CHANNEL.		X	

Table S1. Proteins Identified or Not in MS Patients Compared to Normals and Other Neurologic Disease (OND)					
**IPI**	**Gene**	**Protein Name**	**MS**	**Normal**	**OND**
IPI00013749	PRKCZ	PROTEIN KINASE C ZETA TYPE.		X	
IPI00013847	UQCRC1	CYTOCHROME B-C1 COMPLEX SUBUNIT 1, MITOCHONDRIAL PRECURSOR.		X	
IPI00013945	UMOD	ISOFORM 1 OF UROMODULIN PRECURSOR.		X	
IPI00014340	PPP1R12C	ISOFORM 1 OF PROTEIN PHOSPHATASE 1 REGULATORY SUBUNIT 12C.		X	
IPI00014444	SERAC1	ISOFORM 1 OF PROTEIN SERAC1.		X	
IPI00014516	CALD1	ISOFORM 1 OF CALDESMON.		X	
IPI00014537	CALU	ISOFORM 1 OF CALUMENIN PRECURSOR.		X	
IPI00014899	BRPF3	CDNA FLJ20744 FIS, CLONE HEP06585.		X	
IPI00015047	CD320	8D6 ANTIGEN (FRAGMENT).		X	
IPI00015148	RAP1B	RAS-RELATED PROTEIN RAP-1B PRECURSOR.		X	
IPI00015522	GDF5	GROWTH/DIFFERENTIATION FACTOR 5 PRECURSOR.		X	
IPI00015913	TYRP1	5,6-DIHYDROXYINDOLE-2-CARBOXYLIC ACID OXIDASE PRECURSOR.		X	
IPI00015954	SAR1A	GTP-BINDING PROTEIN SAR1A.		X	
IPI00015973	EPB41L2	BAND 4.1-LIKE PROTEIN 2.		X	
IPI00015980	MPDZ	ISOFORM 2 OF MULTIPLE PDZ DOMAIN PROTEIN.		X	
IPI00015983	EDG3	SPHINGOSINE 1-PHOSPHATE RECEPTOR EDG-3.		X	
IPI00016112	PXDN	PEROXIDASIN HOMOLOG.		X	
IPI00016576	GRHL2	ISOFORM 1 OF GRAINYHEAD-LIKE PROTEIN 2 HOMOLOG.		X	
IPI00016577	CD22	CDNA: FLJ22814 FIS, CLONE KAIA3004.		X	
IPI00016605	C1orf123	UNCHARACTERIZED PROTEIN C1ORF123.		X	
IPI00016685	ENAM	ENAMELIN PRECURSOR.		X	
IPI00016701	P2RY14	P2Y PURINOCEPTOR 14.		X	
IPI00017659	RCSD1	PROTEIN KINASE SUBSTRATE CAPZIP.		X	
IPI00017940	LMBRD2	LMBR1 DOMAIN-CONTAINING PROTEIN 2.		X	
IPI00017964	SNRPD3	SMALL NUCLEAR RIBONUCLEOPROTEIN SM D3.		X	
IPI00018098	PRPF38B	ISOFORM 1 OF PRE-MRNA-SPLICING FACTOR 38B.		X	
IPI00018208	TTC33	TETRATRICOPEPTIDE REPEAT PROTEIN 33.		X	
IPI00018387	FURIN	FURIN PRECURSOR.		X	
IPI00018429	PRRX2	PAIRED MESODERM HOMEOBOX PROTEIN 2.		X	
IPI00018755	HMG1L10	HIGH MOBILITY GROUP PROTEIN 1-LIKE 10.		X	
IPI00018803	HOXD12	HOMEOBOX D12.		X	
IPI00018843	DRD3	ISOFORM 1 OF D(3) DOPAMINE RECEPTOR.		X	
IPI00018879	IDUA	ALPHA-L-IDURONIDASE PRECURSOR.		X	
IPI00018914	PTPN14	TYROSINE-PROTEIN PHOSPHATASE NON-RECEPTOR TYPE 14.		X	

Table S1. Proteins Identified or Not in MS Patients Compared to Normals and Other Neurologic Disease (OND)					
**IPI**	**Gene**	**Protein Name**	**MS**	**Normal**	**OND**
IPI00019158	ADAM8	ADAM METALLOPEPTIDASE DOMAIN 8 PRECURSOR.		X	
IPI00019208		SIMILAR TO 60S RIBOSOMAL PROTEIN L29.		X	
IPI00019242	MMP15	MATRIX METALLOPROTEINASE-15 PRECURSOR.		X	
IPI00019888	ALDH5A1	SUCCINATE-SEMIALDEHYDE DEHYDROGENASE, MITOCHONDRIAL PRECURSOR.		X	
IPI00019901	ADD1	ISOFORM 1 OF ALPHA-ADDUCIN.		X	
IPI00019904	ADD2	ISOFORM 1 OF BETA-ADDUCIN.		X	
IPI00019988	SGSH	N-SULPHOGLUCOSAMINE SULPHOHYDROLASE PRECURSOR.		X	
IPI00020058	ATP7B	ISOFORM 1 OF COPPER-TRANSPORTING ATPASE 2.		X	
IPI00020199	ST8SIA2	ALPHA-2,8-SIALYLTRANSFERASE 8B.		X	
IPI00020356	MAP1A	331 KDA PROTEIN.		X	
IPI00020470	GLT8D1	ISOFORM 1 OF GLYCOSYLTRANSFERASE 8 DOMAIN-CONTAINING PROTEIN 1.		X	
IPI00020966	PIGA	ISOFORM 1 OF PHOSPHATIDYLINOSITOL N-ACETYLGLUCOSAMINYLTRANSFERASE SUBUNIT A.		X	
IPI00021347	UBE2L3	UBIQUITIN-CONJUGATING ENZYME E2 L3.		X	
IPI00021476	EIF4EBP3	EUKARYOTIC TRANSLATION INITIATION FACTOR 4E-BINDING PROTEIN 3.		X	
IPI00021594	GPAA1	ISOFORM 1 OF GLYCOSYLPHOSPHATIDYLINOSITOL ANCHOR ATTACHMENT 1 PROTEIN.		X	
IPI00021634	KLC2	KINESIN LIGHT CHAIN 2.		X	
IPI00021753	KIF13B	KINESIN-LIKE PROTEIN KIF13B.		X	
IPI00021770	HMGCR	ISOFORM 1 OF 3-HYDROXY-3-METHYLGLUTARYL-COENZYME A REDUCTASE.		X	
IPI00021907	MBP	ISOFORM 1 OF MYELIN BASIC PROTEIN.		X	
IPI00021951	KIAA0247	UNCHARACTERIZED PROTEIN KIAA0247 PRECURSOR.		X	
IPI00022277	CCDC56	COILED-COIL DOMAIN-CONTAINING PROTEIN 56.		X	
IPI00022361	SLC4A1	BAND 3 ANION TRANSPORT PROTEIN.		X	
IPI00022606	PSTPIP1	ISOFORM 1 OF PROLINE-SERINE-THREONINE PHOSPHATASE-INTERACTING PROTEIN 1.		X	
IPI00022958	C16orf72	PRO0149.		X	
IPI00022989	RARB	ISOFORM BETA-1 OF RETINOIC ACID RECEPTOR BETA.		X	
IPI00023087	UBE2T	UBIQUITIN-CONJUGATING ENZYME E2 T.		X	
IPI00023152	NAALADL1	ISOFORM 1 OF N-ACETYLATED-ALPHA-LINKED ACIDIC DIPEPTIDASE-LIKE PROTEIN.		X	
IPI00023162	GNE	UDP-N-ACETYLGLUCOSAMINE 2-EPIMERASE/N-ACETYLMANNOSAMINE KINASE.		X	
IPI00023184	PARP3	ISOFORM 1 OF POLY [ADP-RIBOSE] POLYMERASE 3.		X	
IPI00023217	RYR2	ISOFORM 1 OF RYANODINE RECEPTOR 2.		X	
IPI00023322	DPF2	ZINC FINGER PROTEIN UBI-D4.		X	

Table S1. Proteins Identified or Not in MS Patients Compared to Normals and Other Neurologic Disease (OND)					
**IPI**	**Gene**	**Protein Name**	**MS**	**Normal**	**OND**
IPI00023340	MYST3	HISTONE ACETYLTRANSFERASE MYST3.		X	
IPI00023407	NCKAP1L	NCK-ASSOCIATED PROTEIN 1-LIKE.		X	
IPI00023513	CHFR	ISOFORM 1 OF E3 UBIQUITIN-PROTEIN LIGASE CHFR.		X	
IPI00023780	DNAJC5	ISOFORM 2 OF DNAJ HOMOLOG SUBFAMILY C MEMBER 5.		X	
IPI00023942	SDC3	ISOFORM 2 OF SYNDECAN-3.		X	
IPI00024094	RHAG	RHESUS BLOOD GROUP-ASSOCIATED GLYCOPROTEIN.		X	
IPI00024253	FGF14	ISOFORM 1 OF FIBROBLAST GROWTH FACTOR 14.		X	
IPI00024346	SNAPC3	SNRNA-ACTIVATING PROTEIN COMPLEX SUBUNIT 3.		X	
IPI00024502	UBQLN4	UBIQUILIN-4.		X	
IPI00024662	CBX5	CHROMOBOX PROTEIN HOMOLOG 5.		X	
IPI00024664	USP5	ISOFORM LONG OF UBIQUITIN CARBOXYL-TERMINAL HYDROLASE 5.		X	
IPI00024704	UST	URONYL 2-SULFOTRANSFERASE.		X	
IPI00024818	USPL1	ISOFORM 1 OF UBIQUITIN-SPECIFIC PEPTIDASE-LIKE PROTEIN 1.		X	
IPI00024920	ATP5D	ATP SYNTHASE SUBUNIT DELTA, MITOCHONDRIAL PRECURSOR.		X	
IPI00024976	TOMM22	MITOCHONDRIAL IMPORT RECEPTOR SUBUNIT TOM22 HOMOLOG.		X	
IPI00025019	PSMB1	PROTEASOME SUBUNIT BETA TYPE-1 PRECURSOR.		X	
IPI00025094	MYH16	CDNA: FLJ22037 FIS, CLONE HEP08868 (FRAGMENT).		X	
IPI00025622	ZFAND5	AN1-TYPE ZINC FINGER PROTEIN 5.		X	
IPI00025700	CD6	ISOFORM CD6A OF T-CELL DIFFERENTIATION ANTIGEN CD6 PRECURSOR.		X	
IPI00026031	C6orf72	UNCHARACTERIZED PROTEIN C6ORF72 PRECURSOR.		X	
IPI00026241	BST2	BONE MARROW STROMAL ANTIGEN 2 PRECURSOR.		X	
IPI00026268	GNB1	GUANINE NUCLEOTIDE-BINDING PROTEIN G(I)/G(S)/G(T) SUBUNIT BETA-1.		X	
IPI00026299	GYPC	ISOFORM GLYCOPHORIN C OF GLYCOPHORIN-C.		X	
IPI00026570	COX7A2	CYTOCHROME C OXIDASE POLYPEPTIDE VIIA-LIVER/HEART, MITOCHONDRIAL PRECURSOR.		X	
IPI00026612	PPM1B	ISOFORM BETA-1 OF PROTEIN PHOSPHATASE 1B.		X	
IPI00026665	QARS	GLUTAMINYL-TRNA SYNTHETASE.		X	
IPI00027009	PACSIN2	ISOFORM 1 OF PROTEIN KINASE C AND CASEIN KINASE SUBSTRATE IN NEURONS PROTEIN 2.		X	
IPI00027248	TUSC2	TUMOR SUPPRESSOR CANDIDATE 2.		X	
IPI00027255	MYL6; MYL6B	MYOSIN LIGHT POLYPEPTIDE 6B.		X	
IPI00027438	FLOT1	FLOTILLIN-1.		X	
IPI00027626	CCT6A	T-COMPLEX PROTEIN 1 SUBUNIT ZETA.		X	
IPI00027685	CCR1	C-C CHEMOKINE RECEPTOR TYPE 1.		X	

Table S1. Proteins Identified or Not in MS Patients Compared to Normals and Other Neurologic Disease (OND)					
IPI	Gene	Protein Name	MS	Normal	OND
IPI00027726	KLF3	ISOFORM 1 OF KRUEPPEL-LIKE FACTOR 3.		X	
IPI00027782	MMP3	STROMELYSIN-1 PRECURSOR.		X	
IPI00027834	HNRNPL	HETEROGENEOUS NUCLEAR RIBONUCLEOPROTEIN L ISOFORM A.		X	
IPI00027898	C21orf70	ISOFORM A OF UNCHARACTERIZED PROTEIN C21ORF70.		X	
IPI00027984	DNAJC11	PUTATIVE UNCHARACTERIZED PROTEIN.		X	
IPI00028053	GJA9	GAP JUNCTION ALPHA-9 PROTEIN.		X	
IPI00028383	C16orf24	UNCHARACTERIZED PROTEIN C16ORF24.		X	
IPI00028481	RAB8A	RAS-RELATED PROTEIN RAB-8A.		X	
IPI00028520	NDUFV1	ISOFORM 1 OF NADH DEHYDROGENASE [UBIQUINONE] FLAVOPROTEIN 1, MITOCHONDRIAL PRECURSOR.		X	
IPI00028601	MT1P3	PUTATIVE METALLOTHIONEIN C20ORF127.		X	
IPI00028614	EPB42	ERYTHROCYTE MEMBRANE PROTEIN BAND 4.2 ISOFORM 2.		X	
IPI00028912	VEZF1	ZINC FINGER PROTEIN 161.		X	
IPI00029123	EDNRB	ISOFORM A OF ENDOTHELIN B RECEPTOR PRECURSOR.		X	
IPI00029175	KIAA0196	STRUMPELLIN.		X	
IPI00029533	ITGB8	INTEGRIN BETA-8 PRECURSOR.		X	
IPI00029556	C1orf105	UNCHARACTERIZED PROTEIN C1ORF105.		X	
IPI00029623	PSMA6	PROTEASOME SUBUNIT ALPHA TYPE-6.		X	
IPI00029647	ZG16	ZYMOGEN GRANULE MEMBRANE PROTEIN 16 PRECURSOR.		X	
IPI00030009	PAPSS2	ISOFORM A OF BIFUNCTIONAL 3'-PHOSPHOADENOSINE 5'-PHOSPHOSULFATE SYNTHETASE 2.		X	
IPI00030037	ASIP	AGOUTI-SIGNALING PROTEIN PRECURSOR.		X	
IPI00030319	FOXF2	FORKHEAD BOX PROTEIN F2.		X	
IPI00030706	AHSA1	ACTIVATOR OF 90 KDA HEAT SHOCK PROTEIN ATPASE HOMOLOG 1.		X	
IPI00030919	MAP2K1IP1	MITOGEN-ACTIVATED PROTEIN KINASE KINASE 1-INTERACTING PROTEIN 1.		X	
IPI00031005	FLJ23356	PROTEIN KINASE-LIKE PROTEIN SGK196.		X	
IPI00031485	MRP63	MITOCHONDRIAL RIBOSOMAL PROTEIN 63.		X	
IPI00031547	DSG3	DESMOGLEIN-3 PRECURSOR.		X	
IPI00031627	POLR2A	DNA-DIRECTED RNA POLYMERASE II SUBUNIT RPB1.		X	
IPI00032313	S100A4	PROTEIN S100-A4.		X	
IPI00032416	JAG2	ISOFORM LONG OF PROTEIN JAGGED-2 PRECURSOR.		X	
IPI00032425	RAMP3	RECEPTOR ACTIVITY-MODIFYING PROTEIN 3 PRECURSOR.		X	
IPI00032597	RBMX2	RNA-BINDING MOTIF PROTEIN, X-LINKED 2.		X	
IPI00040730	PCDH21	PROTOCADHERIN 21 PRECURSOR.		X	

Table S1. Proteins Identified or Not in MS Patients Compared to Normals and Other Neurologic Disease (OND)					
**IPI**	**Gene**	**Protein Name**	**MS**	**Normal**	**OND**
IPI00040900	HS2ST1	ISOFORM 2 OF HEPARAN SULFATE 2-O-SULFOTRANSFERASE 1.		X	
IPI00043731	RUNDC3B	CDNA FLJ30671 FIS, CLONE FCBBF1000687, MODERATELY SIMILAR TO MUS MUSCULUS RAP2 INTERACTING PROTEIN 8 (RPIP8) MRNA.		X	
IPI00043978	PARD3B	ISOFORM 1 OF PARTITIONING-DEFECTIVE 3 HOMOLOG B.		X	
IPI00044607	PPP1R2P1	PROTEIN PHOSPHATASE INHIBITOR 2-LIKE PROTEIN 1.		X	
IPI00044842	RAB3IP	ISOFORM 2 OF RAB3A-INTERACTING PROTEIN.		X	
IPI00045219	SNAG1	SORTING NEXIN-18.		X	
IPI00045360	CIC	CAPICUA-LIKE PROTEIN/DOUBLE HOMEODOMAIN 4 FUSION PROTEIN.		X	
IPI00045511	CLCC1	ISOFORM 1 OF CHLORIDE CHANNEL CLIC-LIKE PROTEIN 1 PRECURSOR.		X	
IPI00045928	SLC9A7	SODIUM/HYDROGEN EXCHANGER 7.		X	
IPI00045939	ADO	2-AMINOETHANETHIOL DIOXYGENASE.		X	
IPI00046057	STXBP1	ISOFORM 2 OF SYNTAXIN-BINDING PROTEIN 1.		X	
IPI00056314	TSR2	PRE-RRNA-PROCESSING PROTEIN TSR2 HOMOLOG.		X	
IPI00060146	SMCR7	ISOFORM 1 OF SMITH-MAGENIS SYNDROME CHROMOSOME REGION CANDIDATE GENE 7 PROTEIN.		X	
IPI00060265	ZNF775	ZINC FINGER PROTEIN 775.		X	
IPI00060546	C10orf35	UNCHARACTERIZED PROTEIN C10ORF35.		X	
IPI00062730	C16orf45	UNCHARACTERIZED PROTEIN C16ORF45.		X	
IPI00064241	IKZF4	ISOFORM 1 OF ZINC FINGER PROTEIN EOS.		X	
IPI00064296		PRO0633.		X	
IPI00065276	ASPSCR1	ISOFORM 2 OF TETHER CONTAINING UBX DOMAIN FOR GLUT4.		X	
IPI00071185	CDC2L1	ISOFORM SV1 OF PITSLRE SERINE/THREONINE-PROTEIN KINASE CDC2L1.		X	
IPI00071824	CKAP2	ISOFORM 1 OF CYTOSKELETON-ASSOCIATED PROTEIN 2.		X	
IPI00072044	C11orf54	ISOFORM 1 OF ESTER HYDROLASE C11ORF54.		X	
IPI00090764	TLR1	TOLL-LIKE RECEPTOR 1 PRECURSOR.		X	
IPI00098902	OGDH	2-OXOGLUTARATE DEHYDROGENASE E1 COMPONENT, MITOCHONDRIAL PRECURSOR.		X	
IPI00100154	TOLLIP	TOLL-INTERACTING PROTEIN.		X	
IPI00100980	EHD2	EH DOMAIN-CONTAINING PROTEIN 2.		X	
IPI00101927	LZTS2	LEUCINE ZIPPER PUTATIVE TUMOR SUPPRESSOR 2.		X	
IPI00102678	PCNX	ISOFORM 1 OF PECANEX-LIKE PROTEIN 1.		X	
IPI00102808	DUSP19	ISOFORM 1 OF DUAL SPECIFICITY PROTEIN PHOSPHATASE 19.		X	
IPI00103510	RXFP2	RELAXIN RECEPTOR 2.		X	
IPI00103630	PPM1E	ISOFORM 2 OF PROTEIN PHOSPHATASE 1E.		X	

Table S1. Proteins Identified or Not in MS Patients Compared to Normals and Other Neurologic Disease (OND)					
**IPI**	**Gene**	**Protein Name**	**MS**	**Normal**	**OND**
IPI00103874	ZFYVE1	ISOFORM 1 OF ZINC FINGER FYVE DOMAIN-CONTAINING PROTEIN 1.		X	
IPI00103891	MTMR14	PUTATIVE UNCHARACTERIZED PROTEIN.		X	
IPI00104907	C14orf106	ISOFORM 1 OF UNCHARACTERIZED POTENTIAL DNA-BINDING PROTEIN C14ORF106.		X	
IPI00106506	ECSIT	ISOFORM 1 OF EVOLUTIONARILY CONSERVED SIGNALING INTERMEDIATE IN TOLL PATHWAY, MITOCHONDRIAL PRECURSOR.		X	
IPI00144243	HIVEP2	HUMAN IMMUNODEFICIENCY VIRUS TYPE I ENHANCER-BINDING PROTEIN 2.		X	
IPI00145805	TNIK	ISOFORM 1 OF TRAF2 AND NCK-INTERACTING PROTEIN KINASE.		X	
IPI00149044	ZNF280D	ISOFORM 2 OF SUPPRESSOR OF HAIRY WING HOMOLOG 4.		X	
IPI00149375	C11orf56	ISOFORM 2 OF UNCHARACTERIZED PROTEIN C11ORF56.		X	
IPI00151990	TXNDC15	ISOFORM 1 OF THIOREDOXIN DOMAIN-CONTAINING PROTEIN 15 PRECURSOR.		X	
IPI00152050	A2BP1	ATAXIN 2-BINDING PROTEIN 1 ISOFORM 3.		X	
IPI00152072	FIBIN	HYPOTHETICAL PROTEIN LOC387758.		X	
IPI00152145	OSR1	PROTEIN ODD-SKIPPED-RELATED 1.		X	
IPI00152344	PHOSPHO2	PYRIDOXAL PHOSPHATE PHOSPHATASE PHOSPHO2.		X	
IPI00152470	PROKR1	PROKINETICIN RECEPTOR 1.		X	
IPI00152849	CCNB3	ISOFORM 1 OF G2/MITOTIC-SPECIFIC CYCLIN-B3.		X	
IPI00154528	SMC6	ISOFORM 1 OF STRUCTURAL MAINTENANCE OF CHROMOSOMES PROTEIN 6.		X	
IPI00155447	MMP28	MMP28 PROTEIN.		X	
IPI00158992	SNAPC4	SNRNA-ACTIVATING PROTEIN COMPLEX SUBUNIT 4.		X	
IPI00159049	SETBP1	SET-BINDING PROTEIN.		X	
IPI00160369	PICK1	PRKCA-BINDING PROTEIN.		X	
IPI00161119	NFKBIB	ISOFORM 1 OF NF-KAPPA-B INHIBITOR BETA.		X	
IPI00163391	METT10D	ISOFORM 1 OF PUTATIVE METHYLTRANSFERASE METT10D.		X	
IPI00163601	FLJ10213	PUTATIVE UNCHARACTERIZED PROTEIN FLJ10213.		X	
IPI00163851	EIF2AK4	ISOFORM 1 OF EUKARYOTIC TRANSLATION INITIATION FACTOR 2-ALPHA KINASE 4.		X	
IPI00164066	CCDC136	ISOFORM 4 OF COILED-COIL DOMAIN-CONTAINING PROTEIN 136.		X	
IPI00164861	KIF13A	ISOFORM 3 OF KINESIN-LIKE PROTEIN KIF13A.		X	
IPI00164949	TH1L	ISOFORM NELF-C OF NEGATIVE ELONGATION FACTOR C/D.		X	
IPI00166010	CNOT1	ISOFORM 1 OF CCR4-NOT TRANSCRIPTION COMPLEX SUBUNIT 1.		X	
IPI00166071	BCL6B	B-CELL CLL/LYMPHOMA 6 MEMBER B PROTEIN.		X	
IPI00166161	C14orf39	PROTEIN SIX6OS1.		X	
IPI00166257		CDNA FLJ37614 FIS, CLONE BRCOC2011769.		X	
IPI00166619	SVOPL	ISOFORM 2 OF PUTATIVE TRANSPORTER SVOPL.		X	
IPI00166776	CREG2	PROTEIN CREG2 PRECURSOR.		X	

Table S1. Proteins Identified or Not in MS Patients Compared to Normals and Other Neurologic Disease (OND)					
IPI	Gene	Protein Name	MS	Normal	OND
IPI00166817	ZNF561	ZINC FINGER PROTEIN 561.		X	
IPI00166865	CISD2	CDGSH IRON SULFUR DOMAIN-CONTAINING PROTEIN 2.		X	
IPI00167006	C13orf26	UNCHARACTERIZED PROTEIN C13ORF26.		X	
IPI00167089	TNK2	ISOFORM 2 OF ACTIVATED CDC42 KINASE 1.		X	
IPI00167137	SLAMF7	ISOFORM 3 OF SLAM FAMILY MEMBER 7 PRECURSOR.		X	
IPI00167154	MAPKBP1	UNCHARACTERIZED PROTEIN MAPKBP1.		X	
IPI00167560	PAPD4	PAP-ASSOCIATED DOMAIN-CONTAINING PROTEIN 4.		X	
IPI00167638	GTPBP10	ISOFORM 1 OF GTP-BINDING PROTEIN 10.		X	
IPI00168404	ZBTB34	ZINC FINGER AND BTB DOMAIN CONTAINING 34.		X	
IPI00169259	SVIP	SMALL VCP/P97-INTERACTING PROTEIN.		X	
IPI00169331	SGMS2	PHOSPHATIDYLCHOLINE:CERAMIDE CHOLINEPHOSPHOTRANSFERASE 2.		X	
IPI00171199	PSMA3	ISOFORM 2 OF PROTEASOME SUBUNIT ALPHA TYPE-3.		X	
IPI00171647	SIGLEC8	ISOFORM 1 OF SIALIC ACID-BINDING IG-LIKE LECTIN 8 PRECURSOR.		X	
IPI00171737	LRDD	ISOFORM 2 OF LEUCINE-RICH REPEAT AND DEATH DOMAIN-CONTAINING PROTEIN.		X	
IPI00175019	LOC643677	SIMILAR TO TEMPORARILY ASSIGNED GENE NAME FAMILY MEMBER.		X	
IPI00175989	MCF2L2	RHO FAMILY GUANINE-NUCLEOTIDE EXCHANGE FACTOR.		X	
IPI00177728	CNDP2	CYTOSOLIC NON-SPECIFIC DIPEPTIDASE.		X	
IPI00177878	TMEM16D	ISOFORM 3 OF TRANSMEMBRANE PROTEIN 16D.		X	
IPI00178727	C10orf79	NOVEL PROTEIN.		X	
IPI00178894	ZBTB20	ZINC FINGER AND BTB DOMAIN-CONTAINING PROTEIN 20.		X	
IPI00179589	MTPN	MYOTROPHIN.		X	
IPI00180386	GYG1	ISOFORM GN-1L OF GLYCOGENIN-1.		X	
IPI00180426	GRK4	ISOFORM 3 OF G PROTEIN-COUPLED RECEPTOR KINASE 4.		X	
IPI00181743	BAIAP3	ISOFORM 1 OF BAI1-ASSOCIATED PROTEIN 3.		X	
IPI00183206	RIMBP2	ISOFORM 1 OF RIM-BINDING PROTEIN 2.		X	
IPI00184884	NSMCE1	NON-STRUCTURAL MAINTENANCE OF CHROMOSOMES ELEMENT 1 HOMOLOG.		X	
IPI00185146	IPO9	IMPORTIN-9.		X	
IPI00186621	OFCC1	OROFACIAL CLEFTING CHROMOSOMAL BREAKPOINT REGION 1.		X	
IPI00186826	EPHB4	EPHRIN RECEPTOR.		X	
IPI00186966	BIN1	ISOFORM IIA OF MYC BOX-DEPENDENT-INTERACTING PROTEIN 1.		X	
IPI00215610	MPP1	55 KDA ERYTHROCYTE MEMBRANE PROTEIN.		X	
IPI00215777	SLC25A3	ISOFORM B OF PHOSPHATE CARRIER PROTEIN, MITOCHONDRIAL PRECURSOR.		X	
IPI00215914	ARF1	ADP-RIBOSYLATION FACTOR 1.		X	
IPI00216348	DYNC1I2	ISOFORM 2C OF CYTOPLASMIC DYNEIN 1 INTERMEDIATE CHAIN 2.		X	

Table S1. Proteins Identified or Not in MS Patients Compared to Normals and Other Neurologic Disease (OND)					
IPI	Gene	Protein Name	MS	Normal	OND
IPI00216470	PIP4K2B	ISOFORM 1 OF PHOSPHATIDYLINOSITOL-5-PHOSPHATE 4-KINASE TYPE-2 BETA.		X	
IPI00216508	SNX3	ISOFORM 2 OF SORTING NEXIN-3.		X	
IPI00216572	BARX2	BARH-LIKE HOMEOBOX 2.		X	
IPI00216651	IL28RA	ISOFORM 1 OF INTERLEUKIN-28 RECEPTOR ALPHA CHAIN PRECURSOR.		X	
IPI00216683	CDC25C	M-PHASE INDUCER PHOSPHATASE 3.		X	
IPI00216699	FERMT3	ISOFORM 2 OF UNC-112-RELATED PROTEIN 2.		X	
IPI00216704	SPTB	ISOFORM 2 OF SPECTRIN BETA CHAIN, ERYTHROCYTE.		X	
IPI00216914	VMO1	VITELLINE MEMBRANE OUTER LAYER PROTEIN 1 HOMOLOG PRECURSOR.		X	
IPI00216921	STMN4	ISOFORM 2 OF STATHMIN-4.		X	
IPI00216963	CFLAR	ISOFORM 9 OF CASP8 AND FADD-LIKE APOPTOSIS REGULATOR PRECURSOR.		X	
IPI00217012	PSD	PLECKSTRIN AND SEC7 DOMAIN CONTAINING.		X	
IPI00217258	CCDC100	CCDC100 PROTEIN.		X	
IPI00217405	UBR1	ISOFORM 1 OF E3 UBIQUITIN-PROTEIN LIGASE UBR1.		X	
IPI00217465	HIST1H1C	HISTONE H1.2.		X	
IPI00217467	HIST1H1E	HISTONE H1.4.		X	
IPI00217617	MPP7	PALMITOYLATED MEMBRANE PROTEIN 7.		X	
IPI00217740	C20orf12	C20ORF12 PROTEIN.		X	
IPI00217791	CCDC105	COILED-COIL DOMAIN-CONTAINING PROTEIN 105.		X	
IPI00217831	ANKRD13A	ANKYRIN REPEAT DOMAIN-CONTAINING PROTEIN 13A.		X	
IPI00217871	ALDH4A1	DELTA-1-PYRROLINE-5-CARBOXYLATE DEHYDROGENASE, MITOCHONDRIAL PRECURSOR.		X	
IPI00217948	FRMD4B	FRMD4B PROTEIN.		X	
IPI00217989	SCYL3	ISOFORM 1 OF PROTEIN-ASSOCIATING WITH THE CARBOXYL-TERMINAL DOMAIN OF EZRIN.		X	
IPI00218075	FAM9B	PROTEIN FAM9B.		X	
IPI00218130	PYGM	GLYCOGEN PHOSPHORYLASE, MUSCLE FORM.		X	
IPI00218131	S100A12	PROTEIN S100-A12.		X	
IPI00218628	ITGA2B	ISOFORM 2 OF INTEGRIN ALPHA-IIB PRECURSOR.		X	
IPI00218637	HLA-DQB2	MAJOR HISTOCOMPATIBILITY COMPLEX, CLASS II, DQ BETA 2.		X	
IPI00218918	ANXA1	ANNEXIN A1.		X	
IPI00219622	PSMA2	PROTEASOME SUBUNIT ALPHA TYPE-2.		X	
IPI00219682	STOM	ERYTHROCYTE BAND 7 INTEGRAL MEMBRANE PROTEIN.		X	
IPI00220070	PFKFB4	6-PHOSPHOFRUCTO-2-KINASE/FRUCTOSE-2,6-BIPHOSPHATASE 4.		X	
IPI00220156	TGFB2	ISOFORM B OF TRANSFORMING GROWTH FACTOR BETA-2 PRECURSOR.		X	

Table S1. Proteins Identified or Not in MS Patients Compared to Normals and Other Neurologic Disease (OND)					
**IPI**	**Gene**	**Protein Name**	**MS**	**Normal**	**OND**
IPI00220271	AKR1A1	ALCOHOL DEHYDROGENASE.		X	
IPI00220578	GNAI3	GUANINE NUCLEOTIDE-BINDING PROTEIN G.		X	
IPI00233358	ICA1L	ISLET CELL AUTOANTIGEN 1,69KDA-LIKE ISOFORM 2.		X	
IPI00235647	LOC345630	SIMILAR TO FIBRILLARIN.		X	
IPI00242905		UNCHARACTERIZED PROTEIN ENSP00000344689 (FRAGMENT).		X	
IPI00243338	KRT23	24 KDA PROTEIN.		X	
IPI00245940	IGSF5	IMMUNOGLOBULIN SUPERFAMILY 5 LIKE.		X	
IPI00249982	DIDO1	ISOFORM 1 OF DEATH-INDUCER OBLITERATOR 1.		X	
IPI00251507	SYN1	ISOFORM IB OF SYNAPSIN-1.		X	
IPI00251596	COL23A1	ISOFORM 1 OF COLLAGEN ALPHA-1(XXIII) CHAIN.		X	
IPI00252845	SYT9	SYT9 PROTEIN.		X	
IPI00253281	EPS8L1	ISOFORM 2 OF EPIDERMAL GROWTH FACTOR RECEPTOR KINASE SUBSTRATE 8-LIKE PROTEIN 1.		X	
IPI00255145	LOC342346	HYPOTHETICAL PROTEIN.		X	
IPI00257508	DPYSL2	DIHYDROPYRIMIDINASE-RELATED PROTEIN 2.		X	
IPI00260755	C20orf95	SIMILAR TO RHO GTPASE ACTIVATING PROTEIN 18.		X	
IPI00289837	CCDC85A	COILED-COIL DOMAIN-CONTAINING PROTEIN 85A.		X	
IPI00289862	SCRN1	SECERNIN-1.		X	
IPI00289965	KCNC3	POTASSIUM VOLTAGE-GATED CHANNEL SUBFAMILY C MEMBER 3.		X	
IPI00290094	SFRS8	SPLICING FACTOR, ARGININE/SERINE-RICH 8.		X	
IPI00290292	RHBDF1	RHOMBOID 5 HOMOLOG 1.		X	
IPI00290308	TRIB1	TRIBBLES HOMOLOG 1.		X	
IPI00290854	AKAP3	A-KINASE ANCHOR PROTEIN 3.		X	
IPI00291463	RSAD2	RADICAL S-ADENOSYL METHIONINE DOMAIN-CONTAINING PROTEIN 2.		X	
IPI00291922	PSMA5	PROTEASOME SUBUNIT ALPHA TYPE-5.		X	
IPI00291939	SMC1A	STRUCTURAL MAINTENANCE OF CHROMOSOMES PROTEIN 1A.		X	
IPI00292393	SCN4A	SODIUM CHANNEL PROTEIN TYPE 4 SUBUNIT ALPHA.		X	
IPI00293095	CCDC83	ISOFORM 1 OF COILED-COIL DOMAIN-CONTAINING PROTEIN 83.		X	
IPI00293361	SGSM2	ISOFORM 2 OF SMALL G PROTEIN SIGNALING MODULATOR 2.		X	
IPI00293396	AP1G1	ADAPTOR-RELATED PROTEIN COMPLEX 1, GAMMA 1 SUBUNIT ISOFORM A.		X	
IPI00293460	ABCA1	ATP-BINDING CASSETTE SUB-FAMILY A MEMBER 1.		X	
IPI00294210	ID2	DNA-BINDING PROTEIN INHIBITOR ID-2.		X	
IPI00294215	KIAA0232	UNCHARACTERIZED PROTEIN KIAA0232.		X	
IPI00294519	TEP1	ISOFORM 1 OF TELOMERASE PROTEIN COMPONENT 1.		X	

Table S1. Proteins Identified or Not in MS Patients Compared to Normals and Other Neurologic Disease (OND)					
**IPI**	**Gene**	**Protein Name**	**MS**	**Normal**	**OND**
IPI00294619	TFG	PROTEIN TFG.		X	
IPI00294910	DKFZP564O0823	PROTEIN PARM-1 PRECURSOR.		X	
IPI00295098	SRPRB	SIGNAL RECOGNITION PARTICLE RECEPTOR SUBUNIT BETA.		X	
IPI00295469	CPNE6	COPINE-6.		X	
IPI00295502	WIZ	ISOFORM 1 OF PROTEIN WIZ.		X	
IPI00295503	DDX58	ISOFORM 2 OF PROBABLE ATP-DEPENDENT RNA HELICASE DDX58.		X	
IPI00296219	GLS2	GLUTAMINASE LIVER ISOFORM, MITOCHONDRIAL PRECURSOR.		X	
IPI00296727	KIF2B	KINESIN-LIKE PROTEIN KIF2B.		X	
IPI00296866	IMPG2	INTERPHOTORECEPTOR MATRIX PROTEOGLYCAN 2.		X	
IPI00296913	NUDT5	ADP-SUGAR PYROPHOSPHATASE.		X	
IPI00298337	SLC14A1	CDNA FLJ77671.		X	
IPI00298476	GREM1	ISOFORM 1 OF GREMLIN-1 PRECURSOR.		X	
IPI00298738	POLRMT	DNA-DIRECTED RNA POLYMERASE, MITOCHONDRIAL PRECURSOR.		X	
IPI00299076	EBAG9	RECEPTOR-BINDING CANCER ANTIGEN EXPRESSED ON SISO CELLS (FRAGMENT).		X	
IPI00299116	PODXL	PODOCALYXIN-LIKE PROTEIN 1 PRECURSOR.		X	
IPI00299263	ARFGAP3	ADP-RIBOSYLATION FACTOR GTPASE-ACTIVATING PROTEIN 3.		X	
IPI00299679	RGL1	ISOFORM B OF RAL GUANINE NUCLEOTIDE DISSOCIATION STIMULATOR-LIKE 1.		X	
IPI00300244	ZCWPW1	ZINC FINGER, CW TYPE WITH PWWP DOMAIN 1.		X	
IPI00300623	PMCH	PRO-MCH PRECURSOR.		X	
IPI00300990	C1orf77	ISOFORM 1 OF UNCHARACTERIZED PROTEIN C1ORF77.		X	
IPI00301058	VASP	VASODILATOR-STIMULATED PHOSPHOPROTEIN.		X	
IPI00301923	CDK9	ISOFORM 1 OF CELL DIVISION PROTEIN KINASE 9.		X	
IPI00302850	SNRPD1	SMALL NUCLEAR RIBONUCLEOPROTEIN SM D1.		X	
IPI00304527	FAM83B	PROTEIN FAM83B.		X	
IPI00305833	SMU1	SMU-1 SUPPRESSOR OF MEC-8 AND UNC-52 PROTEIN HOMOLOG.		X	
IPI00306332	RPL24	60S RIBOSOMAL PROTEIN L24.		X	
IPI00306413	TPPP3	TUBULIN POLYMERIZATION-PROMOTING PROTEIN FAMILY MEMBER 3.		X	
IPI00306549	MLSTD1	CDNA FLJ11065 FIS, CLONE PLACE1004868, WEAKLY SIMILAR TO MALE STERILITY PROTEIN 2.		X	
IPI00306576	ARSB	ARYLSULFATASE B PRECURSOR.		X	
IPI00307702		H53_GS1 (FRAGMENT).		X	
IPI00328260	NSMAF	PROTEIN FAN.		X	
IPI00328270	NPAS2	NEURONAL PAS DOMAIN-CONTAINING PROTEIN 2.		X	

Table S1. Proteins Identified or Not in MS Patients Compared to Normals and Other Neurologic Disease (OND)					
**IPI**	**Gene**	**Protein Name**	**MS**	**Normal**	**OND**
IPI00328298	SMC4	ISOFORM 2 OF STRUCTURAL MAINTENANCE OF CHROMOSOMES PROTEIN 4.		X	
IPI00328522	KTELC1	KTEL MOTIF-CONTAINING PROTEIN 1.		X	
IPI00329791	DDX46	CDNA FLJ78679, HIGHLY SIMILAR TO HOMO SAPIENS DEAD (ASP-GLU-ALA-ASP) BOX POLYPEPTIDE 46 (DDX46), MRNA.		X	
IPI00333410	UBE2Q1	ISOFORM 1 OF UBIQUITIN-CONJUGATING ENZYME E2 Q1.		X	
IPI00335437	ANKZF1	ANKYRIN REPEAT AND ZINC FINGER DOMAIN-CONTAINING PROTEIN 1.		X	
IPI00335541	TIMELESS	ISOFORM 1 OF PROTEIN TIMELESS HOMOLOG.		X	
IPI00335589	RNMTL1	RNA METHYLTRANSFERASE-LIKE PROTEIN 1.		X	
IPI00373823	CYP26C1	CYTOCHROME P450 26C1.		X	
IPI00373872	PKD1L2	POLYCYSTIN 1-LIKE 2 ISOFORM A.		X	
IPI00374039	C1orf189	CONSERVED HYPOTHETICAL PROTEIN.		X	
IPI00374129	NLRP3	NLR FAMILY, PYRIN DOMAIN CONTAINING 3 ISOFORM B.		X	
IPI00374301		HYPOTHETICAL PROTEIN.		X	
IPI00374670	LOC388588	HYPOTHETICAL PROTEIN ISOFORM 2.		X	
IPI00375174	ANKS1B	ANKYRIN REPEAT AND STERILE ALPHA MOTIF DOMAIN-CONTAINING PROTEIN 1B.		X	
IPI00375803	GON4L	ISOFORM 1 OF GON-4-LIKE PROTEIN.		X	
IPI00376087	7A5	PUTATIVE BINDING PROTEIN 7A5.		X	
IPI00376237	LBX2	ISOFORM 2 OF TRANSCRIPTION FACTOR LBX2.		X	
IPI00376587	LOC728780	UNCHARACTERIZED PROTEIN ENSP00000345065.		X	
IPI00382515		CDNA FLJ30384 FIS, CLONE BRACE2008114.		X	
IPI00382756	PLRG1	ISOFORM 2 OF PLEIOTROPIC REGULATOR 1.		X	
IPI00383832	DVL3	PROTEIN KINASE C-BINDING PROTEIN RACK8.		X	
IPI00384861	GIT1	ISOFORM 1 OF ARF GTPASE-ACTIVATING PROTEIN GIT1.		X	
IPI00385003	TIGD1	PUTATIVE TRANSPOSASE.		X	
IPI00385042	GTPBP4	NUCLEOLAR GTP-BINDING PROTEIN 1.		X	
IPI00385480	CASKIN1	CASKIN-1.		X	
IPI00385543	KIAA0907	ISOFORM 3 OF UPF0469 PROTEIN KIAA0907.		X	
IPI00385791	MRPS26	SEROLOGICALLY DEFINED BREAST CANCER ANTIGEN NY-BR-87 (FRAGMENT).		X	
IPI00385918	ZNF627	CDNA FLJ90582 FIS, CLONE PLACE1000442, MODERATELY SIMILAR TO ZINC FINGER PROTEIN ZFP-36.		X	
IPI00386284	OR2AK2	OLFACTORY RECEPTOR, FAMILY 2, SUBFAMILY AK, MEMBER 2.		X	
IPI00386576		IG LAMBDA CHAIN V-IV REGION MOL.		X	
IPI00387004	MCM7	PNAS-146.		X	
IPI00387159	ING3	ISOFORM 1 OF INHIBITOR OF GROWTH PROTEIN 3.		X	

Table S1. Proteins Identified or Not in MS Patients Compared to Normals and Other Neurologic Disease (OND)					
**IPI**	**Gene**	**Protein Name**	**MS**	**Normal**	**OND**
IPI00394879	LRRC9	LEUCINE-RICH REPEAT-CONTAINING PROTEIN 9.		X	
IPI00396961	LRFN5	LEUCINE-RICH REPEAT AND FIBRONECTIN TYPE-III DOMAIN-CONTAINING PROTEIN 5 PRECURSOR.		X	
IPI00397578	PPFIA4	135 KDA PROTEIN.		X	
IPI00398154	AFAP1	ACTIN FILAMENT ASSOCIATED PROTEIN 1.		X	
IPI00398229	LOC392197	SIMILAR TO DEUBIQUITINATING ENZYME 3.		X	
IPI00398992	CHD8	ISOFORM 1 OF CHROMODOMAIN-HELICASE-DNA-BINDING PROTEIN 8.		X	
IPI00399180	SBK1	SERINE/THREONINE-PROTEIN KINASE SBK1.		X	
IPI00399252	PHF17	ISOFORM 1 OF PROTEIN JADE-1.		X	
IPI00399254	OTUD4	ISOFORM 1 OF OTU DOMAIN-CONTAINING PROTEIN 4.		X	
IPI00399296	LOC390110	HYPOTHETICAL PROTEIN LOC390110.		X	
IPI00399328	LOC390245	SIMILAR TO JUMONJI DOMAIN CONTAINING 2D.		X	
IPI00401852	DKFZP434L187	CONSERVED HYPOTHETICAL PROTEIN.		X	
IPI00402144	ZNF555	ISOFORM 1 OF ZINC FINGER PROTEIN 555.		X	
IPI00410093	CCDC69	COILED-COIL DOMAIN CONTAINING 69.		X	
IPI00410657	RNMT	ISOFORM 2 OF MRNA CAP GUANINE-N7 METHYLTRANSFERASE.		X	
IPI00411656	PCLO	ISOFORM 1 OF PROTEIN PICCOLO.		X	
IPI00411674	ZNF254	ISOFORM 1 OF ZINC FINGER PROTEIN 254.		X	
IPI00412216	VPS13C	VACUOLAR PROTEIN SORTING 13C PROTEIN ISOFORM 2B.		X	
IPI00412408	BRCA2	BREAST CANCER TYPE 2 SUSCEPTIBILITY PROTEIN.		X	
IPI00414320	ANXA11	ANNEXIN A11.		X	
IPI00414481	GTF3C1	GTF3C1 PROTEIN.		X	
IPI00418735	LOC400566	HYPOTHETICAL PROTEIN LOC400566.		X	
IPI00419221	MBOAT2	MEMBRANE-BOUND O-ACYLTRANSFERASE DOMAIN-CONTAINING PROTEIN 2.		X	
IPI00419237	LAP3	ISOFORM 1 OF CYTOSOL AMINOPEPTIDASE.		X	
IPI00419253	NAP5	ISOFORM 1 OF NCK-ASSOCIATED PROTEIN 5.		X	
IPI00420014	ASCC3L1	ISOFORM 1 OF U5 SMALL NUCLEAR RIBONUCLEOPROTEIN 200 KDA HELICASE.		X	
IPI00423683	EMID1	ISOFORM 2 OF EMI DOMAIN-CONTAINING PROTEIN 1 PRECURSOR.		X	
IPI00426727	MBD4	ISOFORM 1 OF METHYL-CPG-BINDING DOMAIN PROTEIN 4.		X	
IPI00428741	FLJ35348	LP2477.		X	
IPI00432226		AVLL5809.		X	
IPI00432755	FAM124A	PPRR6495.		X	
IPI00434711		PUTATIVE UNCHARACTERIZED PROTEIN FP6679.		X	

Table S1. Proteins Identified or Not in MS Patients Compared to Normals and Other Neurologic Disease (OND)					
**IPI**	**Gene**	**Protein Name**	**MS**	**Normal**	**OND**
IPI00435925	IGFBP3	PP14214.		X	
IPI00438170	SNX12	ISOFORM 1 OF SORTING NEXIN-12.		X	
IPI00440221	CDC2L5	PUTATIVE UNCHARACTERIZED PROTEIN (FRAGMENT).		X	
IPI00441344	GLB1	BETA-GALACTOSIDASE PRECURSOR.		X	
IPI00442121	ALAD	DELTA-AMINOLEVULINIC ACID DEHYDRATASE ISOFORM A.		X	
IPI00442544		CDNA FLJ27034 FIS, CLONE SLV07984.		X	
IPI00442564		CDNA FLJ26948 FIS, CLONE RCT08241.		X	
IPI00442745		CDNA FLJ26780 FIS, CLONE PRS03837.		X	
IPI00442865	SLC12A1	CDNA FLJ26488 FIS, CLONE KDN05770, HIGHLY SIMILAR TO BUMETANIDE- SENSITIVE SODIUM-(POTASSIUM)-CHLORIDE COTRANSPORTER 2.		X	
IPI00444331	WHSC1L1	ISOFORM 4 OF HISTONE-LYSINE N-METHYLTRANSFERASE NSD3.		X	
IPI00444842		CDNA FLJ45125 FIS, CLONE BRAWH3036561.		X	
IPI00445278		CDNA FLJ44033 FIS, CLONE TESTI4028062.		X	
IPI00445315	FAM47C	PROTEIN FAM47C.		X	
IPI00445364		CDNA FLJ44171 FIS, CLONE THYMU2036058.		X	
IPI00448465	ANKRD12	ISOFORM 1 OF ANKYRIN REPEAT DOMAIN-CONTAINING PROTEIN 12.		X	
IPI00454695	HIST2H2BC	HISTONE H2B TYPE 2-C.		X	
IPI00454858	LOC344875	SIMILAR TO ALPHA 3 TYPE VI COLLAGEN ISOFORM 1 PRECURSOR.		X	
IPI00455852	ARHGEF15	ISOFORM 1 OF RHO GUANINE NUCLEOTIDE EXCHANGE FACTOR 15.		X	
IPI00456578	LOC441054	LOC441054 PROTEIN.		X	
IPI00456599	MGC12966	HYPOTHETICAL PROTEIN LOC84792.		X	
IPI00456670	PAOX	ISOFORM 13 OF PEROXISOMAL N(1)-ACETYL-SPERMINE/SPERMIDINE OXIDASE.		X	
IPI00456827	FAM22G	PROTEIN FAM22G PRECURSOR.		X	
IPI00465044	RCC2	PROTEIN RCC2.		X	
IPI00465045	DIP2B	DIP2 DISCO-INTERACTING PROTEIN 2 HOMOLOG B.		X	
IPI00465123	KIAA0415	KIAA0415 GENE PRODUCT.		X	
IPI00465178	ATP6V0A1	ISOFORM 1 OF VACUOLAR PROTON TRANSLOCATING ATPASE 116 KDA SUBUNIT A ISOFORM 1.		X	
IPI00465234	CSF2RB	CYTOKINE RECEPTOR COMMON BETA CHAIN PRECURSOR.		X	
IPI00470468	EFR3A	ISOFORM 3 OF PROTEIN EFR3 HOMOLOG A.		X	
IPI00470913	RGPD1; RGPD2	RANBP2-LIKE AND GRIP DOMAIN CONTAINING 1.		X	
IPI00472754	KIAA0319L	POLYCYSTIC KIDNEY DISEASE 1-RELATED PROTEIN.		X	
IPI00477361	SDHALP1	10 KDA PROTEIN.		X	

Table S1. Proteins Identified or Not in MS Patients Compared to Normals and Other Neurologic Disease (OND)					
**IPI**	**Gene**	**Protein Name**	**MS**	**Normal**	**OND**
IPI00477468	CTR9	RNA POLYMERASE-ASSOCIATED PROTEIN CTR9 HOMOLOG.		X	
IPI00478124	UCKL1	61 KDA PROTEIN.		X	
IPI00478521	RILPL1	ISOFORM 1 OF UPF0475 PROTEIN.		X	
IPI00478640	C17orf87	ISOFORM 1 OF TRANSMEMBRANE PROTEIN C17ORF87.		X	
IPI00478986	LOC126235	SIMILAR TO 40S RIBOSOMAL PROTEIN S4.		X	
IPI00479125	SRGAP2	SLIT-ROBO RHO GTPASE-ACTIVATING PROTEIN 2.		X	
IPI00479361	B3GNT4	ISOFORM 1 OF UDP-GLCNAC:BETAGAL BETA-1,3-N-ACETYLGLUCOSAMINYLTRANSFERASE 4.		X	
IPI00479669	UHRF1BP1L	ISOFORM 1 OF UNCHARACTERIZED PROTEIN KIAA0701.		X	
IPI00479722	PSME1	PROTEASOME ACTIVATOR COMPLEX SUBUNIT 1.		X	
IPI00480159	INPP5F	INOSITOL POLYPHOSPHATE-5-PHOSPHATASE F.		X	
IPI00514594	FAM5B	ISOFORM 1 OF PROTEIN FAM5B PRECURSOR.		X	
IPI00514622	RANBP6	RAN-BINDING PROTEIN 6.		X	
IPI00549972	LIMD2	LIM DOMAIN-CONTAINING PROTEIN 2.		X	
IPI00550364	PGM2	PHOSPHOGLUCOMUTASE-2.		X	
IPI00550720	C19orf57	ISOFORM 1 OF UNCHARACTERIZED PROTEIN C19ORF57.		X	
IPI00550746	NUDC	NUCLEAR MIGRATION PROTEIN NUDC.		X	
IPI00550876	MRO	PROTEIN MAESTRO.		X	
IPI00550906	CSTF2T	CLEAVAGE STIMULATION FACTOR 64 KDA SUBUNIT, TAU VARIANT.		X	
IPI00550917	TWF2	TWINFILIN-2.		X	
IPI00552591	IGLV10-54	V1-20 PROTEIN.		X	
IPI00554752	PRKAR2B	CAMP-DEPENDENT PROTEIN KINASE TYPE II-BETA REGULATORY SUBUNIT.		X	
IPI00555600	IDUA	SOLUTE CARRIER FAMILY 26, MEMBER 1 ISOFORM A VARIANT (FRAGMENT).		X	
IPI00604430	REEP2	ISOFORM 2 OF RECEPTOR EXPRESSION-ENHANCING PROTEIN 2.		X	
IPI00604551	CDCA7	ISOFORM 1 OF CELL DIVISION CYCLE-ASSOCIATED PROTEIN 7.		X	
IPI00604763	TMEM66	TRANSMEMBRANE PROTEIN 66 PRECURSOR.		X	
IPI00607831	PRAMEF3	PRAME FAMILY MEMBER 3.		X	
IPI00640810	CTDP1	6 KDA PROTEIN.		X	
IPI00642645	MTHFR	METHYLENETETRAHYDROFOLATE REDUCTASE.		X	
IPI00643937	MTHFD1L	METHYLENETETRAHYDROFOLATE DEHYDROGENASE (NADP+ DEPENDENT) 1-LIKE.		X	
IPI00644231	CYFIP1	ISOFORM 1 OF CYTOPLASMIC FMR1-INTERACTING PROTEIN 1.		X	
IPI00644522	PNKP	PNKP PROTEIN.		X	
IPI00644766	TOR1AIP1	CDNA FLJ78048, HIGHLY SIMILAR TO HOMO SAPIENS TORSIN A INTERACTING PROTEIN 1, MRNA.		X	

Table S1. Proteins Identified or Not in MS Patients Compared to Normals and Other Neurologic Disease (OND)					
**IPI**	**Gene**	**Protein Name**	**MS**	**Normal**	**OND**
IPI00645089	KCNIP1	KV CHANNEL INTERACTING PROTEIN 1 ISOFORM 3.		X	
IPI00645814	MAP7D1	ISOFORM 2 OF MAP7 DOMAIN-CONTAINING PROTEIN 1.		X	
IPI00657699	MUC19	PROTEIN.		X	
IPI00658025		PUTATIVE NOVEL TRANSCRIPT.		X	
IPI00658112	SPEG	32 KDA PROTEIN.		X	
IPI00718821	C19orf55	ISOFORM 1 OF UNCHARACTERIZED PROTEIN C19ORF55.		X	
IPI00719505	RABL2A	RABL2A PROTEIN.		X	
IPI00735934	LOC646070	SIMILAR TO CAPICUA HOMOLOG.		X	
IPI00738920	LOC642574	SIMILAR TO CG3104-PA, ISOFORM A.		X	
IPI00739106	LOC647436	SIMILAR TO RIBOSOMAL PROTEIN L5 ISOFORM 1.		X	
IPI00740191	LOC651986	SIMILAR TO FORKHEAD BOX PROTEIN L1.		X	
IPI00741780	LOC652559	SIMILAR TO CG4845-PA.		X	
IPI00742725	LOC388564	CONSERVED HYPOTHETICAL PROTEIN.		X	
IPI00744226		CONSERVED HYPOTHETICAL PROTEIN.		X	
IPI00744366		CONSERVED HYPOTHETICAL PROTEIN.		X	
IPI00744811	LRP5	LOW-DENSITY LIPOPROTEIN RECEPTOR-RELATED PROTEIN 5 PRECURSOR.		X	
IPI00744825		CONSERVED HYPOTHETICAL PROTEIN.		X	
IPI00745122	MGC33894	CONSERVED HYPOTHETICAL PROTEIN.		X	
IPI00745300	NAT11	31 KDA PROTEIN.		X	
IPI00746666	LOC728262	HYPOTHETICAL PROTEIN.		X	
IPI00746681		SIMILAR TO BCL-2-RELATED OVARIAN KILLER PROTEIN.		X	
IPI00746987		RIBOSOMAL PROTEIN S1 FAMILY PROTEIN.		X	
IPI00747142	CTGLF6	CENTAURIN-GAMMA-LIKE FAMILY MEMBER 6.		X	
IPI00747420		MELANOMA-DERIVED PROTEIN (FRAGMENT).		X	
IPI00747494	GRID2	GLUTAMATE RECEPTOR DELTA-2 SUBUNIT PRECURSOR.		X	
IPI00747657	PDE6B	SIMILAR TO ROD CGMP-SPECIFIC 3',5'-CYCLIC PHOSPHODIESTERASE SUBUNIT BETA PRECURSOR.		X	
IPI00748682		PHEROMONE SHUTDOWN-RELATED, TRAB FAMILY PROTEIN.		X	
IPI00748891	C14orf24	HYPOTHETICAL PROTEIN LOC283635 ISOFORM 1.		X	
IPI00749171	LOC340184	CONSERVED HYPOTHETICAL PROTEIN.		X	
IPI00749514	ATRNL1	ATTRACTIN-LIKE 1.		X	
IPI00783753	C15orf40	UPF0235 PROTEIN C15ORF40.		X	
IPI00783855	NBR1	NEIGHBOR OF BRCA1 GENE 1.		X	
IPI00784739	C14orf43	UNCHARACTERIZED PROTEIN C14ORF43.		X	

Table S1. Proteins Identified or Not in MS Patients Compared to Normals and Other Neurologic Disease (OND)					
**IPI**	**Gene**	**Protein Name**	**MS**	**Normal**	**OND**
IPI00784880	LOC440934	CANCER/TESTIS ANTIGEN 75.		X	
IPI00785015	FLJ25778	ISOFORM 1 OF UNCHARACTERIZED PROTEIN KIAA2030.		X	
IPI00786937	LOC731940	SIMILAR TO DELETED IN MALIGNANT BRAIN TUMORS 1 ISOFORM B PRECURSOR.		X	
IPI00786946	LOC642249	SIMILAR TO TEKTIN-3.		X	
IPI00787020	LOC644153	SIMILAR TO DYNAMIN-1.		X	
IPI00787083	LOC256374	SIMILAR TO PEPTIDYLPROLYL ISOMERASE A ISOFORM 1.		X	
IPI00787414	MGC34829	UNCHARACTERIZED PROTEIN ENSP00000381388.		X	
IPI00787932	hCG_1646157	SIMILAR TO ZINC FINGER PROTEIN 10.		X	
IPI00788258	LOXL1	SIMILAR TO LYSYL OXIDASE-LIKE 1 PREPROPROTEIN.		X	
IPI00789181	PLCL1	115 KDA PROTEIN.		X	
IPI00789245	SLC22A23	ISOFORM 2 OF PROBABLE ORGANIC CATION TRANSPORTER PROTEIN C6ORF85.		X	
IPI00790021	ZNF652	ZINC FINGER PROTEIN 652.		X	
IPI00791513	CTA-216E10.6	CDNA FLJ16614 FIS, CLONE TESTI4013365.		X	
IPI00791593	GLYCAM1	8 KDA PROTEIN.		X	
IPI00792229	TATDN1	20 KDA PROTEIN.		X	
IPI00792945	CHFR	38 KDA PROTEIN.		X	
IPI00794119	ABCC8	13 KDA PROTEIN.		X	
IPI00796647	HIGD1C	HIG1 DOMAIN FAMILY, MEMBER 1C.		X	
IPI00796777	CRYAA	17 KDA PROTEIN.		X	
IPI00796906	ABCF3	8 KDA PROTEIN.		X	
IPI00797694		3 KDA PROTEIN.		X	
IPI00797699		20 KDA PROTEIN.		X	
IPI00807418	HELLS	ISOFORM 9 OF LYMPHOID-SPECIFIC HELICASE.		X	
IPI00815786	HK1	HEXOKINASE 1 (FRAGMENT).		X	
IPI00815893	CHD2	ISOFORM 1 OF CHROMODOMAIN-HELICASE-DNA-BINDING PROTEIN 2.		X	
IPI00816274	CMTM1	CHEMOKINE-LIKE FACTOR SUPERFAMILY 1 TRANSCRIPT VARIANT 26.		X	
IPI00827584	KIF27	SIMILAR TO KINESIN FAMILY MEMBER 27.		X	
IPI00827745	RBM24	ISOFORM 1 OF RNA-BINDING PROTEIN 24.		X	
IPI00830051	LOC90925	SIMILAR TO IMMUNOLGOOBULIN HEAVY CHAIN.		X	
IPI00843819		SIMILAR TO DUAL SPECIFICITY PROTEIN KINASE CLK2.		X	
IPI00845508	BAHCC1	BAH DOMAIN AND COILED-COIL CONTAINING 1.		X	
IPI00847335		FLJ45422 PROTEIN.		X	
IPI00847759	DENND4B	DENN DOMAIN-CONTAINING PROTEIN 4B.		X	
IPI00852633	RABL2B	16 KDA PROTEIN.		X	

Table S1. Proteins Identified or Not in MS Patients Compared to Normals and Other Neurologic Disease (OND)					
**IPI**	**Gene**	**Protein Name**	**MS**	**Normal**	**OND**
IPI00852725	PRLR	ISOFORM 7 OF PROLACTIN RECEPTOR PRECURSOR.		X	
IPI00852758	ANKRD18B	SIMILAR TO ANKYRIN REPEAT DOMAIN-CONTAINING PROTEIN 26. ISOFORM 2.		X	
IPI00853312		UNCHARACTERIZED PROTEIN ENSP00000324580.		X	
IPI00853376	ASXL3	ADDITIONAL SEX COMBS LIKE 3.		X	
IPI00855918	MUC5B	MUCIN 5, SUBTYPE B, TRACHEOBRONCHIAL.		X	
IPI00871533	C3orf48	UNCHARACTERIZED PROTEIN C3ORF48 (FRAGMENT).		X	
IPI00871556	ZFYVE28	107 KDA PROTEIN.		X	
IPI00872550	PRDM2	UNCHARACTERIZED PROTEIN PRDM2.		X	
IPI00872739	C18orf2	UNCHARACTERIZED PROTEIN C18ORF2.		X	
IPI00872861	LOC51136	PTD016 PROTEIN.		X	
IPI00873740		UNCHARACTERIZED PROTEIN ENSP00000383832 (FRAGMENT).		X	
IPI00873774		UNCHARACTERIZED PROTEIN ENSP00000383488 (FRAGMENT).		X	
IPI00874023		UNCHARACTERIZED PROTEIN ENSP00000379699.		X	
IPI00877084	CCDC144C	ISOFORM 1 OF COILED-COIL DOMAIN-CONTAINING PROTEIN 144C.		X	
IPI00877615		15 KDA PROTEIN.		X	
IPI00878436	SLC2A11	24 KDA PROTEIN.		X	
IPI00878511		45 KDA PROTEIN.		X	
IPI00878755		43 KDA PROTEIN.		X	
IPI00878962		10 KDA PROTEIN.		X	
IPI00879409		28 KDA PROTEIN.		X	
IPI00879842		6 KDA PROTEIN.		X	
IPI00879950		15 KDA PROTEIN.		X	
IPI00884353		ETS-1 TRANSCRIPT VARIANT ETS-1 DELTA.		X	
IPI00000030	PPP2R5D	ISOFORM DELTA-1 OF SERINE/THREONINE-PROTEIN PHOSPHATASE 2A 56 KDA REGULATORY SUBUNIT DELTA ISOFORM.			X
IPI00000057	COG2	CONSERVED OLIGOMERIC GOLGI COMPLEX COMPONENT 2.			X
IPI00000102	GNRH1	PROGONADOLIBERIN-1 PRECURSOR.			X
IPI00000106	STAT4	SIGNAL TRANSDUCER AND ACTIVATOR OF TRANSCRIPTION 4.			X
IPI00000110	ZNF337	ZINC FINGER PROTEIN 337.			X
IPI00000270	RNASE7	RIBONUCLEASE 7 PRECURSOR.			X
IPI00000388	ZNF175	ZINC FINGER PROTEIN 175.			X
IPI00000656	KIAA0892	ISOFORM 1 OF UNCHARACTERIZED PROTEIN KIAA0892 PRECURSOR.			X
IPI00000663	MLYCD	ISOFORM MITOCHONDRIAL OF MALONYL-COA DECARBOXYLASE, MITOCHONDRIAL PRECURSOR.			X

Table S1. Proteins Identified or Not in MS Patients Compared to Normals and Other Neurologic Disease (OND)					
**IPI**	**Gene**	**Protein Name**	**MS**	**Normal**	**OND**
IPI00000690	AIFM1	ISOFORM 1 OF APOPTOSIS-INDUCING FACTOR 1, MITOCHONDRIAL PRECURSOR.			X
IPI00000787	PSMB9	ISOFORM LMP2.L OF PROTEASOME SUBUNIT BETA TYPE-9 PRECURSOR.			X
IPI00000837	GRM3	METABOTROPIC GLUTAMATE RECEPTOR 3 PRECURSOR.			X
IPI00000861	LASP1	ISOFORM 1 OF LIM AND SH3 DOMAIN PROTEIN 1.			X
IPI00000875	EEF1G	ELONGATION FACTOR 1-GAMMA.			X
IPI00000890	GH2	ISOFORM 1 OF GROWTH HORMONE VARIANT PRECURSOR.			X
IPI00001141	TIMM22	MITOCHONDRIAL IMPORT INNER MEMBRANE TRANSLOCASE SUBUNIT TIM22.			X
IPI00001151	C20orf59	ISOFORM 2 OF UNCHARACTERIZED MFS-TYPE TRANSPORTER C20ORF59.			X
IPI00001317	RHOBTB1	RHO-RELATED BTB DOMAIN-CONTAINING PROTEIN 1.			X
IPI00001516	PCDHAC1; PCDHAC2	ISOFORM LONG OF PROTOCADHERIN ALPHA C2 PRECURSOR.			X
IPI00001580	FYCO1	ISOFORM 1 OF FYVE AND COILED-COIL DOMAIN-CONTAINING PROTEIN 1.			X
IPI00001641	ORC6L	ORIGIN RECOGNITION COMPLEX SUBUNIT 6.			X
IPI00001654	PCM1	ISOFORM 3 OF PERICENTRIOLAR MATERIAL 1 PROTEIN.			X
IPI00001674	TAC3	ISOFORM 1 OF NEUROKININ-B PRECURSOR.			X
IPI00001753	MYH4	MYOSIN-4.			X
IPI00001780	USP16	UBIQUITIN CARBOXYL-TERMINAL HYDROLASE 16.			X
IPI00001922	ST14	SUPPRESSOR OF TUMORIGENICITY PROTEIN 14.			X
IPI00001933	CCDC113	COILED-COIL DOMAIN-CONTAINING PROTEIN 113.			X
IPI00002134	PSMD5	26S PROTEASOME NON-ATPASE REGULATORY SUBUNIT 5.			X
IPI00002344	SRD5A3	STEROID 5 ALPHA-REDUCTASE 3.			X
IPI00002441	SDC1	SYNDECAN-1 PRECURSOR.			X
IPI00002446	PDE4A	PHOSPHODIESTERASE.			X
IPI00002506	ALG5	DOLICHYL-PHOSPHATE BETA-GLUCOSYLTRANSFERASE.			X
IPI00002524	KCNJ14	ATP-SENSITIVE INWARD RECTIFIER POTASSIUM CHANNEL 14.			X
IPI00002534	PURG	ISOFORM 1 OF PURINE-RICH ELEMENT-BINDING PROTEIN GAMMA.			X
IPI00002649	PNN	ISOFORM 2 OF PININ.			X
IPI00002707	SPAST	ISOFORM 1 OF SPASTIN.			X
IPI00002773	JAK3	ISOFORM 2 OF TYROSINE-PROTEIN KINASE JAK3.			X
IPI00002824	CSRP2	CYSTEINE AND GLYCINE-RICH PROTEIN 2.			X
IPI00002841	SLC9A2	SODIUM/HYDROGEN EXCHANGER 2.			X
IPI00002857	MAPK14	ISOFORM CSBP2 OF MITOGEN-ACTIVATED PROTEIN KINASE 14.			X
IPI00002881	BCL2A1	BCL-2-RELATED PROTEIN A1.			X
IPI00002897	GABRA3	GAMMA-AMINOBUTYRIC ACID RECEPTOR SUBUNIT ALPHA-3 PRECURSOR.			X

Table S1. Proteins Identified or Not in MS Patients Compared to Normals and Other Neurologic Disease (OND)					
**IPI**	**Gene**	**Protein Name**	**MS**	**Normal**	**OND**
IPI00002970	SGCB	BETA-SARCOGLYCAN.			X
IPI00003053	ARMC9	CDNA: FLJ23510 FIS, CLONE LNG03216.			X
IPI00003084	DRAP1	ISOFORM 1 OF DR1-ASSOCIATED COREPRESSOR.			X
IPI00003377	SFRS7	ISOFORM 1 OF SPLICING FACTOR, ARGININE/SERINE-RICH 7.			X
IPI00003421	TBR1	T-BRAIN-1 PROTEIN.			X
IPI00003438	DNAJC8	DNAJ HOMOLOG SUBFAMILY C MEMBER 8.			X
IPI00003483	NEURL	ISOFORM 1 OF NEURALIZED-LIKE PROTEIN 1.			X
IPI00003571	CABP1	ISOFORM S-CABP1 OF CALCIUM-BINDING PROTEIN 1.			X
IPI00003768	PES1	ISOFORM 1 OF PESCADILLO HOMOLOG 1.			X
IPI00003842	MAP2	ISOFORM 1 OF MICROTUBULE-ASSOCIATED PROTEIN 2.			X
IPI00003881	HNRPF	HETEROGENEOUS NUCLEAR RIBONUCLEOPROTEIN F.			X
IPI00003893	PCDHGB3	ISOFORM 1 OF PROTOCADHERIN GAMMA B3 PRECURSOR.			X
IPI00003894	RNF11	RING FINGER PROTEIN 11.			X
IPI00003897	PCDHGB6	ISOFORM 1 OF PROTOCADHERIN GAMMA B6 PRECURSOR.			X
IPI00003949	UBE2N	UBIQUITIN-CONJUGATING ENZYME E2 N.			X
IPI00003990	BPHL	ISOFORM 2 OF VALACYCLOVIR HYDROLASE PRECURSOR.			X
IPI00004101	BHMT	BETAINE--HOMOCYSTEINE S-METHYLTRANSFERASE 1.			X
IPI00004288	SIGLEC7	ISOFORM 1 OF SIALIC ACID-BINDING IG-LIKE LECTIN 7 PRECURSOR.			X
IPI00004337	ZBTB11	ZINC FINGER AND BTB DOMAIN-CONTAINING PROTEIN 11.			X
IPI00004362	MORC1	MORC FAMILY CW-TYPE ZINC FINGER PROTEIN 1.			X
IPI00004416	CHMP2A	CHARGED MULTIVESICULAR BODY PROTEIN 2A.			X
IPI00004419	ZNF646	ZINC FINGER PROTEIN 646.			X
IPI00004445	SNTG2	GAMMA-2-SYNTROPHIN.			X
IPI00004446	SRPX2	SUSHI REPEAT-CONTAINING PROTEIN SRPX2 PRECURSOR.			X
IPI00004472	WNK1	ISOFORM 1 OF SERINE/THREONINE-PROTEIN KINASE WNK1.			X
IPI00004509	LTC4S	LEUKOTRIENE C4 SYNTHASE.			X
IPI00004527	KIAA0355	UNCHARACTERIZED PROTEIN KIAA0355.			X
IPI00004538	NACAD	SIMILAR TO NASCENT POLYPEPTIDE-ASSOCIATED COMPLEX ALPHA POLYPEPTIDE ISOFORM 1.			X
IPI00004563	FAM105A	PROTEIN FAM105A.			X
IPI00004569	MZF1	ISOFORM MZF1A OF MYELOID ZINC FINGER 1.			X
IPI00004671	GOLGB1	GOLGIN SUBFAMILY B MEMBER 1.			X
IPI00004838	CRK	ISOFORM CRK-II OF PROTO-ONCOGENE C-CRK.			X
IPI00004944	SLC4A10	ISOFORM 1 OF SODIUM-DRIVEN CHLORIDE BICARBONATE EXCHANGER.			X

Table S1. Proteins Identified or Not in MS Patients Compared to Normals and Other Neurologic Disease (OND)					
**IPI**	**Gene**	**Protein Name**	**MS**	**Normal**	**OND**
IPI00005013	PCBP4	POLY(RC) BINDING PROTEIN 4 ISOFORM B.			X
IPI00005024	MYBBP1A	ISOFORM 1 OF MYB-BINDING PROTEIN 1A.			X
IPI00005087	TMOD3	TROPOMODULIN-3.			X
IPI00005171	HLA-DRA	HLA CLASS II HISTOCOMPATIBILITY ANTIGEN, DR ALPHA CHAIN PRECURSOR.			X
IPI00005254	PARK2	ISOFORM 1 OF E3 UBIQUITIN-PROTEIN LIGASE PARKIN.			X
IPI00005530	NPVF	FMRFAMIDE-RELATED PEPTIDES PRECURSOR.			X
IPI00005537	MRPL12	39S RIBOSOMAL PROTEIN L12, MITOCHONDRIAL PRECURSOR.			X
IPI00005563	TINAGL1	ISOFORM 1 OF TUBULOINTERSTITIAL NEPHRITIS ANTIGEN-LIKE PRECURSOR.			X
IPI00005567	CACNB3	ISOFORM 3A OF VOLTAGE-DEPENDENT L-TYPE CALCIUM CHANNEL SUBUNIT BETA-3.			X
IPI00005616	TAAR2	ISOFORM 1 OF TRACE AMINE-ASSOCIATED RECEPTOR 2.			X
IPI00005621	SYDE1	7H3 PROTEIN (FRAGMENT).			X
IPI00005661	MLX	ISOFORM GAMMA OF MAX-LIKE PROTEIN X.			X
IPI00005708	HS3ST3B1	HEPARAN SULFATE GLUCOSAMINE 3-O-SULFOTRANSFERASE 3B1.			X
IPI00005710	HS3ST2	HEPARAN SULFATE GLUCOSAMINE 3-O-SULFOTRANSFERASE 2.			X
IPI00005711	HDAC6	HISTONE DEACETYLASE 6.			X
IPI00005721	DEFA1; LOC728358	NEUTROPHIL DEFENSIN 1 PRECURSOR.			X
IPI00005793	AP3B2	AP-3 COMPLEX SUBUNIT BETA-2.			X
IPI00005978	SFRS2	SPLICING FACTOR, ARGININE/SERINE-RICH 2.			X
IPI00006003	CD83	CD83 ANTIGEN PRECURSOR.			X
IPI00006030	DNAI1	DYNEIN INTERMEDIATE CHAIN 1, AXONEMAL.			X
IPI00006077	PHF16	PROTEIN JADE-3.			X
IPI00006152	EDG8	ISOFORM 1 OF SPHINGOSINE 1-PHOSPHATE RECEPTOR EDG-8.			X
IPI00006158	LRMP	LYMPHOID-RESTRICTED MEMBRANE PROTEIN.			X
IPI00006173	CETP	ISOFORM 1 OF CHOLESTERYL ESTER TRANSFER PROTEIN PRECURSOR.			X
IPI00006178	NPY6R	Y6 ENCODING PROTEIN.			X
IPI00006236	PDGFRL	PLATELET-DERIVED GROWTH FACTOR RECEPTOR-LIKE PROTEIN PRECURSOR.			X
IPI00006298	PPIG	ISOFORM 1 OF PEPTIDYL-PROLYL CIS-TRANS ISOMERASE G.			X
IPI00006356	PNPLA5	ISOFORM 1 OF PATATIN-LIKE PHOSPHOLIPASE DOMAIN-CONTAINING PROTEIN 5.			X
IPI00006543	CFHR5	COMPLEMENT FACTOR H-RELATED 5.			X
IPI00006561	KIAA0317	ISOFORM 1 OF PROTEIN KIAA0317.			X
IPI00006612	SNAP91	ISOFORM 1 OF CLATHRIN COAT ASSEMBLY PROTEIN AP180.			X
IPI00006631	SV2B	SYNAPTIC VESICLE GLYCOPROTEIN 2B.			X
IPI00006663	ALDH2	ALDEHYDE DEHYDROGENASE, MITOCHONDRIAL PRECURSOR.			X

Table S1. Proteins Identified or Not in MS Patients Compared to Normals and Other Neurologic Disease (OND)					
**IPI**	**Gene**	**Protein Name**	**MS**	**Normal**	**OND**
IPI00006675	ABCC4	MULTIDRUG RESISTANCE-ASSOCIATED PROTEIN 4.			X
IPI00006715	RAD21	DOUBLE-STRAND-BREAK REPAIR PROTEIN RAD21 HOMOLOG.			X
IPI00006800	ATG5	ISOFORM LONG OF AUTOPHAGY PROTEIN 5.			X
IPI00007057	ZFYVE20	RABENOSYN-5.			X
IPI00007067	C9orf19	GOLGI-ASSOCIATED PLANT PATHOGENESIS-RELATED PROTEIN 1.			X
IPI00007074	YARS	TYROSYL-TRNA SYNTHETASE, CYTOPLASMIC.			X
IPI00007207	LIPA	ISOFORM 1 OF LYSOSOMAL ACID LIPASE/CHOLESTERYL ESTER HYDROLASE PRECURSOR.			X
IPI00007208	DDX41	PROBABLE ATP-DEPENDENT RNA HELICASE DDX41.			X
IPI00007300	BTBD3	BTB/POZ DOMAIN-CONTAINING PROTEIN 3.			X
IPI00007327	TAPBP	ISOFORM 1 OF TAPASIN PRECURSOR.			X
IPI00007360	ZNF238	ZINC FINGER PROTEIN 238.			X
IPI00007423	ANP32B	ISOFORM 1 OF ACIDIC LEUCINE-RICH NUCLEAR PHOSPHOPROTEIN 32 FAMILY MEMBER B.			X
IPI00007444	EPYC	EPIPHYCAN PRECURSOR.			X
IPI00007461	DPY19L1	ISOFORM 1 OF PROTEIN DPY-19 HOMOLOG 1.			X
IPI00007648	SLC6A11	SODIUM- AND CHLORIDE-DEPENDENT GABA TRANSPORTER 3.			X
IPI00007757	IKZF2	ISOFORM 1 OF ZINC FINGER PROTEIN HELIOS.			X
IPI00007836	TRPC5	SHORT TRANSIENT RECEPTOR POTENTIAL CHANNEL 5.			X
IPI00007910	SLC34A2	ISOFORM 1 OF SODIUM-DEPENDENT PHOSPHATE TRANSPORT PROTEIN 2B.			X
IPI00007917	COLEC10	COLLECTIN-10 PRECURSOR.			X
IPI00008444	CDADC1	ISOFORM 1 OF CYTIDINE AND DCMP DEAMINASE DOMAIN-CONTAINING PROTEIN 1.			X
IPI00008449	FIP1L1	ISOFORM 3 OF PRE-MRNA 3'-END-PROCESSING FACTOR FIP1.			X
IPI00008475	HMGCS1	HYDROXYMETHYLGLUTARYL-COA SYNTHASE, CYTOPLASMIC.			X
IPI00008495	ND4	NADH-UBIQUINONE OXIDOREDUCTASE CHAIN 4.			X
IPI00008632	PHTF1	ISOFORM 1 OF PUTATIVE HOMEODOMAIN TRANSCRIPTION FACTOR 1.			X
IPI00008708	RSL1D1	RIBOSOMAL L1 DOMAIN-CONTAINING PROTEIN 1.			X
IPI00008753	MT1X	METALLOTHIONEIN-1X.			X
IPI00008816	REV1	ISOFORM 1 OF DNA REPAIR PROTEIN REV1.			X
IPI00008837	PTPN5	TYROSINE-PROTEIN PHOSPHATASE NON-RECEPTOR TYPE 5.			X
IPI00008868	MAP1B	MICROTUBULE-ASSOCIATED PROTEIN 1B.			X
IPI00009040	OAZ3	ORNITHINE DECARBOXYLASE ANTIZYME 3.			X
IPI00009043		APOPTOTIC-RELATED PROTEIN PCAR (FRAGMENT).			X

Table S1. Proteins Identified or Not in MS Patients Compared to Normals and Other Neurologic Disease (OND)					
IPI	Gene	Protein Name	MS	Normal	OND
IPI00009066	PTPRQ	SIMILAR TO PROTEIN TYROSINE PHOSPHATASE, RECEPTOR TYPE, Q ISOFORM 1 PRECURSOR.			X
IPI00009101	SRCAP	ISOFORM 2 OF HELICASE SRCAP.			X
IPI00009213	BCL11B	ISOFORM 1 OF B-CELL LYMPHOMA/LEUKEMIA 11B.			X
IPI00009315	ACBD3	GOLGI RESIDENT PROTEIN GCP60.			X
IPI00009333	TMEM9	TRANSMEMBRANE PROTEIN 9 PRECURSOR.			X
IPI00009375	HAAO	3-HYDROXYANTHRANILATE 3,4-DIOXYGENASE.			X
IPI00009504	SLC20A2	SOLUTE CARRIER FAMILY 20 MEMBER 2.			X
IPI00009524	TRIM10	ISOFORM ALPHA OF TRIPARTITE MOTIF-CONTAINING PROTEIN 10.			X
IPI00009645	RANBP17	RAN-BINDING PROTEIN 17.			X
IPI00009724	EFCAB6	ISOFORM 1 OF EF-HAND CALCIUM-BINDING DOMAIN-CONTAINING PROTEIN 6.			X
IPI00009804	SATB1	DNA-BINDING PROTEIN SATB1.			X
IPI00009851	PCDH12	PCDH12 PROTEIN.			X
IPI00009853	VN1R1	VOMERONASAL TYPE-1 RECEPTOR 1.			X
IPI00009891	TAF1	ISOFORM 1 OF TRANSCRIPTION INITIATION FACTOR TFIID SUBUNIT 1.			X
IPI00009982	TDRKH	ISOFORM 1 OF TUDOR AND KH DOMAIN-CONTAINING PROTEIN.			X
IPI00010085	PLAGL2	ZINC FINGER PROTEIN PLAGL2.			X
IPI00010130	GLUL	GLUTAMINE SYNTHETASE.			X
IPI00010142	UBE2G2	UBIQUITIN-CONJUGATING ENZYME E2 G2.			X
IPI00010157	MAT2A	S-ADENOSYLMETHIONINE SYNTHETASE ISOFORM TYPE-2.			X
IPI00010212	TUT1	U6 SNRNA-SPECIFIC TERMINAL URIDYLYLTRANSFERASE 1.			X
IPI00010277	TNFRSF12A	ISOFORM 1 OF TUMOR NECROSIS FACTOR RECEPTOR SUPERFAMILY MEMBER 12A PRECURSOR.			X
IPI00010334	PLCL2	ISOFORM 3 OF INACTIVE PHOSPHOLIPASE C-LIKE PROTEIN 2.			X
IPI00010420	SLC25A31	ADP/ATP TRANSLOCASE 4.			X
IPI00010448	ARHGAP24	ISOFORM 2 OF RHO GTPASE-ACTIVATING PROTEIN 24.			X
IPI00010604	PLCE1	ISOFORM 1 OF 1-PHOSPHATIDYLINOSITOL-4,5-BISPHOSPHATE PHOSPHODIESTERASE EPSILON-1.			X
IPI00010740	SFPQ	ISOFORM LONG OF SPLICING FACTOR, PROLINE- AND GLUTAMINE-RICH.			X
IPI00010807	FZD8	FRIZZLED-8 PRECURSOR.			X
IPI00010843	APBB1	ISOFORM 1 OF AMYLOID BETA A4 PRECURSOR PROTEIN-BINDING FAMILY B MEMBER 1.			X
IPI00010893	LEFTY2	LEFT-RIGHT DETERMINATION FACTOR 2 PRECURSOR.			X
IPI00010953	EXOC6	CDNA FLJ11251 FIS, CLONE PLACE1008813.			X

Table S1. Proteins Identified or Not in MS Patients Compared to Normals and Other Neurologic Disease (OND)					
**IPI**	**Gene**	**Protein Name**	**MS**	**Normal**	**OND**
IPI00011253	RPS3	40S RIBOSOMAL PROTEIN S3.			X
IPI00011274	HNRPDL	ISOFORM 1 OF HETEROGENEOUS NUCLEAR RIBONUCLEOPROTEIN D-LIKE.			X
IPI00011488	STK4	ISOFORM 1 OF SERINE/THREONINE-PROTEIN KINASE 4.			X
IPI00011589	SEPT4	ISOFORM A OF SEPTIN-4.			X
IPI00011604	GCSH; LOC730107	GLYCINE CLEAVAGE SYSTEM H PROTEIN, MITOCHONDRIAL PRECURSOR.			X
IPI00011631	ZW10	CENTROMERE/KINETOCHORE PROTEIN ZW10 HOMOLOG.			X
IPI00011652	EFS	ISOFORM EFS1 OF EMBRYONAL FYN-ASSOCIATED SUBSTRATE.			X
IPI00011756	MEIS1	HOMEOBOX PROTEIN MEIS1.			X
IPI00011773	C20orf177	UNCHARACTERIZED PROTEIN C20ORF177.			X
IPI00011832	SPP2	SECRETED PHOSPHOPROTEIN 24 PRECURSOR.			X
IPI00011894		MRNA CLONE WITH SIMILARITY TO L-GLYCEROL-3-PHOSPHATE:NAD OXIDOREDUCTASE AND ALBUMIN GENE SEQUENCES.			X
IPI00011951	KIAA0427	ISOFORM 2 OF UNCHARACTERIZED PROTEIN KIAA0427.			X
IPI00012079	EIF4B	EUKARYOTIC TRANSLATION INITIATION FACTOR 4B.			X
IPI00012353	MRPL3	MITOCHONDRIAL 39S RIBOSOMAL PROTEIN L3.			X
IPI00012402	CHST6	CARBOHYDRATE SULFOTRANSFERASE 6.			X
IPI00012411	ZNF32	ZINC FINGER PROTEIN 32.			X
IPI00012438	PRKRIR	ISOFORM LONG OF 52 KDA REPRESSOR OF THE INHIBITOR OF THE PROTEIN KINASE.			X
IPI00012451	GNB4	GUANINE NUCLEOTIDE-BINDING PROTEIN SUBUNIT BETA-4.			X
IPI00012480	RACGAP1P	FKSG42.			X
IPI00012573	PEX12	PEROXISOME ASSEMBLY PROTEIN 12.			X
IPI00012687	C1orf83	27 KDA PROTEIN.			X
IPI00012756	IFIT5	INTERFERON-INDUCED PROTEIN WITH TETRATRICOPEPTIDE REPEATS 5.			X
IPI00012759	CPLX2	COMPLEXIN-2.			X
IPI00012760	LEP	LEPTIN PRECURSOR.			X
IPI00012869	SMAD6	ISOFORM A OF MOTHERS AGAINST DECAPENTAPLEGIC HOMOLOG 6.			X
IPI00012891	PHKG2	PHOSPHORYLASE B KINASE GAMMA CATALYTIC CHAIN, TESTIS/LIVER ISOFORM.			X
IPI00012902	SYT7	SYNAPTOTAGMIN VII.			X
IPI00013079	EMILIN1	EMILIN-1 PRECURSOR.			X
IPI00013205	JMJD2B	ISOFORM 1 OF JMJC DOMAIN-CONTAINING HISTONE DEMETHYLATION PROTEIN 3B.			X
IPI00013256	CSTF2	ISOFORM 1 OF CLEAVAGE STIMULATION FACTOR 64 KDA SUBUNIT.			X
IPI00013257	SSBP4	SINGLE-STRANDED DNA-BINDING PROTEIN 4.			X
IPI00013260	LSP1	LYMPHOCYTE-SPECIFIC PROTEIN 1.			X

Table S1. Proteins Identified or Not in MS Patients Compared to Normals and Other Neurologic Disease (OND)					
**IPI**	**Gene**	**Protein Name**	**MS**	**Normal**	**OND**
IPI00013269	C6orf106	ISOFORM 1 OF UNCHARACTERIZED PROTEIN C6ORF106.			X
IPI00013296	RPS18	40S RIBOSOMAL PROTEIN S18.			X
IPI00013371	SPRY3	PROTEIN SPROUTY HOMOLOG 3.			X
IPI00013373	HCRT	OREXIN PRECURSOR.			X
IPI00013378	TOP3A	ISOFORM LONG OF DNA TOPOISOMERASE 3-ALPHA.			X
IPI00013404	SMAD4	MOTHERS AGAINST DECAPENTAPLEGIC HOMOLOG 4.			X
IPI00013418	BIRC2	BACULOVIRAL IAP REPEAT-CONTAINING PROTEIN 2.			X
IPI00013438	IGLL1	IMMUNOGLOBULIN LAMBDA-LIKE POLYPEPTIDE 1 PRECURSOR.			X
IPI00013468	BUB3	MITOTIC CHECKPOINT PROTEIN BUB3.			X
IPI00013488	CHN1	ISOFORM ALPHA-2 OF N-CHIMAERIN.			X
IPI00013651	RBM33	ISOFORM 3 OF RNA-BINDING PROTEIN 33.			X
IPI00013721	PRPF4B	SERINE/THREONINE-PROTEIN KINASE PRP4 HOMOLOG.			X
IPI00013743	BUD13	ISOFORM 1 OF BUD13 HOMOLOG.			X
IPI00013788	HTATSF1	HIV TAT-SPECIFIC FACTOR 1.			X
IPI00013789	SMYD5	SET AND MYND DOMAIN-CONTAINING PROTEIN 5.			X
IPI00013871	RRM1	RIBONUCLEOSIDE-DIPHOSPHATE REDUCTASE LARGE SUBUNIT.			X
IPI00013880	SEMA5A	SEMAPHORIN-5A PRECURSOR.			X
IPI00013887	ST6GAL1	BETA-GALACTOSIDE ALPHA-2,6-SIALYLTRANSFERASE 1.			X
IPI00013895	S100A11	PROTEIN S100-A11.			X
IPI00014230	C1QBP	COMPLEMENT COMPONENT 1 Q SUBCOMPONENT-BINDING PROTEIN, MITOCHONDRIAL PRECURSOR.			X
IPI00014232	ARL6IP1	ADP-RIBOSYLATION FACTOR-LIKE PROTEIN 6-INTERACTING PROTEIN 1.			X
IPI00014316	CDK5R1	CYCLIN-DEPENDENT KINASE 5 ACTIVATOR 1 PRECURSOR.			X
IPI00014367	NSMCE4A	ISOFORM 1 OF NON-SMC ELEMENT 4 HOMOLOG A.			X
IPI00014577	RAB18	RAS-RELATED PROTEIN RAB-18.			X
IPI00014877	QPCTL	ISOFORM 2 OF GLUTAMINYL-PEPTIDE CYCLOTRANSFERASE-LIKE PROTEIN.			X
IPI00014878	PRKD1	UNCHARACTERIZED PROTEIN PRKD1.			X
IPI00014897	PLCB4	ISOFORM 2 OF 1-PHOSPHATIDYLINOSITOL-4,5-BISPHOSPHATE PHOSPHODIESTERASE BETA-4.			X
IPI00014903	FGFR1OP2	ISOFORM 1 OF FGFR1 ONCOGENE PARTNER 2.			X
IPI00014958	PON2	ISOFORM 1 OF SERUM PARAOXONASE/ARYLESTERASE 2.			X
IPI00015135	EXTL3	EXOSTOSIN-LIKE 3.			X
IPI00015159	EFNA2	EPHRIN-A2 PRECURSOR.			X
IPI00015181	ITGA9	INTEGRIN ALPHA-9 PRECURSOR.			X

Table S1. Proteins Identified or Not in MS Patients Compared to Normals and Other Neurologic Disease (OND)					
**IPI**	**Gene**	**Protein Name**	**MS**	**Normal**	**OND**
IPI00015195	CSTF3	CLEAVAGE STIMULATION FACTOR 77 KDA SUBUNIT.			X
IPI00015345	CELSR3; SLC26A6	CADHERIN EGF LAG SEVEN-PASS G-TYPE RECEPTOR 3 PRECURSOR.			X
IPI00015473	SLC1A3	EXCITATORY AMINO ACID TRANSPORTER 1.			X
IPI00015602	TOMM70A	MITOCHONDRIAL PRECURSOR PROTEINS IMPORT RECEPTOR.			X
IPI00015737	DCAKD	15 KDA PROTEIN.			X
IPI00015782	C16orf35	UPF0171 PROTEIN C16ORF35.			X
IPI00015802	ZFPM2	ISOFORM 1 OF ZINC FINGER PROTEIN ZFPM2.			X
IPI00015990	EYA3	ISOFORM 1 OF EYES ABSENT HOMOLOG 3.			X
IPI00016006	GPHN	ISOFORM 1 OF GEPHYRIN.			X
IPI00016007	MYO5C	MYOSIN-VC.			X
IPI00016046	C20orf52	PROTEIN MGR2 HOMOLOG.			X
IPI00016095	MTERF	TRANSCRIPTION TERMINATION FACTOR, MITOCHONDRIAL PRECURSOR.			X
IPI00016373	RAB13	RAS-RELATED PROTEIN RAB-13.			X
IPI00016480	NLRP2	ISOFORM 1 OF NACHT, LRR AND PYD DOMAINS-CONTAINING PROTEIN 2.			X
IPI00016600	TSPAN7	TETRASPANIN-7.			X
IPI00016604	PDE4B	ISOFORM PDE4B1 OF CAMP-SPECIFIC 3',5'-CYCLIC PHOSPHODIESTERASE 4B.			X
IPI00016859	BRF1	ISOFORM 1 OF TRANSCRIPTION FACTOR IIIB 90 KDA SUBUNIT.			X
IPI00016861	GTF3C2	102 KDA PROTEIN.			X
IPI00016940	PLSCR2	PHOSPHOLIPID SCRAMBLASE 2.			X
IPI00016988	WDR13	WD REPEAT-CONTAINING PROTEIN 13.			X
IPI00017203	RMI1	PROTEIN RMI1 HOMOLOG.			X
IPI00017297	MATR3	MATRIN-3.			X
IPI00017533	COX3	CYTOCHROME C OXIDASE SUBUNIT 3.			X
IPI00017538	ESPL1	ISOFORM 2 OF SEPARIN.			X
IPI00017551	RGN	ISOFORM 1 OF REGUCALCIN.			X
IPI00017592	LETM1	LEUCINE ZIPPER-EF-HAND-CONTAINING TRANSMEMBRANE PROTEIN 1, MITOCHONDRIAL PRECURSOR.			X
IPI00017603	F8	COAGULATION FACTOR VIII PRECURSOR.			X
IPI00017630	NUFIP1	NUCLEAR FRAGILE X MENTAL RETARDATION-INTERACTING PROTEIN 1.			X
IPI00017640	SLIT3	ISOFORM 1 OF SLIT HOMOLOG 3 PROTEIN PRECURSOR.			X
IPI00017726	HSD17B10	ISOFORM 1 OF 3-HYDROXYACYL-COA DEHYDROGENASE TYPE-2.			X
IPI00017800	ABCA3	ATP-BINDING CASSETTE SUB-FAMILY A MEMBER 3.			X

Table S1. Proteins Identified or Not in MS Patients Compared to Normals and Other Neurologic Disease (OND)					
**IPI**	**Gene**	**Protein Name**	**MS**	**Normal**	**OND**
IPI00017802	AUH	ISOFORM 1 OF METHYLGLUTACONYL-COA HYDRATASE, MITOCHONDRIAL PRECURSOR.			X
IPI00017819	BAAT	BILE ACID-COA:AMINO ACID N-ACYLTRANSFERASE.			X
IPI00017991	JMJD2D	JUMONJI DOMAIN CONTAINING 2D.			X
IPI00018099	FANCL	E3 UBIQUITIN-PROTEIN LIGASE FANCL.			X
IPI00018203	SFRS6	ISOFORM SRP55-2 OF SPLICING FACTOR, ARGININE/SERINE-RICH 6.			X
IPI00018240	SDAD1	ISOFORM 1 OF PROTEIN SDA1 HOMOLOG.			X
IPI00018245	POLR3E	ISOFORM 3 OF DNA-DIRECTED RNA POLYMERASE III SUBUNIT RPC5.			X
IPI00018402	TBCE	TUBULIN-SPECIFIC CHAPERONE E.			X
IPI00018583	HABP4	ISOFORM 1 OF INTRACELLULAR HYALURONAN-BINDING PROTEIN 4.			X
IPI00018691	MRPS18A	28S RIBOSOMAL PROTEIN S18A, MITOCHONDRIAL PRECURSOR.			X
IPI00018805	C14orf105	UNCHARACTERIZED PROTEIN C14ORF105.			X
IPI00018854	CCDC68	COILED-COIL DOMAIN-CONTAINING PROTEIN 68.			X
IPI00018880	TNFRSF1A	TUMOR NECROSIS FACTOR RECEPTOR SUPERFAMILY MEMBER 1A PRECURSOR.			X
IPI00018953	DPP4	DIPEPTIDYL PEPTIDASE 4.			X
IPI00018956	ALX1	ALX HOMEOBOX PROTEIN 1.			X
IPI00019090	COL19A1	COLLAGEN ALPHA-1.			X
IPI00019243	MMP16	ISOFORM LONG OF MATRIX METALLOPROTEINASE-16 PRECURSOR.			X
IPI00019244	NOVA1	ISOFORM 1 OF RNA-BINDING PROTEIN NOVA-1.			X
IPI00019270	TNKS2	TANKYRASE-2.			X
IPI00019278	SLC19A3	THIAMINE TRANSPORTER 2.			X
IPI00019690	DIP	DEATH-INDUCING-PROTEIN.			X
IPI00019848	HCFC1	ISOFORM 1 OF HOST CELL FACTOR.			X
IPI00019932	UBE2D2	UBIQUITIN-CONJUGATING ENZYME E2D 2 ISOFORM 2.			X
IPI00019992	MYBL1	MYB-RELATED PROTEIN A.			X
IPI00020005	ARSE	ARYLSULFATASE E PRECURSOR.			X
IPI00020017	C10orf116	ADIPOSE MOST ABUNDANT GENE TRANSCRIPT 2 PROTEIN.			X
IPI00020036	CHRNA4	NEURONAL ACETYLCHOLINE RECEPTOR SUBUNIT ALPHA-4 PRECURSOR.			X
IPI00020039	CYLC1	CYLICIN-1.			X
IPI00020230	FZD9	FRIZZLED-9 PRECURSOR.			X
IPI00020354	IL13RA1	INTERLEUKIN-13 RECEPTOR ALPHA-1 CHAIN PRECURSOR.			X
IPI00020454	DCK	DEOXYCYTIDINE KINASE.			X
IPI00020533	BEX1	PROTEIN BEX1.			X
IPI00020565		PRO1598.			X

Table S1. Proteins Identified or Not in MS Patients Compared to Normals and Other Neurologic Disease (OND)					
**IPI**	**Gene**	**Protein Name**	**MS**	**Normal**	**OND**
IPI00020729	IRS4	INSULIN RECEPTOR SUBSTRATE 4.			X
IPI00020754		PRO1751.			X
IPI00020771	MAP7	ISOFORM 3 OF ENSCONSIN.			X
IPI00020795	DPP9	R33083_1 (FRAGMENT).			X
IPI00020903	AFF2	ISOFORM 1 OF AF4/FMR2 FAMILY MEMBER 2.			X
IPI00021131	LGR5	LEUCINE-RICH REPEAT-CONTAINING G-PROTEIN COUPLED RECEPTOR 5 PRECURSOR.			X
IPI00021143	BHLHB2	CLASS B BASIC HELIX-LOOP-HELIX PROTEIN 2.			X
IPI00021266	hCG_16001; RPL23A	60S RIBOSOMAL PROTEIN L23A.			X
IPI00021302	SUSD2	SUSHI DOMAIN-CONTAINING PROTEIN 2 PRECURSOR.			X
IPI00021338	DLAT	DIHYDROLIPOYLLYSINE-RESIDUE ACETYLTRANSFERASE COMPONENT OF PYRUVATE DEHYDROGENASE COMPLEX, MITOCHONDRIAL PRECURSOR.			X
IPI00021458	EHD3	EH DOMAIN-CONTAINING PROTEIN 3.			X
IPI00021473	CBFA2T3	59 KDA PROTEIN.			X
IPI00021715	COL4A5	ISOFORM 1 OF COLLAGEN ALPHA-5(IV) CHAIN PRECURSOR.			X
IPI00021751	NEFH	NEUROFILAMENT HEAVY POLYPEPTIDE.			X
IPI00021772	MAT1A	S-ADENOSYLMETHIONINE SYNTHETASE ISOFORM TYPE-1.			X
IPI00021828	CSTB	CYSTATIN-B.			X
IPI00021979	PEX11A	PEROXISOMAL MEMBRANE PROTEIN 11A.			X
IPI00022048	PTGFRN	PROSTAGLANDIN F2 RECEPTOR NEGATIVE REGULATOR PRECURSOR.			X
IPI00022061	SLC9A6	SODIUM/HYDROGEN EXCHANGER 6.			X
IPI00022143	FAM62A	ISOFORM 1 OF EXTENDED-SYNAPTOTAGMIN-1.			X
IPI00022228	HDLBP	VIGILIN.			X
IPI00022250	LY96	LYMPHOCYTE ANTIGEN 96 PRECURSOR.			X
IPI00022256	AP2M1	AP-2 COMPLEX SUBUNIT MU-1.			X
IPI00022290	DEFB1	BETA-DEFENSIN 1 PRECURSOR.			X
IPI00022325	NRG2	ISOFORM 4 OF PRO-NEUREGULIN-2, MEMBRANE-BOUND ISOFORM PRECURSOR.			X
IPI00022334	OAT	ORNITHINE AMINOTRANSFERASE, MITOCHONDRIAL PRECURSOR.			X
IPI00022433	HSPB6	HEAT-SHOCK PROTEIN BETA-6.			X
IPI00022438	MAF	ISOFORM LONG OF TRANSCRIPTION FACTOR MAF.			X
IPI00022471	HMHA1	MINOR HISTOCOMPATIBILITY ANTIGEN HA-1.			X
IPI00022479	HERC1	GUANINE NUCLEOTIDE EXCHANGE FACTOR P532.			X
IPI00022621	MFAP2	MICROFIBRILLAR-ASSOCIATED PROTEIN 2 PRECURSOR.			X

Table S1. Proteins Identified or Not in MS Patients Compared to Normals and Other Neurologic Disease (OND)					
**IPI**	**Gene**	**Protein Name**	**MS**	**Normal**	**OND**
IPI00022735	SYT4	SYNAPTOTAGMIN-4.			X
IPI00022799	AQP4	ISOFORM 2 OF AQUAPORIN-4.			X
IPI00022820	GTF2B	TRANSCRIPTION INITIATION FACTOR IIB.			X
IPI00022830	NSFL1C	ISOFORM 2 OF NSFL1 COFACTOR P47.			X
IPI00022881	CLTCL1	ISOFORM 1 OF CLATHRIN HEAVY CHAIN 2.			X
IPI00022918	FAM125B	ISOFORM 2 OF PROTEIN FAM125B.			X
IPI00023095	MLF2	MYELOID LEUKEMIA FACTOR 2.			X
IPI00023110	KCNK3	POTASSIUM CHANNEL SUBFAMILY K MEMBER 3.			X
IPI00023164	HCN4	POTASSIUM/SODIUM HYPERPOLARIZATION-ACTIVATED CYCLIC NUCLEOTIDE-GATED CHANNEL 4.			X
IPI00023330	KIAA1468	ISOFORM 2 OF LISH DOMAIN AND HEAT REPEAT-CONTAINING PROTEIN KIAA1468.			X
IPI00023410	ITGA8	INTEGRIN ALPHA-8 PRECURSOR.			X
IPI00023555	OSBPL3	ISOFORM 1A OF OXYSTEROL-BINDING PROTEIN-RELATED PROTEIN 3.			X
IPI00023586	SENP6	ISOFORM 1 OF SENTRIN-SPECIFIC PROTEASE 6.			X
IPI00023617	ZNF197	ZINC FINGER PROTEIN 197.			X
IPI00023635	IMPA2	ISOFORM 1 OF INOSITOL MONOPHOSPHATASE 2.			X
IPI00023663	OVOL1	PUTATIVE TRANSCRIPTION FACTOR OVO-LIKE 1.			X
IPI00023757	RPGR	ISOFORM 1 OF X-LINKED RETINITIS PIGMENTOSA GTPASE REGULATOR.			X
IPI00023843	DNASE1L2	DEOXYRIBONUCLEASE I-LIKE 2 PRECURSOR.			X
IPI00023856	BRPF1	PEREGRIN.			X
IPI00023860	NAP1L1	NUCLEOSOME ASSEMBLY PROTEIN 1-LIKE 1.			X
IPI00023919	PSMC5	26S PROTEASE REGULATORY SUBUNIT 8.			X
IPI00024255	GPKOW	G PATCH DOMAIN AND KOW MOTIFS-CONTAINING PROTEIN.			X
IPI00024285	LOC138652	SIMILAR TO TESTICULAR SERINE PROTEASE 2.			X
IPI00024292	LRP2	LOW-DENSITY LIPOPROTEIN RECEPTOR-RELATED PROTEIN 2 PRECURSOR.			X
IPI00024319	FOXJ1	FORKHEAD BOX PROTEIN J1.			X
IPI00024523	DNAJB6	ISOFORM A OF DNAJ HOMOLOG SUBFAMILY B MEMBER 6.			X
IPI00024776	CLGN	CALMEGIN PRECURSOR.			X
IPI00024801	RNF41	E3 UBIQUITIN-PROTEIN LIGASE NRDP1.			X
IPI00024915	PRDX5	ISOFORM MITOCHONDRIAL OF PEROXIREDOXIN-5, MITOCHONDRIAL PRECURSOR.			X
IPI00025039	FBL	RRNA 2'-O-METHYLTRANSFERASE FIBRILLARIN.			X
IPI00025308	PROP1	HOMEOBOX PROTEIN PROPHET OF PIT-1.			X
IPI00025310	ZNF217	ZINC FINGER PROTEIN 217.			X
IPI00025489	MAK	SERINE/THREONINE-PROTEIN KINASE MAK.			X

Table S1. Proteins Identified or Not in MS Patients Compared to Normals and Other Neurologic Disease (OND)					
**IPI**	**Gene**	**Protein Name**	**MS**	**Normal**	**OND**
IPI00025717	MTX2	METAXIN-2.			X
IPI00025721	COPS3	COP9 SIGNALOSOME COMPLEX SUBUNIT 3.			X
IPI00025861	CDH1	EPITHELIAL CADHERIN PRECURSOR.			X
IPI00025862	C4BPB	ISOFORM 1 OF C4B-BINDING PROTEIN BETA CHAIN PRECURSOR.			X
IPI00025974	CHMP4B	CHARGED MULTIVESICULAR BODY PROTEIN 4B.			X
IPI00026036	DPP8	ISOFORM 5 OF DIPEPTIDYL PEPTIDASE 8.			X
IPI00026058	LZTS1	ISOFORM 1 OF LEUCINE ZIPPER PUTATIVE TUMOR SUPPRESSOR 1.			X
IPI00026105	SCP2	ISOFORM SCPX OF NON-SPECIFIC LIPID-TRANSFER PROTEIN.			X
IPI00026126	SCGB2A1	MAMMAGLOBIN-B PRECURSOR.			X
IPI00026185	CAPZB	ISOFORM 1 OF F-ACTIN-CAPPING PROTEIN SUBUNIT BETA.			X
IPI00026305	HOOK1	HOOK HOMOLOG 1.			X
IPI00026320	UBR5	E3 UBIQUITIN-PROTEIN LIGASE UBR5.			X
IPI00026492	GCAT	2-AMINO-3-KETOBUTYRATE COENZYME A LIGASE, MITOCHONDRIAL PRECURSOR.			X
IPI00026497	PARP2	ISOFORM 1 OF POLY [ADP-RIBOSE] POLYMERASE 2.			X
IPI00026512	ERAL1	ISOFORM HERA-A OF GTP-BINDING PROTEIN ERA HOMOLOG.			X
IPI00026646	FCGRT	IGG RECEPTOR FCRN LARGE SUBUNIT P51 PRECURSOR (FRAGMENT).			X
IPI00026941	PRSS23	SERINE PROTEASE 23 PRECURSOR.			X
IPI00026952	PKP3	PLAKOPHILIN-3.			X
IPI00027144	CYB561	CYTOCHROME B561.			X
IPI00027220	CDON	ISOFORM 1 OF CELL ADHESION MOLECULE-RELATED/DOWN-REGULATED BY ONCOGENES PRECURSOR.			X
IPI00027228	PET112L	PROBABLE GLUTAMYL-TRNA(GLN) AMIDOTRANSFERASE SUBUNIT B, MITOCHONDRIAL PRECURSOR.			X
IPI00027232	IGF1R	INSULIN-LIKE GROWTH FACTOR 1 RECEPTOR PRECURSOR.			X
IPI00027269	CBL	E3 UBIQUITIN-PROTEIN LIGASE CBL.			X
IPI00027356	ADCY8	ADENYLATE CYCLASE TYPE 8.			X
IPI00027415	DHX36	ISOFORM 1 OF PROBABLE ATP-DEPENDENT RNA HELICASE DHX36.			X
IPI00027422	ITGB4	ISOFORM BETA-4C OF INTEGRIN BETA-4 PRECURSOR.			X
IPI00027436	NGFR	TUMOR NECROSIS FACTOR RECEPTOR SUPERFAMILY MEMBER 16 PRECURSOR.			X
IPI00027442	AARS	ALANYL-TRNA SYNTHETASE, CYTOPLASMIC.			X
IPI00027487	CKM	CREATINE KINASE M-TYPE.			X
IPI00027596	SFRP2	SECRETED FRIZZLED-RELATED PROTEIN 2 PRECURSOR.			X
IPI00027642	JMJD2C	ISOFORM 1 OF JMJC DOMAIN-CONTAINING HISTONE DEMETHYLATION PROTEIN 3C.			X
IPI00027667	CCKBR	GASTRIN/CHOLECYSTOKININ TYPE B RECEPTOR.			X

Table S1. Proteins Identified or Not in MS Patients Compared to Normals and Other Neurologic Disease (OND)					
**IPI**	**Gene**	**Protein Name**	**MS**	**Normal**	**OND**
IPI00027694	HOXA1	ISOFORM 3 OF HOMEOBOX PROTEIN HOX-A1.			X
IPI00027723	ELA2B	ELASTASE-2B PRECURSOR.			X
IPI00027774	THAP2	THAP DOMAIN-CONTAINING PROTEIN 2.			X
IPI00028050	EEFSEC	SELENOCYSTEINE-SPECIFIC ELONGATION FACTOR.			X
IPI00028066	ADH7	CLASS IV ALCOHOL DEHYDROGENASE 7 MU OR SIGMA SUBUNIT.			X
IPI00028122	PSIP1	ISOFORM 1 OF PC4 AND SFRS1-INTERACTING PROTEIN.			X
IPI00028158	NTS	NEUROTENSIN/NEUROMEDIN N PRECURSOR.			X
IPI00028213	SEMA3D	SEMAPHORIN-3D PRECURSOR.			X
IPI00028232	FGF4	PUTATIVE UNCHARACTERIZED PROTEIN.			X
IPI00028262	KIAA1754	67 KDA PROTEIN.			X
IPI00028357	XPO4	EXPORTIN-4.			X
IPI00028392	C1orf116	SPECIFICALLY ANDROGEN-REGULATED PROTEIN ISOFORM 1.			X
IPI00028493	TSC2	ISOFORM 1 OF TUBERIN.			X
IPI00028579	TAF6	TRANSCRIPTION INITIATION FACTOR TFIID SUBUNIT 6.			X
IPI00028641	PPP1R12B	ISOFORM 4 OF PROTEIN PHOSPHATASE 1 REGULATORY SUBUNIT 12B.			X
IPI00028828	ZNF202	ISOFORM BETA OF ZINC FINGER PROTEIN 202.			X
IPI00028864	NXPH3	NEUREXOPHILIN-3 PRECURSOR.			X
IPI00028880	POLN	PUTATIVE DNA POLYMERASE.			X
IPI00028881	NDUFA3	NADH DEHYDROGENASE [UBIQUINONE] 1 ALPHA SUBCOMPLEX SUBUNIT 3.			X
IPI00028980	KIAA0133	UNCHARACTERIZED PROTEIN KIAA0133.			X
IPI00029039	REG3A	REGENERATING ISLET-DERIVED PROTEIN 3 ALPHA PRECURSOR.			X
IPI00029133	ATP5F1	ATP SYNTHASE SUBUNIT B, MITOCHONDRIAL PRECURSOR.			X
IPI00029144	PPP2R3A	ISOFORM PR130 OF SERINE/THREONINE-PROTEIN PHOSPHATASE 2A REGULATORY SUBUNIT B'' SUBUNIT ALPHA.			X
IPI00029162	CDC2L5	ISOFORM 2 OF CELL DIVISION CYCLE 2-LIKE PROTEIN KINASE 5.			X
IPI00029184	HAPLN2	HYALURONAN AND PROTEOGLYCAN LINK PROTEIN 2 PRECURSOR.			X
IPI00029227	LOC93622	LOC93622 PROTEIN.			X
IPI00029446	SBF1	ISOFORM 1 OF MYOTUBULARIN-RELATED PROTEIN 5.			X
IPI00029473	C17orf75	PROTEIN NJMU-R1.			X
IPI00029631	ERH	ENHANCER OF RUDIMENTARY HOMOLOG.			X
IPI00029662	KCNH2	ISOFORM 1 OF POTASSIUM VOLTAGE-GATED CHANNEL SUBFAMILY H MEMBER 2.			X
IPI00029737	ACSL4	ISOFORM LONG OF LONG-CHAIN-FATTY-ACID--COA LIGASE 4.			X
IPI00029769	HCK	ISOFORM P59-HCK OF TYROSINE-PROTEIN KINASE HCK.			X
IPI00030070	GJB5	GAP JUNCTION BETA-5 PROTEIN.			X

Table S1. Proteins Identified or Not in MS Patients Compared to Normals and Other Neurologic Disease (OND)					
**IPI**	**Gene**	**Protein Name**	**MS**	**Normal**	**OND**
IPI00030099	ADCY9	ADENYLATE CYCLASE TYPE 9.			X
IPI00030237	C1orf49	ISOFORM 1 OF UNCHARACTERIZED PROTEIN C1ORF49.			X
IPI00030279	ZRANB3	ISOFORM 1 OF ZINC FINGER RAN-BINDING DOMAIN-CONTAINING PROTEIN 3.			X
IPI00030307	SLC41A2	SOLUTE CARRIER FAMILY 41 MEMBER 2.			X
IPI00030355	PPP1R11	PROTEIN PHOSPHATASE 1 REGULATORY SUBUNIT 11.			X
IPI00030360	RUNDC2A	RUN DOMAIN-CONTAINING PROTEIN 2A.			X
IPI00030404	NFX1	TRANSCRIPTIONAL REPRESSOR NF-X1.			X
IPI00030487	KIAA1627	CDNA FLJ30993 FIS, CLONE HLUNG1000064, WEAKLY SIMILAR TO KARYOGAMY PROTEIN KAR4.			X
IPI00030648	ZFYVE9	ISOFORM 1 OF ZINC FINGER FYVE DOMAIN-CONTAINING PROTEIN 9.			X
IPI00030703	MNX1	MOTOR NEURON AND PANCREAS HOMEOBOX PROTEIN 1.			X
IPI00030814	FGF22	FIBROBLAST GROWTH FACTOR 22 PRECURSOR.			X
IPI00030874		UNCHARACTERIZED GASTRIC PROTEIN YA42P (FRAGMENT).			X
IPI00030909		BB1 FAMILY PROTEIN.			X
IPI00030945	UCN2	UROCORTIN-2 PRECURSOR.			X
IPI00030986	KBTBD10	ISOFORM LONG OF KELCH REPEAT AND BTB DOMAIN-CONTAINING PROTEIN 10.			X
IPI00031047	IQCG	CDNA: FLJ23571 FIS, CLONE LNG12303.			X
IPI00031056	ZMYND15	ZINC FINGER MYND DOMAIN-CONTAINING PROTEIN 15.			X
IPI00031082	LDOC1L	PUTATIVE UNCHARACTERIZED PROTEIN DKFZP761O17121.			X
IPI00031101	NPPB	NATRIURETIC PEPTIDES B PRECURSOR.			X
IPI00031195	VANGL1	ISOFORM 1 OF VANG-LIKE PROTEIN 1.			X
IPI00031228	PLEKHB2	ISOFORM 2 OF PLECKSTRIN HOMOLOGY DOMAIN-CONTAINING FAMILY B MEMBER 2.			X
IPI00031288	CLDN14	CLAUDIN-14.			X
IPI00031386	PIK3CA	PHOSPHATIDYLINOSITOL-4,5-BISPHOSPHATE 3-KINASE CATALYTIC SUBUNIT ALPHA ISOFORM.			X
IPI00031410	FRAP1	FKBP12-RAPAMYCIN COMPLEX-ASSOCIATED PROTEIN.			X
IPI00031497	C7orf49	CONSERVED HYPOTHETICAL PROTEIN.			X
IPI00031522	HADHA	TRIFUNCTIONAL ENZYME SUBUNIT ALPHA, MITOCHONDRIAL PRECURSOR.			X
IPI00031554	DDX50	ATP-DEPENDENT RNA HELICASE DDX50.			X
IPI00031768	HOOK3	HOOK HOMOLOG 3.			X
IPI00031812	YBX1	NUCLEASE SENSITIVE ELEMENT-BINDING PROTEIN 1.			X
IPI00031960	POLR1A	POLYMERASE (RNA) I POLYPEPTIDE A, 194KDA.			X
IPI00032158	NARG1	ISOFORM 2 OF NMDA RECEPTOR-REGULATED PROTEIN 1.			X
IPI00032342	TRIP12	TRIP12 PROTEIN.			X

Table S1. Proteins Identified or Not in MS Patients Compared to Normals and Other Neurologic Disease (OND)					
**IPI**	**Gene**	**Protein Name**	**MS**	**Normal**	**OND**
IPI00032358	POM121	NUCLEAR ENVELOPE PORE MEMBRANE PROTEIN POM 121.			X
IPI00032541	KRT85	KERATIN TYPE II CUTICULAR HB5.			X
IPI00032598	ETV3	ISOFORM 1 OF ETS TRANSLOCATION VARIANT 3.			X
IPI00032680	APOL5	APOLIPOPROTEIN-L5.			X
IPI00032785	APBB3	ISOFORM II OF AMYLOID BETA A4 PRECURSOR PROTEIN-BINDING FAMILY B MEMBER 3.			X
IPI00032876	CYTL1	CYTOKINE-LIKE PROTEIN 1 PRECURSOR.			X
IPI00032929	THSD1	ISOFORM 1 OF THROMBOSPONDIN TYPE-1 DOMAIN-CONTAINING PROTEIN 1 PRECURSOR.			X
IPI00032939	WIPF3	UNCHARACTERIZED PROTEIN WIPF3.			X
IPI00032958	ANLN	ISOFORM 2 OF ACTIN-BINDING PROTEIN ANILLIN.			X
IPI00033019	KCNB1	POTASSIUM VOLTAGE-GATED CHANNEL SUBFAMILY B MEMBER 1.			X
IPI00034088	EBI3	INTERLEUKIN-27 BETA CHAIN PRECURSOR.			X
IPI00034277	ATP13A1	ISOFORM A OF PROBABLE CATION-TRANSPORTING ATPASE 13A1.			X
IPI00039626	FAM120A	ISOFORM D OF UPF0318 PROTEIN FAM120A.			X
IPI00043069	ANKRD30A	ANKYRIN REPEAT DOMAIN-CONTAINING PROTEIN 30A.			X
IPI00043237	LOC730193; STX6	CDNA FLJ32095 FIS, CLONE OCBBF2000998.			X
IPI00043294	C7orf26	ISOFORM 1 OF UNCHARACTERIZED PROTEIN C7ORF26.			X
IPI00043370	WDR52	WD REPEAT-CONTAINING PROTEIN 52.			X
IPI00043467	TIGD1	TIGGER TRANSPOSABLE ELEMENT-DERIVED PROTEIN 1.			X
IPI00043499	UROC1	PROBABLE UROCANATE HYDRATASE.			X
IPI00043526	C14orf44	UNCHARACTERIZED PROTEIN C14ORF44.			X
IPI00044608	KIAA1881	ISOFORM 1 OF PROTEIN KIAA1881.			X
IPI00044761	PUS7	PSEUDOURIDYLATE SYNTHASE 7 HOMOLOG.			X
IPI00045208	ZNF300	ZINC FINGER PROTEIN 300-B.			X
IPI00045473	LOC339123	SIMILAR TO C. ELEGANS PROTEIN F17C8.5.			X
IPI00045503	PPP1R14A	ISOFORM 2 OF PROTEIN PHOSPHATASE 1 REGULATORY SUBUNIT 14A.			X
IPI00045801	FIZ1	FLT3-INTERACTING ZINC FINGER PROTEIN 1.			X
IPI00054042	GTF2I	ISOFORM 1 OF GENERAL TRANSCRIPTION FACTOR II-I.			X
IPI00056324	HPS3	ISOFORM 1 OF HERMANSKY-PUDLAK SYNDROME 3 PROTEIN.			X
IPI00056507	FBXW5	ISOFORM 1 OF F-BOX/WD REPEAT-CONTAINING PROTEIN 5.			X
IPI00059139	ATP6V1E2	VACUOLAR PROTON PUMP SUBUNIT E 2.			X
IPI00059144	FAM46B	PROTEIN FAM46B.			X

Table S1. Proteins Identified or Not in MS Patients Compared to Normals and Other Neurologic Disease (OND)					
**IPI**	**Gene**	**Protein Name**	**MS**	**Normal**	**OND**
IPI00059190	SLAMF9	ISOFORM 1 OF SLAM FAMILY MEMBER 9 PRECURSOR.			X
IPI00059711	LRP11	LRP11 PROTEIN (FRAGMENT).			X
IPI00060379	INTS12	INTEGRATOR COMPLEX SUBUNIT 12.			X
IPI00060423	CTHRC1	ISOFORM 1 OF COLLAGEN TRIPLE HELIX REPEAT-CONTAINING PROTEIN 1 PRECURSOR.			X
IPI00060439	KIAA0226	KIAA0226 PROTEIN.			X
IPI00060549	GPRASP1	G-PROTEIN COUPLED RECEPTOR-ASSOCIATED SORTING PROTEIN 1.			X
IPI00060569	ABHD12	ISOFORM 2 OF ABHYDROLASE DOMAIN-CONTAINING PROTEIN 12.			X
IPI00060627	CCDC124	COILED-COIL DOMAIN-CONTAINING PROTEIN 124.			X
IPI00060866	AP1S3	ISOFORM 1 OF AP-1 COMPLEX SUBUNIT SIGMA-3.			X
IPI00060969	FAM55B	ISOFORM 1 OF PROTEIN FAM55B.			X
IPI00061009	WDR67	ISOFORM 1 OF WD REPEAT-CONTAINING PROTEIN 67.			X
IPI00061148	SLC4A5	SODIUM BICARBONATE COTRANSPORTER NBC4D.			X
IPI00061206	PABPC5	POLYADENYLATE-BINDING PROTEIN 5.			X
IPI00061355	ZNF673	ISOFORM 3 OF PROTEIN ZNF673.			X
IPI00061680	CCDC101	SAGA-ASSOCIATED FACTOR 29 HOMOLOG.			X
IPI00061780	ITCH	ISOFORM 1 OF E3 UBIQUITIN-PROTEIN LIGASE ITCHY HOMOLOG.			X
IPI00062599		71 KDA PROTEIN.			X
IPI00062882	ATPBD3	ATP-BINDING DOMAIN-CONTAINING PROTEIN 3.			X
IPI00063181	CDKN2AIPNL	CDKN2A INTERACTING PROTEIN N-TERMINAL LIKE.			X
IPI00063523	FLJ40176	SIMILAR TO TEMPORARILY ASSIGNED GENE NAME FAMILY MEMBER.			X
IPI00063878	ABCC12	ISOFORM 1 OF MULTIDRUG RESISTANCE-ASSOCIATED PROTEIN 9.			X
IPI00064606	MEGF11	ISOFORM 1 OF MULTIPLE EPIDERMAL GROWTH FACTOR-LIKE DOMAINS 11 PRECURSOR.			X
IPI00065057	CXorf58	PUTATIVE UNCHARACTERIZED PROTEIN CXORF58.			X
IPI00065085	FLJ25371	CDNA FLJ25371 FIS, CLONE TST01885.			X
IPI00065121	CCDC36	ISOFORM 2 OF COILED-COIL DOMAIN-CONTAINING PROTEIN 36.			X
IPI00065253	C3orf30	UNCHARACTERIZED PROTEIN C3ORF30.			X
IPI00065352	FLJ25006	ISOFORM 1 OF UNCHARACTERIZED SERINE/THREONINE-PROTEIN KINASE SGK494.			X
IPI00065378	DCST1	ISOFORM 1 OF DC-STAMP DOMAIN-CONTAINING PROTEIN 1.			X
IPI00065388	CCDC17	ISOFORM 1 OF COILED-COIL DOMAIN-CONTAINING PROTEIN 17.			X
IPI00065415	CCDC138	ISOFORM 1 OF COILED-COIL DOMAIN-CONTAINING PROTEIN 138.			X
IPI00065457	FLJ32682	OTTHUMP00000018353.			X

Table S1. Proteins Identified or Not in MS Patients Compared to Normals and Other Neurologic Disease (OND)					
**IPI**	**Gene**	**Protein Name**	**MS**	**Normal**	**OND**
IPI00065484	PTRH2	CDNA FLJ32471 FIS, CLONE SKNMC2000322, WEAKLY SIMILAR TO MAJOR CENTROMERE AUTOANTIGEN B.			X
IPI00065500	C1orf58	BRO1 DOMAIN-CONTAINING PROTEIN BROX.			X
IPI00065520	PPM1M	ISOFORM 1 OF PROTEIN PHOSPHATASE 1M.			X
IPI00065547	RP11-93B14.6	OTTHUMP00000031495.			X
IPI00066367	SCARF1	ENDOTHELIAL CELLS SCAVENGER RECEPTOR PRECURSOR.			X
IPI00067920	FXYD7	FXYD DOMAIN-CONTAINING ION TRANSPORT REGULATOR 7.			X
IPI00070070	FLJ32784	NOVEL PROTEIN.			X
IPI00072377	SET	ISOFORM 1 OF PROTEIN SET.			X
IPI00073769	PALM2	48 KDA PROTEIN.			X
IPI00074656	C6orf65	ISOFORM 1 OF COILED-COIL DOMAIN-CONTAINING PROTEIN C6ORF65.			X
IPI00084582	COL13A1	ISOFORM 7 OF COLLAGEN ALPHA-1(XIII) CHAIN.			X
IPI00084684	ZNF469	SIMILAR TO ZINC FINGER PROTEIN 469.			X
IPI00094740	RNF31	ISOFORM 1 OF RING FINGER PROTEIN 31.			X
IPI00099977	PDGFC	PLATELET-DERIVED GROWTH FACTOR C PRECURSOR.			X
IPI00100067	TNFRSF19	ISOFORM 1 OF TUMOR NECROSIS FACTOR RECEPTOR SUPERFAMILY MEMBER 19 PRECURSOR.			X
IPI00100106	RIC8A	RESISTANCE TO INHIBITORS OF CHOLINESTERASE 8 HOMOLOG A.			X
IPI00100247	TXNDC13	THIOREDOXIN DOMAIN-CONTAINING PROTEIN 13 PRECURSOR.			X
IPI00100362	EPB41L4B	ERYTHROCYTE MEMBRANE PROTEIN BAND 4.1 LIKE 4B ISOFORM 1.			X
IPI00100787	RGPD6; RGPD7	RANBP2-LIKE AND GRIP DOMAIN-CONTAINING PROTEIN 7.			X
IPI00100867	TAF1L	TRANSCRIPTION INITIATION FACTOR TFIID 210 KDA SUBUNIT.			X
IPI00101186	RRP12	ISOFORM 1 OF RRP12-LIKE PROTEIN.			X
IPI00101267	C19orf60	ISOFORM 1 OF UNCHARACTERIZED PROTEIN C19ORF60.			X
IPI00101299	C1GALT1C1	C1GALT1-SPECIFIC CHAPERONE 1.			X
IPI00101405	FDPS	FARNESYL DIPHOSPHATE SYNTHASE.			X
IPI00101987	C19orf62	UNCHARACTERIZED PROTEIN C19ORF62.			X
IPI00102281	SASP	RETROVIRAL-LIKE ASPARTIC PROTEASE 1 PRECURSOR.			X
IPI00102803	ANKRD22	ANKYRIN REPEAT DOMAIN-CONTAINING PROTEIN 22.			X
IPI00102820	MTUS1	ISOFORM 3 OF MITOCHONDRIAL TUMOR SUPPRESSOR 1.			X
IPI00103055	SLC35F5	ISOFORM 1 OF SOLUTE CARRIER FAMILY 35 MEMBER F5.			X
IPI00103090	LEO1	ISOFORM 1 OF RNA POLYMERASE-ASSOCIATED PROTEIN LEO1.			X
IPI00103142	NUDCD2	NUDC DOMAIN-CONTAINING PROTEIN 2.			X

Table S1. Proteins Identified or Not in MS Patients Compared to Normals and Other Neurologic Disease (OND)					
**IPI**	**Gene**	**Protein Name**	**MS**	**Normal**	**OND**
IPI00103146	PDLIM5	PDZ AND LIM DOMAIN 5 ISOFORM E.			X
IPI00103373	CYGB	CYTOGLOBIN.			X
IPI00103426	SLC13A3	ISOFORM 1 OF SOLUTE CARRIER FAMILY 13 MEMBER 3.			X
IPI00103595	CEP350	CENTROSOME-ASSOCIATED PROTEIN 350.			X
IPI00103604	CACNG8	VOLTAGE-DEPENDENT CALCIUM CHANNEL GAMMA-8 SUBUNIT.			X
IPI00103749	TREM2	ISOFORM 2 OF TRIGGERING RECEPTOR EXPRESSED ON MYELOID CELLS 2 PRECURSOR.			X
IPI00103812	PWWP2B	PWWP DOMAIN-CONTAINING PROTEIN 2B.			X
IPI00104050	THRAP3	THYROID HORMONE RECEPTOR-ASSOCIATED PROTEIN 3.			X
IPI00106491	MRTO4	MRNA TURNOVER PROTEIN 4 HOMOLOG.			X
IPI00107855	HEPH	HEPHAESTIN PRECURSOR.			X
IPI00141933	BUB1B	MITOTIC CHECKPOINT SERINE/THREONINE-PROTEIN KINASE BUB1 BETA.			X
IPI00142538	SETX	ISOFORM 1 OF PROBABLE HELICASE SENATAXIN.			X
IPI00145121	PIGB	GPI MANNOSYLTRANSFERASE 3.			X
IPI00148768	TRIOBP	TRIO AND F-ACTIN BINDING PROTEIN ISOFORM 1.			X
IPI00151141	WNK4	ISOFORM 1 OF SERINE/THREONINE-PROTEIN KINASE WNK4.			X
IPI00151366	TRPM6	ISOFORM TRPM6A OF TRANSIENT RECEPTOR POTENTIAL CATION CHANNEL SUBFAMILY M MEMBER 6.			X
IPI00152011	ARHGAP29	PTPL1-ASSOCIATED RHOGAP 1.			X
IPI00152149		CENP-B, N-TERMINAL DNA-BINDING DOMAIN CONTAINING PROTEIN.			X
IPI00152157	ZNF509	ISOFORM 1 OF ZINC FINGER PROTEIN 509.			X
IPI00152189		PUTATIVE UNCHARACTERIZED PROTEIN.			X
IPI00152432	GPT2	ISOFORM 1 OF ALANINE AMINOTRANSFERASE 2.			X
IPI00152536	TMC2	ISOFORM 1 OF TRANSMEMBRANE CHANNEL-LIKE PROTEIN 2.			X
IPI00152722	CCDC35	PROTEIN CCDC35.			X
IPI00152733	OLFML2A	FLJ00237 PROTEIN (FRAGMENT).			X
IPI00152853	KIAA1949	ISOFORM 1 OF PHOSTENSIN.			X
IPI00152879	SLC16A10	MONOCARBOXYLATE TRANSPORTER 10.			X
IPI00152960	RRN3	RNA POLYMERASE I-SPECIFIC TRANSCRIPTION INITIATION FACTOR RRN3.			X
IPI00152975	CCDC90A	ISOFORM 1 OF COILED-COIL DOMAIN-CONTAINING PROTEIN 90A, MITOCHONDRIAL PRECURSOR.			X
IPI00153031	C17orf80	ISOFORM 1 OF UNCHARACTERIZED PROTEIN C17ORF80.			X
IPI00153053	CALR3	CALRETICULIN-3 PRECURSOR.			X
IPI00153060	ANGPTL4	ANGIOPOIETIN-RELATED PROTEIN 4 PRECURSOR.			X

Table S1. Proteins Identified or Not in MS Patients Compared to Normals and Other Neurologic Disease (OND)					
**IPI**	**Gene**	**Protein Name**	**MS**	**Normal**	**OND**
IPI00154451	MMS19	MMS19 NUCLEOTIDE EXCISION REPAIR HOMOLOG.			X
IPI00154553	CEP76	CENTROSOMAL PROTEIN OF 76 KDA.			X
IPI00154567	SPATA20	ISOFORM 2 OF SPERMATOGENESIS-ASSOCIATED PROTEIN 20 PRECURSOR.			X
IPI00154834	NOPE	ISOFORM 1 OF NEIGHBOR OF PUNC E11 PRECURSOR.			X
IPI00155168	PTPRC	LEUKOCYTE COMMON ANTIGEN PRECURSOR (EC 3.1.3.48) (L-CA) (T200) (CD45 ANTIGEN). ISOFORM 2.			X
IPI00156804	ZNF665	CDNA FLJ77550, HIGHLY SIMILAR TO HOMO SAPIENS ZINC FINGER PROTEIN 665 (ZNF665), MRNA.			X
IPI00156871	USP49	ISOFORM 2 OF UBIQUITIN CARBOXYL-TERMINAL HYDROLASE 49.			X
IPI00157237	MPP4	ISOFORM 1 OF MAGUK P55 SUBFAMILY MEMBER 4.			X
IPI00157820	TXNRD2	THIOREDOXIN REDUCTASE 2 ISOFORM 1 VARIANT.			X
IPI00158506	ZNF688	ISOFORM 3 OF ZINC FINGER PROTEIN 688.			X
IPI00158804	BIVM	BASIC, IMMUNOGLOBULIN-LIKE VARIABLE MOTIF CONTAINING.			X
IPI00159652	FRYL	ISOFORM 2 OF PROTEIN FURRY HOMOLOG-LIKE.			X
IPI00161055	PRHOXNB	PUTATIVE 2-OXO-4-HYDROXY-4-CARBOXY-5-UREIDOIMIDAZOLINE DECARBOXYLASE.			X
IPI00163718	VPS54	ISOFORM 4 OF VACUOLAR PROTEIN SORTING-ASSOCIATED PROTEIN 54.			X
IPI00165357	MTA3	ISOFORM 1 OF METASTASIS-ASSOCIATED PROTEIN MTA3.			X
IPI00165591	TMC6	ISOFORM 4 OF TRANSMEMBRANE CHANNEL-LIKE PROTEIN 6.			X
IPI00165955	MAPK15	ISOFORM 1 OF MITOGEN-ACTIVATED PROTEIN KINASE 15.			X
IPI00165990	RFX4	ISOFORM 4 OF TRANSCRIPTION FACTOR RFX4.			X
IPI00166043	PAQR6	ISOFORM 2 OF PROGESTIN AND ADIPOQ RECEPTOR FAMILY MEMBER 6.			X
IPI00166044	KIAA1303	ISOFORM 1 OF REGULATORY-ASSOCIATED PROTEIN OF MTOR.			X
IPI00166153	KIAA0082	PROTEIN KIAA0082.			X
IPI00166190	C19orf19	ISOFORM 1 OF UNCHARACTERIZED PROTEIN C19ORF19.			X
IPI00166323	LOC152485	LOC152485 PROTEIN.			X
IPI00166331	ANKRD35	ANKYRIN REPEAT DOMAIN-CONTAINING PROTEIN 35.			X
IPI00166361	FLJ33590	PUTATIVE UNCHARACTERIZED PROTEIN FLJ33590.			X
IPI00166414	CCDC96	COILED-COIL DOMAIN-CONTAINING PROTEIN 96.			X
IPI00166465	C6orf223	HYPOTHETICAL PROTEIN LOC221416.			X
IPI00166528	RICTOR	ISOFORM 3 OF RAPAMYCIN-INSENSITIVE COMPANION OF MTOR.			X
IPI00166606	KIAA0913	KIAA0913 PROTEIN (FRAGMENT).			X
IPI00166738	ZADH2	ZINC-BINDING ALCOHOL DEHYDROGENASE DOMAIN-CONTAINING PROTEIN 2.			X
IPI00166833	LASS5	LAG1 LONGEVITY ASSURANCE HOMOLOG 5.			X

Table S1. Proteins Identified or Not in MS Patients Compared to Normals and Other Neurologic Disease (OND)					
**IPI**	**Gene**	**Protein Name**	**MS**	**Normal**	**OND**
IPI00166863	CCDC123	ISOFORM 2 OF COILED-COIL DOMAIN-CONTAINING PROTEIN 123, MITOCHONDRIAL PRECURSOR.			X
IPI00166933	C10orf12	UNCHARACTERIZED PROTEIN C10ORF12.			X
IPI00167014	EID2	EID2 PROTEIN (FRAGMENT).			X
IPI00167194	FLJ25694	CDNA FLJ25694 FIS, CLONE TST04471.			X
IPI00167228	CHCHD7	CDNA FLJ40966 FIS, CLONE UTERU2012407.			X
IPI00167241	ZNF283	ZINC FINGER PROTEIN 283.			X
IPI00167280	FLJ25439	CDNA FLJ40447 FIS, CLONE TESTI2040642.			X
IPI00167419	ANKRD44	ISOFORM 2 OF ANKYRIN REPEAT DOMAIN-CONTAINING PROTEIN 44.			X
IPI00167433	FDX1L	CDNA FLJ39703 FIS, CLONE SMINT2012195.			X
IPI00167446	C17orf78	ISOFORM 2 OF UNCHARACTERIZED PROTEIN C17ORF78.			X
IPI00167639	ATP6V0D2	VACUOLAR PROTON PUMP SUBUNIT D 2.			X
IPI00167762	C19orf47	ISOFORM 1 OF UNCHARACTERIZED PROTEIN C19ORF47.			X
IPI00167764	TMEM102	TRANSMEMBRANE PROTEIN 102.			X
IPI00167861	SMG5	PROTEIN SMG5.			X
IPI00167913	SPERT	ISOFORM 1 OF SPERMATID-ASSOCIATED PROTEIN.			X
IPI00167930	MARCH10	RING FINGER PROTEIN 190.			X
IPI00168056	ZBTB38	ZINC FINGER AND BTB DOMAIN-CONTAINING PROTEIN 38.			X
IPI00168255	LOC400451	HYPOTHETICAL PROTEIN LOC400451.			X
IPI00168607	TTC21A	TETRATRICOPEPTIDE REPEAT DOMAIN 21A ISOFORM 1.			X
IPI00168627	CXorf20	UNCHARACTERIZED PROTEIN CXORF20.			X
IPI00168848	B3GNT7	UDP-GLCNAC:BETAGAL BETA-1,3-N-ACETYLGLUCOSAMINYLTRANSFERASE 7.			X
IPI00168877	HELB	HELICASE (DNA) B.			X
IPI00168913	EVC2	ISOFORM 2 OF LIMBIN.			X
IPI00168977		OLFACTORY RECEPTOR 6J1.			X
IPI00168995	OR4D9	OLFACTORY RECEPTOR 4D9.			X
IPI00169253	OR5AT1	OLFACTORY RECEPTOR OR1-45.			X
IPI00169276		TRYPSINOGEN C.			X
IPI00169377	GAS2L2	ISOFORM 1 OF GAS2-LIKE PROTEIN 2.			X
IPI00169430	STRBP	ISOFORM 1 OF SPERMATID PERINUCLEAR RNA-BINDING PROTEIN.			X
IPI00170509	CAMKK2	ISOFORM 3 OF CALCIUM/CALMODULIN-DEPENDENT PROTEIN KINASE KINASE 2.			X
IPI00170548	ATAD2	ISOFORM 1 OF ATPASE FAMILY AAA DOMAIN-CONTAINING PROTEIN 2.			X
IPI00170765	AGXT2L2	ISOFORM 3 OF ALANINE--GLYOXYLATE AMINOTRANSFERASE 2-LIKE 2.			X
IPI00170770	PHF3	ISOFORM 1 OF PHD FINGER PROTEIN 3.			X

Table S1. Proteins Identified or Not in MS Patients Compared to Normals and Other Neurologic Disease (OND)					
**IPI**	**Gene**	**Protein Name**	**MS**	**Normal**	**OND**
IPI00170800	OTOA	ISOFORM 1 OF OTOANCORIN PRECURSOR.			X
IPI00170855	TTC19	TETRATRICOPEPTIDE REPEAT DOMAIN 19.			X
IPI00170961	WDR16	ISOFORM 2 OF WD REPEAT-CONTAINING PROTEIN 16.			X
IPI00171160	LRRC17	ISOFORM 1 OF LEUCINE-RICH REPEAT-CONTAINING PROTEIN 17 PRECURSOR.			X
IPI00171500	C14orf45	UNCHARACTERIZED PROTEIN C14ORF45.			X
IPI00171525	SENP3	SENTRIN-SPECIFIC PROTEASE 3.			X
IPI00171716	TTC28	OTTHUMP00000028696.			X
IPI00175169	ARFGAP1	ISOFORM 1 OF ADP-RIBOSYLATION FACTOR GTPASE-ACTIVATING PROTEIN 1.			X
IPI00175193	KIF4B	CHROMOSOME-ASSOCIATED KINESIN KIF4B.			X
IPI00175439	FBXO43	F-BOX ONLY PROTEIN 43.			X
IPI00175649	LRRK2	LEUCINE-RICH REPEAT SERINE/THREONINE-PROTEIN KINASE 2.			X
IPI00176125	COL29A1	COLLAGEN XXIX ALPHA 1.			X
IPI00176574	LOC284230	SIMILAR TO LARGE SUBUNIT RIBOSOMAL PROTEIN L36A.			X
IPI00176778	KIAA1841	KIAA1841 PROTEIN.			X
IPI00176976	RP1-57A13.2	NOVEL PROTEIN SIMILAR TO MITOCHONDRIAL RIBOSOME RECYCLING FACTOR ISOFORM 1.			X
IPI00178150	KIF4A	ISOFORM 1 OF CHROMOSOME-ASSOCIATED KINESIN KIF4A.			X
IPI00179172	PPFIBP1	ISOFORM 2 OF LIPRIN-BETA-1.			X
IPI00179415	PPP3CA	ISOFORM 1 OF SERINE/THREONINE-PROTEIN PHOSPHATASE 2B CATALYTIC SUBUNIT ALPHA ISOFORM.			X
IPI00179452	CBFA2T2	ISOFORM 1 OF PROTEIN CBFA2T2.			X
IPI00179694	KLB	BETA-KLOTHO.			X
IPI00180305	UBR4	ISOFORM 5 OF E3 UBIQUITIN-PROTEIN LIGASE UBR4.			X
IPI00180375	ARRB2	ISOFORM 1 OF BETA-ARRESTIN-2.			X
IPI00180781	MLKL	ISOFORM 1 OF MIXED LINEAGE KINASE DOMAIN-LIKE PROTEIN.			X
IPI00181279	LOC728863; LRRC37A4	HYPOTHETICAL PROTEIN.			X
IPI00182774	SH3YL1	SH3YL1 PROTEIN.			X
IPI00182833	CCHCR1	COILED-COIL ALPHA-HELICAL ROD PROTEIN 1.			X
IPI00183425	CIZ1	CDKN1A INTERACTING ZINC FINGER PROTEIN 1.			X
IPI00183572	DOCK7	ISOFORM 2 OF DEDICATOR OF CYTOKINESIS PROTEIN 7.			X
IPI00184160	RNF180	ISOFORM 2 OF RING FINGER PROTEIN 180.			X
IPI00185038	DUOX1	ISOFORM 1 OF DUAL OXIDASE 1 PRECURSOR.			X
IPI00185361	DDX55	ATP-DEPENDENT RNA HELICASE DDX55.			X

Table S1. Proteins Identified or Not in MS Patients Compared to Normals and Other Neurologic Disease (OND)					
**IPI**	**Gene**	**Protein Name**	**MS**	**Normal**	**OND**
IPI00185892	ABLIM3	ISOFORM 1 OF ACTIN-BINDING LIM PROTEIN 3.			X
IPI00186114	ADAMTS16	ISOFORM 2 OF ADAMTS-16 PRECURSOR.			X
IPI00186145	PPM1L	ISOFORM 1 OF PROTEIN PHOSPHATASE 1L.			X
IPI00186439	ZNF643	ZINC FINGER PROTEIN 643.			X
IPI00186554	KIAA1853	UNCHARACTERIZED PROTEIN KIAA1853.			X
IPI00186853	PCNXL2	PECANEX-LIKE 2.			X
IPI00187082	MCTP1	ISOFORM 3 OF MULTIPLE C2 AND TRANSMEMBRANE DOMAIN-CONTAINING PROTEIN 1.			X
IPI00187091	MKNK1	ISOFORM 2 OF MAP KINASE-INTERACTING SERINE/THREONINE-PROTEIN KINASE 1.			X
IPI00215786	PDE10A	3',5'-CYCLIC NUCLEOTIDE PHOSPHODIESTERASE 10A2.			X
IPI00215884	SFRS1	ISOFORM ASF-1 OF SPLICING FACTOR, ARGININE/SERINE-RICH 1.			X
IPI00215925	GNMT	GLYCINE N-METHYLTRANSFERASE.			X
IPI00215948	CTNNA1	ISOFORM 1 OF CATENIN ALPHA-1.			X
IPI00215965	HNRNPA1	ISOFORM A1-B OF HETEROGENEOUS NUCLEAR RIBONUCLEOPROTEIN A1.			X
IPI00216132	RPS6KB1	ISOFORM ALPHA I OF RIBOSOMAL PROTEIN S6 KINASE BETA-1.			X
IPI00216137	SYCP1	SYNAPTONEMAL COMPLEX PROTEIN 1.			X
IPI00216139	SEPT6	ISOFORM I OF SEPTIN-6.			X
IPI00216164	EHHADH	PEROXISOMAL BIFUNCTIONAL ENZYME.			X
IPI00216236	TNNI2	TROPONIN I, FAST SKELETAL MUSCLE.			X
IPI00216256	WDR1	ISOFORM 2 OF WD REPEAT-CONTAINING PROTEIN 1.			X
IPI00216308	VDAC1	VOLTAGE-DEPENDENT ANION-SELECTIVE CHANNEL PROTEIN 1.			X
IPI00216313	VSNL1	VISININ-LIKE PROTEIN 1.			X
IPI00216346	NOL3	ISOFORM 2 OF NUCLEOLAR PROTEIN 3.			X
IPI00216356	RASAL1	ISOFORM 2 OF RASGAP-ACTIVATING-LIKE PROTEIN 1.			X
IPI00216484	ARMC9	ISOFORM 2 OF LISH DOMAIN-CONTAINING PROTEIN ARMC9.			X
IPI00216544	HR	ISOFORM 2 OF PROTEIN HAIRLESS.			X
IPI00216593	NOX1	ISOFORM NOH-1LV OF NADPH OXIDASE HOMOLOG 1.			X
IPI00216702	ABCA9	ISOFORM 1 OF ATP-BINDING CASSETTE SUB-FAMILY A MEMBER 9.			X
IPI00216710	RORC	ISOFORM 1 OF NUCLEAR RECEPTOR ROR-GAMMA.			X
IPI00216815	TOP3B	ISOFORM 2 OF DNA TOPOISOMERASE 3-BETA-1.			X
IPI00216917	C22orf9	ISOFORM 1 OF UNCHARACTERIZED PROTEIN C22ORF9.			X
IPI00216969	ABL1	ISOFORM IA OF PROTO-ONCOGENE TYROSINE-PROTEIN KINASE ABL1.			X
IPI00216986	CR2	ISOFORM C OF COMPLEMENT RECEPTOR TYPE 2 PRECURSOR.			X
IPI00217051	NAV3	ISOFORM 1 OF NEURON NAVIGATOR 3.			X

Table S1. Proteins Identified or Not in MS Patients Compared to Normals and Other Neurologic Disease (OND)					
**IPI**	**Gene**	**Protein Name**	**MS**	**Normal**	**OND**
IPI00217117	GLYCTK	ISOFORM 1 OF GLYCERATE KINASE.			X
IPI00217418	ARHGAP12	ISOFORM 2 OF RHO GTPASE-ACTIVATING PROTEIN 12.			X
IPI00217442	ANKHD1-EIF4EBP3; EIF4EBP3	ANKHD1-EIF4EBP3 PROTEIN.			X
IPI00217490	FNDC3B	ISOFORM 1 OF FIBRONECTIN TYPE III DOMAIN-CONTAINING PROTEIN 3B.			X
IPI00217494	SMG7	ISOFORM 1 OF PROTEIN SMG7.			X
IPI00217662	C11orf35	UNCHARACTERIZED PROTEIN C11ORF35.			X
IPI00217687	CCDC135	ISOFORM 1 OF COILED-COIL DOMAIN-CONTAINING PROTEIN 135.			X
IPI00217850	GRAMD1C	ISOFORM 1 OF GRAM DOMAIN-CONTAINING PROTEIN 1C.			X
IPI00217895	SHC4	RAI-LIKE PROTEIN.			X
IPI00217899	RNF168	RING FINGER PROTEIN 168.			X
IPI00217915	TMEM136	ISOFORM 1 OF TRANSMEMBRANE PROTEIN 136.			X
IPI00217955	H1FOO	H1 HISTONE FAMILY, MEMBER O, OOCYTE-SPECIFIC.			X
IPI00217975	LMNB1	LAMIN-B1.			X
IPI00217979	MLL5	MYELOID/LYMPHOID OR MIXED-LINEAGE LEUKEMIA 5.			X
IPI00218235	DHRS2	DEHYDROGENASE/REDUCTASE MEMBER 2 ISOFORM 2.			X
IPI00218271	MPP2	ISOFORM 3 OF MAGUK P55 SUBFAMILY MEMBER 2.			X
IPI00218559	SGCG	GAMMA-SARCOGLYCAN.			X
IPI00218687	CACNA1G	ISOFORM 10 OF VOLTAGE-DEPENDENT T-TYPE CALCIUM CHANNEL SUBUNIT ALPHA-1G.			X
IPI00218715	ADAM33	ISOFORM 2 OF ADAM 33 PRECURSOR.			X
IPI00218743	BEST1	ISOFORM 2 OF BESTROPHIN-1.			X
IPI00218823	MLL4	ISOFORM 1 OF WW DOMAIN-BINDING PROTEIN 7.			X
IPI00218839	CYP1A1	CYTOCHROME P450 1A1.			X
IPI00218896	ADH1A	ALCOHOL DEHYDROGENASE 1A.			X
IPI00219036	DICER1	DICER1.			X
IPI00219503	FBXO24	ISOFORM 1 OF F-BOX ONLY PROTEIN 24.			X
IPI00219563	PLCB1	ISOFORM A OF 1-PHOSPHATIDYLINOSITOL-4,5-BISPHOSPHATE PHOSPHODIESTERASE BETA-1.			X
IPI00219604	MAP2K1	DUAL SPECIFICITY MITOGEN-ACTIVATED PROTEIN KINASE KINASE 1.			X
IPI00219661	PLP1	ISOFORM 1 OF MYELIN PROTEOLIPID PROTEIN.			X
IPI00219689	VAV3	ISOFORM BETA OF PROTEIN VAV-3.			X
IPI00219703	PVALB	PARVALBUMIN ALPHA.			X

Table S1. Proteins Identified or Not in MS Patients Compared to Normals and Other Neurologic Disease (OND)					
**IPI**	**Gene**	**Protein Name**	**MS**	**Normal**	**OND**
IPI00219817	MAPK8IP3	ISOFORM 2 OF C-JUN-AMINO-TERMINAL KINASE-INTERACTING PROTEIN 3.			X
IPI00219898	NPHS1; PRODH2	ISOFORM 2 OF NEPHRIN PRECURSOR.			X
IPI00219953	CMPK1	CYTIDINE MONOPHOSPHATE (UMP-CMP) KINASE 1, CYTOSOLIC.			X
IPI00220002	PALM	ISOFORM 2 OF PARALEMMIN.			X
IPI00220038	ARS2	ISOFORM B OF ARSENITE-RESISTANCE PROTEIN 2.			X
IPI00220039	TMEM16K	ISOFORM 1 OF TRANSMEMBRANE PROTEIN 16K.			X
IPI00220113	MAP4	ISOFORM 2 OF MICROTUBULE-ASSOCIATED PROTEIN 4.			X
IPI00220126	SPAG11B	ISOFORM EP2B OF SPERM-ASSOCIATED ANTIGEN 11B PRECURSOR.			X
IPI00220167	NSUN7	ISOFORM 1 OF PUTATIVE METHYLTRANSFERASE NSUN7.			X
IPI00220257	TTLL1	ISOFORM 3 OF PROBABLE TUBULIN POLYGLUTAMYLASE.			X
IPI00220308	NF2	ISOFORM 2 OF MERLIN.			X
IPI00220325	INSR	ISOFORM SHORT OF INSULIN RECEPTOR PRECURSOR.			X
IPI00220412	S100A1	S100 CALCIUM BINDING PROTEIN A1.			X
IPI00220473	ATP2C1	ISOFORM 2 OF CALCIUM-TRANSPORTING ATPASE TYPE 2C MEMBER 1.			X
IPI00220493	LSM5	U6 SNRNA-ASSOCIATED SM-LIKE PROTEIN LSM5.			X
IPI00220503	DCTN2	DYNACTIN 2.			X
IPI00220533	TMPRSS3	ISOFORM D OF TRANSMEMBRANE PROTEASE, SERINE 3.			X
IPI00220736	NEU3	SIALIDASE 3.			X
IPI00220901	TBC1D4	TBC1D4 PROTEIN.			X
IPI00220986	ADAMTS9	ISOFORM 3 OF ADAMTS-9 PRECURSOR.			X
IPI00221009	TCF7L2	ISOFORM 7 OF TRANSCRIPTION FACTOR 7-LIKE 2.			X
IPI00221101	SI	SUCRASE-ISOMALTASE, INTESTINAL.			X
IPI00221240	LNPEP	ISOFORM 2 OF LEUCYL-CYSTINYL AMINOPEPTIDASE.			X
IPI00221325	RANBP2	E3 SUMO-PROTEIN LIGASE RANBP2.			X
IPI00221372	AGPAT2	ISOFORM 2 OF 1-ACYL-SN-GLYCEROL-3-PHOSPHATE ACYLTRANSFERASE BETA.			X
IPI00232571	GPC4	GLYPICAN-4 PRECURSOR.			X
IPI00232891	FNDC1	ISOFORM 3 OF FIBRONECTIN TYPE III DOMAIN-CONTAINING PROTEIN 1.			X
IPI00235003	FAS	TUMOR NECROSIS FACTOR RECEPTOR SUPERFAMILY, MEMBER 6 ISOFORM 1 VARIANT (FRAGMENT).			X
IPI00235167	PFN3	PROFILIN-3.			X
IPI00235622	CDCP1	ISOFORM 3 OF CUB DOMAIN-CONTAINING PROTEIN 1 PRECURSOR.			X
IPI00235842	ZNF483	ZINC FINGER PROTEIN 483.			X
IPI00237884	AKAP12	ISOFORM 1 OF A-KINASE ANCHOR PROTEIN 12.			X

Table S1. Proteins Identified or Not in MS Patients Compared to Normals and Other Neurologic Disease (OND)					
**IPI**	**Gene**	**Protein Name**	**MS**	**Normal**	**OND**
IPI00240059	TMCC3	TRANSMEMBRANE AND COILED-COIL DOMAINS PROTEIN 3.			X
IPI00240345	CLEC14A	C-TYPE LECTIN DOMAIN FAMILY 14 MEMBER A PRECURSOR.			X
IPI00240909	hCG_15200	SIMILAR TO EUKARYOTIC TRANSLATION INITIATION FACTOR 3, SUBUNIT 5 EPSILON, 47KDA ISOFORM 1.			X
IPI00244346	TNNI3	TROPONIN I, CARDIAC MUSCLE.			X
IPI00244812	TTC6	TETRATRICOPEPTIDE REPEAT PROTEIN 6.			X
IPI00247063	MME	NEPRILYSIN.			X
IPI00250716	BRWD1	ISOFORM B OF BROMODOMAIN AND WD REPEAT-CONTAINING PROTEIN 1.			X
IPI00253323	ANKRD57	ANKYRIN REPEAT DOMAIN-CONTAINING PROTEIN 57.			X
IPI00254408	BPTF	BROMODOMAIN PHD FINGER TRANSCRIPTION FACTOR ISOFORM 1.			X
IPI00259671	TAS2R44	TASTE RECEPTOR TYPE 2 MEMBER 44.			X
IPI00288939	CCDC40	ISOFORM 3 OF COILED-COIL DOMAIN-CONTAINING PROTEIN 40.			X
IPI00289097	RASGEF1B	ISOFORM 1 OF RAS-GEF DOMAIN-CONTAINING FAMILY MEMBER 1B.			X
IPI00289914	MSH4	MUTS PROTEIN HOMOLOG 4.			X
IPI00289931	MADCAM1	ISOFORM 1 OF MUCOSAL ADDRESSIN CELL ADHESION MOLECULE 1 PRECURSOR.			X
IPI00289954	HS3ST6	HEPARAN SULFATE GLUCOSAMINE 3-O-SULFOTRANSFERASE 6.			X
IPI00290155	ZNF148	66 KDA PROTEIN.			X
IPI00290432	SLC11A1	NATURAL RESISTANCE-ASSOCIATED MACROPHAGE PROTEIN 1.			X
IPI00290562	NFIL3	NUCLEAR FACTOR INTERLEUKIN-3-REGULATED PROTEIN.			X
IPI00290785	SETD6	ISOFORM 1 OF SET DOMAIN-CONTAINING PROTEIN 6.			X
IPI00290837	FBXW10	ISOFORM 2 OF F-BOX/WD REPEAT-CONTAINING PROTEIN 10.			X
IPI00290903	C2orf65	CHROMOSOME 2 OPEN READING FRAME 65.			X
IPI00291662	LONRF1	LON PEPTIDASE N-TERMINAL DOMAIN AND RING FINGER 1.			X
IPI00291792	ITGB2	INTEGRIN BETA-2 PRECURSOR.			X
IPI00291839	RAPGEF6	ISOFORM 2 OF RAP GUANINE NUCLEOTIDE EXCHANGE FACTOR 6.			X
IPI00291840	ZFP90	ISOFORM 1 OF ZINC FINGER PROTEIN 90 HOMOLOG.			X
IPI00292086	CSNK1A1	HLCDGP1.			X
IPI00292130	DPT	DERMATOPONTIN PRECURSOR.			X
IPI00292134	EPS15	EPIDERMAL GROWTH FACTOR RECEPTOR SUBSTRATE 15.			X
IPI00292470	DACT2	DAPPER, ANTAGONIST OF BETA-CATENIN, HOMOLOG 2.			X
IPI00292471	CENTD1	CENTAURIN-DELTA-1.			X
IPI00292579	STAB2	STABILIN-2 PRECURSOR.			X
IPI00292894	TSR1	PRE-RRNA-PROCESSING PROTEIN TSR1 HOMOLOG.			X
IPI00292953	RAI14	ISOFORM 2 OF ANKYCORBIN.			X

Table S1. Proteins Identified or Not in MS Patients Compared to Normals and Other Neurologic Disease (OND)					
**IPI**	**Gene**	**Protein Name**	**MS**	**Normal**	**OND**
IPI00293409	ZNF81	ZINC FINGER PROTEIN 81.			X
IPI00293431	MGC70863	WUGSC:H_DJ0855D21.2 PROTEIN.			X
IPI00293565	FLT4	FMS-RELATED TYROSINE KINASE 4 ISOFORM 1.			X
IPI00293613	TBK1	SERINE/THREONINE-PROTEIN KINASE TBK1.			X
IPI00293616	DDX3Y	ATP-DEPENDENT RNA HELICASE DDX3Y.			X
IPI00293857	ARRB1	ISOFORM 1A OF BETA-ARRESTIN-1.			X
IPI00293867	DDT	D-DOPACHROME DECARBOXYLASE.			X
IPI00294187	PADI2	PROTEIN-ARGININE DEIMINASE TYPE-2.			X
IPI00294241	GTF3A	TRANSCRIPTION FACTOR IIIA.			X
IPI00294386	MYO1A	MYOSIN-IA.			X
IPI00294398	HADH	ISOFORM 1 OF HYDROXYACYL-COENZYME A DEHYDROGENASE, MITOCHONDRIAL PRECURSOR.			X
IPI00294618	PRCC	PROLINE-RICH PROTEIN PRCC.			X
IPI00294728	DMXL1	DMX-LIKE PROTEIN 1.			X
IPI00294810	FAM21A	CDNA FLJ10824 FIS, CLONE NT2RP4001086 (FRAGMENT).			X
IPI00295253	CD200R1	ISOFORM 1 OF CELL SURFACE GLYCOPROTEIN OX2 RECEPTOR PRECURSOR.			X
IPI00295400	WARS	TRYPTOPHANYL-TRNA SYNTHETASE, CYTOPLASMIC.			X
IPI00295461	FAP	ISOFORM 1 OF SEPRASE.			X
IPI00295519	C3orf17	ISOFORM 1 OF UNCHARACTERIZED PROTEIN C3ORF17.			X
IPI00295601	CEND1	CELL CYCLE EXIT AND NEURONAL DIFFERENTIATION PROTEIN 1.			X
IPI00295999	COL9A1	ISOFORM 2 OF COLLAGEN ALPHA-1(IX) CHAIN PRECURSOR.			X
IPI00296063	SCN11A	ISOFORM 1 OF SODIUM CHANNEL PROTEIN TYPE 11 SUBUNIT ALPHA.			X
IPI00296196	DMGDH	DIMETHYLGLYCINE DEHYDROGENASE, MITOCHONDRIAL PRECURSOR.			X
IPI00296286	TMEFF2	ISOFORM 1 OF TOMOREGULIN-2 PRECURSOR.			X
IPI00296421	EHBP1L1	EH DOMAIN-BINDING PROTEIN 1-LIKE PROTEIN 1.			X
IPI00296434	SLIT1	ISOFORM 1 OF SLIT HOMOLOG 1 PROTEIN PRECURSOR.			X
IPI00296535	HACL1	2-HYDROXYACYL-COA LYASE 1.			X
IPI00296563	GUF1	GTP-BINDING PROTEIN GUF1 HOMOLOG.			X
IPI00296803	TCP11L2	30 KDA PROTEIN.			X
IPI00296942	CDH12	CADHERIN-12 PRECURSOR.			X
IPI00297212	KIAA1274	PALADIN.			X
IPI00297235	CCPG1	CDNA FLJ77204.			X
IPI00297407	SLC45A4	SOLUTE CARRIER FAMILY 45 MEMBER 4.			X
IPI00297462	C1orf65	UNCHARACTERIZED PROTEIN C1ORF65.			X

Table S1. Proteins Identified or Not in MS Patients Compared to Normals and Other Neurologic Disease (OND)					
**IPI**	**Gene**	**Protein Name**	**MS**	**Normal**	**OND**
IPI00297559	USHBP1	ISOFORM 1 OF USH1C-BINDING PROTEIN 1.			X
IPI00297763	ABCA4	RETINAL-SPECIFIC ATP-BINDING CASSETTE TRANSPORTER.			X
IPI00297767	CSNK1G2	CASEIN KINASE I ISOFORM GAMMA-2.			X
IPI00297859	MLL2	ISOFORM 1 OF MYELOID/LYMPHOID OR MIXED-LINEAGE LEUKEMIA PROTEIN 2.			X
IPI00297931	AP1GBP1	ISOFORM 1 OF AP1 SUBUNIT GAMMA-BINDING PROTEIN 1.			X
IPI00297985	C6orf103	UNCHARACTERIZED PROTEIN C6ORF103.			X
IPI00298058	SUPT5H	ISOFORM 1 OF TRANSCRIPTION ELONGATION FACTOR SPT5.			X
IPI00298214	ABCG2	ISOFORM 1 OF ATP-BINDING CASSETTE SUB-FAMILY G MEMBER 2.			X
IPI00298447	TARBP1	PROBABLE METHYLTRANSFERASE TARBP1.			X
IPI00298870	TMEM1	TRANSMEMBRANE PROTEIN 1.			X
IPI00299040	PKD2	POLYCYSTIN-2.			X
IPI00299084	TMEM33	TRANSMEMBRANE PROTEIN 33.			X
IPI00299301	DMN	ISOFORM 1 OF DESMUSLIN.			X
IPI00299402	PC	PYRUVATE CARBOXYLASE, MITOCHONDRIAL PRECURSOR.			X
IPI00299406	ADCYAP1R1	PITUITARY ADENYLATE CYCLASE-ACTIVATING POLYPEPTIDE TYPE I RECEPTOR PRECURSOR.			X
IPI00299507	NCAPH	CONDENSIN COMPLEX SUBUNIT 2.			X
IPI00299554	KIF14	KINESIN-LIKE PROTEIN KIF14.			X
IPI00299729	TCN1	TRANSCOBALAMIN-1 PRECURSOR.			X
IPI00299853	PCDHB16	PROTOCADHERIN BETA 16 PRECURSOR.			X
IPI00299880	KCNMB3	ISOFORM 2 OF CALCIUM-ACTIVATED POTASSIUM CHANNEL SUBUNIT BETA-3.			X
IPI00300046	PSG4	SIMILAR TO PREGNANCY-SPECIFIC BETA 1 GLYCOPROTEIN.			X
IPI00300053	KRT82	KERATIN TYPE II CUTICULAR HB2.			X
IPI00300060	WDR70	WD REPEAT-CONTAINING PROTEIN 70.			X
IPI00300086	QPRT	NICOTINATE-NUCLEOTIDE PYROPHOSPHORYLASE.			X
IPI00300221	ELL	PUTATIVE UNCHARACTERIZED PROTEIN DKFZP434I1916.			X
IPI00300426	TTC29	ISOFORM 1 OF TETRATRICOPEPTIDE REPEAT PROTEIN 29.			X
IPI00300594	SPATA13	CDNA FLJ35435 FIS, CLONE SMINT2002620.			X
IPI00300621	SYNJ2	ISOFORM 2B2 OF SYNAPTOJANIN-2.			X
IPI00301144	SUMF1	ISOFORM 1 OF SULFATASE-MODIFYING FACTOR 1 PRECURSOR.			X
IPI00301503	SFRS10	ISOFORM 1 OF SPLICING FACTOR, ARGININE/SERINE-RICH 10.			X
IPI00301583	ZNF691	ISOFORM 1 OF ZINC FINGER PROTEIN 691.			X
IPI00301609	NEK9	SERINE/THREONINE-PROTEIN KINASE NEK9.			X
IPI00301726	C3orf20	ISOFORM 1 OF UNCHARACTERIZED PROTEIN C3ORF20.			X

Table S1. Proteins Identified or Not in MS Patients Compared to Normals and Other Neurologic Disease (OND)					
**IPI**	**Gene**	**Protein Name**	**MS**	**Normal**	**OND**
IPI00301844	ZHX3	ZINC FINGERS AND HOMEOBOXES PROTEIN 3.			X
IPI00302176	NOLA1	ISOFORM 1 OF H/ACA RIBONUCLEOPROTEIN COMPLEX SUBUNIT 1.			X
IPI00302351	STK33	ISOFORM 1 OF SERINE/THREONINE-PROTEIN KINASE 33.			X
IPI00302458	XPO7	EXPORTIN-7.			X
IPI00302647	CC2D1A	ISOFORM 1 OF COILED-COIL AND C2 DOMAIN-CONTAINING PROTEIN 1A.			X
IPI00303195	SYNC1	SYNCOILIN-1.			X
IPI00303207	ABCE1	ATP-BINDING CASSETTE SUB-FAMILY E MEMBER 1.			X
IPI00303343	SCAF1	SPLICING FACTOR, ARGININE/SERINE-RICH 19.			X
IPI00303622	OR1N2	OLFACTORY RECEPTOR OR9-23.			X
IPI00303732	C20orf106	UNCHARACTERIZED PROTEIN C20ORF106 PRECURSOR.			X
IPI00303870		SEVEN TRANSMEMBRANE HELIX RECEPTOR.			X
IPI00303980	C14orf93	ISOFORM 1 OF UNCHARACTERIZED PROTEIN C14ORF93 PRECURSOR.			X
IPI00304324	HDAC11	HISTONE DEACETYLASE 11.			X
IPI00304587	DISP2	PROTEIN DISPATCHED HOMOLOG 2.			X
IPI00304589	TNKS1BP1	ISOFORM 1 OF 182 KDA TANKYRASE 1-BINDING PROTEIN.			X
IPI00304885	CENPC1	CENTROMERE PROTEIN C 1.			X
IPI00305477	CST1	CYSTATIN-SN PRECURSOR.			X
IPI00305692	TXNL1	THIOREDOXIN-LIKE PROTEIN 1.			X
IPI00305856	HOXD11	HOMEOBOX PROTEIN HOX-D11.			X
IPI00306127	THUMPD3	THUMP DOMAIN-CONTAINING PROTEIN 3.			X
IPI00306166	GSG1	GERM CELL ASSOCIATED 1 ISOFORM 2.			X
IPI00306343	RBM41	ISOFORM 1 OF RNA-BINDING PROTEIN 41.			X
IPI00306402	LOXL4	LYSYL OXIDASE HOMOLOG 4 PRECURSOR.			X
IPI00306611	TRAIP	TRAF-INTERACTING PROTEIN.			X
IPI00306749	SLC4A1AP	KANADAPTIN.			X
IPI00306929	MYO18B	ISOFORM 2 OF MYOSIN-XVIIIB.			X
IPI00307016	MT1P2	METALLOTHIONEIN 1 PSEUDOGENE 2.			X
IPI00307165	TRIM47	TRIPARTITE MOTIF-CONTAINING PROTEIN 47.			X
IPI00307259	DNAJC13	DNAJ HOMOLOG SUBFAMILY C MEMBER 13.			X
IPI00307536	TEX11	ISOFORM 1 OF TESTIS-EXPRESSED SEQUENCE 11 PROTEIN.			X
IPI00307733	SETD2	ISOFORM 1 OF HISTONE-LYSINE N-METHYLTRANSFERASE SETD2.			X
IPI00307757	PITPNM3	ISOFORM 1 OF MEMBRANE-ASSOCIATED PHOSPHATIDYLINOSITOL TRANSFER PROTEIN 3.			X
IPI00328089	HIVEP3	KAPPA B AND V(D)J RECOMBINATION SIGNAL SEQUENCES BINDING PROTEIN.			X

Table S1. Proteins Identified or Not in MS Patients Compared to Normals and Other Neurologic Disease (OND)					
**IPI**	**Gene**	**Protein Name**	**MS**	**Normal**	**OND**
IPI00328094	FOXP3	ISOFORM 1 OF FORKHEAD BOX PROTEIN P3.			X
IPI00328156	MAOB	AMINE OXIDASE [FLAVIN-CONTAINING] B.			X
IPI00328159	C1orf88	ISOFORM 1 OF UNCHARACTERIZED PROTEIN C1ORF88.			X
IPI00328178	TOM1L2	ISOFORM 3 OF TOM1-LIKE PROTEIN 2.			X
IPI00328225	TBX20	ISOFORM 1 OF T-BOX TRANSCRIPTION FACTOR TBX20.			X
IPI00328268	EIF4G3	EIF4G3 PROTEIN.			X
IPI00328291	CKAP2L	CYTOSKELETON-ASSOCIATED PROTEIN 2-LIKE.			X
IPI00328400	ZAR1	ZYGOTE ARREST PROTEIN 1.			X
IPI00328409	ARMC4	ARMADILLO REPEAT-CONTAINING PROTEIN 4.			X
IPI00328715	MTDH	PROTEIN LYRIC.			X
IPI00328748	ARMET	PROTEIN ARMET PRECURSOR.			X
IPI00328793	SREBF2	STEROL REGULATORY ELEMENT-BINDING PROTEIN 2.			X
IPI00328825	N4BP2	ISOFORM 1 OF NEDD4-BINDING PROTEIN 2.			X
IPI00328840	THOC4	THO COMPLEX SUBUNIT 4.			X
IPI00328842	ARHGAP4	ARHGAP4 PROTEIN.			X
IPI00328872	LOC339742	HYPOTHETICAL PROTEIN.			X
IPI00328885	DKFZp434B123 1	EEF1A2 BINDING PROTEIN.			X
IPI00329014	ANKRD46	ISOFORM 1 OF ANKYRIN REPEAT DOMAIN-CONTAINING PROTEIN 46.			X
IPI00329054	OSTM1	OSTEOPETROSIS-ASSOCIATED TRANSMEMBRANE PROTEIN 1 PRECURSOR.			X
IPI00329142	ERICH1	GLUTAMATE-RICH PROTEIN 1.			X
IPI00329251	TMEM146	ISOFORM 1 OF TRANSMEMBRANE PROTEIN 146 PRECURSOR.			X
IPI00329488	ABL2	ISOFORM IB OF TYROSINE-PROTEIN KINASE ABL2.			X
IPI00332106	PBXIP1	ISOFORM 1 OF PRE-B-CELL LEUKEMIA TRANSCRIPTION FACTOR-INTERACTING PROTEIN 1.			X
IPI00332583	CSH1; CSH2; GH1	CHORIONIC SOMATOMAMMOTROPIN HORMONE 1 ISOFORM 2.			X
IPI00332628	OTOP3	OTOPETRIN-3.			X
IPI00333016	DNAJC11	ISOFORM 3 OF DNAJ HOMOLOG SUBFAMILY C MEMBER 11.			X
IPI00333503		UNCHARACTERIZED PROTEIN ENSP00000315571 (FRAGMENT).			X
IPI00334845	KIAA1946	ISOFORM 3 OF UNCHARACTERIZED PROTEIN KIAA1946 PRECURSOR.			X
IPI00335259	DLC1	DELETED IN LIVER CANCER 1 ISOFORM 1.			X
IPI00335581	UBR3	ISOFORM 1 OF E3 UBIQUITIN-PROTEIN LIGASE UBR3.			X
IPI00337335	MYH14	ISOFORM 1 OF MYOSIN-14.			X

Table S1. Proteins Identified or Not in MS Patients Compared to Normals and Other Neurologic Disease (OND)					
**IPI**	**Gene**	**Protein Name**	**MS**	**Normal**	**OND**
IPI00337348	GDF7	GROWTH DIFFERENTIATION FACTOR 7.			X
IPI00337415	GNAI1	GUANINE NUCLEOTIDE-BINDING PROTEIN G(I), ALPHA-1 SUBUNIT.			X
IPI00339297	GALNT4	POLYPEPTIDE N-ACETYLGALACTOSAMINYLTRANSFERASE 4.			X
IPI00339309	MCF2	ISOFORM 1 OF PROTO-ONCOGENE DBL.			X
IPI00373824	TESSP1	TESTIS SERINE PROTEASE 1 PRECURSOR.			X
IPI00373867	GLTPD2	GLYCOLIPID TRANSFER PROTEIN DOMAIN CONTAINING 2.			X
IPI00374068	ADAMTSL4	ISOFORM 1 OF ADAMTS-LIKE PROTEIN 4 PRECURSOR.			X
IPI00374074	LOC388795	SIMILAR TO CG40449-PA.3.			X
IPI00374223	C3orf54	HYPOTHETICAL PROTEIN LOC389119.			X
IPI00374293	LOC729595	SIMILAR TO HIGH-MOBILITY GROUP BOX 3.			X
IPI00374804	DGKD	ISOFORM 1 OF DIACYLGLYCEROL KINASE DELTA.			X
IPI00375225	OR2AE1	OLFACTORY RECEPTOR 2AE1.			X
IPI00375325	KRTAP10-10	KERATIN-ASSOCIATED PROTEIN 10-10.			X
IPI00375820	IFITM5	INTERFERON-INDUCED TRANSMEMBRANE PROTEIN 5.			X
IPI00376206	HSD17B13	ISOFORM 2 OF 17-BETA HYDROXYSTEROID DEHYDROGENASE 13 PRECURSOR.			X
IPI00376213	GPR120	ISOFORM 1 OF G-PROTEIN COUPLED RECEPTOR 120.			X
IPI00376317	EDC4	ISOFORM 1 OF ENHANCER OF MRNA-DECAPPING PROTEIN 4.			X
IPI00377211	CDH26	ISOFORM 3 OF CADHERIN-LIKE PROTEIN 26 PRECURSOR.			X
IPI00377214	NLRX1	ISOFORM 2 OF NLR FAMILY MEMBER X1.			X
IPI00382394	PLA2G4B	ISOFORM 5 OF CYTOSOLIC PHOSPHOLIPASE A2 BETA.			X
IPI00382422		IG LAMBDA CHAIN V-I REGION NEWM.			X
IPI00382424		IG LAMBDA CHAIN V-II REGION NEI.			X
IPI00382426		IG LAMBDA CHAIN V-II REGION TRO.			X
IPI00382439		IG LAMBDA CHAIN V-IV REGION X.			X
IPI00382483		IG HEAVY CHAIN V-III REGION GA.			X
IPI00382490		IG HEAVY CHAIN V-III REGION BUR.			X
IPI00382492		IG HEAVY CHAIN V-III REGION LAY.			X
IPI00382495		IG HEAVY CHAIN V-III REGION ZAP.			X
IPI00382671	CLEC4M	ISOFORM 6 OF C-TYPE LECTIN DOMAIN FAMILY 4 MEMBER M.			X
IPI00382831	BAP1	CEREBRAL PROTEIN-13.			X
IPI00383261	PEX6	PEROXIN PEX6P.			X
IPI00383294	MNT	PUTATIVE UNCHARACTERIZED PROTEIN PP7518.			X
IPI00383318		PRO2272.			X
IPI00383774	CASP4	MIH1/TX PROTEIN.			X

Table S1. Proteins Identified or Not in MS Patients Compared to Normals and Other Neurologic Disease (OND)					
**IPI**	**Gene**	**Protein Name**	**MS**	**Normal**	**OND**
IPI00383895	PRDM11	CDNA FLJ40559 FIS, CLONE THYMU2002910.			X
IPI00384013	GALNTL2	POLYPEPTIDE N-ACETYLGALACTOSAMINYLTRANSFERASE-LIKE PROTEIN 2.			X
IPI00384140	ESR2	FULL-LENGTH CDNA CLONE CS0DA005YA22 OF NEUROBLASTOMA OF HOMO SAPIENS (FRAGMENT).			X
IPI00384164	PGF	FULL-LENGTH CDNA CLONE CS0DI075YC18 OF PLACENTA OF HOMO SAPIENS (FRAGMENT).			X
IPI00384268	XRCC6BP1	XRCC6BP1 PROTEIN.			X
IPI00384280	PCYOX1	PRENYLCYSTEINE OXIDASE 1 PRECURSOR.			X
IPI00384447	NEXN	SARCOMA ANTIGEN NY-SAR-22 (FRAGMENT).			X
IPI00384546		CDNA FLJ25883 FIS, CLONE CBR02735.			X
IPI00384549	TTC31	TTC31 PROTEIN (FRAGMENT).			X
IPI00384767	TBC1D10C	FLJ00332 PROTEIN (FRAGMENT).			X
IPI00384874	ABCA10	ISOFORM 1 OF ATP-BINDING CASSETTE SUB-FAMILY A MEMBER 10.			X
IPI00384971	DKFZP686E2158	HYPOTHETICAL PROTEIN LOC643155.			X
IPI00385020	CAPRIN2	PUTATIVE UNCHARACTERIZED PROTEIN DKFZP686D159 (FRAGMENT).			X
IPI00385065	CCDC46	ISOFORM 1 OF COILED-COIL DOMAIN-CONTAINING PROTEIN 46.			X
IPI00385153	ATXN10	HUMEEP.			X
IPI00385255		IG LAMBDA CHAIN V-II REGION NIG-84.			X
IPI00385321	ZMYND8	ISOFORM 2 OF PROTEIN KINASE C-BINDING PROTEIN 1.			X
IPI00385495	LMF2	TRANSMEMBRANE PROTEIN 153.			X
IPI00385511	TNRC6B	UNCHARACTERIZED PROTEIN TNRC6B.			X
IPI00385528		ALPHA-1,4-GALACTOSYLTRANSFERASE.			X
IPI00385578	MLL5	HDCMC04P.			X
IPI00385687		SIMILAR TO UBIQUINOL-CYTOCHROME C REDUCTASE COMPLEX 14 KDA PROTEIN.			X
IPI00385737	LOC646853	SIMILAR TO NONHISTONE CHROMOSOMAL PROTEIN HMG-17.			X
IPI00385799	GFER	ERV1-LIKE GROWTH FACTOR.			X
IPI00386113	C20orf59	CDNA: FLJ23412 FIS, CLONE HEP20516.			X
IPI00386119	SF1	ISOFORM 5 OF SPLICING FACTOR 1.			X
IPI00386185		FLJ00370 PROTEIN (FRAGMENT).			X
IPI00386364	OR2L2	OLFACTORY RECEPTOR 2L2.			X
IPI00386390	OR6N2	OLFACTORY RECEPTOR 6N2.			X
IPI00386574		IG LAMBDA CHAIN V-I REGION MEM.			X
IPI00386651	LOC404266	LOC404266 PROTEIN.			X

Table S1. Proteins Identified or Not in MS Patients Compared to Normals and Other Neurologic Disease (OND)					
**IPI**	**Gene**	**Protein Name**	**MS**	**Normal**	**OND**
IPI00386797	FNIP1	ISOFORM 1 OF FOLLICULIN-INTERACTING PROTEIN 1.			X
IPI00386822	TGFBI	PUTATIVE UNCHARACTERIZED PROTEIN (FRAGMENT).			X
IPI00387098		IG KAPPA CHAIN V-I REGION OU.			X
IPI00387099		IG KAPPA CHAIN V-I REGION REI.			X
IPI00387109		IG KAPPA CHAIN V-II REGION FR.			X
IPI00387132	hCG_2007354	CDNA FLJ32661 FIS, CLONE TESTI1000055, WEAKLY SIMILAR TO HOMEOBOX PROTEIN SIX1.			X
IPI00387161		IG LAMBDA CHAIN V-I REGION VOR.			X
IPI00394807	REG3G	REGENERATING ISLET-DERIVED PROTEIN 3 GAMMA PRECURSOR.			X
IPI00394818	KANK3	ISOFORM 1 OF ANKYRIN REPEAT DOMAIN-CONTAINING PROTEIN 47.			X
IPI00394960	GPR133	ISOFORM 1 OF PROBABLE G-PROTEIN COUPLED RECEPTOR 133 PRECURSOR.			X
IPI00394994	C14orf49	ISOFORM 1 OF NESPRIN-3.			X
IPI00395010	C5orf42	HYPOTHETICAL PROTEIN LOC65250.			X
IPI00395376	SEMA5B	ISOFORM 1 OF SEMAPHORIN-5B.			X
IPI00395444	CAMSAP1	CALMODULIN REGULATED SPECTRIN-ASSOCIATED PROTEIN 1.			X
IPI00395473	ZNF135	ZINC FINGER PROTEIN 135.			X
IPI00395603	C18orf34	ISOFORM 3 OF UNCHARACTERIZED PROTEIN C18ORF34.			X
IPI00395630	SCRIB	PUTATIVE UNCHARACTERIZED PROTEIN PP6170.			X
IPI00395631	TRAF7	ISOFORM 1 OF E3 UBIQUITIN-PROTEIN LIGASE TRAF7.			X
IPI00395667	IFRD2; NAT6	INTERFERON-RELATED IFRD2 (PC4-B) PROTEIN.			X
IPI00395769	ATP5C1	ISOFORM HEART OF ATP SYNTHASE SUBUNIT GAMMA, MITOCHONDRIAL PRECURSOR.			X
IPI00396025	FLJ30934	HYPOTHETICAL PROTEIN LOC254122.			X
IPI00396063	DENND4A	C-MYC PROMOTER-BINDING PROTEIN.			X
IPI00396147	PCDH20	PROTOCADHERIN-20 PRECURSOR.			X
IPI00396279	CLASP1	ISOFORM 1 OF CLIP-ASSOCIATING PROTEIN 1.			X
IPI00396314	CNOT10	ISOFORM 3 OF CCR4-NOT TRANSCRIPTION COMPLEX SUBUNIT 10.			X
IPI00396527	CCDC128	ISOFORM 1 OF COILED-COIL DOMAIN-CONTAINING PROTEIN 128.			X
IPI00397015	AHDC1	A.T HOOK DNA-BINDING MOTIF-CONTAINING PROTEIN 1.			X
IPI00397358		SIMILAR TO RIBOSOMAL PROTEIN S27.			X
IPI00397622	TMIGD1	ISOFORM 1 OF TRANSMEMBRANE AND IMMUNOGLOBULIN DOMAIN-CONTAINING PROTEIN 1 PRECURSOR.			X
IPI00397740	ZNF749	ZINC FINGER PROTEIN 749.			X
IPI00397759	LOC388621	SIMILAR TO RIBOSOMAL PROTEIN L21.			X

Table S1. Proteins Identified or Not in MS Patients Compared to Normals and Other Neurologic Disease (OND)					
IPI	Gene	Protein Name	MS	Normal	OND
IPI00398162	NRAP	ISOFORM 2 OF NEBULIN-RELATED-ANCHORING PROTEIN.			X
IPI00398272	COL17A1	ISOFORM 1 OF COLLAGEN ALPHA-1(XVII) CHAIN.			X
IPI00398421	KCNT2	ISOFORM 1 OF POTASSIUM CHANNEL SUBFAMILY T MEMBER 2.			X
IPI00398586	LOC199800	HYPOTHETICAL PROTEIN LOC199800.			X
IPI00398709	CATSPER4	ISOFORM 1 OF CATION CHANNEL SPERM-ASSOCIATED PROTEIN 4.			X
IPI00398725	ZNF644	ISOFORM 1 OF ZINC FINGER PROTEIN 644.			X
IPI00398779	PLEC1	ISOFORM 4 OF PLECTIN-1.			X
IPI00398900	HMX2	HOMEOBOX PROTEIN HMX2.			X
IPI00399320	TMEM59	TRANSMEMBRANE PROTEIN 59 PRECURSOR.			X
IPI00400795		54 KDA PROTEIN.			X
IPI00400834	MED13L	MEDIATOR OF RNA POLYMERASE II TRANSCRIPTION SUBUNIT 13-LIKE.			X
IPI00401002	ZNF740	ZINC FINGER PROTEIN 740.			X
IPI00401190	LOC155006	LOC155006 PROTEIN.			X
IPI00401586	C18orf62	CHROMOSOME 18 OPEN READING FRAME 62.			X
IPI00401776	MUC6	MUCIN 6, GASTRIC.			X
IPI00401789	HDX	ISOFORM 2 OF HIGHLY DIVERGENT HOMEOBOX.			X
IPI00401829		PUTATIVE UNCHARACTERIZED PROTEIN ENST00000281581.			X
IPI00402006		SIMILAR TO DNA-BINDING PROTEIN.			X
IPI00402291	LOC402117	UNCHARACTERIZED PROTEIN ENSP00000308976.			X
IPI00409659	UBQLN2	UBIQUILIN-2.			X
IPI00410214	BPNT1	ISOFORM 1 OF 3'(2'),5'-BISPHOSPHATE NUCLEOTIDASE 1.			X
IPI00410320	SLC5A11	SODIUM-DEPENDENT GLUCOSE TRANSPORTER.			X
IPI00410391	MFSD2	ISOFORM 1 OF MAJOR FACILITATOR SUPERFAMILY DOMAIN-CONTAINING PROTEIN 2.			X
IPI00410464	ZNF324B; ZNF584	ISOFORM 1 OF ZINC FINGER PROTEIN 324B.			X
IPI00410564	FLJ45248	CDNA FLJ45248 FIS, CLONE BRHIP2006819.			X
IPI00410590	LSM14A	ISOFORM 2 OF LSM14 PROTEIN HOMOLOG A.			X
IPI00410616	NEIL2	ISOFORM 1 OF ENDONUCLEASE VIII-LIKE 2.			X
IPI00410631	FAM45A; FAM45B	PROTEIN FAM45A.			X
IPI00411291	PEX1	PEROXISOME BIOGENESIS FACTOR 1.			X
IPI00411298	CNKSR3	CONNECTOR ENHANCER OF KINASE SUPPRESSOR OF RAS 3.			X
IPI00411452	DOCK11	UNCHARACTERIZED PROTEIN DOCK11.			X
IPI00411480	LOC729461	HYPOTHETICAL PROTEIN.			X

Table S1. Proteins Identified or Not in MS Patients Compared to Normals and Other Neurologic Disease (OND)					
**IPI**	**Gene**	**Protein Name**	**MS**	**Normal**	**OND**
IPI00411545	THPO	ISOFORM 3 OF THROMBOPOIETIN PRECURSOR.			X
IPI00411635		180 KDA PROTEIN.			X
IPI00411637	GAL3ST4	GALACTOSE-3-O-SULFOTRANSFERASE 4.			X
IPI00411690	LARP1	ISOFORM 3 OF LA-RELATED PROTEIN 1.			X
IPI00411979	FMNL3	ISOFORM 3 OF FORMIN-LIKE PROTEIN 3.			X
IPI00412024	ZNF485	ZINC FINGER PROTEIN 485.			X
IPI00412107	FLJ43980	FLJ43980 PROTEIN.			X
IPI00412272	SH3BGRL2	SH3 DOMAIN-BINDING GLUTAMIC ACID-RICH-LIKE PROTEIN 2.			X
IPI00412298	ATR	ISOFORM 1 OF SERINE/THREONINE-PROTEIN KINASE ATR.			X
IPI00412977		PROTHYMOSIN ALPHA.			X
IPI00413272	MED23	ISOFORM 3 OF MEDIATOR OF RNA POLYMERASE II TRANSCRIPTION SUBUNIT 23.			X
IPI00413293	TOR1A	ISOFORM 1 OF TORSIN-1A PRECURSOR.			X
IPI00413385	ITIH5L	INTER-ALPHA-TRYPSIN INHIBITOR HEAVY CHAIN H5-LIKE PROTEIN PRECURSOR.			X
IPI00413655	ANKRD55	ANKYRIN REPEAT DOMAIN 55 ISOFORM 1.			X
IPI00413868	C6orf60	HYPOTHETICAL PROTEIN LOC79632 ISOFORM 1.			X
IPI00413930	ELOVL2	ELONGATION OF VERY LONG CHAIN FATTY ACIDS PROTEIN 2.			X
IPI00414122	CCDC141	COILED-COIL DOMAIN CONTAINING 141.			X
IPI00414259	PSG4	ISOFORM 2 OF PREGNANCY-SPECIFIC BETA-1-GLYCOPROTEIN 4 PRECURSOR.			X
IPI00414402	OR2T27	OLFACTORY RECEPTOR 2T27.			X
IPI00414591	MICAL2	FLAVOPROTEIN OXIDOREDUCTASE.			X
IPI00414784	CD300A	ISOFORM 1 OF CMRF35-H ANTIGEN PRECURSOR.			X
IPI00414819	SKIV2L	HELICASE SKI2W.			X
IPI00414973	FASTKD5	FAST KINASE DOMAIN-CONTAINING PROTEIN 5.			X
IPI00418164	LOC731440; RP4-692D3.1	NOVEL PROTEIN.			X
IPI00418236	FIGLA	FACTOR IN THE GERMLINE ALPHA.			X
IPI00418238	HRBL	ISOFORM 1 OF HIV-1 REV-BINDING PROTEIN-LIKE PROTEIN.			X
IPI00418336	INTS3	ISOFORM 2 OF INTEGRATOR COMPLEX SUBUNIT 3.			X
IPI00418408	CYP2D7P1	CYTOCHROME P450.			X
IPI00418426	CNNM4	METAL TRANSPORTER CNNM4.			X
IPI00418573	UNC5A	ISOFORM 2 OF NETRIN RECEPTOR UNC5A PRECURSOR.			X
IPI00418614		67 KDA PROTEIN.			X
IPI00418885	LCN10	EPIDIDYMAL-SPECIFIC LIPOCALIN-10 PRECURSOR.			X
IPI00419180	CC2D2B	CDNA FLJ41429 FIS, CLONE BRHIP2005354.			X

Table S1. Proteins Identified or Not in MS Patients Compared to Normals and Other Neurologic Disease (OND)					
**IPI**	**Gene**	**Protein Name**	**MS**	**Normal**	**OND**
IPI00419215	A2ML1	ALPHA-2-MACROGLOBULIN-LIKE 1.			X
IPI00419385	OR52E8	OLFACTORY RECEPTOR, FAMILY 52, SUBFAMILY E, MEMBER 8.			X
IPI00419575	C7orf20	PROTEIN OF UNKNOWN FUNCTION DUF410 FAMILY PROTEIN.			X
IPI00419791	RSRC2	ISOFORM 1 OF ARGININE/SERINE-RICH COILED-COIL PROTEIN 2.			X
IPI00419815	GDF6	GROWTH/DIFFERENTIATION FACTOR 6 PRECURSOR.			X
IPI00419903	LOC554235	PUTATIVE L-ASPARTATE DEHYDROGENASE.			X
IPI00419912	YY2	YY2 TRANSCRIPTION FACTOR.			X
IPI00419992	AASDH	ISOFORM 1 OF ACYL-COA SYNTHETASE FAMILY MEMBER 4.			X
IPI00423379	C20orf96	PUTATIVE UNCHARACTERIZED PROTEIN DKFZP686G24192.			X
IPI00423562	USP6	ISOFORM 1 OF UBIQUITIN CARBOXYL-TERMINAL HYDROLASE 6.			X
IPI00425404	KIF21A	ISOFORM 1 OF KINESIN-LIKE PROTEIN KIF21A.			X
IPI00425688	RUSC1	ISOFORM 1 OF RUN AND SH3 DOMAIN-CONTAINING PROTEIN 1.			X
IPI00426267	LRRC7	ISOFORM 1 OF LEUCINE-RICH REPEAT-CONTAINING PROTEIN 7.			X
IPI00427808	C10orf71	ISOFORM 1 OF UNCHARACTERIZED PROTEIN C10ORF71.			X
IPI00428677		PUTATIVE OLFACTORY RECEPTOR (FRAGMENT).			X
IPI00429190	RAB11A	RAS-RELATED PROTEIN RAB-11A.			X
IPI00430079	PHF8	ISOFORM 2 OF PHD FINGER PROTEIN 8.			X
IPI00430792	LMAN1L	ISOFORM 1 OF PROTEIN ERGIC-53-LIKE PRECURSOR.			X
IPI00431697	RP13-36C9.6	CANCER/TESTIS ANTIGEN 45-5.			X
IPI00432893	INPP5F	INPP5F PROTEIN (FRAGMENT).			X
IPI00435928	RASGRF1	PP13187.			X
IPI00436518		BENZODIAZEPINE RECEPTOR LIGAND.			X
IPI00438355	FBN3	FIBRILLIN-3 PRECURSOR.			X
IPI00439935	UGT1A4; UGT1A6	UDP-GLUCURONOSYLTRANSFERASE 1-4 PRECURSOR.			X
IPI00440764	GGT6	ISOFORM 1 OF GAMMA-GLUTAMYLTRANSFERASE 6 PRECURSOR.			X
IPI00441064	C15orf26	ISOFORM 1 OF UNCHARACTERIZED PROTEIN C15ORF26.			X
IPI00442002	SCUBE2	ISOFORM 3 OF SIGNAL PEPTIDE, CUB AND EGF-LIKE DOMAIN-CONTAINING PROTEIN 2 PRECURSOR.			X
IPI00442208	ADCY4	CDNA FLJ16344 FIS, CLONE TESTI2032643, MODERATELY SIMILAR TO ADENYLATE CYCLASE, TYPE IV.			X
IPI00442551	ATP9B	CDNA FLJ26989 FIS, CLONE SLV03395.			X
IPI00442582		CDNA FLJ26893 FIS, CLONE RCT00305.			X
IPI00442601		CDNA FLJ26817 FIS, CLONE PRS06446.			X

Table S1. Proteins Identified or Not in MS Patients Compared to Normals and Other Neurologic Disease (OND)					
**IPI**	**Gene**	**Protein Name**	**MS**	**Normal**	**OND**
IPI00444138	AFAP1	CDNA FLJ46074 FIS, CLONE TESTI2001915, HIGHLY SIMILAR TO HOMO SAPIENS ACTIN FILAMENT ASSOCIATED PROTEIN.			X
IPI00444208	RNF44	CDNA FLJ45771 FIS, CLONE NETRP2003103.			X
IPI00444234	FLJ45743	CDNA FLJ45743 FIS, CLONE KIDNE2016464.			X
IPI00444330		CDNA FLJ45693 FIS, CLONE FEBRA2012625.			X
IPI00444375	RHCE	RH BLOOD GROUP, CCEE ANTIGENS.			X
IPI00444395		CDNA FLJ45615 FIS, CLONE BRTHA3026180.			X
IPI00444408		CDNA FLJ45600 FIS, CLONE BRTHA3020369.			X
IPI00444502	FLJ45482	CDNA FLJ45482 FIS, CLONE BRTHA2001953.			X
IPI00444706	KALRN	CDNA FLJ45007 FIS, CLONE BRAWH3012005.			X
IPI00445054	FLJ44715	CDNA FLJ44715 FIS, CLONE BRACE3021430.			X
IPI00445167	SRCAP	CDNA FLJ44499 FIS, CLONE UTERU3000665, HIGHLY SIMILAR TO HOMO SAPIENS SNF2-RELATED CBP ACTIVATOR PROTEIN.			X
IPI00445563	C4orf42	PUTATIVE UNCHARACTERIZED PROTEIN MGC21675/FLJ43787.			X
IPI00445643		CDNA FLJ43684 FIS, CLONE TBAES2001492.			X
IPI00445750		CDNA FLJ43142 FIS, CLONE CTONG3007870.			X
IPI00445796	ACAD8	CDNA FLJ43383 FIS, CLONE OCBBF2006058, HIGHLY SIMILAR TO HOMO SAPIENS ACYL-COENZYME A DEHYDROGENASE-8 MRNA.			X
IPI00445804	DKFZp761B107	UNCHARACTERIZED PROTEIN ENSP00000374264.			X
IPI00445894		CDNA FLJ42978 FIS, CLONE BRTHA2004821.			X
IPI00446138		CDNA FLJ42768 FIS, CLONE BRAWH3003522.			X
IPI00446312		CDNA FLJ42271 FIS, CLONE TKIDN2015788.			X
IPI00446355	IER5L	CDNA FLJ41803 FIS, CLONE NHNPC2002749.			X
IPI00446685		CDNA FLJ41345 FIS, CLONE BRAWH2002761.			X
IPI00446711		CDNA FLJ41261 FIS, CLONE BRAMY2034920.			X
IPI00446753	SSPO	SCO-SPONDIN PRECURSOR.			X
IPI00446767	TLE4	ISOFORM 3 OF TRANSDUCIN-LIKE ENHANCER PROTEIN 4.			X
IPI00446785		CDNA FLJ41116 FIS, CLONE BRACE1000572.			X
IPI00447328	CCDC40	ISOFORM 5 OF COILED-COIL DOMAIN-CONTAINING PROTEIN 40.			X
IPI00449071	PAX6	ISOFORM 5A OF PAIRED BOX PROTEIN PAX-6.			X
IPI00450358	TOP	PUTATIVE UNCHARACTERIZED PROTEIN.			X
IPI00451413	ITSN1	INTERSECTIN 1 ISOFORM 7.			X
IPI00451450	PRSS35	INACTIVE SERINE PROTEASE 35 PRECURSOR.			X

Table S1. Proteins Identified or Not in MS Patients Compared to Normals and Other Neurologic Disease (OND)					
**IPI**	**Gene**	**Protein Name**	**MS**	**Normal**	**OND**
IPI00452161	MCOLN1	ISOFORM 1 OF MUCOLIPIN-1.			X
IPI00454620		HERV-K_11Q22.1 PROVIRUS ANCESTRAL POL PROTEIN.			X
IPI00455076	DOC2A	DOUBLE C2-LIKE DOMAIN-CONTAINING PROTEIN ALPHA.			X
IPI00455083		UNCHARACTERIZED PROTEIN ENSP00000371558.			X
IPI00455397	DMWD	DMWD PROTEIN.			X
IPI00455675	CEP192	ISOFORM 2 OF CENTROSOMAL PROTEIN OF 192 KDA.			X
IPI00455731	FAM26F	PROTEIN FAM26F.			X
IPI00455927		UNCHARACTERIZED PROTEIN ENSP00000366638 (FRAGMENT).			X
IPI00456060		PROTEIN.			X
IPI00456996	PVT1	CONSERVED HYPOTHETICAL PROTEIN.			X
IPI00457114	IQSEC1	ISOFORM 1 OF IQ MOTIF AND SEC7 DOMAIN-CONTAINING PROTEIN 1.			X
IPI00464980	SIN3B	ISOFORM 1 OF PAIRED AMPHIPATHIC HELIX PROTEIN SIN3B.			X
IPI00464994	GTPBP3	ISOFORM 1 OF TRNA MODIFICATION GTPASE GTPBP3, MITOCHONDRIAL PRECURSOR.			X
IPI00465054	THUMPD1	PUTATIVE UNCHARACTERIZED PROTEIN DKFZP686C1054.			X
IPI00465070	HIST1H2BN; HIST1H3A; HIST1H3B; HIST1H3C; HIST1H3D; HIST1H3E; HIST1H3F; HIST1H3G; HIST1H3H; HIST1H3I; HIST1H3J	HISTONE H3.1.			X
IPI00465087	BAP1	UBIQUITIN CARBOXYL-TERMINAL HYDROLASE BAP1.			X
IPI00465168	TAOK2	ISOFORM 1 OF SERINE/THREONINE-PROTEIN KINASE TAO2.			X
IPI00465173	SLC47A1	ISOFORM 1 OF MULTIDRUG AND TOXIN EXTRUSION PROTEIN 1.			X
IPI00465177	TGFA	TRANSFORMING GROWTH FACTOR-ALPHA VARIANT I.			X
IPI00465247	STRA6	ISOFORM 1 OF STIMULATED BY RETINOIC ACID GENE 6 PROTEIN HOMOLOG.			X
IPI00465273	UHRF1BP1	UHRF1-BINDING PROTEIN 1.			X
IPI00465319	CHPF	CHONDROITIN SULFATE SYNTHASE 2.			X
IPI00465345	PRPF40B	ISOFORM 3 OF PRE-MRNA-PROCESSING FACTOR 40 HOMOLOG B.			X

Table S1. Proteins Identified or Not in MS Patients Compared to Normals and Other Neurologic Disease (OND)					
**IPI**	**Gene**	**Protein Name**	**MS**	**Normal**	**OND**
IPI00470515	C1orf173	ISOFORM 1 OF UNCHARACTERIZED PROTEIN C1ORF173.			X
IPI00470518	MAD1L1	MITOTIC SPINDLE ASSEMBLY CHECKPOINT PROTEIN MAD1.			X
IPI00470596	ZNF638	ISOFORM 1 OF ZINC FINGER PROTEIN 638.			X
IPI00470606	TRMT11	ISOFORM 1 OF TRNA GUANOSINE-2'-O-METHYLTRANSFERASE TRM11 HOMOLOG.			X
IPI00470657		ANTI-COLORECTAL CARCINOMA HEAVY CHAIN.			X
IPI00470771	SAMD4B	SAMD4B PROTEIN.			X
IPI00470812	LOC339977	PUTATIVE UNCHARACTERIZED PROTEIN DKFZP781E21107.			X
IPI00470896	ZFYVE26	ISOFORM 2 OF ZINC FINGER FYVE DOMAIN-CONTAINING PROTEIN 26.			X
IPI00470917	KIAA1529	ISOFORM 2 OF UNCHARACTERIZED PROTEIN KIAA1529.			X
IPI00472171	RPL7	RPL7 PROTEIN.			X
IPI00472374	ZNF98	ZINC FINGER PROTEIN 98.			X
IPI00472426	ZNF780B	ZINC FINGER PROTEIN 780B.			X
IPI00472712	CCDC7	COILED-COIL DOMAIN CONTAINING 7.			X
IPI00472782	PHF14	PHD FINGER PROTEIN 14 ISOFORM 1.			X
IPI00477262	TRIM41	UNCHARACTERIZED PROTEIN TRIM41 (FRAGMENT).			X
IPI00477396	ZNF324B; ZNF584	UNCHARACTERIZED PROTEIN ZNF324B (FRAGMENT).			X
IPI00477692	LOC131149	UNCHARACTERIZED PROTEIN ENSP00000330808.			X
IPI00477693	LOC646486	FATTY ACID BINDING PROTEIN.			X
IPI00477763	CDC42BPB	SERINE/THREONINE-PROTEIN KINASE MRCK BETA.			X
IPI00477766	BBS9	ISOFORM 3 OF PROTEIN PTHB1.			X
IPI00477949	ZMYM4	ISOFORM 1 OF ZINC FINGER MYM-TYPE PROTEIN 4.			X
IPI00478058	NARG1L	ISOFORM 2 OF NMDA RECEPTOR-REGULATED 1-LIKE PROTEIN.			X
IPI00478110	GPR135	PROBABLE G-PROTEIN COUPLED RECEPTOR 135.			X
IPI00478354	CXorf31	PUTATIVE UNCHARACTERIZED PROTEIN CXORF31.			X
IPI00478572	COL4A4	COLLAGEN ALPHA-4(IV) CHAIN PRECURSOR.			X
IPI00479296	ABCA8	ISOFORM 1 OF ATP-BINDING CASSETTE SUB-FAMILY A MEMBER 8.			X
IPI00479430	C1QTNF9	ISOFORM 2 OF COMPLEMENT C1Q TUMOR NECROSIS FACTOR-RELATED PROTEIN 9 PRECURSOR.			X
IPI00479578	hCG_1659830	SIMILAR TO NONHISTONE CHROMOSOMAL PROTEIN HMG-14.			X
IPI00479625	ELAVL4	ISOFORM 1 OF ELAV-LIKE PROTEIN 4.			X
IPI00479789	C1orf103	ISOFORM 1 OF UNCHARACTERIZED PROTEIN C1ORF103.			X
IPI00479893	C10orf18	ISOFORM 2 OF UNCHARACTERIZED PROTEIN C10ORF18.			X
IPI00479897	C9orf75	CHROMOSOME 9 OPEN READING FRAME 75.			X

Table S1. Proteins Identified or Not in MS Patients Compared to Normals and Other Neurologic Disease (OND)					
IPI	Gene	Protein Name	MS	Normal	OND
IPI00479904	COL27A1	UNCHARACTERIZED PROTEIN COL27A1.			X
IPI00514028	RNF207	21 KDA PROTEIN.			X
IPI00514153	TMTC4	TRANSMEMBRANE AND TETRATRICOPEPTIDE REPEAT CONTAINING 4 ISOFORM 1.			X
IPI00514197	LOC648822	SIMILAR TO NONHISTONE CHROMOSOMAL PROTEIN HMG-17.			X
IPI00514394	FAM120AOS	FAMILY WITH SEQUENCE SIMILARITY 120A OPPOSITE STRAND.			X
IPI00514533	SYCP2L	UNCHARACTERIZED PROTEIN SYCP2L.			X
IPI00514551	SCML4	OTTHUMP00000016936.			X
IPI00514795	TTLL7	ISOFORM 1 OF TUBULIN--TYROSINE LIGASE-LIKE PROTEIN 7.			X
IPI00514843	KIAA0082	KIAA0082.			X
IPI00514966	RBPJL	RECOMBINING BINDING PROTEIN SUPPRESSOR OF HAIRLESS.			X
IPI00514975	C1orf133	CHROMOSOME 1 OPEN READING FRAME 133.			X
IPI00515034	KIFAP3	87 KDA PROTEIN.			X
IPI00549189	THOP1	THIMET OLIGOPEPTIDASE.			X
IPI00549384	BRD9	ISOFORM 1 OF BROMODOMAIN-CONTAINING PROTEIN 9.			X
IPI00549844	FAM134B	ISOFORM 1 OF PROTEIN FAM134B.			X
IPI00550069	RNH1	RIBONUCLEASE INHIBITOR.			X
IPI00550272	INTS10	INTEGRATOR COMPLEX SUBUNIT 10.			X
IPI00550571	C5orf15	KERATINOCYTES-ASSOCIATED TRANSMEMBRANE PROTEIN 2 PRECURSOR.			X
IPI00550862	LOC387911	HYPOTHETICAL PROTEIN LOC387911.			X
IPI00552213	DLG1	ISOFORM 6 OF DISKS LARGE HOMOLOG 1.			X
IPI00552434	MYH7B	MYOSIN, HEAVY CHAIN 7B, CARDIAC MUSCLE, BETA.			X
IPI00552455	INPP5A	12 KDA PROTEIN.			X
IPI00552601		PUTATIVE UNCHARACTERIZED PROTEIN DKFZP686M12165 (FRAGMENT).			X
IPI00552840	GPR112	PROBABLE G-PROTEIN COUPLED RECEPTOR 112.			X
IPI00553006	PARS2	PROBABLE PROLYL-TRNA SYNTHETASE, MITOCHONDRIAL PRECURSOR.			X
IPI00553067	CCDC132	ISOFORM 1 OF COILED-COIL DOMAIN-CONTAINING PROTEIN 132.			X
IPI00554488	PRKAR1B	CAMP-DEPENDENT PROTEIN KINASE TYPE I-BETA REGULATORY SUBUNIT.			X
IPI00554701	UCRC	CYTOCHROME B-C1 COMPLEX SUBUNIT 9.			X
IPI00555595		UBIQUITIN CARBOXYL-TERMINAL ESTERASE L1 (UBIQUITIN THIOLESTERASE) VARIANT (FRAGMENT).			X
IPI00555621		P21-ACTIVATED KINASE 2 VARIANT (FRAGMENT).			X
IPI00556381	TRIM16	PUTATIVE UNCHARACTERIZED PROTEIN (FRAGMENT).			X
IPI00604549	INPP5D; LOC646743	ISOFORM 2 OF PHOSPHATIDYLINOSITOL-3,4,5-TRISPHOSPHATE 5-PHOSPHATASE 1.			X

Table S1. Proteins Identified or Not in MS Patients Compared to Normals and Other Neurologic Disease (OND)					
**IPI**	**Gene**	**Protein Name**	**MS**	**Normal**	**OND**
IPI00604752	SAMD13	ISOFORM 3 OF STERILE ALPHA MOTIF DOMAIN-CONTAINING PROTEIN 13.			X
IPI00604798		BITTER TASTE RECEPTOR T2R2 (FRAGMENT).			X
IPI00607829	RFPL2	ISOFORM 1 OF RET FINGER PROTEIN-LIKE 2.			X
IPI00619927	NAALAD2	NAALAD2 PROTEIN.			X
IPI00639924	LOC374920	97 KDA PROTEIN.			X
IPI00640240	SPTLC3	59 KDA PROTEIN.			X
IPI00640293	TIMP3	23 KDA PROTEIN.			X
IPI00640654	RAP1GAP	RAP1 GTPASE ACTIVATING PROTEIN.			X
IPI00640865	ITGBL1	INTEGRIN BETA-LIKE PROTEIN 1 PRECURSOR.			X
IPI00641155	HIRIP3	20 KDA PROTEIN.			X
IPI00641614	AKAP8L	47 KDA PROTEIN.			X
IPI00641920	NECAB2	N-TERMINAL EF-HAND CALCIUM-BINDING PROTEIN 2.			X
IPI00641954	TMED5	22 KDA PROTEIN.			X
IPI00642204	TXNDC16	THIOREDOXIN DOMAIN-CONTAINING PROTEIN 16 PRECURSOR.			X
IPI00642305	MCM8	MCM8 PROTEIN.			X
IPI00642798	SC65	NUCLEOLAR AUTOANTIGEN NO55.			X
IPI00643747	C22orf30	ISOFORM 1 OF UNCHARACTERIZED PROTEIN C22ORF30.			X
IPI00643809	CNTNAP3B	PROTEIN.			X
IPI00644529	ZNF615	ISOFORM 2 OF ZINC FINGER PROTEIN 615.			X
IPI00644785	PLXNB3	PROTEIN.			X
IPI00644968		SIMILAR TO HETEROGENEOUS NUCLEAR RIBONUCLEOPROTEIN A1.			X
IPI00645614	CDH3	ISOFORM 2 OF CADHERIN-3 PRECURSOR.			X
IPI00645729	C1QTNF8	62 KDA PROTEIN.			X
IPI00646199	ZMYM2	ZINC FINGER, MYM-TYPE 2.			X
IPI00646323	C10orf76	ISOFORM 1 OF UNCHARACTERIZED PROTEIN C10ORF76.			X
IPI00646410	AOF1	ISOFORM 2 OF FLAVIN-CONTAINING AMINE OXIDASE DOMAIN-CONTAINING PROTEIN 1.			X
IPI00646423	TAF4B	ISOFORM 2 OF TRANSCRIPTION INITIATION FACTOR TFIID SUBUNIT 4B.			X
IPI00646485	EFCAB2	PROTEIN.			X
IPI00646826	FLJ32658	PUTATIVE UNCHARACTERIZED PROTEIN FLJ32658.			X
IPI00646831		34 KDA PROTEIN.			X
IPI00647124	COL19A1	A1 CHAIN OF TYPE XIX COLLAGEN.			X
IPI00647205	C6orf174	UNCHARACTERIZED PROTEIN C6ORF174 PRECURSOR.			X
IPI00647254		7 KDA PROTEIN.			X

Table S1. Proteins Identified or Not in MS Patients Compared to Normals and Other Neurologic Disease (OND)					
**IPI**	**Gene**	**Protein Name**	**MS**	**Normal**	**OND**
IPI00647897	RNF113B	RING FINGER PROTEIN 113B.			X
IPI00651633	CASKIN1	CASKIN1 PROTEIN (FRAGMENT).			X
IPI00651669		PUTATIVE UNCHARACTERIZED PROTEIN.			X
IPI00654693	FAM44C	PROTEIN FAM44C.			X
IPI00655760		ENVELOPE POLYPROTEIN.			X
IPI00656052	SPANXB1; SPANXB2; SPANXF1	SPANX-C.			X
IPI00657645	XRN1	ISOFORM 2 OF 5'-3' EXORIBONUCLEASE 1.			X
IPI00657892	F8	FACTOR VIII.			X
IPI00658167	CRAMP1L; HN1L	ISOFORM 1 OF PROTEIN CRAMPED-LIKE.			X
IPI00719669	MRLC2	MYOSIN REGULATORY LIGHT CHAIN.			X
IPI00719690	SAMD9L	ISOFORM 1 OF STERILE ALPHA MOTIF DOMAIN-CONTAINING PROTEIN 9-LIKE.			X
IPI00735313		SIMILAR TO PEPTIDYL-PROLYL CIS-TRANS ISOMERASE NIMA-INTERACTING 4.			X
IPI00735857	LOC651123	SIMILAR TO ATP-BINDING CASSETTE SUB-FAMILY D MEMBER 1.			X
IPI00736558	THAP4	THAP DOMAIN CONTAINING 4.			X
IPI00736826	LOC402217	SIMILAR TO TEMPORARILY ASSIGNED GENE NAME FAMILY MEMBER.			X
IPI00737392	LOC646352	SIMILAR TO PROLINE-RICH PROTEIN BSTNI SUBFAMILY 3 PRECURSOR.			X
IPI00737448	LOC338805	SIMILAR TO HEAT SHOCK 70KD PROTEIN BINDING PROTEIN.			X
IPI00737712	CCDC125	CCDC125 PROTEIN.			X
IPI00737735	LOC646913	SIMILAR TO CELL DIVISION CYCLE 10 ISOFORM 1.			X
IPI00737972	TCF24	TRANSCRIPTION FACTOR 24 (FRAGMENT).			X
IPI00738999	LOC643227	SIMILAR TO UBIQUITIN-CONJUGATING ENZYME E2 VARIANT 1.			X
IPI00739387	FLJ45872	HYPOTHETICAL PROTEIN LOC401478.			X
IPI00739676	DDX26B	ISOFORM 2 OF PROTEIN DDX26B.			X
IPI00739927	LOC645464	SIMILAR TO VASOACTIVE INTESTINAL PEPTIDE RECEPTOR 2.			X
IPI00740336	FMN1	FORMIN 1.			X
IPI00741524	THSD7A	THROMBOSPONDIN TYPE-1 DOMAIN-CONTAINING PROTEIN 7A PRECURSOR.			X
IPI00742031	LOC645137	SIMILAR TO GOLGI AUTOANTIGEN, GOLGIN SUBFAMILY A, 8G.			X
IPI00742114	LOC653978	SIMILAR TO TRIPARTITE MOTIF PROTEIN 11.			X
IPI00742127		SIMILAR TO HETEROGENEOUS NUCLEAR RIBONUCLEOPROTEIN A1 (HELIX-DESTABILIZING PROTEIN) (SINGLE-STRAND RNA-BINDING PROTEIN) (HNRNP CORE PROTEIN A1). ISOFORM A1-A.			X

Table S1. Proteins Identified or Not in MS Patients Compared to Normals and Other Neurologic Disease (OND)					
**IPI**	**Gene**	**Protein Name**	**MS**	**Normal**	**OND**
IPI00743335	MYO1C	MYOSIN IC ISOFORM A.			X
IPI00743360	CALCOCO2	CALCIUM-BINDING AND COILED-COIL DOMAIN-CONTAINING PROTEIN 2.			X
IPI00743545		CONSERVED HYPOTHETICAL PROTEIN.			X
IPI00744029		SIMILAR TO ZINC FINGER, FYVE DOMAIN CONTAINING 21.			X
IPI00744179		UNCHARACTERIZED PROTEIN ENSP00000370856 (FRAGMENT).			X
IPI00744357		CONSERVED HYPOTHETICAL PROTEIN.			X
IPI00744527		CONSERVED HYPOTHETICAL PROTEIN.			X
IPI00744574		SIMILAR TO TROPOMYOSIN ALPHA-4 CHAIN.			X
IPI00744816		CONSERVED HYPOTHETICAL PROTEIN.			X
IPI00744872	KIAA0423	ISOFORM 1 OF UNCHARACTERIZED PROTEIN KIAA0423.			X
IPI00744927	FABP6	GASTROTROPIN ISOFORM 1.			X
IPI00744932	ZDHHC1	CONSERVED HYPOTHETICAL PROTEIN.			X
IPI00745346	PAOX	ISOFORM 11 OF PEROXISOMAL N(1)-ACETYL-SPERMINE/SPERMIDINE OXIDASE.			X
IPI00745395	KIAA0508	CONSERVED HYPOTHETICAL PROTEIN.			X
IPI00745396	HNRPLL	STROMAL RNA REGULATING FACTOR.			X
IPI00745433	EIF2C2	EUKARYOTIC TRANSLATION INITIATION FACTOR 2C, 2.			X
IPI00745832		IQ CALMODULIN-BINDING REGION DOMAIN CONTAINING PROTEIN.			X
IPI00745955	EBNA1BP2	EBNA1 BINDING PROTEIN 2.			X
IPI00746034	NAB2	NAB2 PROTEIN.			X
IPI00746222	ABCB5	ATP-BINDING CASSETTE, SUB-FAMILY B (MDR/TAP), MEMBER 5.			X
IPI00746459		CDNA FLJ34517 FIS, CLONE HLUNG2006781.			X
IPI00746600	B4GALT3	44 KDA PROTEIN.			X
IPI00747017	NEK1	PUTATIVE UNCHARACTERIZED PROTEIN DKFZP686D06121.			X
IPI00747264	GABRR1	GAMMA-AMINOBUTYRIC-ACID RECEPTOR SUBUNIT RHO-1 PRECURSOR.			X
IPI00747271	ZNF695	ZINC FINGER PROTEIN SBZF3.			X
IPI00747758	HSPG2	UNCHARACTERIZED PROTEIN HSPG2 (FRAGMENT).			X
IPI00748216	GTF3C5	CDNA FLJ20187 FIS, CLONE COLF0433.			X
IPI00748342	BECN1	BECLIN-1.			X
IPI00748607		CONSERVED HYPOTHETICAL PROTEIN.			X
IPI00748649		25 KDA PROTEIN.			X
IPI00748733		SIMILAR TO SLIT HOMOLOG 3 PROTEIN PRECURSOR.			X
IPI00749062		CONSERVED HYPOTHETICAL PROTEIN.			X
IPI00749338	hCG_1651889	HYPOTHETICAL GENE SUPPORTED BY BC063892.			X
IPI00749489	PKHD1L1	FIBROCYSTIN L.			X

Table S1. Proteins Identified or Not in MS Patients Compared to Normals and Other Neurologic Disease (OND)					
**IPI**	**Gene**	**Protein Name**	**MS**	**Normal**	**OND**
IPI00760993	IQSEC2	IQ MOTIF AND SEC7 DOMAIN 2 ISOFORM 2.			X
IPI00782960	LRRC44	ISOFORM 1 OF LEUCINE-RICH REPEAT AND IQ DOMAIN-CONTAINING PROTEIN 3.			X
IPI00782965	HIP1	HUNTINGTIN INTERACTING PROTEIN 1.			X
IPI00782966	ZFP106	ZINC FINGER PROTEIN 106 HOMOLOG.			X
IPI00783200	RAPGEF3	38 KDA PROTEIN.			X
IPI00783271	LRPPRC	LEUCINE-RICH PPR MOTIF-CONTAINING PROTEIN, MITOCHONDRIAL PRECURSOR.			X
IPI00783656	MRPL38	39S RIBOSOMAL PROTEIN L38, MITOCHONDRIAL PRECURSOR.			X
IPI00784013	JAK1	JANUS KINASE 1.			X
IPI00784027	PDE6B	ROD CGMP-SPECIFIC 3',5'-CYCLIC PHOSPHODIESTERASE SUBUNIT BETA PRECURSOR.			X
IPI00784272		PUTATIVE UNCHARACTERIZED PROTEIN.			X
IPI00786867	DIVA	DUAL INTRACELLULAR VON WILLEBRAND FACTOR DOMAIN A.			X
IPI00786880		MYOSIN-XVB.			X
IPI00786926	IGHV1-69	MYOSIN-REACTIVE IMMUNOGLOBULIN HEAVY CHAIN VARIABLE REGION (FRAGMENT).			X
IPI00786931	LOC729037	SIMILAR TO FATTY ACID AMIDE HYDROLASE.			X
IPI00787099	LOC728676	SIMILAR TO SR PROTEIN RELATED FAMILY MEMBER.			X
IPI00787189	LOC727931	SIMILAR TO THIOREDOXIN DOMAIN CONTAINING 4.			X
IPI00787410	GALNT9	SIMILAR TO POLYPEPTIDE N-ACETYLGALACTOSAMINYLTRANSFERASE 9.			X
IPI00787412	LOC441616	SIMILAR TO PROTEIN C11ORF2.			X
IPI00787427	ANKRD36	ANKYRIN REPEAT DOMAIN 36.			X
IPI00787443	LOC730037	SIMILAR TO COLLAGEN ALPHA-2(XI) CHAIN PRECURSOR.			X
IPI00787587	LOC729549	SIMILAR TO TEMPORARILY ASSIGNED GENE NAME FAMILY MEMBER.			X
IPI00787593	LOC730407	SIMILAR TO CG33300-PA.			X
IPI00787625	LOC389101	SIMILAR TO 60S RIBOSOMAL PROTEIN L23A.			X
IPI00787697	FLJ32810	SIMILAR TO OLIGOPHRENIN 1 ISOFORM 4.			X
IPI00787765	SLC35F4	SIMILAR TO SOLUTE CARRIER FAMILY 35, MEMBER F4.			X
IPI00787774	LOC730756	SIMILAR TO COLLAGEN, TYPE I, ALPHA 1.			X
IPI00787990	LOC644119	SIMILAR TO RIBOSOMAL PROTEIN S14.			X
IPI00788010	LOC728501; RPL21	SIMILAR TO 60S RIBOSOMAL PROTEIN L21.			X
IPI00788014	LOC729015	SIMILAR TO OPIOID GROWTH FACTOR RECEPTOR.			X
IPI00788120	LOC730094	SIMILAR TO CG14182-PA.			X
IPI00789324	JUP	JUP PROTEIN.			X

Table S1. Proteins Identified or Not in MS Patients Compared to Normals and Other Neurologic Disease (OND)					
**IPI**	**Gene**	**Protein Name**	**MS**	**Normal**	**OND**
IPI00789476		7 KDA PROTEIN.			X
IPI00789494	TUSC4	8 KDA PROTEIN.			X
IPI00789564	MPPE1	ISOFORM 3 OF METALLOPHOSPHOESTERASE 1.			X
IPI00789856	TTLL3	TUBULIN TYROSINE LIGASE-LIKE FAMILY, MEMBER 3.			X
IPI00789889		HYPOTHETICAL PROTEIN.			X
IPI00790447		12 KDA PROTEIN.			X
IPI00790813	RAD51AP1	8 KDA PROTEIN.			X
IPI00791447	LOC339929	PSEUDOGENE CANDIDATE.			X
IPI00791509	NEDD1	NEURAL PRECURSOR CELL EXPRESSED, DEVELOPMENTALLY DOWN-REGULATED 1.			X
IPI00791831	ADRBK1	10 KDA PROTEIN.			X
IPI00792370	GNAT1	6 KDA PROTEIN.			X
IPI00793408	ADAMTS9	6 KDA PROTEIN.			X
IPI00793454	USP19	7 KDA PROTEIN.			X
IPI00793585	OC90	57 KDA PROTEIN.			X
IPI00793836	ARHGDIB	11 KDA PROTEIN.			X
IPI00794248		47 KDA PROTEIN.			X
IPI00794307	LOC729505	HYPOTHETICAL PROTEIN.			X
IPI00794791	IQCD	ISOFORM 2 OF IQ DOMAIN-CONTAINING PROTEIN D.			X
IPI00794829	ACADS	16 KDA PROTEIN.			X
IPI00794880	CHD7	ISOFORM 1 OF CHROMODOMAIN-HELICASE-DNA-BINDING PROTEIN 7.			X
IPI00795119	LTBP1	PROTEIN.			X
IPI00795566		6 KDA PROTEIN.			X
IPI00795575		39 KDA PROTEIN.			X
IPI00795736		FER-1-LIKE 4.			X
IPI00795917	CXorf45	126 KDA PROTEIN.			X
IPI00795943		7 KDA PROTEIN.			X
IPI00795992		HYPOTHETICAL SHORT PROTEIN.			X
IPI00796518	NGRN	16 KDA PROTEIN.			X
IPI00796745	PPP1R14D	22 KDA PROTEIN.			X
IPI00796972	ETV5	62 KDA PROTEIN.			X
IPI00797567	GNAI2	9 KDA PROTEIN.			X
IPI00797832	ACAD10	ISOFORM 3 OF ACYL-COA DEHYDROGENASE FAMILY MEMBER 10.			X
IPI00797859	LOC643711	10 KDA PROTEIN.			X
IPI00807406	LOC285346	HYPOTHETICAL PROTEIN ISOFORM 1.			X

Table S1. Proteins Identified or Not in MS Patients Compared to Normals and Other Neurologic Disease (OND)					
**IPI**	**Gene**	**Protein Name**	**MS**	**Normal**	**OND**
IPI00807675	ZNF440	ZNF440 PROTEIN.			X
IPI00815976	TMEM120B	TRANSMEMBRANE PROTEIN 120B.			X
IPI00816289		V2-1 PROTEIN.			X
IPI00829625	FAM91A1	UNCHARACTERIZED PROTEIN FAM91A1.			X
IPI00829745		CELLULAR TITIN ISOFORM PEVK VARIANT 2 (FRAGMENT).			X
IPI00829904		UNCHARACTERIZED PROTEIN ENSP00000374814.			X
IPI00829915	LOC647868	SIMILAR TO PR DOMAIN ZINC FINGER PROTEIN 16.			X
IPI00829925	ELA2A	UNCHARACTERIZED PROTEIN ENSP00000365090.			X
IPI00830059		UNCHARACTERIZED PROTEIN ENSP00000375013.			X
IPI00830073	RNF207	ISOFORM 1 OF RING FINGER PROTEIN 207.			X
IPI00830137		UNCHARACTERIZED PROTEIN ENSP00000374864.			X
IPI00843923	PDE7B	PHOSPHODIESTERASE PDE7B2.			X
IPI00844501	SUV39H1	MG44 PROTEIN.			X
IPI00844512	RHOV	RHOV PROTEIN (FRAGMENT).			X
IPI00845299	LOC285733	PUTATIVE UNCHARACTERIZED PROTEIN LOC285733.			X
IPI00845401	NAV1	ISOFORM 6 OF NEURON NAVIGATOR 1.			X
IPI00847237		CONSERVED HYPOTHETICAL PROTEIN.			X
IPI00847373	KIAA1383	HYPOTHETICAL PROTEIN LOC54627.			X
IPI00847391	ZNF276	ISOFORM 1 OF ZINC FINGER PROTEIN 276.			X
IPI00847409	LOC440338	HYPOTHETICAL PROTEIN.			X
IPI00847415	CLK3	ISOFORM 4 OF DUAL SPECIFICITY PROTEIN KINASE CLK3.			X
IPI00847485		SIMILAR TO PFIV PROTEIN.			X
IPI00847644	SMCHD1	STRUCTURAL MAINTENANCE OF CHROMOSOMES FLEXIBLE HINGE DOMAIN CONTAINING 1.			X
IPI00847790		PROTEIN OF UNKNOWN FUNCTION DUF1725 DOMAIN CONTAINING PROTEIN.			X
IPI00848180		CONSERVED HYPOTHETICAL PROTEIN.			X
IPI00848229	ELTD1	EGF, LATROPHILIN AND SEVEN TRANSMEMBRANE DOMAIN-CONTAINING PROTEIN 1 PRECURSOR.			X
IPI00848252	LOC285501	HYPOTHETICAL PROTEIN LOC285501.			X
IPI00848330	LOC285144	CONSERVED HYPOTHETICAL PROTEIN.			X
IPI00852619	ATP10B	ISOFORM A OF PROBABLE PHOSPHOLIPID-TRANSPORTING ATPASE VB.			X
IPI00852669	ZNF516	ZINC FINGER PROTEIN 516.			X
IPI00852688	LRP5L	ISOFORM 1 OF LOW DENSITY LIPOPROTEIN RECEPTOR-RELATED PROTEIN 5-LIKE PROTEIN.			X

Table S1. Proteins Identified or Not in MS Patients Compared to Normals and Other Neurologic Disease (OND)					
**IPI**	**Gene**	**Protein Name**	**MS**	**Normal**	**OND**
IPI00852699		4 KDA PROTEIN.			X
IPI00853079	C14orf166B	ISOFORM 1 OF UNCHARACTERIZED PROTEIN C14ORF166B.			X
IPI00853133	DDX60L	DEAD (ASP-GLU-ALA-ASP) BOX POLYPEPTIDE 60-LIKE.			X
IPI00853149	TMEM131	RW1 PROTEIN.			X
IPI00853224	STARD7	START DOMAIN CONTAINING 7.			X
IPI00855742	SAMD12	STERILE ALPHA MOTIF DOMAIN CONTAINING 12 ISOFORM A.			X
IPI00855747	RP13-347D8.3	HYPOTHETICAL PROTEIN LOC57481.			X
IPI00855922	DENND3	ISOFORM 4 OF DENN DOMAIN-CONTAINING PROTEIN 3.			X
IPI00855960	GPR137C	INTEGRAL MEMBRANE PROTEIN GPR137C.			X
IPI00856045	AHNAK2	AHNAK NUCLEOPROTEIN 2.			X
IPI00867582	ZNF496	SIMILAR TO SCAN DOMAIN-CONTAINING PROTEIN 2. ISOFORM 2.			X
IPI00867744	PLEKHH2	ISOFORM 3 OF PLECKSTRIN HOMOLOGY DOMAIN-CONTAINING FAMILY H MEMBER 2.			X
IPI00868835		SIMILAR TO HETEROGENEOUS NUCLEAR RIBONUCLEOPROTEINS C1/C2 (HNRNP C1 / HNRNP C2). ISOFORM 4.			X
IPI00869070		ANKYRIN REPEAT CONTAINING PROTEIN.			X
IPI00869224		PSEUDOGENE CANDIDATE.			X
IPI00871452	PWWP2A	ISOFORM 1 OF PWWP DOMAIN-CONTAINING PROTEIN 2A.			X
IPI00871458	C10orf4	39 KDA PROTEIN.			X
IPI00871625	PTGER3	CDNA FLJ77072, HIGHLY SIMILAR TO HOMO SAPIENS PROSTAGLANDIN E RECEPTOR 3 (SUBTYPE EP3) (PTGER3), TRANSCRIPT VARIANT 9, MRNA.			X
IPI00872098	IKZF5	ZINC FINGER PROTEIN PEGASUS.			X
IPI00872208	KIAA1856	122 KDA PROTEIN.			X
IPI00872354	ARMC2	ARMADILLO REPEAT CONTAINING 2.			X
IPI00872730	MACF1	MACROPHIN 1 ISOFORM.			X
IPI00872928	ANKRD42	SEVERAL ANKYRIN REPEAT PROTEIN TRANSCRIPT VARIANT 2 (FRAGMENT).			X
IPI00873139	B4GALT3	ISOFORM 2 OF BETA-1,4-GALACTOSYLTRANSFERASE 3.			X
IPI00873348	A1CF	63 KDA PROTEIN.			X
IPI00873544		UNCHARACTERIZED PROTEIN ENSP00000380627 (FRAGMENT).			X
IPI00873759		UNCHARACTERIZED PROTEIN ENSP00000381675 (FRAGMENT).			X
IPI00874276	EPB41L4B	PUTATIVE UNCHARACTERIZED PROTEIN DKFZP761N1814.			X
IPI00876910	ASB18	ISOFORM 1 OF ANKYRIN REPEAT AND SOCS BOX-CONTAINING PROTEIN 18.			X
IPI00877627	NFAT5	NUCLEAR FACTOR OF ACTIVATED T-CELLS 5 ISOFORM D.			X
IPI00878015		12 KDA PROTEIN.			X
IPI00878142		68 KDA PROTEIN.			X

Table S1. Proteins Identified or Not in MS Patients Compared to Normals and Other Neurologic Disease (OND)					
**IPI**	**Gene**	**Protein Name**	**MS**	**Normal**	**OND**
IPI00878369		12 KDA PROTEIN.			X
IPI00878483		30 KDA PROTEIN.			X
IPI00879159		9 KDA PROTEIN.			X
IPI00879162		UNCHARACTERIZED PROTEIN ENSP00000374866.			X
IPI00879277	TRRAP	436 KDA PROTEIN.			X
IPI00879389	ZMYND11	20 KDA PROTEIN.			X
IPI00883751		RNA-DIRECTED DNA POLYMERASE (REVERSE TRANSCRIPTASE), RELATED DOMAIN CONTAINING PROTEIN.			X
IPI00883929		CONSERVED HYPOTHETICAL PROTEIN.			X
IPI00883997	CCDC19	COILED-COIL DOMAIN-CONTAINING PROTEIN 19.			X
IPI00884076		ENV POLYPROTEIN (COAT POLYPROTEIN) FAMILY PROTEIN.			X
IPI00884216		RNA-DIRECTED DNA POLYMERASE (REVERSE TRANSCRIPTASE), RELATED DOMAIN CONTAINING PROTEIN.			X
IPI00884334	LOC729968	CONSERVED HYPOTHETICAL PROTEIN.			X
IPI00884371		UNCHARACTERIZED PROTEIN ENSP00000383215.			X
IPI00884399		RNA-DIRECTED DNA POLYMERASE (REVERSE TRANSCRIPTASE), RELATED DOMAIN CONTAINING PROTEIN.			X
