# Supplementary material for: Gray Matter Is Targeted in First-Attack Multiple Sclerosis
Source: PLoS One. 2013 Sep 10;8(9):e66117. doi: 10.1371/journal.pone.0066117 (PMC3769274; doi:10.1371/journal.pone.0066117)
Supplement: Table S2 — Proteins with significant difference in abundance by ANOVA (p-value <0.05). (PDF) [file pone.0066117.s002.pdf]

Supplemental Table S2. Proteins with significant difference in abundance by ANOVA (p-value <0.05)					
			Avg Protein Abundance (log 2)		
**IPI**	**Gene**	**Name**	**Control**	**CIS**	**RR**
IPI00001952	ENDOD1	ENDONUCLEASE DOMAIN-CONTAINING 1 PROTEIN PRECURSOR.	25.05	24.65	25.05
IPI00002147	CHI3L1	CHITINASE-3-LIKE PROTEIN 1 PRECURSOR.	25.29	25.15	26.06
IPI00006601	CHGB	SECRETOGRANIN-1 PRECURSOR.	25.59	26.52	25.87
IPI00008318	EPHA4	EPHRIN TYPE-A RECEPTOR 4 PRECURSOR.	26.30	26.87	26.03
IPI00008997	WFDC1	WAP FOUR-DISULFIDE CORE DOMAIN PROTEIN 1 PRECURSOR.	22.27	19.70	19.91
IPI00011140	NOV	PROTEIN NOV HOMOLOG PRECURSOR.	25.34	25.22	24.91
IPI00011302	CD59	CD59 GLYCOPROTEIN PRECURSOR.	27.52	27.52	27.28
IPI00011605	CBLN1	CEREBELLIN-1 PRECURSOR.	23.05	23.74	22.62
IPI00012887	CTSL1	CATHEPSIN L1 PRECURSOR.	24.94	24.87	24.68
IPI00013303	LSAMP	LIMBIC SYSTEM-ASSOCIATED MEMBRANE PROTEIN PRECURSOR.	25.22	25.18	24.52
IPI00014048	RNASE1	RIBONUCLEASE PANCREATIC PRECURSOR.	27.87	27.78	27.16
IPI00016334	MCAM	ISOFORM 1 OF CELL SURFACE GLYCOPROTEIN MUC18 PRECURSOR.	24.24	24.47	23.62
IPI00019568	F2	PROTHROMBIN PRECURSOR (FRAGMENT).	26.54	26.56	26.49
IPI00019580	PLG	PLASMINOGEN PRECURSOR.	24.76	25.22	25.01
IPI00021842	APOE	APOLIPOPROTEIN E PRECURSOR.	26.78	27.50	26.01
IPI00022392	C1QA	COMPLEMENT C1Q SUBCOMPONENT SUBUNIT A PRECURSOR.	21.31	22.39	21.19
IPI00023845	KLK6	KALLIKREIN-6 PRECURSOR.	28.97	28.32	28.20
IPI00024966	CNTN2	CONTACTIN-2 PRECURSOR.	24.38	24.24	24.07
IPI00025257	SEMA7A	SEMAPHORIN-7A PRECURSOR.	23.82	24.12	23.32
IPI00026104	IDS	ISOFORM LONG OF IDURONATE 2-SULFATASE PRECURSOR.	23.33	23.28	22.67
IPI00027166	TIMP2	METALLOPROTEINASE INHIBITOR 2 PRECURSOR.	24.51	24.65	23.69
IPI00029235	IGFBP6	INSULIN-LIKE GROWTH FACTOR-BINDING PROTEIN 6 PRECURSOR.	27.69	27.44	26.68
IPI00029739	CFH	ISOFORM 1 OF COMPLEMENT FACTOR H PRECURSOR.	23.99	24.07	23.54
IPI00031769	C2orf40	ESOPHAGEAL CANCER-RELATED GENE 4 PROTEIN PRECURSOR.	23.85	24.67	24.04
IPI00156243	PLGLA1	PLASMINOGEN-RELATED PROTEIN A PRECURSOR.	24.77	24.57	24.41
IPI00163563	PEBP4	PEBP FAMILY PROTEIN PRECURSOR.	23.42	23.58	23.27
IPI00166729	AZGP1	ALPHA-2-GLYCOPROTEIN 1, ZINC.	24.65	24.11	24.10
IPI00168358	RGMB	RGM DOMAIN FAMILY, MEMBER B ISOFORM 2 PRECURSOR.	23.02	23.30	22.68
IPI00169276		TRYPSINOGEN C.	25.99	26.99	25.62
IPI00176221	NEGR1	NEURONAL GROWTH REGULATOR 1 PRECURSOR.	25.18	25.41	24.70
IPI00186736	IGSF8	ISOFORM 3 OF IMMUNOGLOBULIN SUPERFAMILY MEMBER 8 PRECURSOR.	25.47	24.67	25.14

Supplemental Table S2. Proteins with significant difference in abundance by ANOVA (p-value <0.05)					
			Avg Protein Abundance (log 2)		
**IPI**	**Gene**	**Name**	**Control**	**CIS**	**RR**
IPI00215631	VCAN	ISOFORM VINT OF VERSICAN CORE PROTEIN PRECURSOR.	22.53	21.49	20.56
IPI00216641	CNTN1	ISOFORM 2 OF CONTACTIN-1 PRECURSOR.	24.09	24.44	23.98
IPI00219029	GOT1	ASPARTATE AMINOTRANSFERASE, CYTOPLASMIC.	22.77	24.00	23.27
IPI00242956	FCGBP	IGGFC-BINDING PROTEIN PRECURSOR.	23.61	23.10	22.52
IPI00247243		31 KDA PROTEIN.	23.28	23.14	21.72
IPI00296608	C7	COMPLEMENT COMPONENT C7 PRECURSOR.	26.63	26.92	26.69
IPI00299738	PCOLCE	PROCOLLAGEN C-ENDOPEPTIDASE ENHANCER 1 PRECURSOR.	26.21	25.31	24.97
IPI00301395	CPVL	PROBABLE SERINE CARBOXYPEPTIDASE CPVL PRECURSOR.	22.47	21.79	21.54
IPI00305380	IGFBP4	INSULIN-LIKE GROWTH FACTOR-BINDING PROTEIN 4 PRECURSOR.	23.26	23.24	22.63
IPI00383751		CALRETICULIN=CALCIUM BINDING PROTEIN (FRAGMENT).	24.04	24.34	23.55
IPI00412924	APP	ISOFORM APP751 OF AMYLOID BETA A4 PROTEIN PRECURSOR (FRAGMENT).	27.95	28.01	27.63
IPI00426071	KLK6	KALLIKREIN 6 VARIANT 4.	25.72	24.03	25.20
IPI00445227	MCAM	ISOFORM 2 OF CELL SURFACE GLYCOPROTEIN MUC18 PRECURSOR.	21.94	22.91	22.02
IPI00513767	PTGDS	PROSTAGLANDIN D2 SYNTHASE 21KDA.	29.18	31.44	30.81
IPI00515041	CFH	UNCHARACTERIZED PROTEIN CFH.	25.95	26.25	25.54
IPI00554656	DGCR2	PUTATIVE UNCHARACTERIZED PROTEIN DKFZP686I1730.	22.15	21.91	21.37
IPI00555812	GC	VITAMIN D-BINDING PROTEIN PRECURSOR.	25.69	26.06	26.14
IPI00607600	APLP1	AMYLOID PRECURSOR-LIKE PROTEIN 1 ISOFORM 1 PRECURSOR.	27.68	27.65	27.26
IPI00640525	CTSA	CATHEPSIN A PRECURSOR.	21.76	21.81	21.67
IPI00642045	CNDP1	14 KDA PROTEIN.	27.18	26.42	26.59
IPI00645710	NEGR1	NEGR1 PROTEIN.	26.48	26.83	26.39
IPI00645849	ECM1	EXTRACELLULAR MATRIX PROTEIN 1.	25.16	25.01	24.72
IPI00647027	CHGB	32 KDA PROTEIN.	22.85	23.47	22.28
IPI00743517	PTPRS	PROTEIN TYROSINE PHOSPHATASE, RECEPTOR TYPE, SIGMA ISOFORM 2 PRECURSOR.	21.97	22.06	21.68
IPI00743766	FETUB	FETUIN-B PRECURSOR.	22.12	23.52	22.88
IPI00748395	SEZ6	SEIZURE RELATED 6 HOMOLOG ISOFORM 2.	23.42	23.10	22.73
IPI00749466	CNDP1	35 KDA PROTEIN.	27.96	27.40	27.79
IPI00788096	PRRT3	ISOFORM 3 OF PROLINE-RICH TRANSMEMBRANE PROTEIN 3 PRECURSOR.	21.97	22.97	22.41
IPI00788189	FCGBP	SIMILAR TO FC FRAGMENT OF IGG BINDING PROTEIN.	24.88	24.15	23.98
IPI00789078	SOD1	UNCHARACTERIZED PROTEIN SOD1.	24.12	24.07	23.78

Supplemental Table S2. Proteins with significant difference in abundance by ANOVA (p-value <0.05)					
			Avg Protein Abundance (log 2)		
**IPI**	**Gene**	**Name**	**Control**	**CIS**	**RR**
IPI00790679	C1S	21 KDA PROTEIN.	25.11	24.70	23.90
IPI00795153	CFI	43 KDA PROTEIN.	24.51	25.03	24.19
IPI00795624	NELL2	CEREBRAL PROTEIN-12.	25.09	25.78	25.17
IPI00796888	LUM	26 KDA PROTEIN.	25.55	25.72	25.34
IPI00797025	PRNP	MAJOR PRION PROTEIN.	25.40	25.17	24.19
IPI00828004	COL18A1	MULTI-FUNCTIONAL PROTEIN MFP.	22.28	21.55	20.10
IPI00829813	PIK3IP1	ISOFORM 2 OF PHOSPHOINOSITIDE-3-KINASE-INTERACTING PROTEIN 1 PRECURSOR.	25.41	26.20	25.02
IPI00844536	RBP4	UNCHARACTERIZED PROTEIN RBP4.	27.36	27.10	27.18
IPI00847589	RELN	REELIN ISOFORM B.	22.10	22.09	21.69
IPI00853537	CPE	20 KDA PROTEIN.	25.60	25.88	25.37
IPI00855821		NRXN1-ALPHA.	24.47	24.54	24.08
IPI00855913	PLG	PLASMINOGEN.	26.69	26.66	26.84
IPI00855916		TRANSTHYRETIN.	30.38	30.60	29.95
IPI00868938		BETA-2-MICROGLOBULIN.	29.18	28.87	28.39
IPI00871339	CACNA2D2	129 KDA PROTEIN.	23.93	23.57	23.93
IPI00872363	PTPRD	PTPRD PROTEIN.	22.73	22.88	22.17
IPI00872555	CFI	CDNA FLJ76262, HIGHLY SIMILAR TO HOMO SAPIENS I FACTOR (COMPLEMENT) (IF), MRNA.	23.78	24.05	23.78
IPI00873016	NRXN3	ISOFORM 2 OF NEUREXIN-3-ALPHA PRECURSOR.	25.47	25.46	24.85
IPI00873030	KIAA2026	UNCHARACTERIZED PROTEIN KIAA2026.	26.71	25.61	25.67
IPI00873201	PSAP	ISOFORM SAP-MU-6 OF PROACTIVATOR POLYPEPTIDE PRECURSOR.	24.81	24.40	24.26
IPI00873210	FN1	263 KDA PROTEIN.	22.88	22.55	22.05
IPI00877967	F2	36 KDA PROTEIN.	27.83	28.20	27.78
IPI00878772	C1S	19 KDA PROTEIN.	25.54	25.30	24.81
IPI00879319		260 KDA PROTEIN.	24.74	24.93	24.56
IPI00880148	LGALS1	4 KDA PROTEIN.	20.62	18.31	19.74
